# Nitrile Oxide, Alkenes, Dipolar Cycloaddition, Isomerization and Metathesis Involved in the Syntheses of 2-Isoxazolines

**DOI:** 10.3390/molecules28062547

**Published:** 2023-03-10

**Authors:** Stanisław Krompiec, Piotr Lodowski, Aneta Kurpanik-Wójcik, Bogumiła Gołek, Angelika Mieszczanin, Aleksandra Fijołek, Marek Matussek, Klaudia Kaszuba

**Affiliations:** 1 Institute of Chemistry, Faculty of Science and Technology, University of Silesia, Bankowa 14, 40-007 Katowice, Poland; 2 Department of Chemical Organic Technology and Petrochemistry, Faculty of Chemistry, Silesian University of Technology, B. Krzywoustego 4., 44-100 Gliwice, Poland

**Keywords:** 2-isoxazolines, nitrile oxide, 1,3-dipolar cycloaddition, allyl compounds, reaction mechanism, isoxazolines in pharmacy, syntheses, isoxazolines in organic synthesis, isoxazolines in agriculture

## Abstract

The involvement of 1,3-dipolar cycloaddition (1,3-DP), double bond migration, metathesis, and nitrile oxide (including in situ-generated nitrile oxide) as dipoles, together with the C=C bond containing dipolarophiles, in the syntheses of 2-isoxazolines is presented. Methods for synthesizing isoxazolines (other than 1,3-DP cycloaddition) were also presented briefly. Various methods of nitrile oxide preparation, especially in situ-generated procedures, are presented. Special attention was paid to the application of various combinations of 1,3-DP cycloaddition with double bond migration (DBM) and with alkene metathesis (AM) in the syntheses of trisubstituted isoxazolines. Allyl compounds of the type QCH_2_CH=CH_2_ (Q = ArO, ArS, Ar, and others) play the role of dipolarophile precursors in the combinations of DPC mentioned, DBM and AM. Mechanistic aspects of cycloadditions, i.e., concerted or stepwise reaction mechanism and their regio- and stereoselectivity are also discussed from experimental and theoretical points of view. Side reactions accompanying cycloaddition, especially nitrile oxide dimerization, are considered. 2-Isoxazoline applications in organic synthesis and their biological activity, broad utility in medicine, agriculture, and other fields were also raised. Some remaining challenges in the field of 1,3-DP cycloaddition in the syntheses of isoxazolines are finally discussed.

## 1. Introduction

2-Isoxazolines, i.e., 3,4-dihydro-1,2-oxazoles, play a crucial role as substrates in organic synthesis, ligands in catalysis, and above all, in medicinal chemistry and pharmacology due to their biological activity. Undoubtedly, the broad spectrum of biological activity of isoxazolines arouses the most significant interest of scientists and industries—pharmacy, agriculture, and others. The discovery of isoxazolines of natural origin contributed to the constant growth of interest in isoxazolines and their hybrids with other carbo- and heterocycles. Introductions of almost all publications, especially those devoted to the biological activity of isoxazolines, begin with a list of isoxazolines of natural origin. In each work, especially those from the last 20 years, isoxazolines are also mentioned which are currently used as drugs, pesticides, and ligands in catalytic systems, to name a few applications. That is why the methods of isoxazolines synthesis are so important and constantly evolving; new ones are sought based on new synthetic ideas, not only on 1,3-DP cycloaddition.

The importance of isoxazolines is perfectly shown in the article by Dr. Sandeep Juneja (Business Head, Animal Health Industry Professional) published on 25 May 2022 [1]. The author states: “Over the past 40 years in the animal health industry, three molecule families in the antiparasitics space have brought much needed succor to pets. These molecules have become super blockbuster product groups (exceeding $1 billion in annual sales). These three molecule families still co-exist to this date and maintain significant sales stature all at the same time. Isoxazolines have been a phenomenally successful story—even more so than the previous advances made by macrocyclic lactones (such as ivermectin and other ‘mectins’) and phenylpyrazoles (fipronil). Isoxazolines recorded cumulative sales exceeding an estimated $2.5 bn in 2021 and, with strong signs of future growth, remain the most lucrative animal health industry opportunity. While the first isoxazoline molecule—Afoxalaner—will soon be celebrating a decade of phenomenal commercial triumph in 2023, the real success story of these molecules is only just unfolding. Smart product lifecycle management initiated by the industry’s top four businesses (Zoetis, Merck Animal Health, Boehringer Ingelheim Animal Health and Elanco) has combined various endoparasiticides to broaden the compounds’ spectrum of efficacy. The companion animal parasiticides market is estimated to be worth $6.2 bn in 2021 with an annual growth rate of 8.3% year-on-year. Isoxazolines recorded around $2.5 bn of cumulative revenues in 2021 and now represent over 40% share of the companion animal parasiticides market—a dominance not seen before with macrocyclic lactones and phenylpyrazoles.”

**Figure 1:**
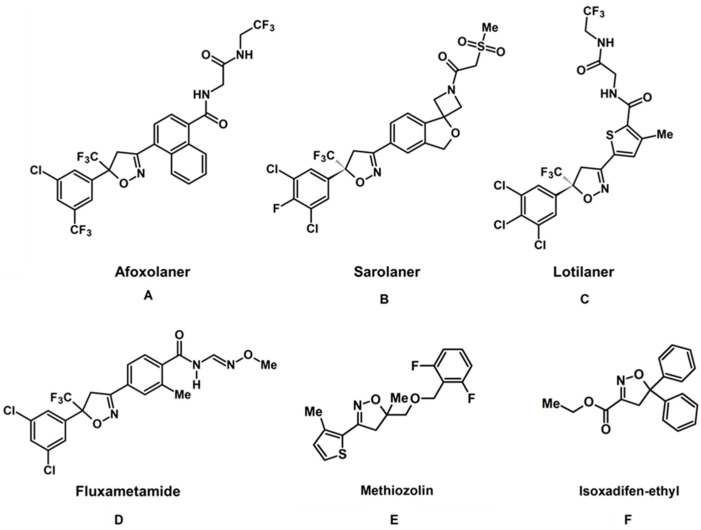
Examples of commercially available isoxazolines: insecticides (**A**–**D**) and herbicides (**E**,**F**).

Below (in Figure 1), we present examples of commercially available isoxazolines (insecticides and herbicides) that are particularly profitable.

This review discusses the methods of obtaining 2-isoxazolines via nitrile 1,3-dipolar cycloaddition to the carbon–carbon double bond, namely mono-, di-, up to tetrasubstituted double bonds. This type of cycloaddition was realized for the first time in 1992, and this important scientific event is recalled in many papers, including reviews and books—see references quoted in this work (especially in Section 3, Section 4 and Section 6). Other methods of obtaining 2-isoxazolines (without the step of 1,3-dipolar cycloaddition of RCNO to the carbon–carbon double bond), e.g., from α-nitroketones, organometallics, are briefly presented. However, virtually all “other methods” are listed, and numerous references discuss these other methods. It should be added that the review discusses publications and patents devoted to the synthesis of isoxazolines via 1,3-DP cycloaddition of nitrile oxide starting from the 21st century. Regarding patents, selected ones with non-routine procedures or fascinating structures were obtained. Nevertheless, it is obvious that the wide range of patent claims limits the scope of novelty of other works and, above all, the possibility of the commercial use of the obtained isoxazolines (compounds in general). Much space was devoted to the biological activity of isoxazolines because the biological activity of properly designed compounds was the goal of most of the work. However, since our review focuses on the synthesis via 1,3-DP cycloaddition, the results of the biological evaluation are presented without detailed analysis. That has been the subject of many quoted reviews.

Our review is multifaceted as it contains information about the synthesis via cycloaddition (primarily), information about other important synthesis methods, mechanistic considerations and the applications of isoxazolines and other compounds, as well as books and other literature in the introduction and the introductions to subsections. We have added all the necessary references in the most appropriate places, which makes our work more reader-friendly.

Importantly, when commenting on cycloaddition methods other than 1,3-DP, we emphasize that their importance is constantly growing because they are usually regio-, stereo-, and enantioselective. Moreover, thanks to these different methods, most often developed recently, it is possible to synthesize isoxazolines that cannot be obtained via 1,3-DP cycloaddition. Therefore, when designing structures and selecting synthesis methods for the expected target structures, it is necessary to know other possibilities besides 1,3-DP cycloaddition.

## 2. Syntheses of Substituted 2-Isoxazolines via 1,3-DP Cycloaddition of Nitrile Oxide to a Carbon–Carbon Double Bond

The literature on the synthesis is discussed in the following order: monosubstituted (3- and others), disubstituted (3,5-, 5,5-, and others), trisubstituted (3,4,5-, 3,5,5-, and others), and tetrasubstituted (3,4,4,5-, and others) isoxazolines. We used the division of the reaction due to the type of synthesized isoxazoline, i.e., the number and location of substituents in the 2-isoxazoline scaffold. In our opinion, this is the most important division for those who design new structures. The mechanism is the same for all reactions, namely 1,3-DP cycloaddition of RCNO into the carbon–carbon double bond. Of course, catalysts (for instance, Mg, Ir-complexes) and auxiliaries presented in the substrates (dipolarophiles or dipoles) impact the interaction between RCNO and dipolarophiles. In addition, the 1,3-DP cycloaddition may be concerted or staged, further complicating different ways of presenting the state of the art when compared to the approach adopted in this review. In conclusion, we tried to present the state of the art so that the reader would quickly find an answer to the question of how to obtain the designed isoxazoline using 1,3-DP cycloaddition as a synthetic tool. Furthermore, if the reader does not find the answer among the methods of synthesis using 1,3-DP, this review offers supplementary insights on other known possibilities.

### 2.1. 3-Substituted 2-Isoxazolines

The synthesis of 3-substituted isoxazoline via dipolar cycloaddition of nitrile oxide containing a bicycloheptene motif with ethene was described in the European patent from 2002—Scheme 1 [2].

In the following patent from the same year, the synthesis of 3-substituted isoxazoline via dipolar cycloaddition of nitrile oxide containing a (*E*)-1-propenyl motif with ethene was shown—Scheme 2. Nitrile oxide was in situ typically generated from the appropriate oxime [3].

A group of researchers obtained 3-substituted 2-isoxazolines in 2005 via 1,3-DP cycloaddition of nitrile oxides or dioxide-dinitrile to ethylene, which was bubbled through the reaction mixture—Scheme 3 [4].

### 2.2. 3,4-Disubstituted 2-Isoxazolines

**Figure 2:**
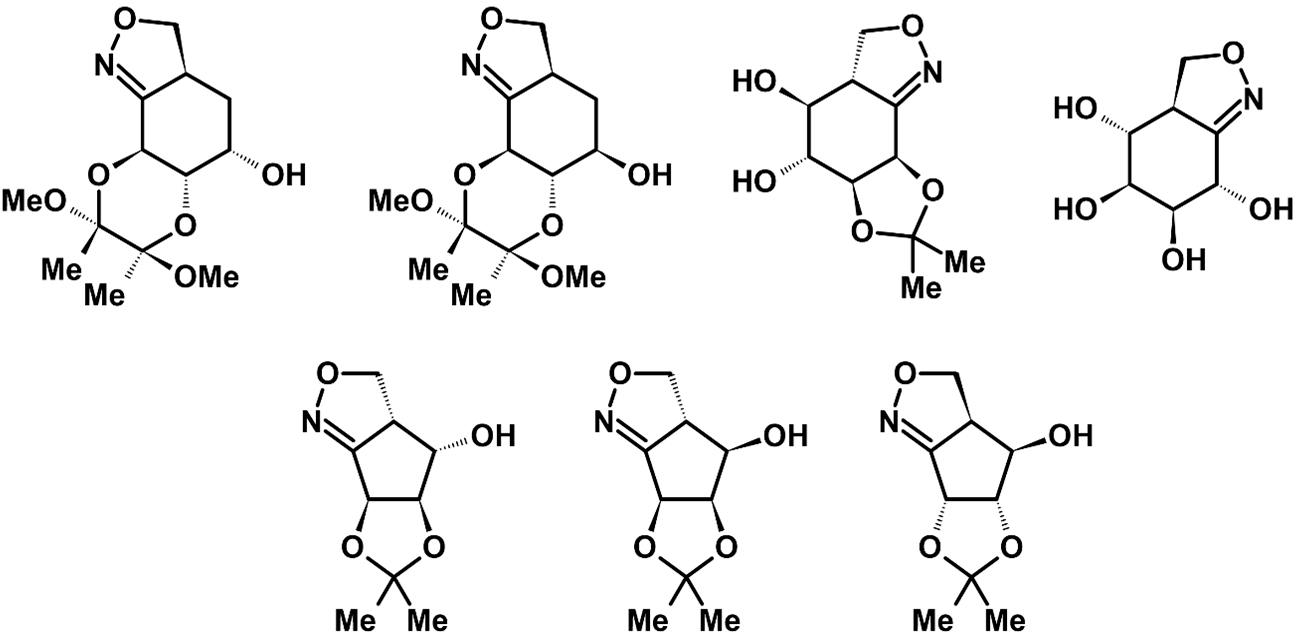
The selected products obtained via INOC—see scheme above.

In 2007, 3,4-disubstituted, highly functionalized, 2-isoxazolines were synthesized by the INOC (intramolecular nitrile oxide cycloaddition) strategy—Scheme 4 and Figure 2 [5]. All obtained products contained an isoxazoline moiety fused with sugar-derived fragments. Notably, the substrates possessed from one to four unmasked hydroxyl groups.

The synthetic procedure leading to 3,4-disubstituted 2-isoxazolines via intramolecular dipolar cycloaddition of a nitrile oxide motif to the double bond coming from the allyloxy group present in the substrate was described in an article from 2010—Scheme 5 [6]. This attempt was carried out in an aqueous environment. The nitrile oxide motif was generated in situ from the oxime group using [hydroxy(tosyloxy)iodo]benzene (HTIB).

The derivative of 1-naphthyl carboaldehyde oxime was also successfully used to synthesize an appropriate isoxazoline. It is worth noting that this work has been mentioned in a 2021 review work from Plumet [7].

1,3-DP cycloaddition was an initial step in the synthetic route leading to benzoylphenylureas containing an isoxazoline motif—Scheme 6 [8].

The evaluation of the larvicidal activities against the oriental armyworm, mosquito, and diamondback moth of the obtained isoxazolines (and also isoxazoles) revealed that some of them showed nearly the same level of insecticidal activity against oriental armyworm as the commercial insecticide Flucycloxuron. Surprisingly, some tested compounds exhibited much higher larvicidal activities against diamondback moth than Flucycloxuron.

### 2.3. 3,5-Disubstituted Isoxazolines

The usage of monoclanal antibody 29G12 as a catalyst achieved high enantioselectivity (up to 98% ee) in the 1,3-DP cycloaddition of *p*-AcNHC_6_H_4_CNO to *N*,*N*-dimethylacryl amide, as reported by Toker et al. in their article from 2000—Scheme 7 [9].

The analysis of activation parameters of the noncatalyzed (∆H^‡^ = 10.8 kcal/mol ∆S^‡^ = −28.1 eu) and antibody-catalyzed (∆H^‡^ = 4.0 kcal/mol and ∆S^‡^ = −38.1 eu) 1,3-DP cycloaddition revealed that 29G12 stabilizes the free energy of the transition state by a significant reduction in the activation enthalpy (6.8 kcal/mol) rather than the activation entropy.

Kanemasa and colleagues described in 2000 using [BMTA][ICl_4_] for the in situ creation of oxymoyl chloride followed by nitrile oxide generation and finally 1,3-DP cycloaddition of RCNO to CH_2_=CHR type dipolarophiles—Scheme 8 [10].

The same year, using a camphor-derived auxiliary for the 1,3-DP cycloaddition of RCNO to the double bond of the chiral *N*-acryloyl dipolarophile motif achieved highly regioselective, enantiopure isoxazolines—Scheme 9 [11].

Another article from 2000 presents the diastereoselective 1,3-DP cycloaddition of benzonitrile oxide derivatives to acrylamide-type chiral dipolarophile auxiliary. It was engaged in synthesizing chiral 3,5-disubstituted isoxazolines bearing the oxazolidinone motif in position 5—Scheme 10 [12].

It is worth mentioning that other tested additives, namely ZnI, Cu(OTf)_2_, Ni(ClO_4_)_2_, and Fe(ClO_4_)_3_, and reaction without any additive were much less selective.

A team of researchers also reported in 2000 that over 30 examples of potential antagonists of the integrin α_v_ß_3_ were synthesized starting from simple 1,3-DP cycloaddition followed by various modifications of the obtained 3,5-disubstituted—Scheme 11 [13].

Another report from 2000 describes chiral isoxazolines obtained via asymmetric 1,3-DP cycloaddition of PhCNO to methyl acrylate or styrene equipped with chiral dioxazaborocine auxiliaries—Scheme 12 [14].

Penam sulfone intermediate equipped with a CNO group was prepared in a few steps starting from commercially available (+)-6-aminopenicillanic acid. This nitrile oxide was applied for regioselective 1,3-dipolar cycloaddition reactions with various alkenes (and alkynes) to give isoxazolines (or isoxazoles) as potent ß-lactamase inhibitors—Scheme 13 [15].

The regioselectivity observed in these cycloadditions can be explained by considering the FMO. Depending on the size of the coefficients, a favorable HOMO-LUMO interaction between the dipole and the dipolarophile leads to the observed regioselectivity. The dipole (LUMO)–dipolarophile (HOMO) interaction seems dominant for alkenes. Interestingly, in vitro examinations revealed that some of the obtained compounds can be potent active agents against several ß-lactamase enzymes.

In 2001, 3,5-disubstituted 2-isoxazolines possessing a Ph-substituted (*E*)-stilbene motif (attached through -CH_2_OC(O)- linker) in position 5 were obtained via 1,3-DP cycloaddition of appropriate nitrile oxide to acrylate equipped with above-mentioned stilbene fragment—Scheme 14 [16].

As described by Simoni et al., a 3,5-disubstituted isoxazoline can be obtained in two synthetic ways, namely substituents from position 3 (and analogically from position 5) coming from nitrile oxide (the first way) or dipolarophile (the second way)—Scheme 15 [17].

In 2002, 3,5-disubstituted isoxazolines bearing an ArC(O)O- moiety in position 5 were obtained typically via regioselective 1,3-DP cycloaddition of ArCNO to allyl esters of aromatic acids—Scheme 16 [18].

Interestingly, pharmacological screening showed that 5-(4-bromobenzoyloxy)methyl-3-(3,4-dimethoxyphenyl)isoxazoline displayed marked nootropic activity.

The same year, Yong-Jia and Yan-Guang published a paper regarding the poly(ethylene glycol) (PEG)-supported dipolarophile, i.e., allyl alcohol, which was used in the synthetic route leading to the 3,5-disubstituted isoxazolines—Scheme 17 [19].

It is worth noting that an analogical procedure, i.e., supported propargyl alcohol, was applied for the syntheses of isoxazoles.

**Figure 3:**
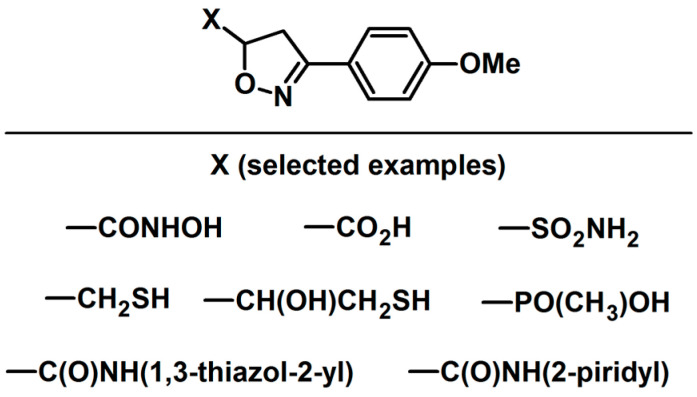
Selected (from a library containing more than 50 examples) 3–5-disubstituted isoxazolines bearing various Zn-complexing moieties in position 5 [20].

In addition, in 2002, Pirrung and colleagues obtained a library of 3,5-disubstituted isoxazolines bearing diverse Zn-binding motifs. The syntheses were typically performed starting from 1,3-DP cycloaddition of *p*-MeC_6_H_4_CNO to various dipolarophiles—Figure 3 [20]. Some of the obtained isoxazolines underwent X-group modification in the following steps.

These comprehensive studies allowed the detection of a new class of inhibitors effective in inhibiting the LpfC enzyme, namely 3,5-disubstituted isoxazolines equipped with a Zn-binding moiety in position 5. LpxC is a zinc amidase that catalyzes the second step of lipid A biosynthesis in Gram-negative bacteria.

The same year, some ROCH=CH_2_ type vinyl ethers (R = sugar motif) were used as chiral templates responsible for asymmetric induction in the syntheses of substituted isoxazolines via 1,3-DP cycloaddition of nitrile oxides to ROvinyl—Scheme 18 [21].

Kim and fellow researchers attempted regio- and diastereoselective 1,3-DP cycloaddition of RCNO to magnesium alkoxide of 1,5-hexadien-3-ol. It was involved in the synthetic route to 2-cyanomethyl-3-hydroxy-5-iodomethyl-tetrahydrofuran. This compound was reached after treating syn-5-(1-hydroxy-3-butenyl)isoxazolines obtained in the first step with ICl—Scheme 19 [22].

It is worth mentioning that 2,5-disubstituted 3-hydroxytetrahydrofuran derivatives are important skeletons in many natural compounds such as muscarines, halichondrin B, (+)-*trans*-kumausyne, palytoxin, (+)-furanomycin, and leukotrienes.

**Figure 4:**
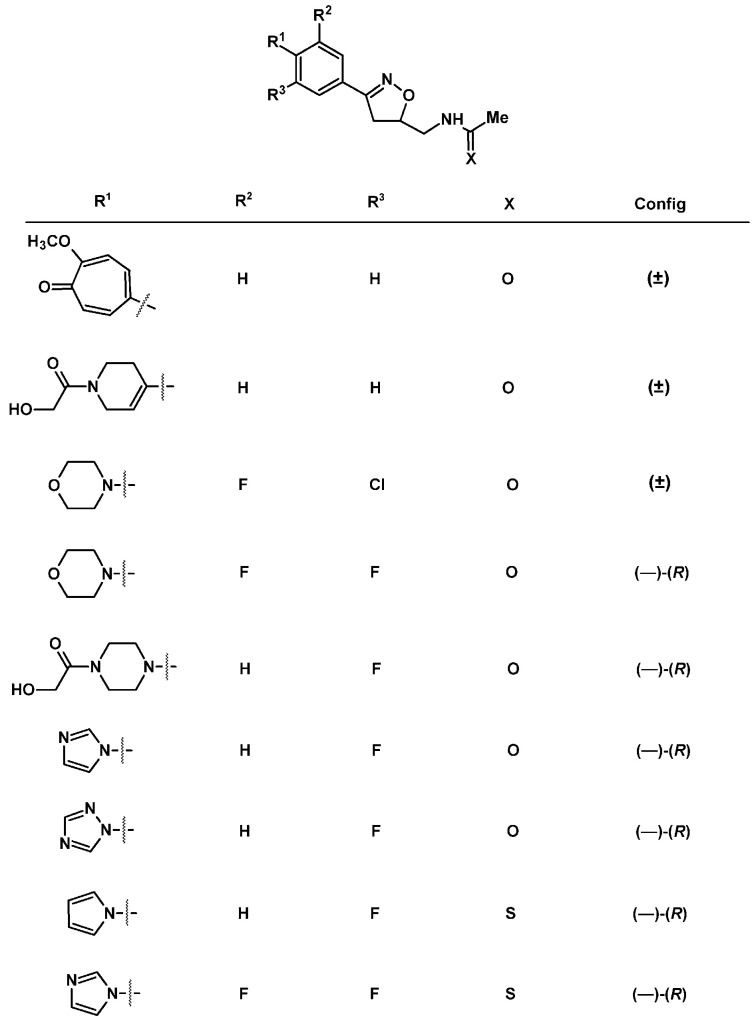
3,5-disubstituted isoxazolines bearing aryl substituent in position 3 and -CH_2_NHC(O or S)Me group in position 5 [23].

A library of 3,5-disubstituted isoxazolines, presented in Figure 2, was obtained using 1,3-DP cycloaddition and classical procedures in 2003 by a group of researchers—Figure 4 [23].

Importantly, all obtained isoxazolines were active against Gram-positive pathogens, and one of the evaluated compounds, a piperazine–isoxazoline hybrid, exhibited a balance of in vitro activity and in vivo efficacy comparable to the clinically used linezolid.

Later that year, Sieburth and colleagues reported that 1,3-DP cycloaddition of *p*-ClC_6_H_4_CNO to chiral allyl silane (95% optical purity) provided a chiral 3,5-disubstituted isoxazoline—Scheme 20 [24].

As Conti et al. reported, the application of ionic liquids, i.e., [BMIM][BF_4_] and [BMIM][PF_6_], allowed 1,3-DP cycloaddition of relatively unstable nitrile oxides (for instance, carboethoxyformonitrile oxide) to different alkenes, and 2-isoxazolines were eventually obtained—Scheme 21 [25].

Other chiral 3,5-disubstituted isoxazolines were obtained via 1,3-DP cycloaddition of RCNO to chiral 3-buten-2-ol—Scheme 22 [26].

Notably, highly regio- and stereoselective 1,3-DP cycloaddition presented in the scheme above can be applied to stereoselective syntheses of some diols and ß-amino acids.

Kociolek and Hongfa described diastereoselective 1,3-DP cycloaddition of PhCNO to homoallylic alcohols mediated by Mg^2+^ leading to 3,5-disubstituted 2-isoxazolines—Scheme 23 [27]. Interestingly, the reaction between PhCNO and chiral homoallylic alcohols (transformed in situ to *Mg*-alkoxides) favored the *syn* isomer of the cycloadduct.

It is worth mentioning that the classical procedure, namely PhCNO generation using Et_3_N/CH_2_Cl_2_, was less effective in cycloaddition stereo-controlling than *Mg*-mediation. Moreover, molecular modelling examination was consistent with the experiments and demonstrated that the transition state leading to the syn isomer is characterized by slightly lower energy (2.36 kcal/mol) than the transition state leading to the anti-adduct.

Carbohydrate derivatives as chiral auxiliaries were used in the regio- and stereoselective 1,3-DP cycloadditions of PhCNO and Me_3_CCNO to methyl 4-*O*-acryloyl-6-deoxy-2,3-di-*O*-(*t*-butyl-dimethylsilyl)-α-D-glucopyranoside. The expected 3,5-disubstituted isoxazolines were obtained after removing the carbohydrate template from carbohydrate–isoxazoline hybrids, as reported by Tamai et al.—Scheme 24 [28].

Ionic liquid, namely [BMIM][BF_4_], was described in a 2003 article as an effective solvent in the syntheses of isoxazolines bearing amido and ester moiety or two amido motifs in positions 3 and 5—Scheme 25 [29]. Importantly, ionic liquid recycling is possible using a special purification procedure.

Specially designed as orally bioavailable factor Xa inhibitors, 3,5-disubstituted isoxazolines were obtained in 2003 by Lam and colleagues. The synthesis was realized via 1,3-DP cycloaddition of ArCNO to acrylamide type dipolarophile tethered to Kaiser oxime resin—Scheme 26 [30].

All obtained compounds were scanned for novel ligands, and 4-chloro-3-aniline was identified as a novel and potent benzamidine mimic.

A series of erytromycin A derivatives containing isoxazoline motifs were synthesized and evaluated against various erythromycin-resistant pathogens. Derivatives equipped with an isoxazoline motif were obtained via 1,3-DP cycloaddition followed by deprotection of the OBz group—Scheme 27 [31].

In vitro evaluation revealed that isoxazoline-containing derivatives showed weak activity against *erm*-resistant *S. pyogenes* and *S. pneumoniae*. However, they presented excellent activity against inducibly *erm*-resistant *S. aureus* and both *mef*-resistant *S. pneumoniae* and *S. pyogenes*.

3-Aryl isoxazoles were obtained by Sheng et al. via 1,3-DP cycloaddition of in situ-generated ArCNO to PhSeCH=CH_2_ followed by oxidative elimination of PhSeOH—Scheme 28 [32].

Unfortunately, cycloaddition was not regioselective if the PhSeCH=CHR (R = Me or Ph) were used instead of PhSeCH=CH_2_. This kind of result, i.e., lack of regioselectivity, is confirmed in our later experiments and DFT calculations [33].

(*S*)-5-carboxymethyl-3-(4-cyanophenyl)-2-isoxazoline is a key intermediate in the synthesis of Roxifiban. In 2004, Pesti et al. reported obtaining this molecule typically: 1,3-DP cycloaddition, followed by enzymatic dynamic kinetic resolution on a large scale—Scheme 29 [34]. More research has shown that the technology can be improved by using S-*i*Bu ester instead of O-*i*Bu ester.

Roxifiban is a potent and selective platelet glycoprotein IIb/IIIa receptor antagonist. Due to this fact, this isoxazoline derivative holds substantial promise for the prevention and treatment of a wide variety of thrombotic diseases resulting from undesired platelet adhesion, such as stroke, acute myocardial infarction, transient ischemic attack, and unstable angina.

Other 3,5-disubstituted 2-isoxazolines were also obtained in 2004. To this end, Alksnis and fellow researchers performed regioselective 1,3-DP cycloaddition of in situ-generated benzonitrile oxide and their substituted derivatives to allyl-aryl ethers—Scheme 30 [35].

Regioselective 1,3-DP cycloaddition of MesCNO to *N*- and *C*-allylpyrazolo-1,5-benzodiazepines was involved in the synthetic route leading to the isoxazolinylmethyl-pyrazolo [1,5,4-*ef*][1,5]benzodiazepinones hybrids, as reported by Bouissane and colleagues the same year—Scheme 31 [36].

As found in another article from 2004, 1,3-DP cycloaddition was a crucial step in a synthetic method leading to a series of oxazolidinone antibacterial agents containing at the carbon C-5n a substituted isoxazol-3-yl moiety—Scheme 32 [37].

In vitro evaluation against a panel of susceptible and resistant Gram-positive organisms showed that several tested isoxazoline–oxazolidine hybrids from this series were comparable or more effective than linezolid.

Alternating 3,5-disubstituted isoxazolines were obtained in 2005 by Xu et al. using allyl selenium as dipolarophile and in situ-generated nitrile oxides, supported on polystyrene—Scheme 33 [38].

Importantly, the selenium dipolarophile (supported on the polymer) can be easily recycled in this cleavage protocol. Moreover, selected isoxazolines were smoothly transformed in the 5-methyl-3-arylisoxazoles in the reaction with DBU or NaCN (in DMF, 80 °C).

In another report, Xu and colleagues described 3,5-disubstituted isoxazolines possessing a CH_2_SePh group in position 5. They were obtained via regioselective 1,3-DP cycloaddition of RC_6_H_4_CNO to allyl-phenyl selenide—Scheme 34 [39].

Chiacchio et al., also in 2005, synthesized 3,5-disubstituted isoxazolines bearing a polyhedral oligomeric silsesquioxane (POSS) motif in position 5 via regioselective 1,3-DP cycloaddition of in situ-generated EtOOCCNO to RCH=CH_2_ type monomers (R = POSS motif)—Scheme 35 [40].

As found in an article from Conti and fellow researchers, 1,3-DP cycloaddition was engaged in synthesizing all stereoisomers of 5-(2-amino-2-carboxyethyl)-4,5-dihydroisoxazole-3-carboxylic acid—Scheme 36 [41].

Two pairs of enantiomeric isoxazolines were obtained via cycloaddition and subsequently resolved using enzymatic procedures. Moreover, it was proven that the pharmacological activity of pure enantiomers is strictly related to their absolute stereochemistry.

On the other hand, using chiral dipolarophiles containing two stereogenic centers allowed Trost and coworkers to obtain chiral isoxazolines with a new stereogenic center—Scheme 37 [42].

As described in an article, isoxazolines bearing a saccharin motif in position 5 were obtained via regioselective 1,3-DP cycloaddition of in situ-generated RCNO to *N*-allylsaccharin by Dirnens, Belyakov, and Lukevics—Scheme 38 [43].

Ros and coworkers synthesized chiral 3,5-di- (and also 3,4,5-tri-) substituted 2-isoxazolines via highly regio- and stereoselective 1,3-DP cycloaddition of RCNO to chiral acrylamides—Scheme 39 [44].

High regio- and stereoselectivity is an effect of excellent facial discrimination by the chiral 2,5-diphenylpyrrolidine motif that efficiently controls the stereochemical course of the 1,3-DP cycloaddition.

Clayton and Ramsden reported obtaining isoxazoline–nitroimidazole hybrids as potential purine nucleoside analogues via 1,3-DP cycloaddition of in situ-generated RCNO to *N*-vinyl nitroimidazole—Scheme 40 [45].

Other chiral 3,5-disubstituted 2-isoxazolines resulted from 29G12 antibody-mediated 1,3-DP cycloaddition of in situ-generated *p*-AcNHC_6_H_4_CNO to acrylamide derivatives—Scheme 41 [46].

A study referring to the structure of substrate tolerance in the above-mentioned reactions has revealed the presence of an unoptimized pocket that accepts a range of bulky hydrophobic dipolarophiles. Moreover, “substrate optimization” suggests that significant reorganization occurs within the active site of 29G12.

*Mg*-mediated 1,3-DP cycloaddition of in situ-generated RCNO to 1,4-pentadien-3-ol and 1,5-hexadiene-3,4-diol allowed Son et al. in 2005 to obtain *syn*,*syn*-bis(1,2-isoxazolin-5-yl)methanols and *syn*,*syn*,*syn*-1,2-bis(1,2-isoxazol-5-yl)ethane-1,2-diols, respectively—Scheme 42 [47].

The following year, Cheng and Al-Abed presented 1,3-DP cycloaddition of benzonitrile oxide derivatives to 3-butenoic acid as a starting step in a synthetic method leading to a structurally modified macrophage Migration Inhibitory Factor (MIF)—Scheme 43 [48]. MIF is a proinflammatory cytokine critically involved in the pathogenesis of inflammatory disorders.

Interestingly, (*R*)-isomer of the final product in which X = F and R = OCH_2_CMe_3_ turned out to be 20-fold more potent than ISO-1 and inhibits MIF tautomerase activity with an IC50 of 550 nM.

As Dallanoce and coworkers reported in 2006, enantiomerically pure 3-substituted-Δ2 -isoxazolin-5-yl-ethanolamines were prepared via a 1,3-DP cycloaddition of RCNO to chiral dipolarophile—Scheme 44 [49].

The obtained compounds, their corresponding 3-isopropenyl derivatives, and some isoxazole analogues were tested as far as their affinity at human ß1-, ß2 -, and ß3-adrenergic receptors are concerned. The tests unveiled that the binding affinities at the ß-ARs of the isoxazolinyl amino alcohols were significantly lower than those of the corresponding isoxazole derivatives.

The same year, Brel published a paper concerning isoxazolines equipped with functions for the next transformation (*E*)-CH_2_CH=CHSF_5_ motif. Those compounds were obtained via regioselective 1,3-DP cycloaddition of in situ-generated Y-PhCNO to a diene possessing SF_5_ group—Scheme 45 [50].

Interestingly, the substituent (*E*)-CH_2_CH=CHSF_5_ can be smoothly transformed into (*E*)-CH=CHCHCH_2_SF_5_ via double bond migration mediated by Cs_2_CO_3_ in MeOH.

Using 1,3-DP cycloaddition of in situ-generated EtOOCCNO to 1,1,1-trifluoro-2-trifluoromethylbut-3-en-2-ol, Cheng and coworkers performed a synthetic route leading to a series of 5-(1,1,1,3,3,3-hexafluoro-2-hydroxypropan-2-yl)-4,5-dihydroisoxazole-3-carboxamides as a new class of malonyl-coenzyme A decarboxylase (MCD) inhibitors—Scheme 46 [51].

All obtained isoxazolines were evaluated in vivo as a new class of malonyl-coenzyme A.

1,3-DP cycloaddition was an initial step in a synthetic method described by Conti et al. in 2007, leading to conformationally constrained homologues of glutamic acid—Scheme 47 [52].

As reported by Quadrelli and coworkers in 2008, 1,3- DP cycloaddition of in situ-generated nitrile oxide into specially designed dipolarophile was a starting point in the procedure leading to pharmacologically attractive nucleoside analogues—Scheme 48 [53].

1,3-DP cycloaddition between a specially designed dipole and dipolarophile was decisive for the stereo-controlled total syntheses of 10-epi-bengazole A belonging to a family of marine natural products that display potent antifungal activity—Scheme 49 [54].

As described in 2007 by Bigdeli, Mahdavinia, and Jafari, 3,5-disubstituted 2-isoxazolines can be successively obtained using 1,3-DP cycloaddition of Y-C_6_H_4_CNO to styrene in solvent-free conditions and using the one-pot procedure—Scheme 50 [55]. Interestingly, reaction mixture ingredients (oxime, oxone, styrene, silica gel, and Et_3_N) were ground together within 15 min and finally extracted.

In another article from 2007, Yamamoto and coworkers reported obtaining chiral 3,5-disubstituted isoxazolines. The synthesis was mediated by Lewis acid, chiral pybox ligands, and 1,3-DP cycloaddition of PhCNO to acrylamide equipped with auxiliaries—Scheme 51 [56]. Various types of Lewis acid, auxiliary motifs, and pybox derivatives were tested, and the best results, as far as *ee* is concerned, were observed for MgBr_2_-(*S*)-ip-pybox as the catalyst and ip-pybox as the auxiliary coordination motif.

(*S*,*R*)-3-Phenyl-4,5-dihydro-5-isoxazole acetic acid (VGX-1027) that exhibits various immunomodulatory properties was described in another article from 2007 by a group of researchers. The published procedure involved 1,3-DP cycloaddition of PhCNO to 3-butenoic acid—Scheme 52 [57].

Another report from Brel concerned isoxazoline–azirine hybrids equipped with the diethoxyphosphoryl motif in the azirine ring, which were obtained via 1,3-DP cycloaddition of PhCNO to 3-vinyl-2H-azirines—Scheme 53 [58].

It is worth mentioning that changing the order of the cycloaddition, i.e., creating the azirine motif first and the isoxazoline at the end, fails.

Starting from regioselective 1,3-DP cycloaddition of in situ-generated ArCNO to methyl acrylate, a library of 3-aryl-4,5-dihydroisoxazole-5-carboxamides was synthesized by Jeddeloh and coworkers—Scheme 54 [59].

The observed regioselectivities of the 1,3-DP cycloaddition reactions presented above result from both steric and electronic effects. The role of electronic factors in determining regioselectivity was considered in terms of FMO theory.

3-Aryl-5-methyl 2-isoxazolines were obtained via regioselective reaction between in situ-generated nitrile oxide and allyl indium bromide as reported by Sawant et al.—Scheme 55 [60].

According to the authors’ proposal, the reaction proceeds in stages, starting from the creation of the C–C bond and then the creation of the C–O bond.

Dirnens and his team employed regioselective 1,3-DP cycloaddition of in situ-generated ArCNO to allyltheobromine in the syntheses of (isoxazoline)-CH_2_-(3,7-dimethylxantine) hybrids—Scheme 56 [61].

In 2008, Yasuhito, Morio, and Toshikazu published an article regarding polymers containing isoxazoline motifs. They synthesized them via 1,3-DP cycloaddition of 1,3-benzodinitrile dioxide to di-acrylate or di-metacrylate of diethylene glycol—Scheme 57 [62].

In the same year, other di- and trisubstituted isoxazolines were obtained via 1,3-DP cycloaddition of sterically crowded Ph_2_CHCNO to dipolarophiles of CH_2_=CHR, RCH=CHR, and CH_2_=CR_2_ type—Scheme 58 (obtained 3,5-disubstituted isoxazolines are presented) [63].

Yuan and fellow researchers realized modifications of micromolide (a natural antituberculosis agent) by engaging 1,3-DP cycloaddition of appropriately designed dipole and dipolarophile—Scheme 59 [64].

Interestingly, most of the obtained (aliphatic chain or Q)-isoxazoline-aliphatic chain-butyrolactam hybrids showed significantly lower activities than natural micromolide, which suggests that the long aliphatic side chain is essential for their activity. However, despite the reduced in vitro activities, some moderately active micromolide analogues showed lower lipophilicities and larger polar surface area (PSA) values.

The following year, Ono et al. applied 1,3-DP cycloaddition of RCNO mediated by *Ni*-complex with chiral ligand (equipped with two chiral isoxazoline motifs) to enamide-type dipolarophile for the syntheses of chiral isoxazolines—Scheme 60 [65]. Notably, the rate of (quantitative) RCNO generation from oxymoyl chloride was controlled by molecular sieve, i.e., their type (4 Å was the best) and amount.

1,3-DP cycloaddition of in situ-generated EtOOCCNO to styrene derivatives constituted a highlight in the synthetic pathway towards 3,5-disubstituted isoxazolines possessing EtOOC substituent in position 3 and various *p*-Y-C_6_H_4_ groups attached to the C5 atom—Scheme 61 [66].

All obtained isoxazolines were evaluated as far as their antituberculosis activity is concerned, and the structure–activity relationships were thoroughly analyzed by Rakesh and coworkers.

In another article from 2009, Giannini et al. described 1,3-DP cycloaddition that led to disubstituted isoxazoline species. It was crucial in the multistep synthesis of potential histone deacetilase (HDAC) inhibitor—Scheme 62 [67]. It is worth noting that a similar synthesis was also carried out for isoxazoles using propargyl alcohol instead of allyl alcohol as a dipolarophile.

The obtained potential HDAC inhibitors based on a *N*-hydroxy-(4-oxime)-cinnamide scaffold (including isoxazoline presented in Scheme 63) were preliminarily tested as far as their in vitro cytotoxic activity on three tumor cell lines, NB4, H460 and HCT116, as well as their inhibitory activity against class I, II, and IV HDAC, are concerned. Interestingly, some evaluated derivatives demonstrated a promising inhibitory activity on HDAC6 and HDAC8 coupled with a good selectivity profile.

1,3,4-Oxadiazoles connected with a 2-isoxazoline motif via a CH_2_OCH_2_– linker were obtained starting from 1,3-dipolar cycloaddition of ArCNO to allyl alcohol, as described by Jayashankar and colleagues in their work from 2009—Scheme 63 [68].

Screening for anti-inflammatory and analgesic activities of the obtained oxadiazole-isoxazoline derivatives revealed their excellent activity comparable to ibuprofen and aspirin at similar dosages.

*Mg*-mediated and hydroxy-group-directed RCNO 1,3-DP cycloaddition to allylic alcohols, homoallylic alcohols, and monoprotected homoallylic diols allowed Lohse-Fraefel and Carreira to obtain various 3,5-disubstituted 2-isoxazolines—Scheme 64 [69,70].

The reaction mechanism, especially the influence of *Mg*-coordination, H–H and H–Me repulsion, and rehybridization of C2 and C4 atoms on regio- and anti-stereoselectivity observed in these 1,3-DP cycloadditions, was thoroughly discussed.

As reported by Mendelsohn and colleagues, other 3,5-disubstituted 2-isoxazolines can be synthesized via firstly the PhI(OAc)_2_ (DIB) oxidation of given oximes and then the regioselective 1,3-DP cycloaddition of obtained RCNO to terminal alkenes—Scheme 65 [71].

Dallanoce and coworkers published a paper about specially designed 4,5-dihydro-3-methylisoxazolyl derivatives, structurally related to epiboxidine, i.e., (1*R*,4*S*,6*S*)-6-(3-methylisoxazol-5-yl)-7-azabicyclo [2.2.1]heptane. Those molecules were obtained by 1,3-DP cycloaddition of acetonitrile oxide to different olefins—Scheme 66 [72].

The obtained compounds were tested for affinity at neuronal nicotinic heteromeric (a4b2) and homomeric (a7) acetylcholine receptors.

Thalassitis and colleagues obtained homo-*N*-nucleosides equipped with a 2-isoxazoline moiety via the 1,3-DP cycloaddition of MesCNO to 9-allyl derivatives of 6-chloro-, piperidinyl-, morpholinyl- pyrrolidinylpurine and 6-*N*,*N*-dibenzoyladenine—Scheme 67 [73].

All obtained 1,3-disubstituted 2-isoxazolines were evaluated in vitro for their ability: (a) to interact with 1,1-diphenyl-2-picryl-hydrazyl (DPPH) stable free radical; (b) to inhibit lipid peroxidation; (c) to scavenge the superoxide anion; (d) to inhibit the soybean lipoxygenase activity; (e) to inhibit in vitro thrombin. Most tested isoxazolines were found to be potent thrombin inhibitors and inhibited in vitro lipid peroxidation. Moreover, the majority of the compounds showed significant lipoxygenase inhibitory activity. Interestingly, the derivative equipped with NHCOPh substituent exhibited promising combined antioxidant–anti-inflammatory activity and thrombin inhibitory ability (100% inhibition at 100 μM). Moreover, isoxazolines equipped with *N*-pyrrolidinyl substituent presented high LO inhibitory activity combined with significant anti-thrombin activity.

Jewett, Sletten, and Bertozzi described regioselective 1,3-DP cycloaddition of *p*-hydrohydroxymethyl benzonitrile oxide to *N*-allyl biaarylazacyclooctene derivative, which was involved in the synthetic route to the isoxazoline motif containing biarylazacyclooctynone (BARAC) derivatives—Scheme 68 [74].

Interestingly, application tests have shown that obtained BARAC derivatives can be promising reagents for live cell fluorescence imaging of azide-labelled glycans. Namely, the high signal-to-background ratio obtained using nanomolar concentrations of tested isoxazoline-containing agents obviated the need for washing steps.

In 2010, the synthesis of 3-bromo- and 3-chloroisoxazolines via typical regioselective 1,3-DP cycloaddition of in situ-generated RCNO to dipolarophile was published—Scheme 69 [75].

The obtained halogenoisoxazolines inspired the design and synthesis of a natural product library of probes for bacterial proteome analysis. Furthermore, a unique preference of the probes to label and inhibit members of the dehydrogenase enzyme family was observed. The probe selectivity for a specific enzyme target was remarkable, and small structural changes, e.g., methyl vs. ethyl substitution, already discriminate binding.

1,3-DP cycloaddition of in situ-generated RCNO to ethyl acrylate was a crucial step in the synthetic route leading to 3-substituted isoxazole carboxamides described by Gucma and Gołębiewski in 2010—Scheme 70 [76].

Some of the obtained 3-substituted isoxazolecarboxamides exhibited high fungicidal activities against *Alternaria alternata, Botrytis cinerea, Rhizoctonia solani*, *Fusarium culmorum*, and *Phytophthora cactorum*. It was envisioned that a combination of electronic and steric effects influences biological activity. High biological activity can be correlated with the low electron density of the ring systems. Moreover, the degree of conjugation between the phenyl and the isoxazole rings is also significant.

Another report from Dallanoce et al. presented the enantiopure diastereomeric 2-isoxazoline–pyrrolidine hybrids, structural analogues of both ABT-418 and its oxyimino ethers. They were synthesized through 1,3-DP cycloaddition of RCNO and (*S*)-*N*-Boc-2-vinylpyrrolidine derivatives as the dipolarophile—Scheme 71 [77].

It was revealed that all considered derivatives showed drastic reduction concerning their binding affinity at nicotinic and muscarinic acetylcholine receptors. Conversely, two isomers showed an affinity for the nAChRs comparable to that observed for the model compound ABT-418. The synthesis of isoxazoline-CH_2_-pyrazole hybrids via 1,3-DP cycloaddition of in situ-generation R_3_CNO to allylpyrazole derivatives was published in another article from 2010—Scheme 72 [78].

Dow AgroSciences has broadly screened the compounds presented above for herbicidal, fungicidal, and insecticidal activity.

A series of isoxazoline- and isoxazole-based histone deacetylase (HDAC) inhibitors structurally related to SAHA were designed and obtained using 1,3-DP cycloaddition of RCNO to the appropriate dipolarophile—Scheme 73 [79]. All obtained compounds were tested as HDAC inhibitors.

Using exclusively regioselective and *Cu*-free 1,3-DP cycloaddition of styrene functionalized DNA to nitrile oxides, a series of modified oligodeoxynucleotides containing 3,5-disubstituted isoxazoline linkers were prepared and described by Gutsmiedl, Fazio, and Carell—Scheme 74 [80].

In another paper from 2010, diastereoselective 1,3-DP cycloaddition of the appropriate nitrile oxide and dipolarophile was reported as an eminent step in the total synthesis of (-)-berkeleyamide A—Scheme 75 [81].

Subsequent work from 2010 presents 3-aryl-5-thiophenyl-2-isoxazolines obtained typically, i.e., via 1,3-DP cycloaddition of ArCNO to phenyl-vinyl sulfide—Scheme 76 [82]. Significantly, some of the obtained isoxazolines were transformed into 3-arylisoxazoles via classic base-mediated elimination.

The synthesis of rotaxane equipped with a 3,5-disubstituted 2-isoxazoline moiety via click end-capping reaction between nitrile oxide, dibenzo-24-crown-8, and the appropriate dipolarophile was described by Matsumura and coworkers in other work—Scheme 77 [83].

A system analogous to that shown in Scheme 77, but containing an isoxazole moiety (triple bond played the role of dipolarophile), was also obtained and tested as a pH-driven molecular shuttling system.

3,5-disubstituted 2-isoxazolines possessing substituted oxopyrimidine motifs in position 5 can also be obtained using 1,3-DP cycloaddition of in situ-generated ArCNO to *N*-allylpyrimidine derivatives, as presented in work by Kushnir, Mel’nichenko, and Vovk—Scheme 78 [84].

**Figure 5:**
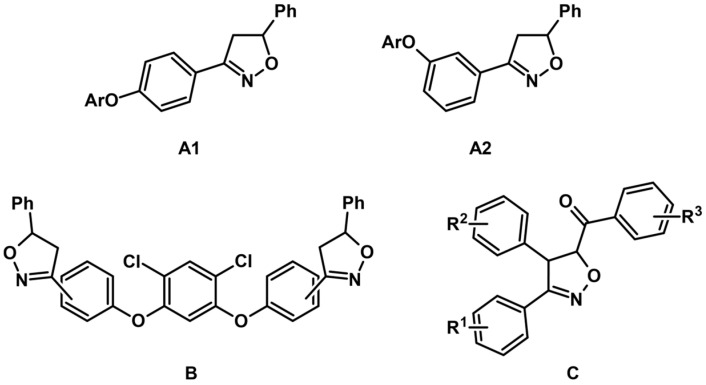
Structures of synthesized 3,5-di-substituted 2-isoxazolines and 3,4,5-trisubstituted isoxazoles [85]. Ar in (**A1**) and (**A2**) = 3,5-Cl_2_Py, 2,5,6-Cl_3_-2-Py, 2,3,5,6-Cl_4_-2-Py; (**B**) (1,4 or 1,3 substitution in benzene ring); R^1^, R^2^, and R^3^ in structure (**C**) (isoxazoles) = O_2_N, Cl, Me; Cl, Cl, MeO; F, H, Cl; and others.

Isoxazolines equipped with di-, tri-, or tetrachloropyridyl motifs were obtained typically, namely via 1,3-DP cycloaddition of nitrile oxide generated from the appropriate pyridinyloxy benzaldoximes to styrene or α,β-unsaturated ketones—Figure 5 [85]. Moreover, 3,4,5-trisubstituted isoxazoles were also synthesized via one-pot 1,3-DP cycloaddition, followed by oxidative isoxazoline dehydrogenation, as described in an article from Shailaja, Manjula, and Rao.

All the obtained isoxazolines and isoxazoles were evaluated for their antimicrobial and antifungal activity and action on isolated frog heart. It was suggested that isoxazolines of the A1 and A2 types reduced the heart rate, cardiac output, and force of contraction, but not as significantly as the referenced agent, i.e., diltiazem. Interestingly, biological tests have shown that isoxazolines were more active than isoxazoles.

The third report from Brel presented 3,5-disubstituted 2-isoxazolines equipped with the -(CH_2_)_n_CH=CHSF_5_ moiety. Those were synthesized typically via 1,3-DP cycloaddition of in situ-generated RCNO to dipolarophile bearing terminal SF_5_ group—Scheme 79 [86].

The SF_5_ moiety in molecular systems influences their physical, chemical, and biological behavior. Compounds containing this group often possess advantageous properties due to the electronegativity, high hydrolytic stability, and steric effect of the SF_5_ substituent. These properties are manifested in various potential applications, such as solvents for polymers, energetic materials, liquid crystals, rocket fuels, and others.

In their article from 2011, Viela et al. presented several 3,5-disubstituted isoxazoles obtained by isoxazoline formation via typical 1,3-DP cycloaddition, followed by oxidative dehydrogenation—Scheme 80 [87].

The same year, Castellano and her team described 1,3-DP cycloaddition of in situ-generated RCNO to allyl alcohol or *N*-Boc-protected 2,5-dihydropyrrole. It was a starting point for a synthetic method to produce isoxazoline-constrained analogues of procaine—Scheme 81 [88].

The activity against DNA methyltransferase 1 (DNMT1) for all obtained isoxazolines was tested. One of them, namely 3-(4-O_2_NPh)-5-(CH_2_NMe_2_)-2-isoxazoline, turned out to be more potent in vitro than other non-nucleoside inhibitors and exhibits a strong antiproliferative effect against HCT116 human colon carcinoma cells.

In particular, a designed dipolarophile, namely protected ß-1-C-allyl-1,4-dideoxy-1,4-imino-L-arabinitol, and *p*-MeOCH_2_C_6_H_4_CNO as a dipole were employed in the synthetic procedure leading to a special 3,5-disubstituted isoxazoline. As described by Chronowska and teammates, successive steps provided an indolizidine, an analogue of the natural (-)-steviamine—Scheme 82 [89].

In situ-generated benzonitrile oxide derivatives, the CH_2_=CHQ type of dipolarophiles, and 1,3-DP cycloaddition allowed Alam’s team to synthesize other 3,5-disubstituted 2-isoxazolines—Scheme 83 [90]. Selected isoxazolines were subjected to further modifications.

MIF-inhibitory activity evaluation revealed that one of the tested isoxazolines (R^1^ = HO, R^2^ = MeO, Q = CH_2_O(*p*-HOCC_6_H_4_) is non-toxic and inhibits tautomerase activity of huMIF and attenuates huMIF-mediated translocation of NF-jB to the nucleus. Moreover, this derivative prevents huMIF-mediated upregulation of iNOS and then NO production, which are well-established factors for inflammation. Due to the properties presented above, this isoxazoline might be beneficial for treating huMIF-induced inflammatory diseases.

An interesting report came from Tavares et al. in 2011. They used double 1,3-DP cycloaddition for the syntheses of liquid crystals, namely 3,7a-bis(4-alkyloxyphenyl)-7,7a-dihydro-6*H*-isoxazolo [2,3-*d*][1,2,4]oxadiazol-6-yl)acetic acid derivatives—Scheme 84 [91]. In the first step of this synthetic route, the 1,3-DP cycloaddition of 4-alkyloxyphenylnitrile oxide to vinylacetic acid gave the initial but unobserved 3,5-disubstituted 2-isoxazolines (1:1 cycloadducts), namely 2-[3-(4-alkyloxyphenyl)-4,5-dihydroisoxazol-5-yl]acetic acid derivatives.

The first reported work in 2012 concerned 2-isoxazoline-linked pseudodisaccharide analogues obtained by regioselective 1,3-DP cycloaddition between α-allyl-*C*-glycosides and sugar-derived nitrile oxides—Scheme 85 [92].

Biological activity evaluation of deprotected derivatives (using Pd(OH)_2_/C catalytic hydrogenation) showed that some of the tested saccharide–isoxazoline–saccharide hybrids behave as active species against HIV.

Several nitrofuran-isoxazoline-phenyl(pyridyl)-(*N*,*O*- or *N*,*S*-heterocycles) hybrids as potential antituberculosis agents were designed and synthesized by Rakesh and coworkers using 1,3-DP cycloaddition and other transformations—Scheme 86 [93].

The best three compounds selected for in vivo pharmacokinetic testing (A, R = N, **3**; CH, **3**; CH, **1**—see Scheme 86) all showed high oral bioavailability, with one notable compound showing a significantly longer half-life and good tolerability.

The same year, Kikuchi, Yoshida, and Shishido provided an example of fully regioselective 1,3-DP cycloaddition of in situ-generated MeCNO to the appropriate dipolarophile in the multistep synthetic total synthesis of (±)-3-hydroxy-ß-ionone—Scheme 87 [94].

Dadiboyena and Nefzi described in 2012 that resin-bounded dipolarophiles were used in the synthetic route leading to the 3,5-disubstituted 2-isoxazolines—Scheme 88 [95]. Despite good and very good efficiencies and regioselectivity, this procedure is hazardous and inconvenient due to the use of anhydrous HF in the final step, namely for cutting from resin.

The last report of 2012 came from Cheng and colleagues, who synthesized isoxazolines as an initial step towards *N*-substituted pyrrole-2-carboxylates, which played the role of crucial intermediates in the total syntheses of natural alkaloids, namely (−)-hanishin, (−)-longmide B, and (−)-longmide B methyl ester—Scheme 89 [96].

In 2013, 1,3-DP cycloaddition of in situ-generated substituted benzonitrile oxide to *N*-vinylpyrrolidyn-2-one or 4-vinylpyridine enabled Gong’s group to obtain several isoxazoline–pyrrolidine or isoxazoline–pyridine hybrids—Scheme 90 [97].

Using ArCNO (Ar = substituted phenyl) with a high electron density on the phenyl ring resulted in higher cycloadduct yields.

Later that year, Liu and coworkers described chiral, biologically interesting isoxazolines obtained in the high regio- and stereoselective reactions of in situ-generated RCNO with axially chiral *o*-*tert*-butylanilide—Scheme 91 [98].

1,3-DP cycloaddition was one of the important steps in the complex and multistep synthesis of spirastrellolide methyl ester—Scheme 92 [99]. Importantly, the key to achieving high selectivity was to use a chiral dipolarophile obtained from the chiral oxirane derivative.

Pyrazole-5-carboxamides containing imine, oxime ether, oxime ester, and dihydroisoxazoline motifs were designed and synthesized using 4-chloro-3-ethyl-*N*-(4-formylbenzyl)-1-methyl-1*H*-pyrazole-5-carboxamide as the key intermediate, as reported by Song et al. The derivatives equipped with the isoxazoline motif were typically obtained via 1,3-DP cycloaddition of specially designed (and in situ-generated) *p*-XC_6_H_4_CNO to 3,3-dimethylbut-1-ene—Scheme 93 [100].

Results of insecticidal evaluation revealed that the compound containing a dihydroisoxazoline moiety gave 60% inhibition at 50 mg/kg against spider mite adults and exhibited suitable activities against mosquitoes (60% at 1 mg/kg), which was near that of tebufenpyrad (70% at 1 mg/kg).

In 2013, Wang’s research group published an article regarding an interesting case. A stable polymer nitrile *N*-oxide (obtained in the reaction between 1,1-diphenylnitroethene with a living anionic polymer) was applied as an effective grafting tool for 1,3-DP cycloaddition to polymers equipped with an alloxy motif—Scheme 94 [101]. It is worth mentioning that the click-grafting procedure requires no catalyst or solvent.

Moreover, remarkable acceleration of the click grafting in the solid state was observed in comparison with 1,3-DP cycloaddition in solution.

As reported by Patel and coworkers, a series of 3,5-disubstituted 2-isoxazolines as potential positive allosteric modulators (“potentiators”) of α-amino-3-hydroxy-5-methyl-4-isoxazolepropionic acid (AMPA) receptors (AMPAR) were created starting from 1,3-DP cycloaddition of benzonitrile oxide derivatives to *N*-allyl iso-propylsulfonamide—Scheme 95 [102].

1,3-DP cycloaddition and 3,5-disubstituted isoxazoline formation were significant steps in the facile protocol for cross-linking unsaturated-bond-containing common polymers via the formation of masked-ketene-functionalized polymers—Scheme 96 [103].

In 2014, Han and colleagues published an article regarding obtaining other 3,5-disubstituted isoxazolines in a typical way, namely via regioselective 1,3-DP cycloaddition of RCNO to RCH=CH_2_—Scheme 97 [104]. RCNO were generated in situ from appropriate oximes using an iodobenzene-*m*-CPBA system generating hypervalent iodine intermediate as a true oxidation agent.

Another instance of dipolar cycloaddition leading to 3,5-di- and 3,5,5-trisubstituted isoxazolines was described in the literature by Rodrigues et al. Such a synthetic step led to the series of hydroxamic acid derivatives—Scheme 98 [105].

The obtained hydroxamic acids were tested as agents for treating Chagas disease. One of the evaluated compounds (X = NHOH, R^1^ = 4-benzyloxy, R^2^ = H) turned out to be very active and promising. Namely, preliminary in vivo data showed that this derivative reduces bloodstream parasites and that all treated mice survived; it was also more effective than the standard drug—benznidazole.

The same year Yu’s team synthesized a 2-isoxazoline motif containing a *N,N*-bidentate ligand equipped with a ferrocene backbone via 1,3-DP cycloaddition between 1,3-beneznedicarbonitrile dioxide and vinylferrocene—Scheme 99 [106].

Notably, the ligand mentioned above turned out to be very thermally stable and is especially effective in the palladium-catalyzed Heck coupling reaction.

Xiang, Li, and Yan published a paper about synthesizing 3,5-disubstituted 2-isoxazolines with a catalytic amount of in situ-generated hypervalent iodine (via reaction of PhI with *m*-ClC_6_H_4_COOOH) for the transformation of appropriate aldoximes into *p*-XC_6_H_4_CNO—Scheme 100 [107]. It is worth mentioning that applying the procedure presented above for obtaining 3,4,5- and 3,5,5-trisubstituted isoxazolines is also possible; namely, five examples of such derivatives were prepared likewise.

In 2015, Roßbach, Harms, and Koert reported applying a stable difluoroalkoxyborane intermediate as a dipolarophile in superior stereoselective 1,3-DP cycloaddition leading to 3,5-disubstituted 2-isoxazolines. Importantly, difluoroboranyl complexation of the α-hydroxy amide fragment of the dipolarophile molecule was essential for reaching high stereoselectivity in 1,3-DP cycloaddition of PhCNO to the F_2_B-complex of the vinyl-hydroxy-amide type dipolarophile—Scheme 101 [108].

The same year, a series of liquid-crystalline 3,5-diarylisoxazoles were obtained using typical methods, i.e., involving 1,3-DP cycloaddition leading to isoxazolines followed by oxidative dehydrogenation of precursors by MnO_2_—Scheme 102 [109].

Regioselective 1,3-DP cycloaddition of ArCNO to *N*-allyl- or *N*,*N*-diallylamines possessing–CH[PO(OEt)_2_]_2_ group leading to aminomethylenebis(phosphonates) with one or two isoxazoline motifs was a subject of two works from 2015 and 2017—Scheme 103 (an example with two isoxazoline motifs is presented) [110,111].

In an international patent from 2015, Fu and coworkers described peculiar 3,5-disubstituted isoxazolines, including chiral derivatives. A crucial step from this synthetic route, namely 1,3-DP cycloaddition of in situ-generated RCNO, is presented below—Scheme 104 [112]. These isoxazolines were designed and synthesized to work against infections caused by Gram-negative bacteria.

As reported by Filali et al., a series of harmine–isoxazoline hybrids were prepared by 1,3-DP cycloaddition of various arylnitrile oxides to *N*-allyl harmine—Scheme 105 [113].

All obtained harmine derivatives were evaluated in vitro against acetylcholinesterase and 5-lipoxygenase enzymes and MCF7 and HCT116 cancer cell lines. The most significant activity against acetylcholinesterase (IC 50 ¼ 10.4 mM) was obtained for unmodified harmine and cytotoxic activities (IC 50 ¼ 0.2 mM) for a hybrid when R = Ph. Two derivatives, namely equipped with R = thiophen-2-yl and furan-2-yl, respectively, showed good activity against the 5-lipoxygenase enzyme (IC 50 ¼ 29.2 and 55.5 mM, respectively).

Another reaction worth mentioning is the synthesis of chiral 3,5-disubstituted 2-isoxazolines involving 1,3-DP cycloaddition of PhCNO to enantiopure allyl alcohol—Scheme 106 [114].

The dipolarophile, namely chiral 1-(1,3-dithian-2-yl)prop-2-en-1-ol, was obtained by enzyme-catalyzed kinetic resolution. Significantly, the obtained isoxazoline can be smoothly transformed into chiral aminodiols, as authors reported in their work from 2015.

Kumar and coworkers also reported in 2015 the synthesis of 3,5-disubstituted isoxazolines equipped with the *N*-methylenedibenzazepine motif in position 5 via regioselective cycloaddition of in situ-generated RNCO from oxymoyl chlorides to *N*-allyldibenzodiazepine—Scheme 107 [115].

When the substituent R was *p*-BrC_6_H_4_, further functionalization was implemented. Namely, via Suzuki–Miyaura coupling, different aryl substituents were introduced into the final structures instead of the bromine atom. Importantly, several obtained isoxazolines possess excellent antimalarial activity with minimal or no cytotoxicity. Thus, overall results suggest that the dibenzoazepine-tethered 3,5-disubstituted isoxazolines are promising candidates for developing antimalarial drugs.

Koyama and colleagues described in 2015 obtaining ^13^C-labelled 3,5-disubstituted 2-isoxazoline from ^13^C-labelled nitrile oxide—Scheme 108 [116].

In 2015, very interesting *Pd*-catalyzed one-pot syntheses of 3,5-disubstituted 2-isoxazolines from arylmethanes as nitrile oxide sources and alkenes were performed—Scheme 109 [117].

It is worth emphasizing the unique and unprecedented triple role of silver nitrate in this cascade as a Pd(0) to Pd(II) oxidant, nitrogen source, and finally, a participant in nitrile oxide formation via dehydration. The Pd(II)–Pd(0) system is engaged in a methyl group C–H activation step leading to the formation of Ar-*Pd*-OTos intermediates. The protocol depicted above was also efficient for synthesizing 3,5,5-trisubstituted 2-isoxazolines—two examples of such derivatives were obtained.

As found in one of the more recent reports, the regioselective 1,3-DP cycloaddition of benzonitrile oxide derivatives to *N*-allyl-3-trihalomethylpyrimidin-2-ones was involved in synthesizing 3,5-disubstituted isoxazolines equipped with the pyrimidinone motif in position 5 (attached via CH_2_ linker) and Y-Ph substituent in position 3—Scheme 110 [118].

Importantly, the antiproliferative activity of the obtained isoxazolines was tested in vitro against five human tumoral cell lines: MCF-7 breast cancer cell line, ERþ (estrogen receptor positive); HepG-2 (hepatoma); T-24 (bladder cancer); HCT-116 cell (colorectal carcinoma); and CACO-2. The evaluation results were promising; some compounds presented IC 50 values below 2 mM and moderate to high selectivity. Moreover, they were less toxic against normal cells, and one of the tested isoxazolines (R = H, X = Cl) was three times more selective than MXT standard anticancer drugs.

A year later, Gucma, Gołębiewski, and Michalczyk reported high regio- and stereoselectivity in the cycloaddition of ArCNO to dipolarophiles bearing multiple double bonds—Scheme 111 [119]. It was unveiled that the addition to the exocyclic and terminal carbon–carbon double bond was strongly favored.

Coumarin motifs containing triazole, isoxazoles, isoxazolines, and aziridines were synthesized by Zayane’s team using 1,3-DP cycloaddition reactions. Synthesis of isoxazolines via 1,3-DP cycloaddition of in situ-generated RCNO to allyloxycumarin is presented below—Scheme 112 [120].

All obtained heterocyclic hybrids were evaluated for their antimicrobial, anticoagulant, and anticholinesterase activities. Interestingly, some of the tested hybrids, especially isoxazolines, displayed better antifungal activities than the parent 4-methylumbelliferone.

In 2016, Kulyashova and Krasavin described in their article 3,5-disubstituted isoxazolines bearing acylamino substituent in position 5. Those molecules were obtained by applying 1,3-DP cycloaddition of in situ-generated nitrile oxide to *N*-vinyl amides—Scheme 113 [121].

DP cycloaddition of RCNO into styrene derivatives was a crucial step in the synthetic route leading to 3,5-disubstituted 2-isoxazolines bearing a Schiff-base-derived motif, as Fritsch and Merlo reported—Scheme 114 [122].

An analogical series of isoxazole-containing derivatives was also obtained via oxidative dehydrogenation (aromatization) of isoxazoline precursors. Both isoxazolines and their aromatic analogues, i.e., isoxazoles, displayed liquid-crystalline behavior, namely they showed N, SmA, and SmC mesophases. Interestingly, isoxazoline-containing materials were more thermally stable than isoxazole-bearing derivatives. However, isoxazoles containing Schiff base liquid-crystalline materials displayed an extensive mesophase range and high clearing temperature.

Another case of 1,3-DP cycloaddition of selected RCNO to 3-trifluoromethyl-5-vinylisoxazole was used in the synthetic route leading to isoxazole–isoxazoline hybrids, this time by Poh et al.—Scheme 115 [123].

Ismail and his team published an article about the synthesis of isoxazoline-functionalized coumarins in which they mentioned 1,3-DP cycloaddition of ArCNO to *O*-allyl coumarin leading to coumarin–isoxazoline hybrids—Scheme 116 [124].

The obtained coumarin–isoxazoline hybrids were tested for their immune potentiating activity and exhibited appreciable activity, especially when Ar = *p*-MeOC_6_H_4_, *o*-MeOC_6_H_4_, and 2,4-(MeO)_2_PhC_6_H_3_.

Isoxazolines described the same year by Brullo and coworkers were typically obtained via 1,3-DP cycloaddition of in situ-generated RCNO to enamide—Scheme 117 [125] Importantly, nitrile oxide and the dipolarophile were explicitly designed for the intended application.

All obtained isoxazolines and structurally analogical 1,2-diazoles were tested regarding their activities to restore memory impairment. The most promising was a derivative with a 1,2-diazole motif.

In another report from 2016, Tsyganov et al. described synthesizing 3,5-disubstituted isoxazoline scaffolds as an initial step in a synthetic method leading to analogues of the bioactive natural alkoxynaphthalene pycnanthulignene D (shown in the frame)—Scheme 118 [126].

One of the obtained 1-arylalkoxynaphthalenes exhibited antiproliferative activity in a phenotypic sea urchin embryo assay.

As presented by Tamborini and colleagues, DP cycloaddition of ArCNO to 2-allylglutamic acid was a crucial step in the syntheses of amino acids belonging to glutamic acid homologues—Scheme 119 [127].

Interestingly, increasing the distance between the acidic amino moiety and the distal carboxylate group via inserting a phenyl-isoxazoline motif as a spacer produced a low-micromolar-affinity NMDA ligand that might represent a lead for the development of a new class of NMDA antagonists.

Other examples of isoxazolines that belong to the discussed class equipped with a boronic ester moiety were synthesized via 1,3-DP cycloaddition between PhCNO and mono- or 1,1-disubstituted alkenyl boronic esters and described by Jeong, Zong, and Choe—Scheme 120 [128].

Importantly, 1,3-DP cycloaddition was fully regioselective, and the boronic ester functionality plays a crucial role in controlling the regiochemistry of the reaction. Namely, only 2-isoxazolines, bearing the boronic ester group on the 5-position of the ring, were created. Furthermore, the cycloaddition reactions of PhCNO to the *trans*-1,2-disubstituted alkenyl boronic esters yielded 2-isoxazolines bearing the boronic ester group at the 4-position of the ring. The DFT calculations revealed that the parameters based on the TS and reaction energies were beneficial for predicting regioselectivity in the gas or solvent phase.

Efremova and her team obtained isoxazolines possessing an indole moiety in position 5 via 1,3-DP cycloaddition of RCNO (generated from RC(Cl)=NOH) to *N*-vinylindole—Scheme 121 [129].

The same year, Jakubiec and coworkers described the synthesis of 3,5-disubstituted 2-isoxazolines equipped with uracyl motif in position 3. They performed regioselective 1,3-DP cycloaddition of uracyl-motif-containing nitrile oxide to dipolarophiles of the RCH=CH_2_ type—see Scheme 122 below [130].

As reported by Choe et al., the regioselective intramolecular nitrile oxide 1,3-dipolar cycloaddition strategy (INOC) was involved in the syntheses of 3,5- and 3,4-disubstituted 2-isoxazolines—Scheme 123 [131].

The nitrile oxide motif was typically generated in situ from a CH_2_NO_2_ motif using *p*-ClC_6_H_4_CNO as a dehydrating agent. The obtained isoxazolines were precursors for the total syntheses of two Hsp90 inhibitors with potent anticancer activity, namely (+)-monocillin II and (+)-pochonin D.

Another article from this year presented two diaminopimelic acid analogous (DAP) diastereomers bearing an isoxazoline moiety synthesized via 1,3-DP cycloaddition—Scheme 124 [132].

After deprotection, the obtained diastereoisomers were evaluated for their inhibition against meso-diaminopimelate dehydrogenase (*m*-Ddh) coming from the periodontal pathogen, Porphyromonas gingivalis. Interestingly, only stereoisomer *S* (*S*-configuration at the C-5 position of the isoxazoline ring) displays significant inhibitory activity against *m*-Ddh under physiological conditions.

Typically obtained isoxazolines via 1,3-DP cycloaddition of ArCNO to 2-nitrostyrene were precursors in the synthetic route leading to the 2-arylquinolines—Scheme 125 [133].

After seven years, the modification of another rotaxane motif in this way was reported. 3,5-disubstituted 2-isoxazolines possessing a catenane motif were presented in the Japanese patent from 2017. These isoxazolines were typically obtained via cycloaddition of stable RCNO to allyltrimethylsilane—Scheme 126 [134].

The highly enantioselective cinchona-alkaloid-based amine-urea-catalyzed 1,3-DC cycloaddition of RCNO to *o*-hydroxystyrenes was performed by Suga and coworkers to obtain chiral isoxazolines with high *ee*—Scheme 127 [135].

DFT calculations strongly support that dual activation, namely the HOMO activation of *o*-hydroxystyrenes and LUMO activation of RCNO, take place in the crucial step in these reactions. Double activation due to hydrogen-bonding interactions between the Brønsted acid/base bifunctional catalyst is essential for obtaining high enantiomeric excess. The hydroxy group in the position *ortho* is also crucial for reaching high *ee*. Namely, changing the position of this substituent from *ortho* to *meta* or *para* or changing *o*-OH to *o*-MeO results in a dramatic decrease or even disappearance of enantioselectivity.

In 2017, Picconi’s team published an article about 1,3-DP cycloaddition providing access to 3,5-disubstituted 2-isoxazolines equipped with nitrofuranyl and pyridyl motifs—Scheme 128 [136].

All obtained isoxazolines were evaluated for their antibacterial activity against multiple-drug-resistant (MDR) *Staphylococcus* strains. Interestingly, compounds with a piperazine linker between the pyridyl group and the isoxazoline ring showed better activity when compared to compounds without the piperazine linker. Moreover, 3-pyridyl nitrofuranyl isoxazoline was more active than corresponding 2-and 4-pyridyl analogues against MDR *Staphylococcus* strains. Importantly, they were found to be non-toxic against non-tumor lung fibroblast WI-38 and cervical cancer cell line HeLa.

In 2018, Korgavkar and Samant described the preparation of cross-linked basic, mesoporous polymers by the polymerization of 4-vinylpyridine and 1-(4-vinylbenzyl)imidazole using DVB as a cross-linker used as heterogeneous, reusable catalysts for the in situ generation of ArCNO from ArC(Cl)NOH. Finally, the ArCNO underwent 1,3-DP cycloaddition to *N*-phenylmaleimide and ethyl acrylate (present in the reaction mixtures)—Scheme 129 below [137].

The polymeric catalyst prepared from imidazole derivative was more active due to higher basicity of imidazole than pyridine, and it is characterized by much higher surface area and porosity than the pyridine analogue.

As Mirosław with her team stated in their article from 2018, 5-(nitromethyl)-3-phenyl-isoxazoline can be obtained as the only product of a high-yielding 1,3-DP cycloaddition of in situ-generated PhCNO to 3-nitroprop-1-ene—Scheme 130 [138].

The quantum-chemical calculation performed at the M06-2X/6-31G(d) (PCM) theoretical level explained the reaction course and the nature of transition states. Importantly, DFT calculations suggested relatively higher stability of the 5-nitromethyl isomer compared to the 4-nitromethyl one.

Another report from Yang and coworkers presented 1,3-DP cycloaddition involved in the syntheses of 3,5-disubstituted 2-isoxazolines equipped with podophyllotoxin or 2′(2′,6′)-(di)halogenopodophyllotoxin moieties—Scheme 131 [139].

Similar to the previous work, all obtained podophyllotoxin–isoxazoline hybrids were evaluated for their pesticidal activities against *Mythimna separata* and *Tetranychus cinnabarinus*. Interestingly, the halogen atoms at the C-2′/C-2′,6′ position of the podophyllotoxin core and the chlorine atom at the C-4 position on the phenyl of the isoxazoline motif were crucial as far as insecticidal and acaricidal activities of tested compounds are concerned.

1,3-DP cycloaddition of in sit- generated RCNO to the styrene derivative performed in the presence of bifunctional chiral amine(cinchonine-based)-urea catalyst resulted in chiral isoxazolines, as Toda et al. described—Scheme 132 [140].

Dipolar cycloaddition of nitrile oxide to *N*-vinylcaprolactam was reported the same year by Sağirli and Dürüst, who utilized it to prepare 3,5-disubstituted 2-isoxazolines bearing a *N*-caprolactam moiety in the fifth position—Scheme 133 [141].

In 2018, Low and coworkers described obtaining 3,5-disubstituted 2-isoxazolines via 1,3-DP cycloaddition of in situ-generated RCNO to allyl chloride—Scheme 134 [142].

The regioselective cycloaddition presented above was an initial step in the multistep synthetic method leading to several fused cyclopropyl-3-amino-2,4-oxazine (BACE1 inhibitors).

Lee’s team identified the novel potential LpxC inhibitors using computational methods that leveraged numerous crystal structures. Such an approach allowed for the selection of isoxazoline (and oxazolidinone), which could be inhibitors of in vitro activity against *P. aeruginosa* and other Gram-negative bacteria. Several promising structures were obtained via 1,3-DP cycloaddition followed by the next transformation—Scheme 135 [143].

Some of the obtained compounds were tested regarding their antibacterial activity against *K. pneumoniae* in in vitro and in vivo experiments.

The next 2018 report from Adardour et al. presented 3,5-disubstituted 2-isoxazolines obtained via regioselective 1,3-dipolar cycloaddition of nitrile oxide to *N*-allyl benzimidazolone—Scheme 136 [144].

In 2019, a green protocol for synthesizing 3,5-disubstituted isoxazolines via 1,3-DP cycloaddition of nitrile oxide to alkenes was proposed by Zhao and colleagues. Namely, nitrile oxides were efficiently generated in situ from Oxone/NaCl oxidation of a broad scope of aliphatic, aromatic, and alkenyl aldoximes without the generation of organic byproducts—Scheme 137 [145].

The following advantages characterize the three-component procedure presented above involving aldehyde, hydroxylamine hydrochloride, and alkenes: simple open-flask operation, low-cost and non-toxic reagents, and air and moisture resistance. Moreover, from the mechanistic point of view, this three-component oxidative cycloaddition is expected to have an extensive impact on the application of 1,3-DP cycloaddition of nitrile oxide to the carbon–carbon double (and triple) bond.

Distante, Collina, and Quadrelli, in their work from 2020, proposed synthesizing isoxazoline that possesses N-Boc-protected (S)-alanine motif in position 5 and phenanthrene motif in position 3 via 1,3-DP cycloaddition between N-Boc-protected (S)-alanine allyl ester and phenanthrene nitrile oxide—Scheme 138 [146].

According to the authors, thanks to its optical properties, the obtained isoxazoline can be helpful in constructing chemical probes in living systems without interfering with natural biochemical processes.

A series of 3,5-disubstituted 2-isoxazolines containing difluorobenzamide motifs were designed, synthesized, and tested, as far as their in vitro and in vivo biological activities are concerned. As reported by Song et al., the isoxazolines mentioned above were obtained typically via dipolar cycloaddition of in situ-generated nitrile oxide to the appropriate alkene, i.e., 3-(2-propenyloxy)-2,6-difluoroethanamide—Scheme 139 [147].

Notably, the obtained compounds were pharmacologically evaluated as FtsZ-targeting antibacterial agents and structure–activity relations were also discussed.

**Figure 6:**
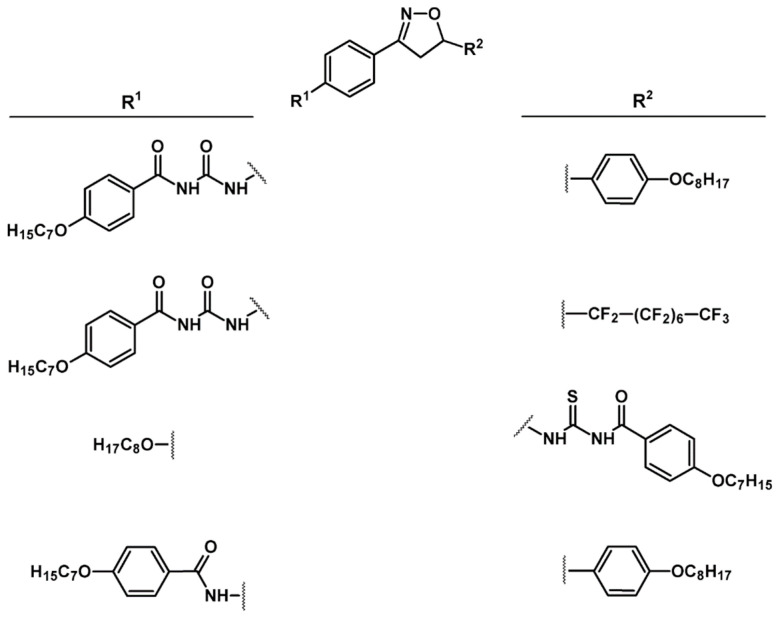
Syntheses of 3,5-disubstituted isoxazolines equipped with motifs generating liquid-crystalline properties [148].

As reported by Gonçalves and colleagues in 2020, typical procedures (1,3-DP cycloaddition and other transformations) were applied in the synthetic methods leading to the isoxazolines (and isoxazoles) equipped with specially designed motifs in positions 3 and 5—Figure 6 [148].

The SmA mesophase was predominant. However, the nematic mesophase was observed only for thioureas and amides equipped with the isoxazole moiety. Interestingly, the isoxazoline derivatives showed a less extensive mesophase range than the isoxazole derivatives.

In 2020, Plumet reported that 3,5-disubstituted 2-isoxazolines can also be prepared under supercritical CO_2_ (“non-conventional” conditions)—see Scheme 140 [149].

Generally, 1,3-dipolar cycloaddition under supercritical CO_2_ is poorly understood. Moreover, when it comes to isoxazolines synthesis, there are no reported benefits from using these demanding “non-conventional conditions”, namely ionic liquids or supercritical carbon dioxide as solvents.

The following report came from Umemoto, Imayoshi, and Tsubaki. They published a procedure involving nitrile oxides derived from *O*-alkyloxime-substituted nitroalkanes, various CH_2_=CHR type dipolarophiles, and 1,3-DP cycloaddition (regioselective) in the syntheses of 3,5-disubstituted 2-isoxazolines—Scheme 141 [150].

The synthetic strategy presented above turned out to be effective for syntheses of 3,4,5- and 3,5,5-trisubstituted 2-isoxazolines as well—see Section 2.5 and Section 2.6.

Saccharin–isoxazoline hybrids equipped with a saccharine motif in position 3 and a aryl/heteroaryl motif in position 5 were successfully obtained by D’Ascenzio and coworkers via 1,3-DP cycloaddition of selected nitrile oxide to *N*-allyl saccharin—Scheme 142 [151].

In vitro examination evidenced that the obtained hybrids were characterized by a strong affinity for hCA IX and XII and marked selectivity over hCA I and II. It is also worth noting that two of the most potent nanomolar hCA IX and XII inhibitors did not display any cytotoxic effect up to 200 μM on primary human fibroblasts despite the substitution pattern (nitro or methoxy moiety). Additionally, the hybrid equipped with Ar = *p*-MeOC_6_H_4_ was shown to act as a chemosensitizer and coadjuvant in combination with subtoxic doses of doxorubicin on the MCF7 breast cancer cell line.

Another team described 1,3-DP cycloaddition of in situ-generated ArCNO to methyl acrylate, resulting in 3,5-disubstituted 2-isoxazolines—Scheme 143 [152].

Importantly, all obtained derivatives were active in vitro against *M. exigua* second-stage juvenile. Moreover, isoxazolines possessing Ph or *m*-ClC_6_H_4_ in position 3 were shown to be the most promising for the development of new commercial nematicides.

Liu and coworkers described performing 1,3-DP cycloaddition of in situ-generated ArCNO to allylbenzene derivative. In a typical way, several sarisan derivatives equipped with 3,5-disubstituted 2-isoxazolines moieties were synthesized—Scheme 144 [153].

All obtained sarisan derivatives equipped with a 3,5-disubstituted 2-isoxazolines motif showed antifungal activities against selected phytopathogenic fungi (*B. cinerea*, *C. lagenarium*, *A. solani*, *F. solani*, and *F. graminearum*). Interestingly, some evaluated compounds displayed relatively low toxicity on normal NRK-52E cells. Moreover, the above-mentioned results will pave the way for the development of sarisan derivatives as fungicide candidates in plant protection.

Regioselective 1,3-DP cycloaddition of ArCNO to *N*-allylbenzothiazinone was utilized in the syntheses of 3,5-disubstituted isoxazolines bearing a benzothiazinone motif in position 5, as Sebbar and colleagues described in their article from 2020—Scheme 145 [154].

Antibacterial activity tests unveiled that some of the obtained hybrids are moderately active against some Gram-positive and Gram-negative microbial strains. The DFT calculations supported the experimental assessment of the molecules’ structure (also mentioned in Section 3).

The following year, Aarjane’s team reported synthesizing 3,5-disubstituted isoxazolines equipped with a *p*-chlorophenyl moiety in position 3 and a acridone–CH_2_ motif in position 5. It was obtained via 1,3-DP cycloaddition of in situ-generated *p*-ClC_6_H_4_CNO to *N*-allylacridone—Scheme 146 [155].

The synthesized isoxazoline was characterized not only experimentally but also theoretically—as commented on in Section 3.

In 2021, the formation of nitrile oxides with diazocarbonyl compounds by nitrosyl transfer from *tert*-butyl nitrite under mild conditions and without the use of a catalyst or an additive was reported by de Angelis et al. [156]. The authors used this transformation to synthesize isoxazolines (and other *N*,*O*-containing heterocycles) by cycloaddition—Scheme 147.

Interestingly, this methodology was also applied in the millimole-scale synthesis of two biologically active compounds.

The same year, Zhang and colleagues observed fully regio- and enantioselective 1,3-DP cycloaddition in a reaction between PhCNO and chiral dipolarophiles—Scheme 148 [157].

Obtaining cholesterol–isoxazoline hybrids via 1,3-DP cycloaddition of derivatives of benzonitrile oxide into cholesterol oxime allyl ether was described by Xu et al.—Scheme 149 [158].

All obtained cholesterol–isoxazoline hybrids (and cholesterol–isoxazole hybrids) were tested regarding their agricultural bioactive properties. Some tested compounds exhibited high activity against *Mythimna seperata* (Walker), *Plutella xylostella* (Linnaeus)*, Aphis citricola* (Van der Goot), and *A.citricola* in the greenhouse. Importantly, SARs analysis suggested that the C-3 hydroxyl group and the C-7 position of cholesterol are two essential modification sites. Undoubtedly, the results presented above pave the way for structural optimization and application of cholesterol–isoxazoline hybrids (and cholesterol–isoxazole hybrids) as potential pesticidal agents in agriculture.

Several 3,5-disubstituted 2-isoxazolines were obtained by Gaikwad and coworkers in the 1,3-DP cycloaddition involving substituted acrylamides as dipolarophiles and benzonitrile oxide derivatives as 1,3-dipoles—Scheme 150 [159].

Antitubercular evaluation of all synthesized compounds showed that when R^1^ = p-Cl and R^2^ = cyclohexyl, such an isoxazoline is highly active (MIC 1 μg/mL) against Mtb and drug-resistant strains. Moreover, all examined isoxazolines are non-toxic when tested against Vero cells. Notably, in in silico studies, the binding patterns of tested compounds to target mycobacterial membrane protein Large-3 exhibited excellent cooperation between isoxazolines and the receptor.

Derivatives of benzonitrile and 2-pyridylnitrile oxide were used for the syntheses of other isoxazolines described in 2021 by Endoori et al. Using 1,3-DP cycloaddition of the formerly mentioned ArCNO to an amide of formic acid, several molecules were synthesized—Scheme 151 [160]. In the next step, isoxazolines were finally modified via acylation of NHC(O) group by selected acyl chlorides.

All isoxazolines presented above were evaluated for in vitro anticancer activity against two human cancer cell lines, HeLa and MCF-7. The obtained results revealed that the selected test compounds (R^1^ = CF_3_, Cl; R^2^ = 3,5-dimethoxyphenyl, 5-methyl-3-yl-isoxazole, 6-methyl-2-pyrazine,) were found to be more effective inhibitors of the growth of two cell lines with IC 50 values that are close to those of standard drugs. Importantly, isoxazoline possessing X = N, R^1^ = CF_3_, and R^2^ = 5-methyl-3-yl-isoxazole was more potent than the standard drug cisplatin, with IC 50 values of 4.11 and 4.03 lM against the Hela cell line and MCF-7 cell line, respectively.

In another article from 2021, Pajkert and others presented 3,5-disubstituted isoxazolines obtained via 1,3-DP cycloaddition of in situ-generated (diethoxyphosphoryl)-difluoromethyl nitrile oxide to selected dipolarophiles of the CH_2_=CHR type—Scheme 152 [161].

Ghosh and Hsu reported in 2021 obtaining specially designed isoxazolines as a crucial step in a multi-step synthetic method leading to a potent anticancer agent—Scheme 153 [162]. A -CNO moiety was created in situ from a –C=NOH moiety via reaction with OXONE.

In 2022, a group of researchers published an article regarding the formation of 3,5-disubstituted isoxazolines via the discussed method. 1,3-DP cycloaddition was an initial step in the synthetic procedure leading to the 3-hydroxypyrrolidin-2-ones, including on a large scale (up to 200 g)—Scheme 154 [163].

Notably, the method presented above is operative not only for the final product, i.e., lactams, but also for the syntheses of isoxazolines, which can be separated before the last step.

The same year, 3,5-disubstituted isoxazolines bearing an RPh motif in position 3 and quinazoline–CH_2_ motif in position 5 were synthesized via regioselective 1,3-DP cycloaddition by Rhazi and colleagues—Scheme 155 [164].

More remarks regarding the theoretical predictions and analysis carried out by the authors are presented in Section 3.

In parallel reports, the procedure of obtaining triazole-eugenol-isoxazoline-*p*-chlorophenyl hybrids involving 1,3-DP cycloaddition of *p*-ClC_6_H_4_CNO to aryltriazole-allyleugenol-type dipolarophiles was described—Scheme 156 [165,166].

Antiproliferative activity tests disclosed that some of the obtained derivatives showed significant cytotoxicity against fibrosarcoma and lung and breast carcinoma cell lines [166]. Interestingly, one of the hybrid compounds (R = Bn) showed the highest anticancer activity against all tested tumor cell lines, with IC 50 values between 15.31 and 23.51 mM [165].

### 2.4. 5,5-Disubstituted 2-Isoxazolines

These types of isoxazolines, i.e., 5,5-disubstituted in position without a substituent in position 3, cannot be obtained via RCNO cycloaddition because R cannot be a hydrogen atom.

### 2.5. 3,4,5-Trisubstituted 2-Isoxazolines

The first report about applying 1,3-DP cycloaddition leading to 3,4,5-trisubstituted isoxazolines came from Batt and colleagues in 2000. They prepared the *trans*- and *cis*-isomers of a ring-hydroxylated metabolite of the GPIIb/IIIa antagonist XV459, the active drug form of Roxifiban—Scheme 157 [167].

3,4,5-trisubstituted isoxazolines were prepared typically and evaluated for their in vitro and in vivo antithrombotic efficacy by Pruitt and colleagues—Scheme 158 [168].

The following year, Faita et al. reported 1,3-DP cycloadditions of MesCNO to supported dipolarophile connected to chiral auxiliary in the presence (stoichiometric quantities) or in the absence of Mg^2+^. Such an attempt was used for the syntheses of chiral isoxazolines—Scheme 159 [169]. Importantly, the presence of acetonitrile as a co-solvent was fundamental for this reaction.

3,4,5-trisubstituted isoxazolines obtained via two synthetic pathways were precursors in the syntheses of isomeric isoxazoles, as described by Easton’s team in their work from 2001—Scheme 160 [170].

Finally, the obtained isoxazoles underwent electrochemical and yeast-catalyzed N–O bond cleavage leading to analogues of the herbicide Grasp.

Using chiral substrates, RCNO, and the dipolarophile, namely allyl alcohol, enantiomerically pure isoxazolines were obtained by Bode and coworkers—Scheme 161 [171]. Coupling of the appropriately substituted isoxazolines provided access to bis-isoxazoline and, finally, polyketide building blocks. It is worth mentioning that in this strategy, the isoxazoline motif plays the role of masked ß-hydroxy carbonyl compounds.

As presented in their article from 2001, Bosanac, Yang, and Wilcox synthesized 3,4,5-trisubstituted 2-isoxazolines possessing a Ph-substituted (*E*)-stilbene motif (attached through –CH_2_OC(O)– linker) in position 5 via 1,3-DP cycloaddition of appropriate nitrile oxide to methacrylate equipped with the above-mentioned stilbene fragment—Scheme 162 [16].

The next year, the obtention of chiral, 3,4,5-trisubstituted 2-isoxazolines (4*S*, 5*S*, 5′*S*) via *Mg*-mediated 1,3-DP cycloaddition of in situ-generated RCNO to chiral (*S*)-α-silylallyl alcohols was described by Kamimura et al.—Scheme 163 [172].

Importantly, all obtained isoxazolines were readily converted into [1,2]-oxazines without loss of optical purity.

As presented in Kai and coworkers’ article from 2002, DP cycloaddition between in situ-generated oxymoyl chloride and vinyl ethers was applied in the syntheses of 3,4,5-trisubstituted 2-isoxazolines bearing alkoxy, substituted benzyloxy, and alkyl groups—Scheme 164 [173].

Bicyclic acidic amino acids, which are conformationally constrained homologues of glutamic acid, were prepared by Conti et al. via a strategy based on a 1,3-DP cycloaddition—Scheme 165 [174].

**Scheme 166:**
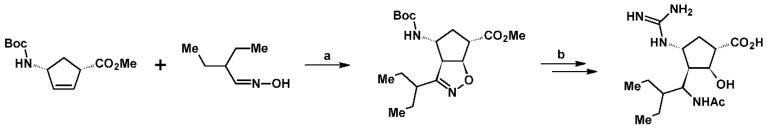
Specially designed 3,4,5-trisubstituted 2-isoxazoline as a crucial intermediate in the total synthesis of the neuraminidase inhibitors as anti-influenza agents [176]. a = NaOCl (bleach), Et_3_N, CH_2_Cl_2_, 61% yield; b = next steps.

**Scheme 1:**
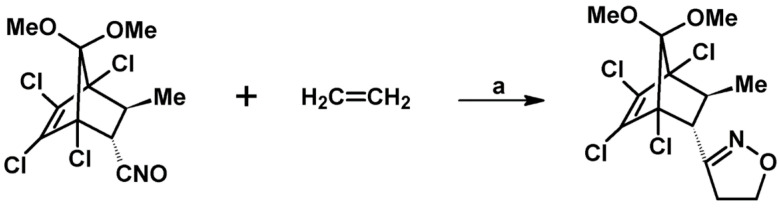
Synthesis of 3-substituted isoxazoline via reaction of ethene with RCNO [2]. a = conditions and the product yield were not given.

**Scheme 2:**
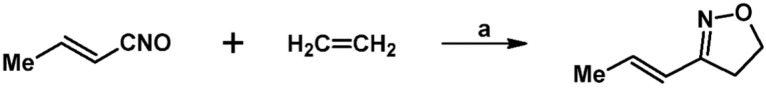
Synthesis of 3-substituted isoxazoline via reaction of ethene with RCNO [3]. a = up to rt; atmospheric pressure, CHCl_3_ (or other solvent); up to 48% yield.

**Scheme 3:**
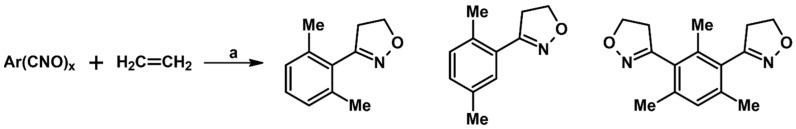
Syntheses of 3-substituted isoxazolines via cycloaddition of R(CNO)_1 or 2_ to ethylene [4]. a = rt, CHCl_3_; quantitative yield.

**Scheme 4:**
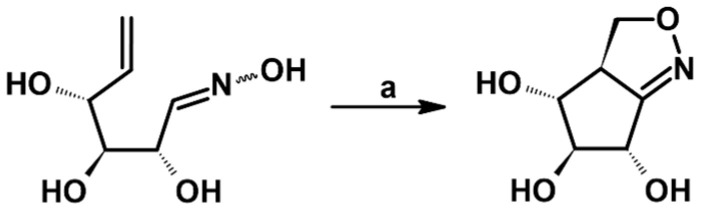
INOC strategy involved in the syntheses of isoxazolines: exemplary reaction and selected products [5]. a = oxime generation; next: chloramine–T, silica gel, EtOH; up to 94% yield.

**Scheme 5:**
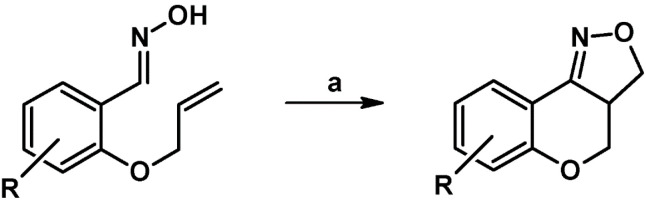
Intramolecular cycloaddition of nitrile group into double bond—syntheses of 3,4-disubstituted isoxazolines [6]. R = H; 3,5-(*t*-Bu)_2_; 3-MeO; 5-Cl; 5-Br; 5-NO_2_; and others; a = HTIB, water, rt, 50 min; up to 89% yield.

**Scheme 6:**
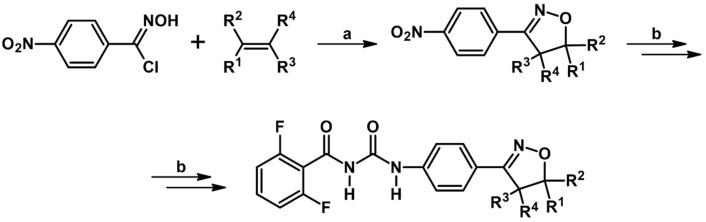
Syntheses of isoxazolines equipped in position 3 with a *N*-(3,5-difluorobenzoyl)-urea motif connected with isoxazoline ring through 1,4-diphenylene linker [8]. R^1^, R^2^, R^3^, R^4^ = *t*-Bu, H, H, H; C_6_H_4_F, H, H, H; *n*-Bu, H, H, H; CH_2_Br, H, H, H; CN, H, H, H; Me, Ph, H, H and others; a = Et_3_N, CH_2_Cl_2_; up to 91% yield; b = next steps; up to 88% yield.

**Scheme 7:**
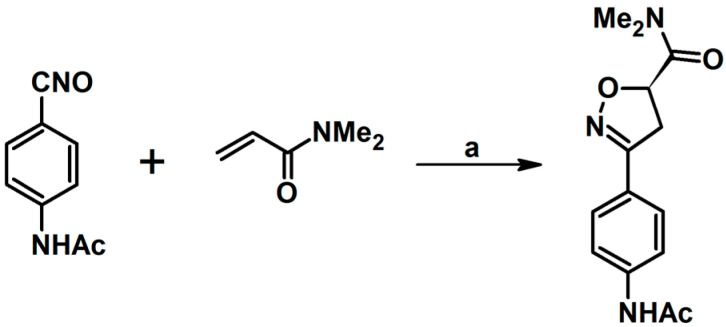
Synthesis of chiral 3,5-disubstituted isoxazolines via highly enantioselective 1,3-DP cycloaddition mediated by monoclonal antibody [9]. a = EtOH, rfx., 3 h; 38% yield.

**Scheme 8:**
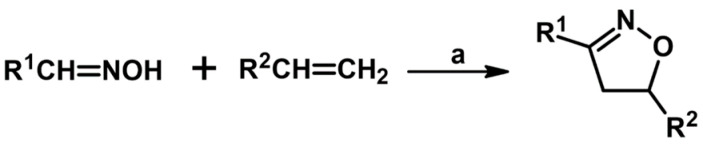
Syntheses of 3,5-disubstituted isoxazolines using [BMTA][ICl_4_] for obtaining oxymoyl chlorides, followed by RCNO, starting from oximes [10]. (1) R^1^ = *p*-MeC_6_H_4_, *p*-MeOC_6_H_4_, *p*-O_2_NC_6_H_4_, 2- or 3- or 4-Py, *t*-Bu and others; R^2^ = Ph; (2) R^1^ = *p*-MeC_6_H_4_; R^2^ = COOEt or CH_2_OH; a = [BMTA][ICl_4_], Et_3_N, CH_2_Cl_2_, rt; up to 95% yield.

**Scheme 9:**
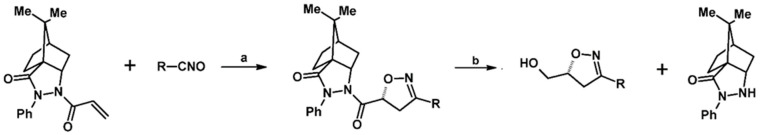
Syntheses of optically pure 3,5-disubstituted isoxazolines engaging chiral camphor-derived auxiliary [11]. R = Ph or *t*-Bu; a = NCS, Et_3_N; b = L-Selectride, THF, −78 °C; regioselectivity = 99/1; 97% yield (for Ph) and 96% yield (for *t*-Bu); *ee* = 99%; auxiliary recovery = up to 92%.

**Scheme 10:**
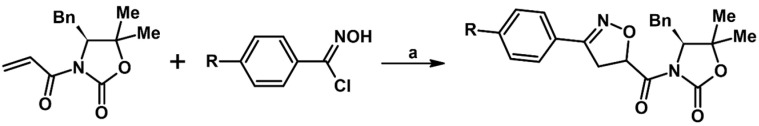
Syntheses of 3,5-disubstituted isoxazolines mediated by MgBr_2_ [12]. R = H, MeO, O_2_N, Cl, F; a = MgBr_2_, Et_3_N, CH_2_Cl_2_, 0 °C, 6 h; up to 92% yield; *de* = up to 96%.

**Scheme 11:**
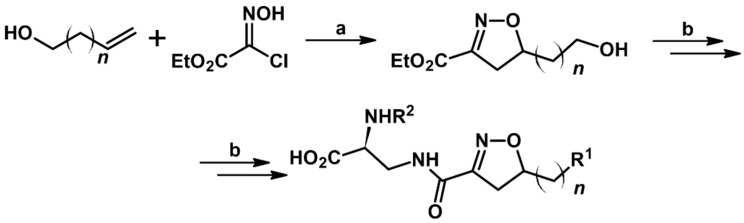
From simple 1,3-DP cycloaddition of RCNO generated from EtOOCC(Cl)=NOH to dipolarophile HO(CH_2_)_2_CH=CH_2_ type to the potent antagonist of the Integrin α_v_β_3_ [13]. R^1^, R^2^, *n* = H_2_NC=NH(NH), SO_2_Mes, 4; pyridin-2-ylNH, SO_2_Mes, 4; isoqinolin-1-ylNH, SO_2_Mes, 4; imidazol-2-ylNH, 2,4,6-(Me)_3_C_6_HSO_2_, 4; pyridin-2-ylNH, 1-naphthylSO_2_; and others (34 examples). a = NaHCO_3_, THF, 0 °C, rt; up to 86% yield; b = next steps.

**Scheme 12:**
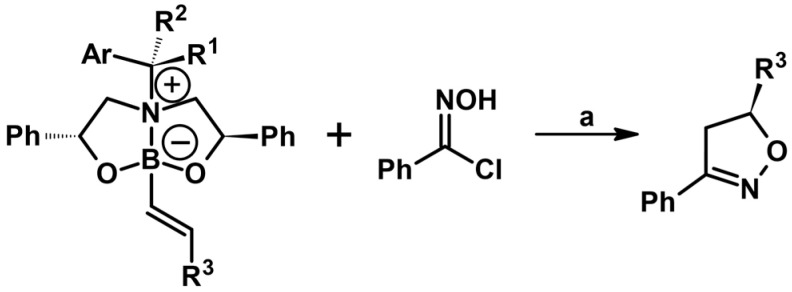
Syntheses of chiral 3,5-disubstituted isoxazolines using dioxazaborocine auxiliaries [14]. Ar, R^1^, R^2^, R^3^ = Ph, Me, H, CO_2_Me; 2-Naphthyl, Me, H, CO_2_Me; Ph, Me, H, Ph; and others; a = Et_3_N, THF, rt; up to 61% yield; *ee* = up to 70%.

**Scheme 13:**
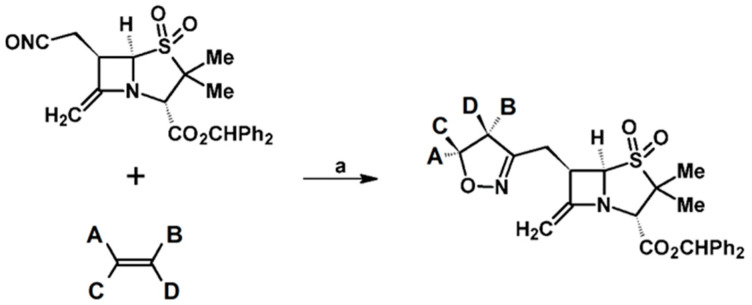
1,3-DP cycloaddition engaged in the syntheses of isoxazolines possessing sulbactam motif in position 3 [15]. A, B, C, D = COOMe, H, H, H; H, H, CO_2_Me, H; H, H, SO_2_C_6_H_4_; C_6_H_4_S, H, H, H and others. a = 0 °C or rt, CH_2_Cl_2_ or benzene, Et_3_N or TBTO, up to 2 h, up to 68% yield.

**Scheme 14:**
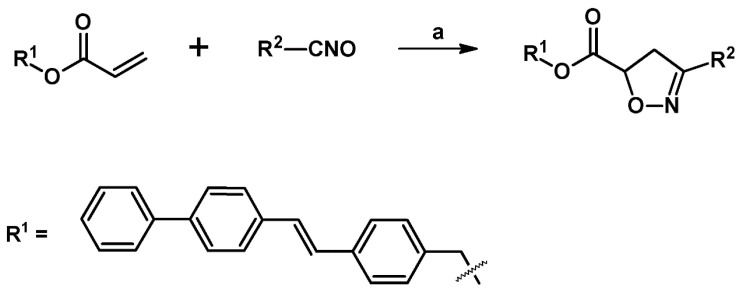
Syntheses of isoxazolines equipped with the (*E*)-stilbene motif [16]. R^2^ = *t*-Bu, Ph, *p*-O_2_NC_6_H_4_; a = Et_3_N, CH_2_Cl_2_, 0 °C; up to 90% yield.

**Scheme 15:**
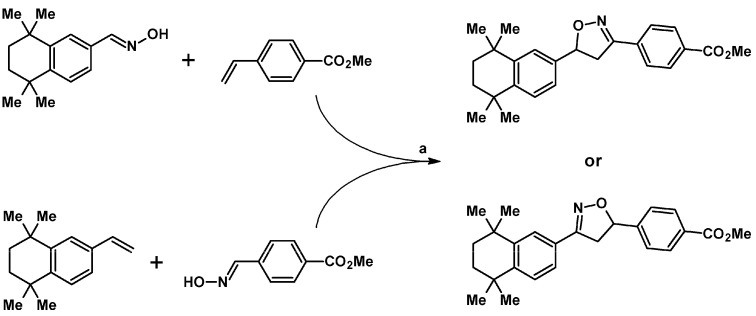
Preparation of isomeric, disubstituted isoxazolines via two synthetic routes [17]. a = CHCl_3_, Py, NCS, Et_3_N; y = 63% (**upper product**) and 60% (**bottom product**).

**Scheme 16:**
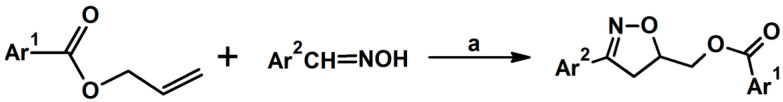
One-pot DP cycloaddition of benzonitrile oxide derivatives to various allyl esters [18]. Ar^1^, Ar^2^ = *p*-BrC_6_H_4_, Ph; *p*-BrC_6_H_4_, 3,4-(MeO)_2_C_6_H_3_; *p*-O_2_NC_6_H_4_, *p*-MeOC_6_H_4_; 5-BrFuryl, *p*-MeC_6_H_4_, and others (30 examples); a = NCS, CHCl_3_, rt; next Et_3_N, rt; up to 72% yield.

**Scheme 17:**
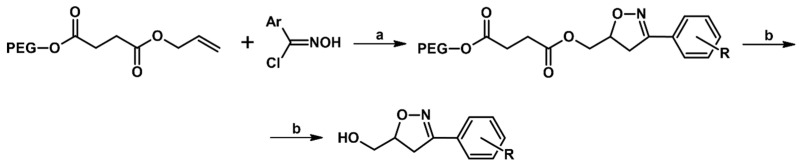
Syntheses of 5-hydroxymethyl-3-(R-Ph)-disubstituted 2-isoxazolines using supported dipolarophile [19]. PEG = poly(ethylene glycol); R = H, 4-MeO, 4-F, 4-Me, 3-O_2_N, 2-Cl; a = Et_3_N, CH_2_Cl_2_, rt; b = 2M NaOH, rt, 3 h; up to 96% yield.

**Scheme 18:**
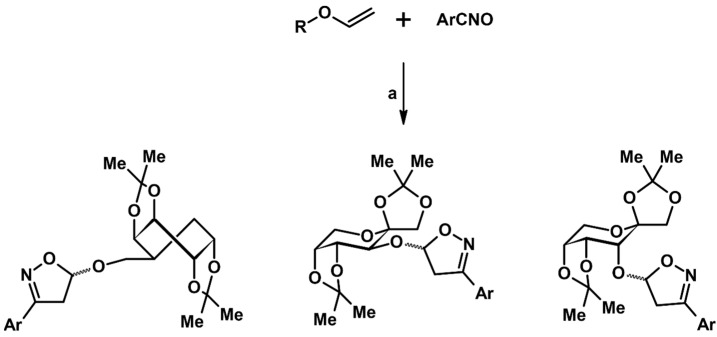
Asymmetric induction in the syntheses of 3,5-disubstituted isoxazolines: sugar motifs as chiral templates [21]. Ar = Ph, 2,4,6-trimethyl- and trimethoxyphenyl; a = pyridine + Et_3_N, Et_2_O + CHCl_3_, rfx, 2 h; up to 98% yield; epimeric ratio = from 1:1 to 28:1.

**Scheme 19:**
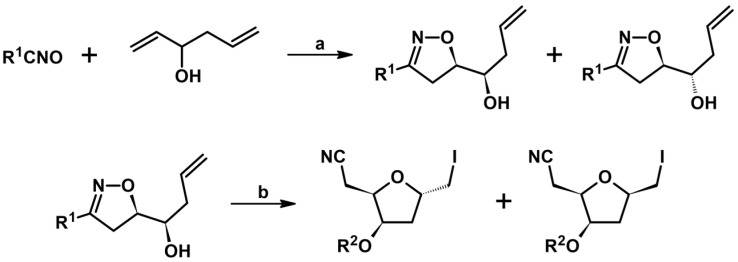
Syntheses of tetrahydrofuran derivatives starting from the syntheses of appropriate isoxazolines obtained in Mg-catalyzed 1,3-DP cycloaddition of RCNO to 1,5-hexadien-3-ol [22]. R^1^ = Ph_3_C, PhCH(OTBDMS); a = EtMgBr, CH_2_Cl_2_, or benzene, 0 °C; up to 81% yield (isoxazolines), syn/anti = for Ph_3_C 8/1 in benzene and 19/1 in CH_2_Cl_2_, for PhCH(OTBDMS) = 7/1 in benzene and 25/1 in CH_2_Cl_2_; b = protection, then ICl; R^2^ = TBDMS, TBDPS, MPM, MOM, MEM.

**Scheme 20:**
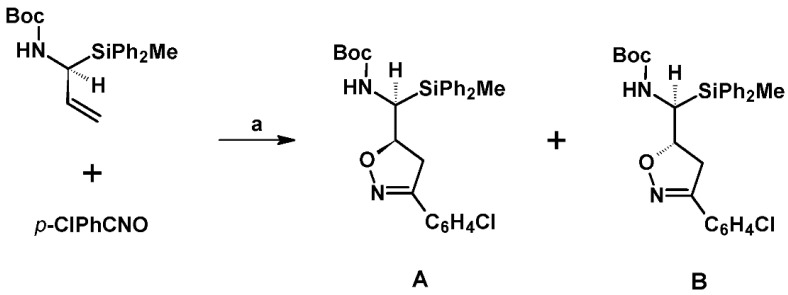
Synthesis of chiral, diastereomeric isoxazolines via cycloaddition of RCNO to chiral allyl silane [24]. a = Et_3_N, 22 h, rt; A/B = 2/1; 43% yield for (**A**) and 22% yield for (**B**).

**Scheme 21:**
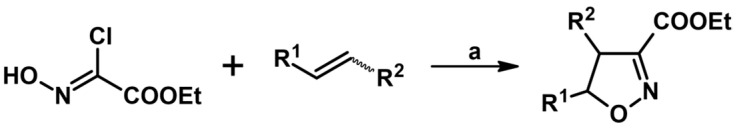
Syntheses of 3,5-di- and 3,4,5-trisubstituted isoxazolines in the ionic liquids [25]. R^1^, R^2^ = EtOOC, H; EtOOC, EtOOC; NC, H; Me_3_Si, H; ClCH_2_, H and others; a = [BMIM][BF_4_] or [BMIM][PF_6_], KHCO_3_, rt, up to 12 h; up to 95% yield.

**Scheme 22:**
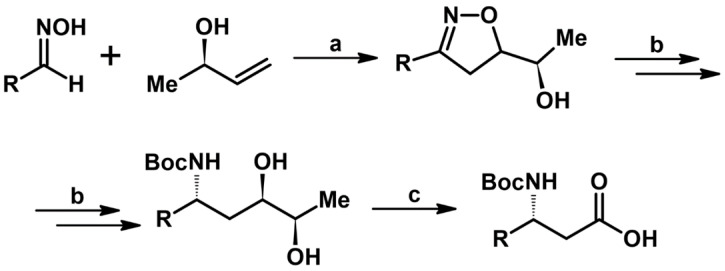
Syntheses of chiral isoxazolines as precursors of chiral diols and amino acids [26]. R = Et, *i*-Pr, TIPS–C≡C–, Ph; a = *t*-BuOCl, CH_2_Cl_2_; next EtMgBr, *i*-PrOH; up to 96% (isoxazolines) yield; b = next steps; c = NaIO_4_/RuCl_3_.

**Scheme 23:**
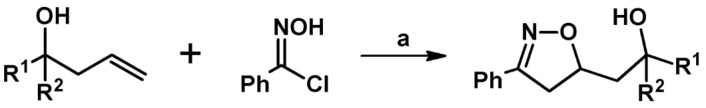
Syntheses of 3,5-disubstituted isoxazolines via 1,3-DP cycloaddition of in situ-generated PhCNO to homoallyl alcohols [27]. R^1^, R^2^ = Me, H; Et, H; *n*-Pr, H; Bn, H; *n*-Bu, H; Ph, H; Me, Ph; a = CH_2_Cl_2_, Et_3_N or THF, EtMgBr; 65–83% yields.

**Scheme 24:**
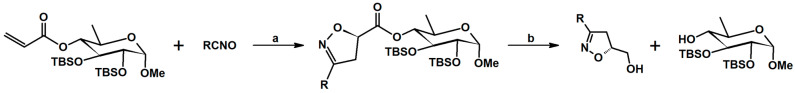
Syntheses of chiral, (*R*)-enriched 5-hydroxymethyl-2-isoxazolines involving a carbohydrate auxiliary [28]. R = Ph or Me_3_C; a = CH_2_Cl_2_, rt; b = Li[BEt_3_H], THF, rt; yield (R, isoxazoline, template) = Ph, 99%, 98%; Me_3_C, 89%, 92%; *dr* = 99/1.

**Scheme 25:**
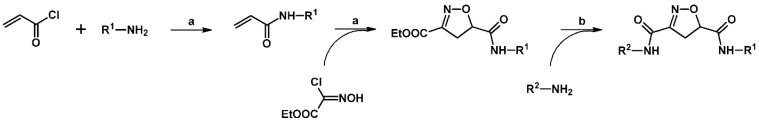
Syntheses of 3,5-disubstituted 2-isoxazolines in ionic liquids [29]. R^1^, R^2^ = cyclohexyl, Ph, *i*-Pr, Bn (16 products). a = KHCO_3_, [BMIM][BF_4_]; b = AlMe_3_, toluene, [BMIM][BF_4_]; up to 50% yield (isoxazolines with two amido groups).

**Scheme 26:**
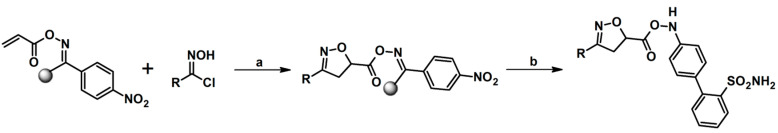
Syntheses of 3,5-disubstituted isoxazolines applying 1,3-DP cycloaddition of ArCNO to Kaiser-oxime-resin-tethered dipolarophiles [30]. R = *p*-ClC_6_H_4_, *m*-F_3_CC_6_H_4_, 3,4-F_2_C_6_H_3_, *m*-MeOC_6_H_4_, *m*-ClC_6_H_4_, *n*-C_5_H_9_, 3-H_2_N-4-ClC_6_H_3_, 1-naphthyl, thiophen-2-yl and others (50 examples); a = Et_3_N, CH_2_Cl_2_, 12 h, rt; b = *p*-H_2_NC_6_H_4_-C_6_H_4_(*o*-SO_2_NH_2_), MeCOOH, DMF, rt, 12 h; up to 61% yield.

**Scheme 27:**
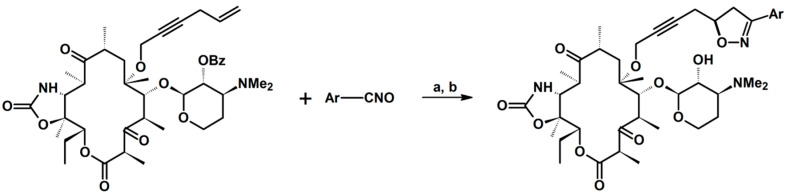
1,3-DP cycloaddition as a crucial step in the modification of the erythromycin A structure [31]. a = Et_3_N, PhH, rt (two diastereoisomers, 1/1 ratio); b = MeOH, rfx; 60% yield (after two steps); Ar = Ph, quinolin-3-yl, pyridin-3-yl, and others.

**Scheme 28:**
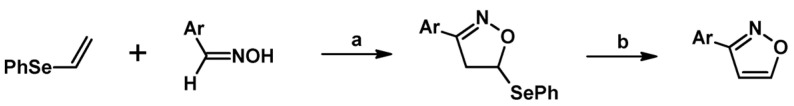
Syntheses of 3-aryl isoxazoles via 1,3-DP cycloaddition of ArCNO to phenyl-vinyl selenide followed by the cycloadduct aromatization [32]. Ar = *p*-MeC_6_H_4_, *p*-MeOC_6_H_4_, *p*-O_2_NC_6_H_4_, 2-Furyl, *n*-Pr, and others; a = NCS, Et_3_N, CHCl_3_, rt, 12h; b = 30% H_2_O_2_, 0 °C, rt, 30 min.; up to 86% yield (isoxazolines).

**Scheme 29:**
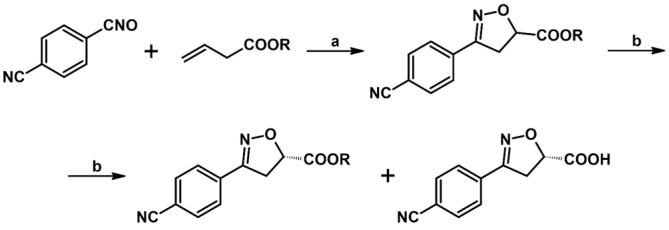
Large-scale synthesis of Roxifiban precursor (starting from 50 kg *O*- or *S*-esters) [34]. R = *O*-*i*Bu or *S*-*i*Bu; a = DMF, 90–95%; b = phosphate buffer, Amano PS 30 Lipase; overall yield = 38%; *ee* ≥ 99% (for *O*-ester); overall yield = 80%, *ee* > 99.9 (for *S*-ester).

**Scheme 30:**
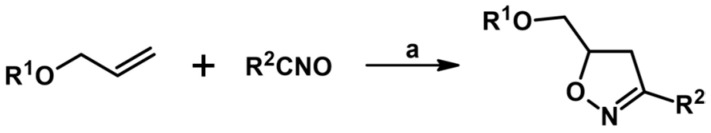
Syntheses of 3-aryl-5-aryloxymethyl-2-isoxazolines [35]. R^1^, R^2^ = 4,6-Br_2_C6H3, Ph; *p*-ClC_6_H_4_, *p*-BrC_6_H_4_; 2,4,5-Cl_3_C6H2, Ph; *p*-O_2_NC_6_H_4_, Ph and others; a = not given; up to 61% yield.

**Scheme 31:**
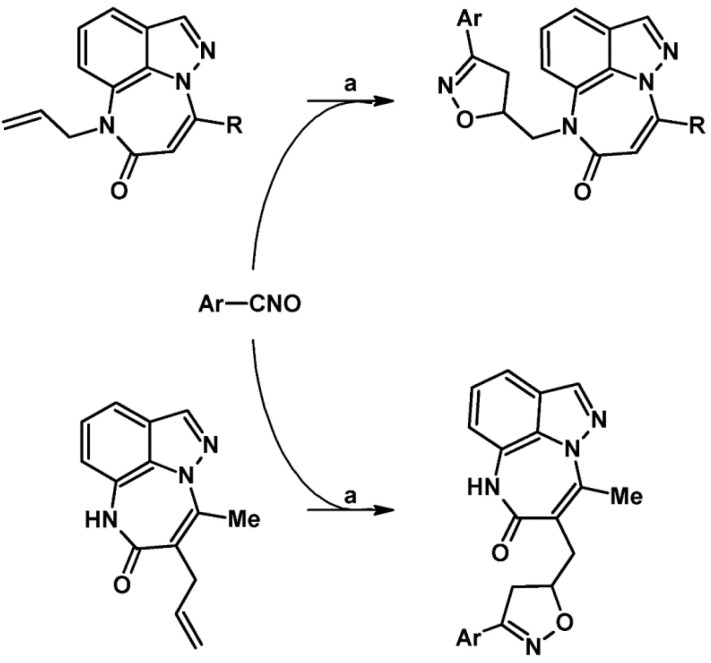
Syntheses of two types of isoxazoline–benzodiazepine hybrids via 1,3-DP cycloaddition [36]. Ar = 2,4,6-(Me)3C6H2; a = benzene, rfx, 4 days; 80% and 85% yield (for R = Me or Ph, respectively; upper product); 90% yield (bottom product).

**Scheme 32:**
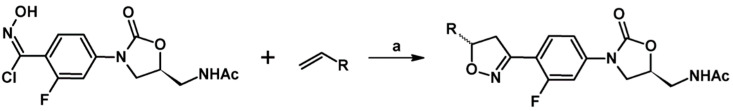
Syntheses of 3,5-disubstituted 2-isoxazolines bearing, tethered by fluorophenylene linker, oxazolidinone motif in position 5 [37]. R = CO_2_Et, C(O)NH_2_, 2-pyridyl, 4-pyridyl, CH_2_NHAc, CN, and others; a = Et_3_N, DMF, yield not given.

**Scheme 33:**
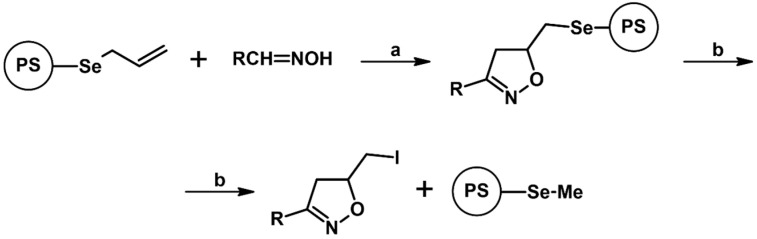
Reusable polystyrene-supported allyl selenide involved in the syntheses of 3,5-disubstituted 2-isoxazolines [38]. PS = polystyrene support; R = Ph, *p*-FC_6_H_4_, *p*-O_2_NC_6_H_4_, *p*-BrC_6_H_4_, *p*-ClC_6_H_4_, *p*-MeC_6_H_4_, COOEt; a = NCS, CH_2_Cl_2_, Et_3_N, rt, 24 h; b = MeI, NaI, DMF, 80 °C, 24 h; up to 87% yield.

**Scheme 34:**
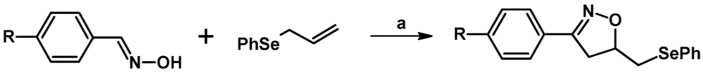
Syntheses of isoxazolines equipped with CH_2_SePh motif in position 5 [39]. R = H, 4-Br, 4-Me, 4-MeO, and others; 86% yield; a = Et_3_N, CH_2_Cl_2_, rt.

**Scheme 35:**
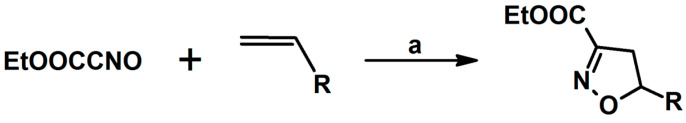
MW-assisted syntheses of isoxazoline–POSS hybrids as macromers [40]. R = *c*-(C_5_H_9_)_7_Si_8_O_12_; *p*-[(c-C_5_ H_9_)_7_Si_8_O_12_]-C_6_H_4_; a = MW (80 W), CHCl_3_; up to 75% yield.

**Scheme 36:**
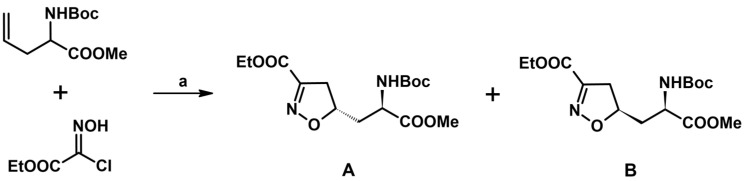
Syntheses of amino acids possessing 3,5-disubstituted isoxazoline moiety [41]. a = NaHCO_3_, AcOEt, yield not given. (**A**,**B**) = mixture of products.

**Scheme 37:**
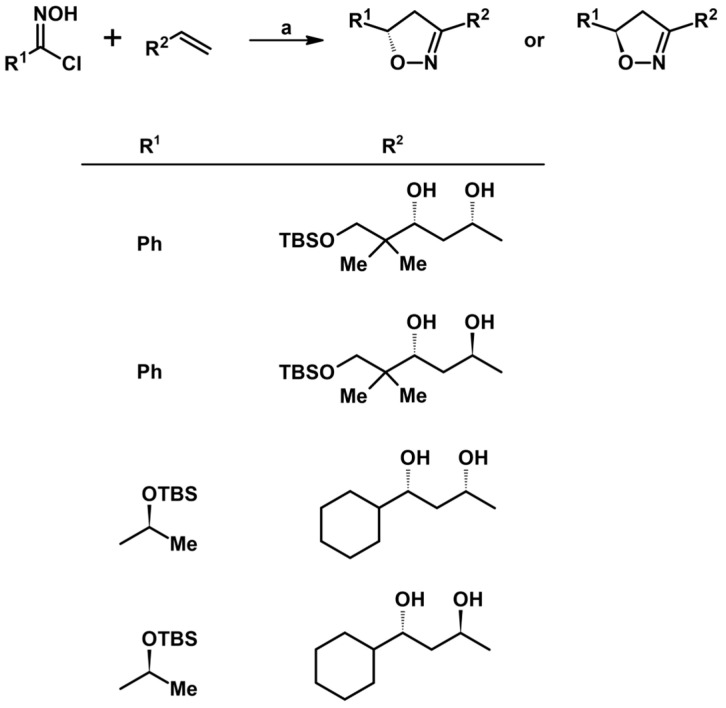
Highly regio- and stereoselective *Mg*-mediated 1,3-DP cycloaddition of RCNO to chiral dipolarophiles leading to chiral 3,5-disubstituted 2-isoxazolines [42]. a = EtMgBr, CH_2_Cl_2_, up to −30 °C, up to 18 h; up to 71% yield; *de* ≥ 95%.

**Scheme 38:**
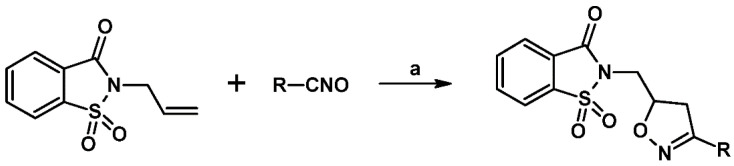
Syntheses of saccharin-CH_2_-3,5-disubstituted isoxazoline hybrids [43]. R = Me, Ph, *o*-ClC_6_H_4_, *p*-BrC_6_H_4_, 3,4-(MeO)_2_C_6_H_3_, *p*-Me_2_NC_6_H_4_, and others; a = NCS, CHCl_3_, next Et_3_N; up to 73% yield.

**Scheme 39:**
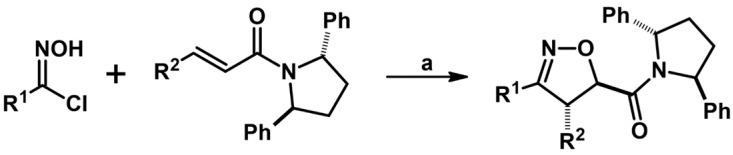
Syntheses of chiral isoxazolines: dipolarophile with a chiral auxiliary (the best results are presented) [44]. R^1^, R^2^ = Ph, H; Ph, Ph; 2,6-Cl_2_C6H3, H; 3-Me-2,6-Cl_2_C6H2, H; cyclohexyl, Ph; *i*-Pr, H and others; a = Et_3_N or Et_2_O or CH_2_Cl_2_, rt, up to 10 days; up to 98% yield; de = usually > 99%.

**Scheme 40:**
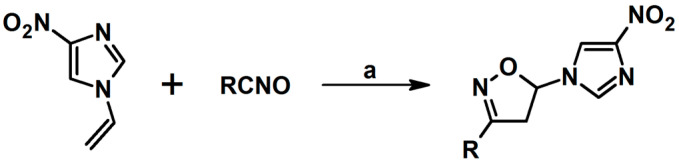
Syntheses of isoxazolines bearing nitroimidazole moiety [45]. R = THPOCH_2_, Ph; a = Et_3_N, THF, rt, 24 h; 31% yield (R = Ph) and 61% yield (R = THPOCH_2_).

**Scheme 41:**
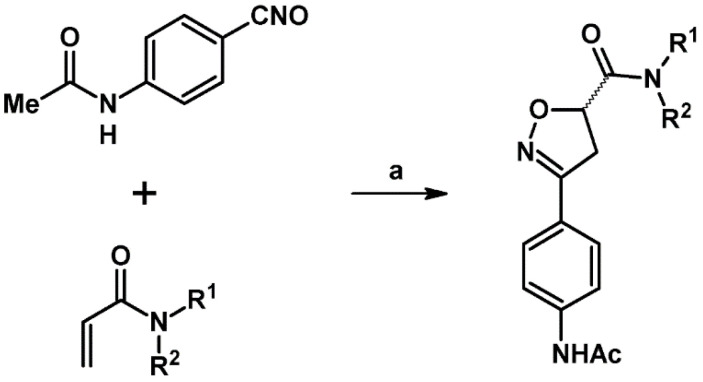
29G12 Antibody as catalyst for enantioselective DP cycloaddition of ArCNO to acrylamide [46]. a = Et_3_N or chloramine-T, EtOH, rt or rfx, up to 8 h, up to 77% yield; R^1^, R^2^, %*ee*, k_rel_ = Me, Me, 98, 1; H, *t*-Bu, 94, 29; H, *i*-Pr, 85, 38; H, *sec*-Bu, 97, 53; H, Ph, 71, 67.

**Scheme 42:**
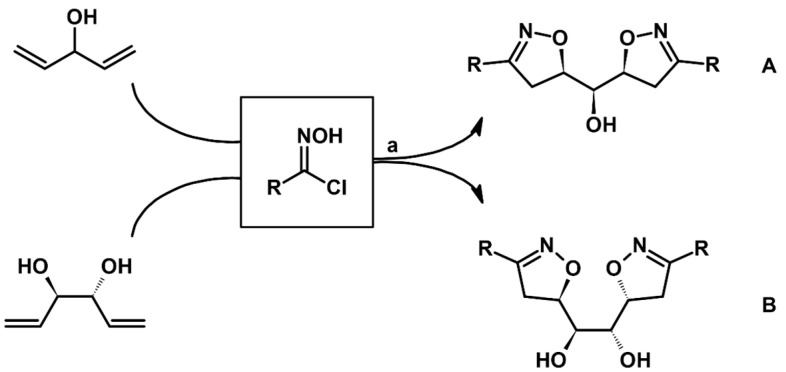
Double diastereoselective 1,3-DP cycloaddition engaged in the syntheses of *syn*,*syn*-bis(1,2-isoxazolin-5-yl)methanols and syn,syn,syn-1,2-bis(1,2-isoxazol-5-yl)ethane-1,2-diols [47]. R (in A) = Ph, Mes, Ph_2_CH, and others; R (in B) = Ph, Mes, Ph_2_CH; a = EtMgBr, CH_2_Cl_2_, 0 °C, 1 h; up to 67% (**A**) and 76% (**B**) yields.

**Scheme 43:**
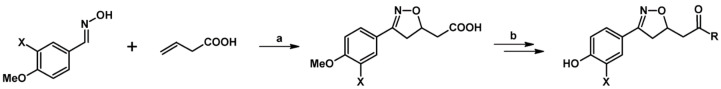
Preparation of 3,5-disubstituted isoxazolines—the method for modified MIF [48]. X = H or F; R = PhO, Mes, EtO, and others; a = DMF, NCS, next Et_3_N; quantitative yield; b = next steps.

**Scheme 44:**
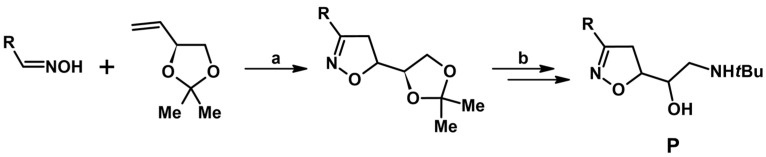
1,3-DP cycloaddition to chiral dipolarophile as a crucial step in the syntheses of enantiopure, especially functionalized isoxazolines [49]. R = Br, CH_3_C(=CH_2_), cyclopropyl; a = BTMA, ICl_4_, CH_2_Cl_2_, then AcOEt, NaHCO_3_; overall yield 45%; b = next steps. P example: R = cyclopropyl, configuration: 5*S*,α*R*.

**Scheme 45:**
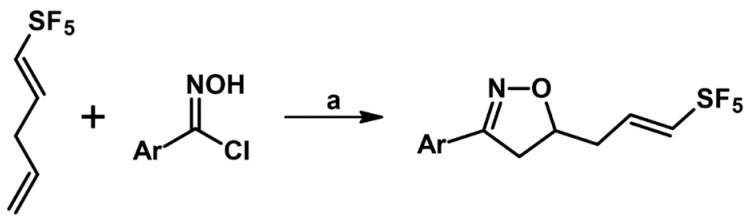
Syntheses of 3,5-disubstituted 2-isoxazolines possessing a (*E*)-CH_2_CH=CHSF_5_ group in position 5 [50]. Ar = Ph or *p*-FC_6_H_4_; a = Et_3_N, Et_2_O, −40 °C; over 85% yield.

**Scheme 46:**
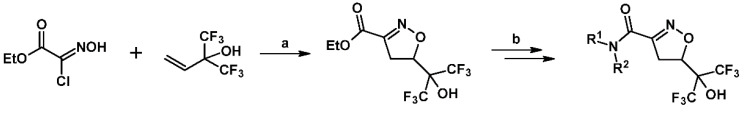
Syntheses of 3,5-disubstituted isoxazolines equipped with the R^1^R^2^NC(O)- group in position 3 and C(OH)(CF_3_)_2_ substituent in position 5 [51]. R^1^, R^2^ = H, alkyl, EtOOCCH_2_-, (S)-*t*-BuOOCCH_2_CH(*t*-Bu)-, and others (more than 30 examples). a = Et_3_N, THF, 50% yield; b = next steps.

**Scheme 47:**
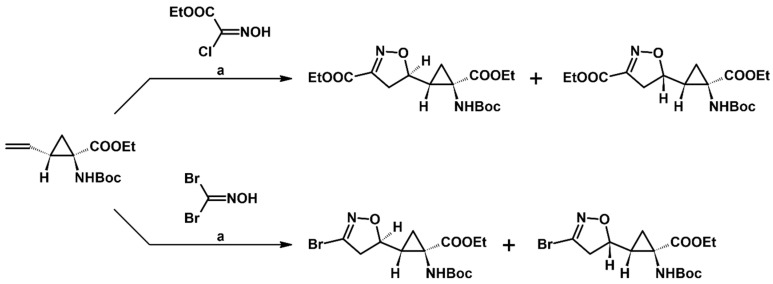
Isoxazolines as a precursor of specially designed homologues of glutamic acid [52]. a = NaHCO_3_, AcOEt; up to 84% yield.

**Scheme 48:**
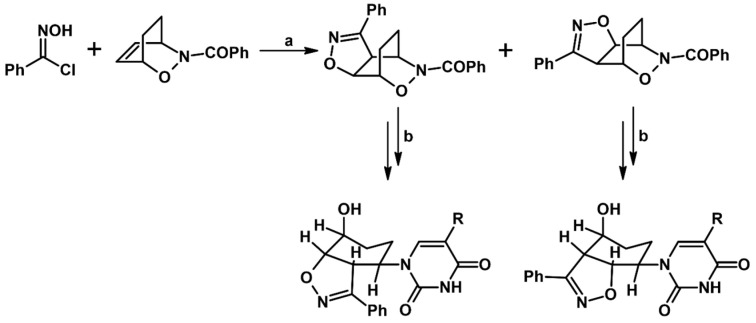
Syntheses of pyrimidine isoxazoline-carbocyclic nucleosides starting from 1,3-DP cycloaddition of in situ-generated PhCNO into specially designed dipolarophile [53]. R = H or Me; a = NMO, CH_2_Cl_2_, rt, 48 h; up to (20 + 22)% yield; b = next steps.

**Scheme 49:**
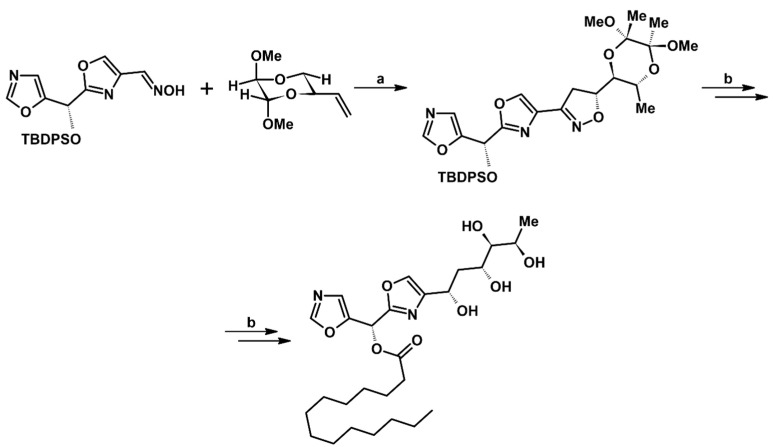
1,3-DP cycloaddition involved in the total synthesis of 10-epi-bengazole A [54]. a = (1) NCS, CH_2_Cl_2_, pyridine, rt; (2) dipolarophile, Cs_2_CO_3_, DME, rt; 72% yield over two steps (dr 2.4:1); b = next steps.

**Scheme 50:**
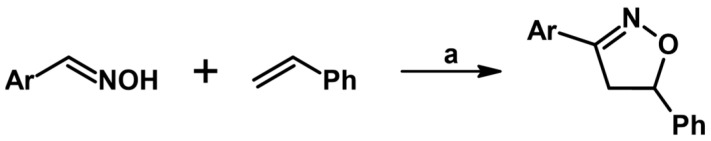
One-pot preparation of 3,5-disubstituted 2-isoxazolines via Y-C_6_H_4_CNO 1,3-DP cycloaddition to styrene [55]. Ar = Y-C_6_H_4_ when Y = H, *p*-Cl, *p*-Me, *p*-O_2_N, and others; a = OXONE, silica-gel, Et_3_N, grinding together, 15 min, rt; 90–92% yield.

**Scheme 51:**
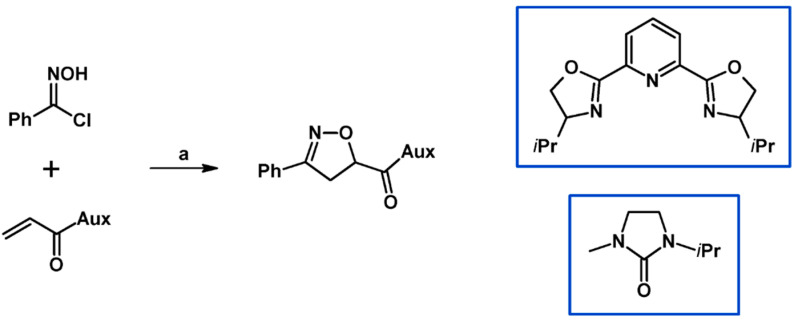
Asymmetric 1,3-DP cycloaddition of PhCNO to acrylamides mediated by chiral Lewis acid–pybox ligand systems [56]. a = Lewis acid (Y(OTf)_3_, MgBr_2_, Mg(ClO_4_)_2_), Et_3_N, CH_2_Cl_2_, chiral pybox ligand (3 examples); auxiliary (3 examples); up to 100% yield; *ee* = up to 87% (for auxiliaries presented in the scheme).

**Scheme 52:**
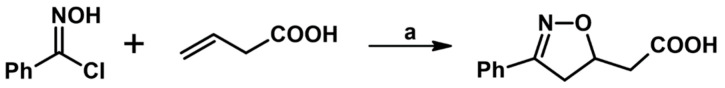
Synthesis of racemic 3-phenyl-5-carboxymethyl-2-isoxazoline [57]. a = Na_2_CO_3_, THF + H_2_O, 12 h; yield was not given.

**Scheme 53:**
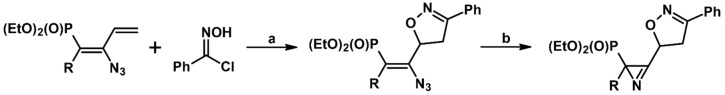
Synthesis of isoxazoline–azirine hybrids via double cycloaddition: nitrile oxide and azide to dienes equipped with azido moiety [58]. R = H, *n*-Pr, *n*-Bu, Ph, CH_2_OH; a = Et_3_N, Et_2_O, −40 °C; b = toluene, 110 °C, 0.5 h; up to 93% yield.

**Scheme 54:**
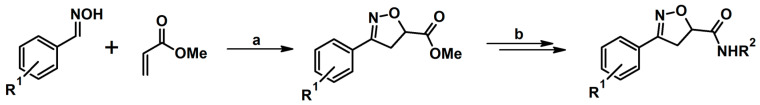
Syntheses of 3,5-disubstituted isoxazolines possessing C(O)NHR^2^ substituent in position 5 [59]. R^1^ = H, 2,6-Cl_2_, 4-O_2_N, 2-O_2_N, 2-F_3_C, and others; R^2^ = *n*-C_4_H_9_, 3-bromobenzyl, 4-methylbenzyl, 4-methoxybenzyl, 4-methoxyphenethyl, 4-methylphenethyl, and others; a = Et_3_N, CH_2_Cl_2_, NaOCl; b = next two steps; overall yield > 65%.

**Scheme 55:**
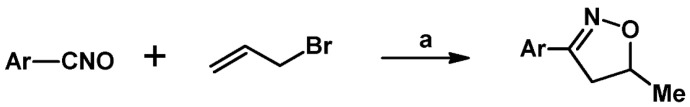
Indium-mediated 1,3-DP cycloaddition of ArCNO to allyl bromide leading to 3,5-disubstituted 2-isoxazolines [60]. Ar = Ph, 2,6-Cl_2_C_6_H_3_, *p*-MeOC_6_H_4_, *o*-O_2_NC_6_H_4_, 2,3-(MeO)_2_C_6_H_3_, and others; a = In, rt, THF-H_2_O, from 0 °C to rt, up to 24 h; up to 84% yield.

**Scheme 56:**
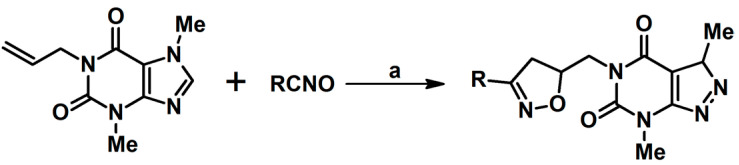
One-pot syntheses of 3,5-disubstituted isoxazolines connected with 3,7-dimethylxantine motif trough CH_2_ bridge [61]. R = Ph, *p*-BrC_6_H_4_, *o*-ClC_6_H_4_, *p*-Me_2_NC_6_H_3_, 2,4-Cl_2_C_6_H_3_, 3,4-(MeO)_2_C_6_H_3_; a = NCS, CHCl_3_, Et_3_N; up to 83% yield.

**Scheme 57:**
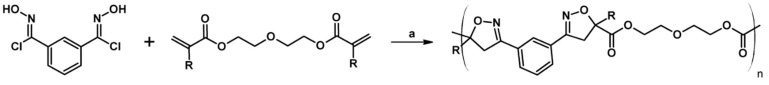
Syntheses of polymers containing 3,5-disubstituted 2-isoxazoline motifs [62]. R = H or Me, a = MS 4Å, DMF, 80 °C, 1 day; 99% yield; M = up to 9000 g/mol.

**Scheme 58:**
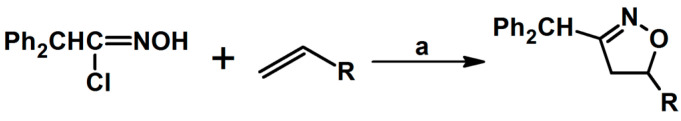
Regioselective cycloaddition of in situ-generated Ph_2_CHCNO to dipolarophiles of CH_2_=CHR type [63]. R = CH_2_OH, Ph, CN, COOEt, OEt; a = Et_3_N, Et_2_O; up to 85% yield.

**Scheme 59:**
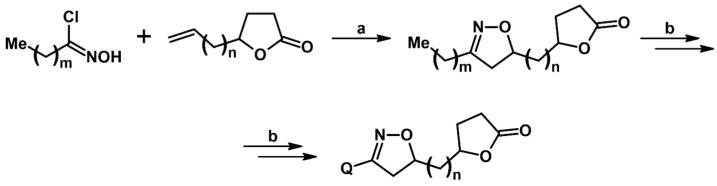
1,3-DP cycloaddition as a crucial step in the syntheses of modified analogues of micromolide [64]. n, m = up to 8; Q = CON(alkyl)_2_, COOEt, COO-*n*-hexyl, and others; a = Et_3_N, 0 °C to rt; up to 73% yield; b = next steps.

**Scheme 60:**
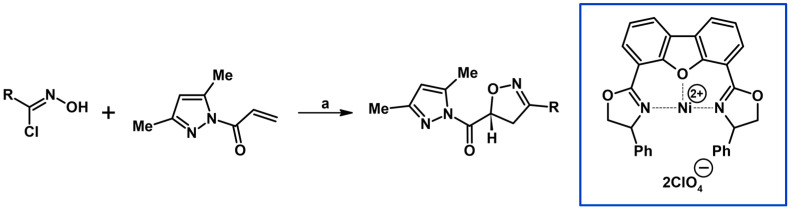
Highly enantioselective syntheses of chiral isoxazolines via cycloaddition mediated by *Ni*-chiral bidentate ligand (equipped with two chiral isoxazoline motifs) [65]. R = Y-C_6_H_4_ where Y = H, *p*-Me, *p*-MeO, *p*-Br, *p*-O_2_N, *o*-Cl, *m*-Cl, and others; a = MS 4Å, R, R-DBFOX/Ph + Ni(BF_4_)_2_·6H_2_O, *i*-PrOH, CH_2_Cl_2_, up to 40 °C; up to 94% yield; *ee* = up to 97%.

**Scheme 61:**
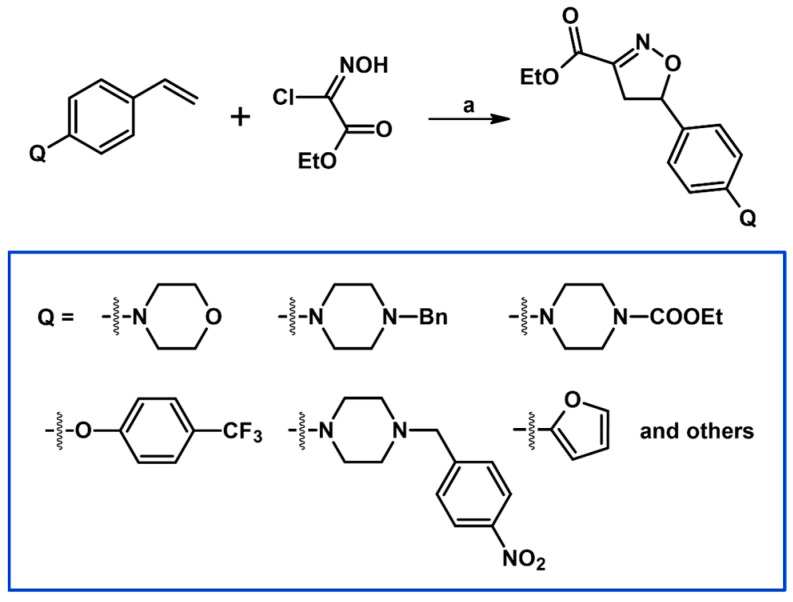
Syntheses of the modified 3-ethoxycarbonyl-5-(*p*-Y-C_6_H_4_)-2-isoxazolines [66]. a = Et_3_N, CH_2_Cl_2_, rt, up to 100% yield.

**Scheme 62:**
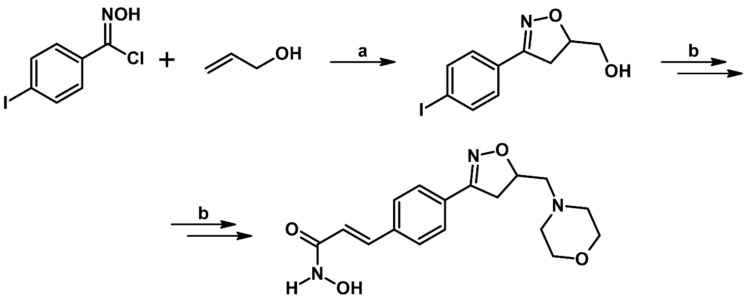
3,5-disubstituted isoxazoline ring creation as a crucial step in the synthetic method leading to the derivative equipped with *N*-hydroxy-(4-oxime)-cinnamide motif [67]. a = Et_3_N (1.5 eq.), CH_2_Cl_2_, rt, 50 °C; yield not reported; b = next steps.

**Scheme 63:**
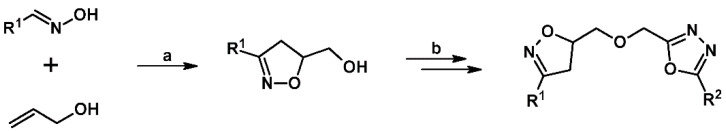
1,3-DP cycloaddition of nitrile oxide to allyl alcohol as a crucial step in the synthetic route leading to 1,3,4-oxadiazole bearing bis(heterocycle) moieties [68]. R^1^ = *p*-MeC_6_H_4_, 2,4,6-(MeO)_3_C_6_H_2_, *p*-O_2_NC_6_H_4_, 2,4-Cl_2_C_6_H_3_, and others; a = chloramine-T, EtOH, 3 h, yield not given; b = next steps.

**Scheme 64:**
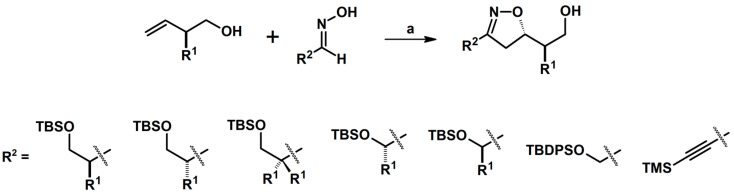
*Mg*-mediated 1,3-DP cycloaddition of various RCNO to (*S*)-2-methyl-3-butenol: syntheses of chiral 3,5-disubstituted 2-isoxazolines [69,70]. R^1^ = Me, CH_2_OTIPS, CH_2_OTBS, and others; a = *t*-BuOCl, CH_2_Cl_2_, or PhMe, −78 °C, then *i*-PrOH, EtMgBr, 0 °C to rt; up to 89% yield, *dr* = from 2:1 to 21:1.

**Scheme 65:**
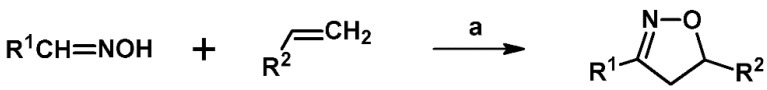
Hypervalent iodine (DIB) oxidation and 1,3-DP cycloaddition engaged in the syntheses of isoxazolines from oximes and alkenes [71]. R^1^, R^2^ = Ph, Ph; *p*-MeO, Ph; *m*-O_2_N, Ph; PhCH_2_CH_2_, Ph; Ph, Br(CH_2_)_4_ and others; a = MeOH, CF_3_COOH (catalytic amount), DIB, rt; up to 91% yield.

**Scheme 66:**
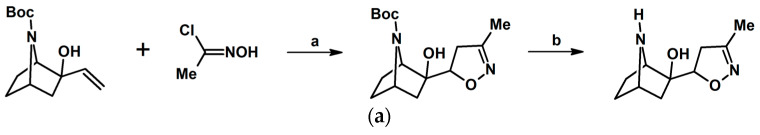

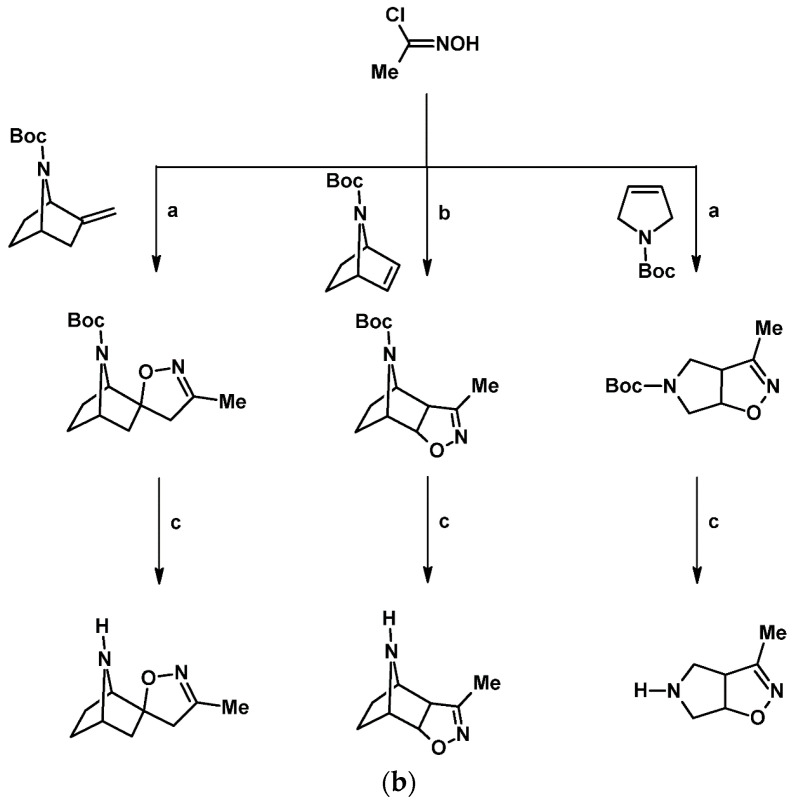
Syntheses of spiro- and bicyclo- isoxazolines—analogues of Epiboxidine—engaging 1,3-DP cycloaddition [72]. (**a**) a = Et_3_N, CH_2_Cl_2_, 48% yield; b = 30% CF_3_COOH, CH_2_Cl_2_ 63%, and 67% isolated yields of two obtained stereoisomers; (**b**) a = ClCH_2_CH_2_Cl, Et_3_N, rfx, 4 days, 11% and 27% yield; b = CH_2_Cl_2_, Et_3_N, rt, 4 h, 87% yield; c = typical removing conditions.

**Scheme 67:**
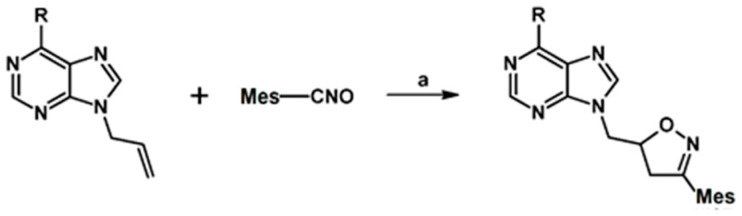
1,3-DP cycloaddition of MesCNO involved in the syntheses of purine and adenine derivatives possessing 2-isoxazoline moiety [73]. a = toluene, rfx, 2–6 days, up to 62% yield; R = Cl, piperidine, morpholine, pyrrolidine.

**Scheme 68:**
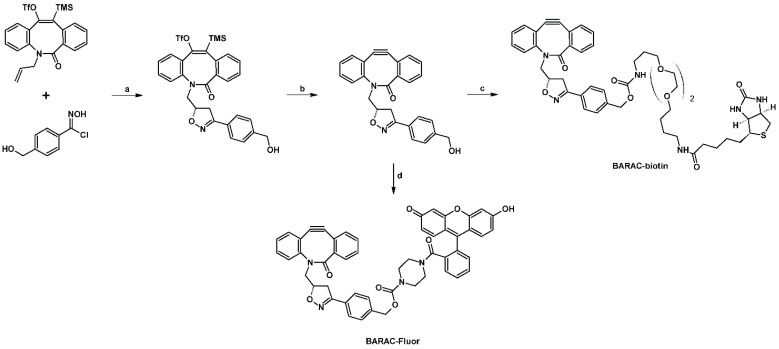
Syntheses of biarylazacyclooctynone derivatives containing 2-isoxazoline motif [74]. a = Et_3_N, CH_2_Cl_2_, 24 h, rt, 47% yield; b = CsF, CH_3_CN, 45 min, rt, 85% yield; c = *N*-(13-amino-4,7,10-trioxatridecanyl)biotinamide, CH_2_Cl_2_, Et_3_N, 3 h, rt and then TBAF, THF, 45 min, rt, 60% yield; d = piperazine-fluorescein, CH_2_Cl_2_, THF, Et_3_N, 6 h, rt and then 45 ^o^C, 5 h, next TBAF, 30 min, rt, 69% yield.

**Scheme 69:**
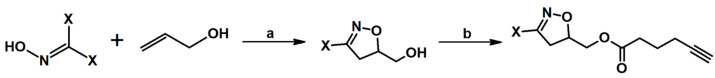
Syntheses of 3-halogeno-5-hydroxymethyl 2-isoxazolines [75]. X = Br or Cl; a = K_2_CO_3_, EtOAc, 24 h, rt; yield 73% for X = Br and 2% for X = Cl; b = hexynoic acid chloride, CH_2_Cl_2_, Et_3_N, 16 h, rt; yield 71% for X = Br and 28% for X = Cl.

**Scheme 70:**
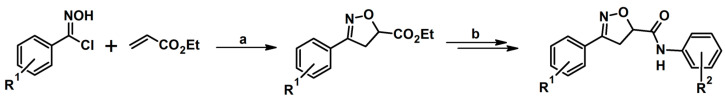
Creation of isoxazoline ring via 1,3-DP cycloaddition as a crucial step in the syntheses of isoxazole derivatives [76]. R^1^ = Alkyl, OC_6_H_4_, OH, Cl, CF_3_; R^2^ = Alkyl, OMe, OCF_3_, Cl, Br, CF_3_, CN, NO_2_, and others; a = PhMe, Et_3_N, anhydrous MgSO_4_, rt, overnight, up to 96% yield; b = next steps, up to 95% yield.

**Scheme 71:**
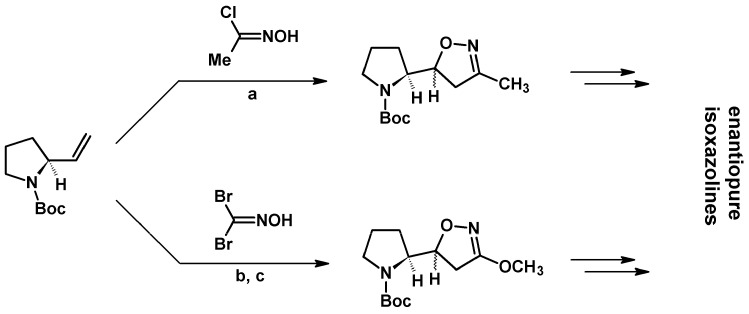
Syntheses of enantiopure chiral, stereoisomeric pyrrolidine–isoxazoline hybrids involving the 1,3-DP cycloaddition of in situ-generated MeCNO and BrCNO to (*S*)-(-)-*N*-Boc-2-vinylpyrrolidine [77]. a = CH_2_Cl_2_, Et_3_N, rt, 1 week; b = AcOEt, NaHCO_3_, rt, 48 h; c = K_2_CO_3_, MeOH, rt, 12 h; up to 94% yield.

**Scheme 72:**
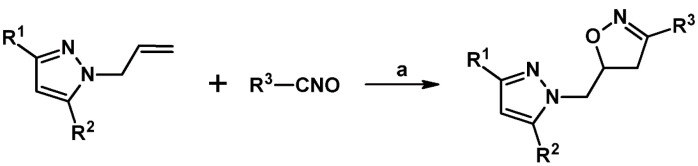
Syntheses of 3,5-disubstituted isoxazolines combined through –CH_2_– linker with pyrazole motifs [78]. R^1^ = *ortho*-substituted phenyl; R^2^ = CO_2_Et; R^3^ = aryl, hetaryl, and others; a = R^3^CNO were generated from R^3^CH=NOH or R^3^C(Cl)=NOH or R^3^CH_2_NO_2_, Et_3_N or NaOCl; up to 97% yield.

**Scheme 73:**
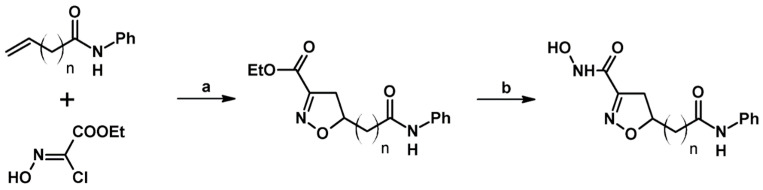
Syntheses of 3,5-disubstituted isoxazolines equipped with unique functional groups [79]. a = NaHCO_3_, AcOEt, MW, 100 °C, up to 98% yield; b = NH_2_OH*HCl, KOH, MeOH, up to 46% yield.

**Scheme 74:**
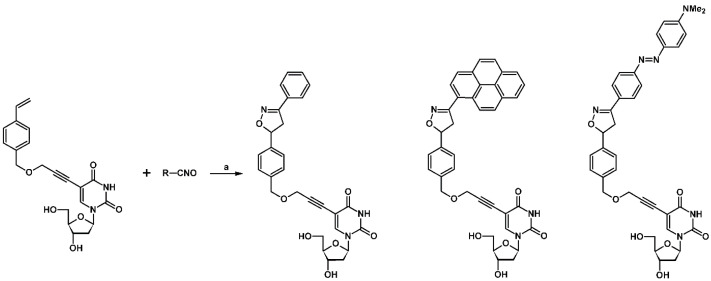
Creation of 3,5-disubstituted isoxazoline motif as a linker in the modified DNA fragments [80]. a = click reaction with nitrile oxide precursor.; R = Ph, pyrene, dabcyl dye, alkynetriphosphate, galaxtose azide; yield was not given.

**Scheme 75:**
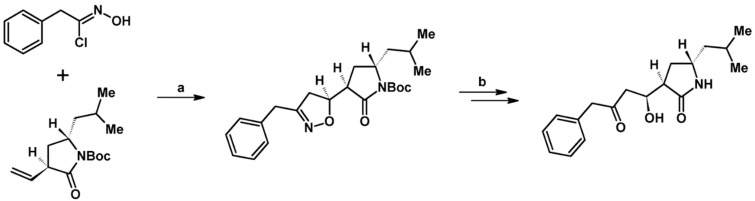
Total synthesis of (-)-berkeleyamide A: preparation of the crucial intermediate containing 3,5-disubstituted isoxazoline motif [81]. Only the reaction path leading to the desired product is presented. a = Et_3_N, CH_2_Cl_2_, from −78 to −45 °C, from 2 to 7 h; 53% yield; b = next steps; 81% yield.

**Scheme 76:**
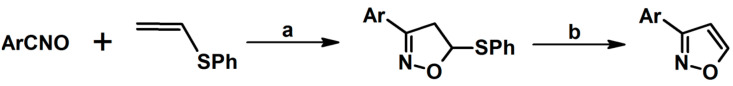
Syntheses of 3-aryl-5-thiophenyl-2-isoxazolines and 3-aryl isoxazoles via 1,3-DP cycloaddition of ArCNO to phenyl-vinyl sulfide followed by the cycloadduct aromatization [82]. Ar = Ph, *p*-MeC_6_H_4_, *p*-MeOC_6_H_4_, *o*-F_3_CC_6_H_4_, *o*-MeC_6_H_4_, and others; a = 12 h, rt, THF; b = NaOMe, rt, MeOH, 3 h; up to 90% isoxazoline yield and up to 91% isoxazole yield.

**Scheme 77:**
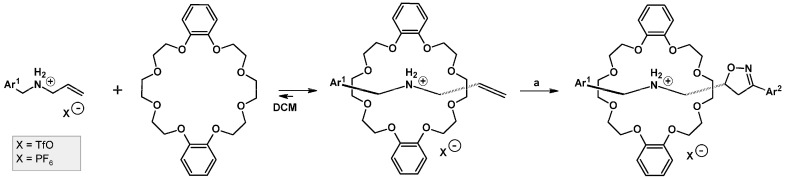
Syntheses of rotaxane possessing 3,5-disubstituted 2-isoxazoline moiety [83]. Ar^1^ = 3,5-Me_2_C_6_H_3_; Ar^2^ = 2,6-(MeO)_2_C_6_H_3_; X = TfO, PF_6_; a = rt, 1 day, CH_2_Cl_2_; up to 98% yield.

**Scheme 78:**
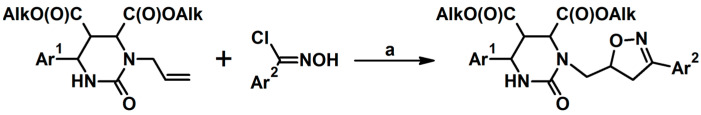
Syntheses of 3,5-disubstituted 2-isoxazolines equipped with oxo-pyrimidine motifs in position 5 [84]. Ar^1^, Ar^2^ = Ph, *m*-BrC_6_H_4_, *m*-NO_2_C_6_H_4_, *p*-NO_2_C_6_H_4_, 3,4-Cl_2_C_6_H_3_, 3,4-Cl_2_C_6_H_3_, *p*-FC_6_H_4_, *p*-MeOC_6_H_4_; Alkyl = Me, Et; a = Et_3_N, CH_2_Cl_2_, from −15 °C to rt, 12 h; 75% yield.

**Scheme 79:**
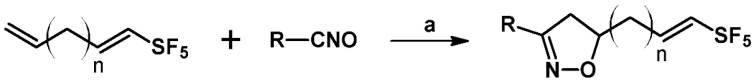
Syntheses of isoxazolines modified by (*E*)-(CH_2_)_n_CH=CHSF_5_ substituent. RCNO were generated in situ from appropriate oxymoyl chlorides [86]. R = MeCO, Ph, *p*-FC_6_H_4_; n = 0 or 1; a = Et_2_O, −20 °C, Et_3_N or CAN, acetone, rfx; up to 85% yield.

**Scheme 80:**
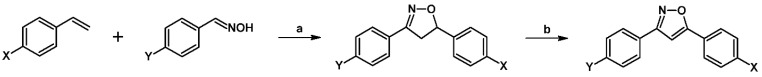
1,3-DP cycloaddition of in situ-generated nitrile oxide to styrene derivatives as an initial step in the syntheses of 3,5-disubstituted isoxazoles [87]. X, Y = Br, C_7_H_15_O; Br, C_17_H_25_O; Me, C_8_H_17_O; Br, Br; Cl, O_2_N; *t*-Bu, Br; and others; a = NCS, Py, CH_2_Cl_2_, 4 h, from 0 °C to rt; b = MnO_2_; 14–93% yields (for isoxazolines); b = MnO_2_, benzene; 47—99% (for isoxazoles).

**Scheme 81:**
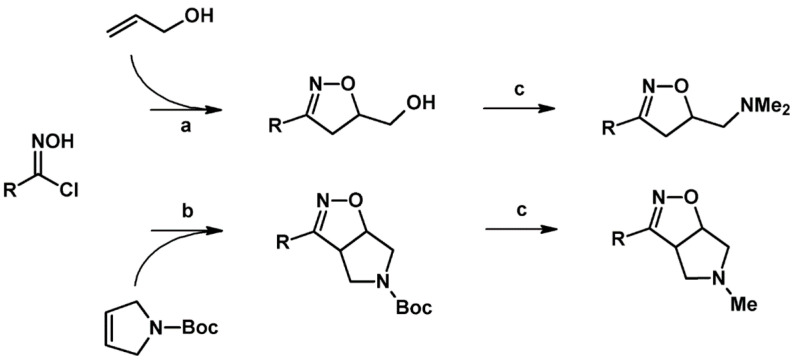
Syntheses of 3,5-di- and 3,4,5-trisubstituted isoxazolines via 1,3-DP cycloaddition of RCNO into allyl alcohols and *N*-protected dihydropyrrole [88]. R = substituted phenyl or benzyl (*p*-O_2_NC_6_H_4_, *p*-MeOC_6_H_4_, *p*-HOOCC_6_H_4_, *p*-O_2_NBn, *p*-MeOBn, and others); a = NaOCl, CH_2_Cl_2_, Et_3_N, 0 °C—rt; b = K_2_CO_3_, AcOEt, 80 °C; c = next step; up to 81% yield.

**Scheme 82:**
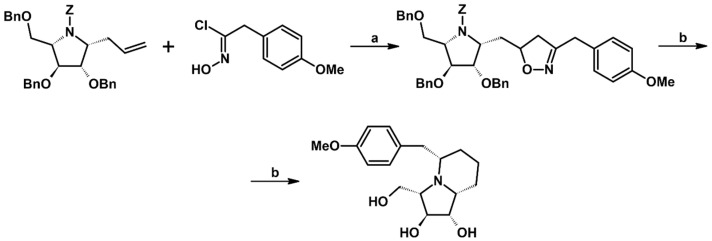
1,3-DP cycloaddition as a crucial step in the synthetic method leading to the analogue of the natural (-)-steviamine [89]. a = Et_3_N, CH_2_Cl_2_, rt, 67% yield (isoxazoline); b = next steps.

**Scheme 83:**
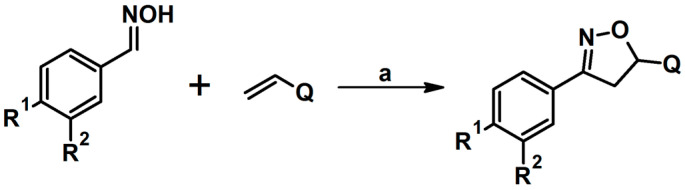
Syntheses of 3,5-disubstituted 2-isoxazolines via 1,3-DP cycloaddition of RCNO to CH_2_=CHQ dipolarophiles [90]. R^1^, R^2^, Q = HO, H, CH_2_COOH; HO, MeO, CH_2_COOH; MeO, MeO, COMe; Me, Me, COOMe; HO, MeO, CH_2_O(*p*-HOCC_6_H_4_), and others; a = NCS, Et_3_N, DMF, rt, 5 h; up to 64% yield.

**Scheme 84:**
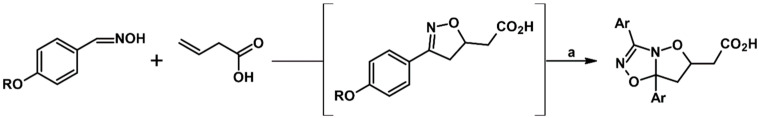
Double 1,3-DP cycloaddition engaged in the synthetic route leading to liquid crystals [91]. Ar = *p*-ROC_6_H_4_; R = C_n_H_2n+1_O, n = 7, 8, 9, and 10; a = NCS, Py, CHCl_3_, up to rt, 4 h; up to 53% yield (for final products).

**Scheme 85:**
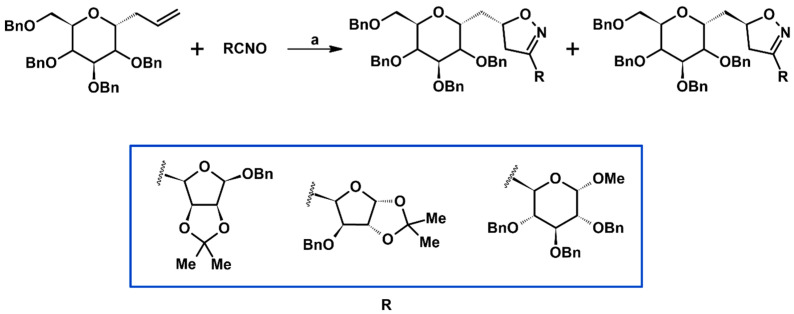
Syntheses of disaccharide combined by isoxazoline motif created via 1,3-DP cycloaddition of dipole and dipolarophile equipped with saccharide motifs [92]. a = DCE, DMAP, rfx, 3 h; up to 90% yield.

**Scheme 86:**
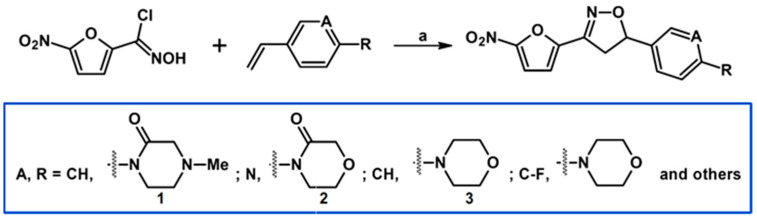
1,3-DP cycloaddition as a crucial step in the syntheses of 3,5-disubstituted 2-isoxazolines connected with nitrofuran and aryl(hetaryl)-saturated heterocycle motifs [93]. a = Et_3_N, CHCl_3_, rt, 2 h; up to 80% yield.

**Scheme 87:**
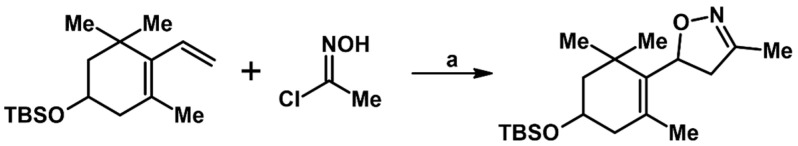
1,3-DP cycloaddition involved in the total synthesis of (±)-3-hydroxy-ß-ionone [94]. a = Et_3_N, CH_2_Cl_2_, rt, 10 h; 75% yield.

**Scheme 88:**
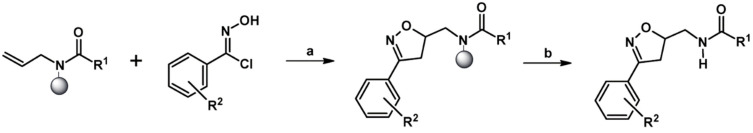
Syntheses of 3,5-disubstituted isoxazolines using resin-bound dipolarophiles [95]. R^1^, R^2^ = piperonyl, H; cyclopentyl, H; *o*-O_2_NC_6_H_4_, *p*-Ph; Ph, 2,6-dichloro; 1-naphthyl, *p*-Ph and others; a = Et_3_N, CH_2_Cl_2_, rt overnight; b = anhydrous HF, 90 min, 0 °C, up to 96% yield.

**Scheme 89:**
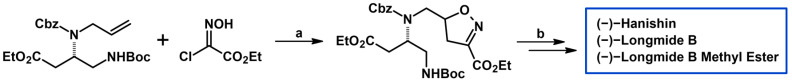
Isoxazoline synthesis via 1,3-DP cycloaddition as a crucial step in the total syntheses of some natural alkaloids [96]. a = NaHCO_3_, AcOEt, rt, 12 h; 97% yield; b = next steps.

**Scheme 90:**
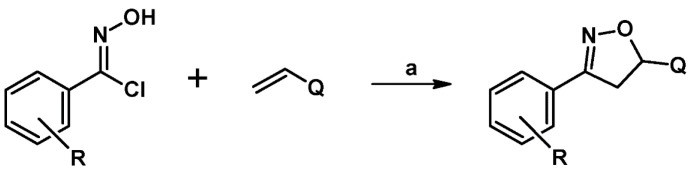
Syntheses of 3,5-disubstituted 2-isoxazolines possessing 2-oxo-pyrrolidin-yl or 4-pyridil substituents in position 5 [97]. Q = 2-oxo-pyrrolidin-1-yl or 4-pyridyl; R = H, *p*-Cl, *m*-Cl, *m*-F_3_C, *p*-O_2_N, *p*-Me, and others; a = Et_3_N, CH_2_Cl_2_, rt, 12 h; up to 93% yield.

**Scheme 91:**
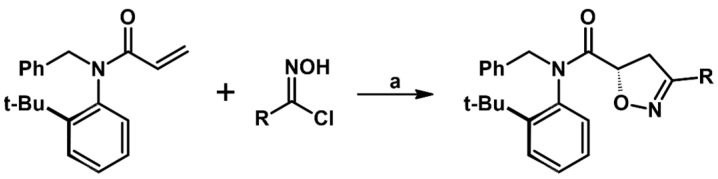
Syntheses of chiral 3,5-disubstituted isoxazolines via 1,3-DP cycloaddition of RCNO to axially chiral acryl amide [98]. *ee* dienophile = 91%; R, %yield, *dr*, %*ee* = Ph, 97, 12:1, 91; *n*-hexyl, 88, >20:1, 90; a = Et_3_N, AcOEt.

**Scheme 92:**
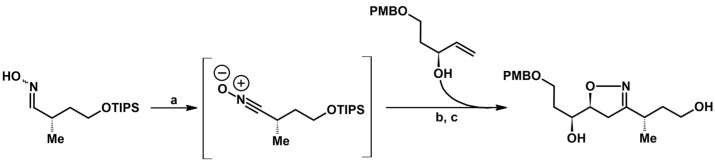
1,3-DP cycloaddition of appropriate nitrile oxide and dipolarophile involved in the synthesis of spirastrellolide methyl ester [99]. a = *t*-BuOCl, CH_2_Cl_2_, −78 °C; b = *i*-PrOH, EtMgBr, CH_2_Cl_2_, 0 °C; c = TBAF, THF, 76% yield (over two steps).

**Scheme 93:**
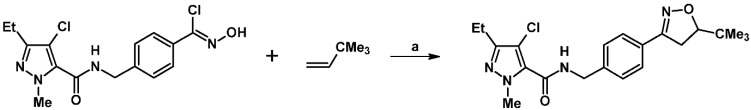
Synthesis of pyrazole-5-carboxamides-isoxazoline hybrid via 1,3-DP cycloaddition of benzonitrile oxide derivative containing pyrazole-carboxyimide motif in the *para*-position to the RCH=CH_2_ type dipolarophile [100]. a = NCS, Py (two drops), oxime in CH_2_Cl_2_-*i*PrOH, rt, next Et_3_N, dienophile in CH_2_Cl_2_, from −10 °C to rt, overnight; 36% yield.

**Scheme 94:**
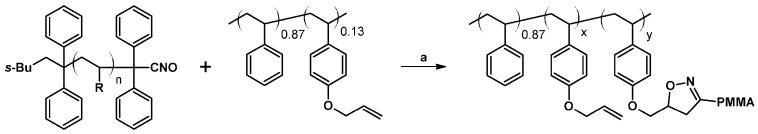
Synthesis of copolymers combined with 3,5-disubstituted isoxazoline motif [101]. R = Ph; n, x, y—depending on the reaction conditions; a = up to 200 °C, conversion = up to 98%; up to 100% yield.

**Scheme 95:**
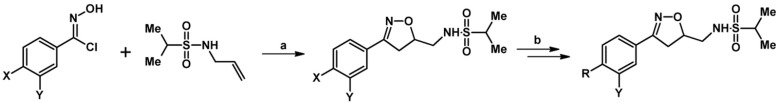
1,3-DP cycloaddition as a crucial step in the synthetic route leading to the “potentiators” [102]. X = H, F; Y = F, H; R = *c*-N(CH_2_)_4_; and others; a = Et_3_N, CH_2_Cl_2_, rt, 20 h, 85–90% yield.

**Scheme 96:**
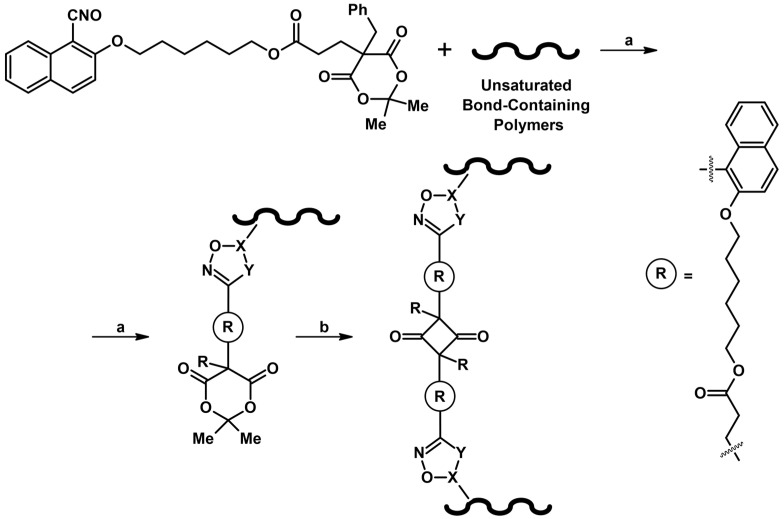
1,3-DP cycloaddition involved in the synthetic strategy leading to the polymers containing isoxazoline motifs (X, Y = C, C or C, N). [103]. R = Bn; X = CH, C(CH_3_), and others; Y = CH, N, and others; a = DMF, up to 70 °C, up to 3 days; up to 99% yield; b = 250 °C, 1 h; up to 99% yield.

**Scheme 97:**
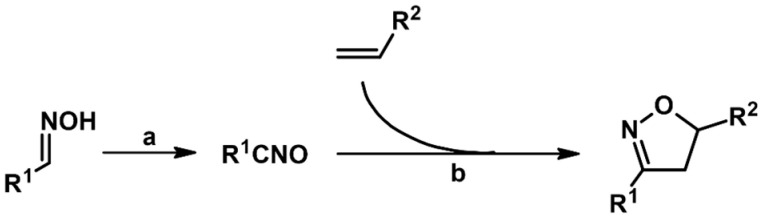
Hypervalent iodine involved in the RCNO generation from oximes, followed by 1,3-DP cycloaddition leading to the 3,5-disubstituted isoxazolines [104]. R^1^, R^2^ = Ph, *n*-Bu; Ph, *p*-BrC_6_H_4_; 2-naphthyl, Ph; Bn, Ph; Ph, Ph; and others; a = *m*-CPBA, PhI, TFE, rt, 30 min; b = TFE, rt, 5 h; up to 96% yield.

**Scheme 98:**
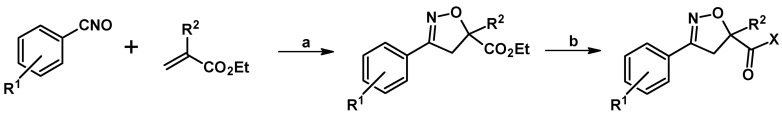
1,3-DP cycloaddition of nitrile oxide to ethyl acrylate or methacrylate involved in the syntheses of hydroxamic acid derivatives [105]. R^1^ = H, 2-, 3-, or 4-Cl, 2-, 3-, or 4-EtO, 4-benzyloxy, and others; R^2^ = H or Me; X = NHOH or NH_2_NH_2_; a = Et_3_N, trichloroisocyanuric acid, 24 h, up to 54% yield; b = next steps.

**Scheme 99:**
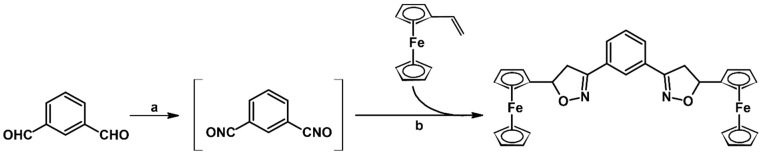
Syntheses of bidentate ligand via 1,3-DP cycloaddition [106]. a = NCS, Et_3_N; b = CH_2_Cl_2_, rt, 24 h; up to 90% yield.

**Scheme 100:**
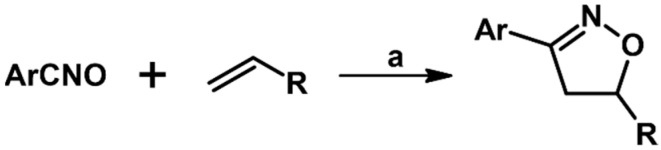
Syntheses of disubstituted isoxazolines using in situ-generated (from ArCH=NOH) ArCNO and RCH=CH_2_ [107]. Ar, R = Ph, Ph; Ph, C_6_H_4_CH_2_; Ph, CO_2_Me; Ph, CH_2_OH; Ph, *n*-Bu; Ph, CH_2_Br; *p*-MeC_6_H_4_, Ph; *p*-MeC_6_H_4_, CO_2_Me; *p*-MeOC_6_H_4_, Ph; a = *m*-ClC_6_H_4_COOOH, PhI, CF_3_CH_2_OH, rt, 12 h; up to 82% yield.

**Scheme 101:**
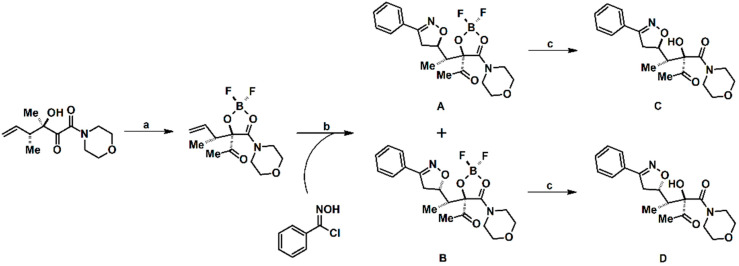
Applying hydroxy-amide dienophile F_2_B-O,O’-complex type for the stereoselective 1,3-DP cycloaddition followed by decomplexation to isoxazoline-hydroxy amide—morpholine hybrids [108]. a = BF_3_·OEt_2_, CH_2_Cl_2_, 70 °C, 16 h; b = Et_3_N, CH_2_Cl_2_, rt, 24 h; c = K_2_CO_3_, MeOH, rt, 1 h; (**A**,**B**) = 83/8; (**C**,**D**) = 56/22.

**Scheme 102:**
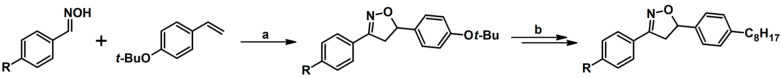
3,5-disubstituted isoxazolines as precursors for liquid crystalline isoxazoles [109]. R = Br, Cl, F, Me, O_2_N; a = NCS, HCl, CH_2_Cl2, Et_3_N, up to rt; up to 59% yield; b = HBr, AcOH, MeOH, next KOH, MeCN, BrC_8_H_17_, up to 84% yield.

**Scheme 103:**
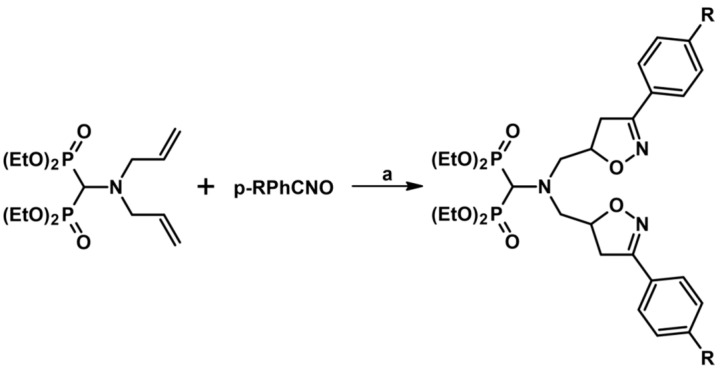
Syntheses of 3,5-disubstituted isoxazolines bearing an aminophosphonate group in position 5 (examples with two isoxazoline groups are presented) [110,111]. ArCNO were generated in situ from ArC(Cl)=CHOH. R = H, Cl, F or Me; a = Et_3_N, CH_2_Cl_2_, up to rt, up to 4 h; up to 65% yield.

**Scheme 104:**
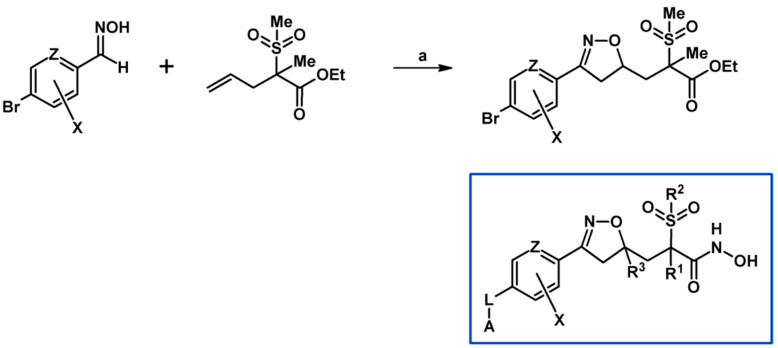
The general structure and crucial step in the synthetic route led to specially designed 3,5-disubstituted isoxazolines [112]. A = alkyl, cycloalkyl, halogen, and others; L = –*S*–, –*O*–, –CC–, and others; L = H, halogen, CN, alkyl, halogenoalkyl, and others; Z = N or C-alkyl, C-halogenoalkyl; R^1^, R^2^, R^3^ = H, alkyl, halogenoalkyl, and others; a = typical conditions (RCNO generation: using NaOCl, *t*-BuOCl, NCS, and other methods); or chiral ligand or chiral HPLC for obtaining a chiral isoxazolines.

**Scheme 105:**
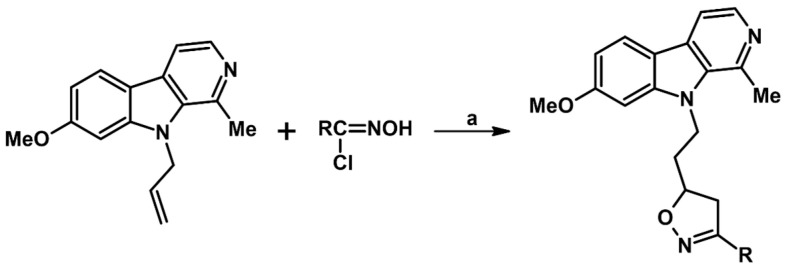
Syntheses of 2-isoxazoline equipped with the harmine motif in position 5 [113]. R = Ph, *p*-MeOC_6_H_4_, *p*-ClC_6_H_4_, 2-thienyl, and others; a = Et_3_N, CH_2_Cl_2_, rfx, 48 h; up to 75% yield.

**Scheme 106:**
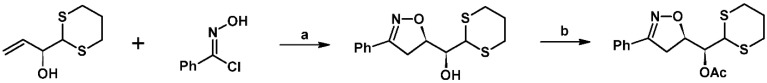
Enantioselective 1,3-DP cycloaddition leading to enantiopure isoxazoline [114]. a = EtMgBr, CH_2_Cl_2_ + *i*-PrOH, up to rt, overnight; 76% yield; b = Ac_2_O, Py; de ≥ 98%.

**Scheme 107:**
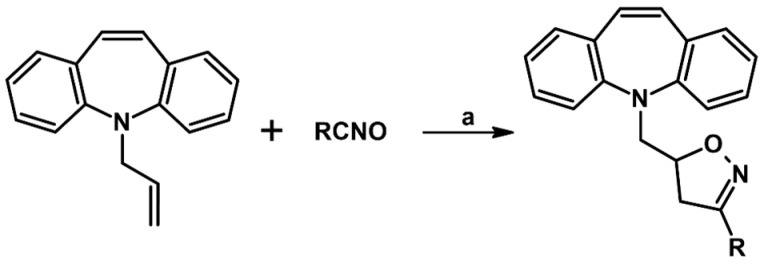
1,3-DP cycloaddition involved in the syntheses of isoxazolines possessing dibenzoazepine motif tethered to position 5 by –CH_2_N– linker [115]. R = *p*-BrC_6_H_4_, *p*-(3-pyridyl)C_6_H_4_, *p*-MeOC_6_H_4_, and others; a = Et_3_N, CHCl_3_, from 0 °C up to rt; 65–85% yield.

**Scheme 108:**
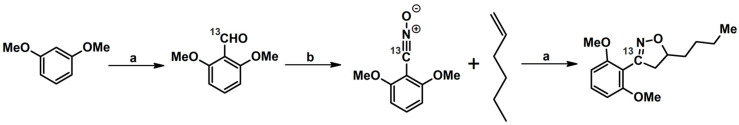
Synthesis of ^13^C-labelled isoxazoline using ^13^C-labelled DMF for the synthesis of labelled nitrile oxide [116]. a = BuLi, ^13^C-DMF, cyclohexane, 0 °C; b = NH_2_OH·HCl, NaOH, EtOH/H_2_O, rt, 10 min then NCS, Et_3_N, CHCl_3_, 0 °C, 10 min; c = CHCl_3_, rt, 17 h; quantitative isoxazoline yield.

**Scheme 109:**
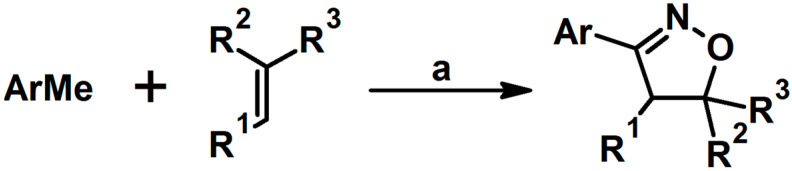
One-pot syntheses of 3,5-disubstituted 2-isoxazolines from arylmethane. Formation of nitrile oxide from arylmethane [117]. Ar, R^1^, R^2^, R^3^ = *p*-XC_6_H_4_ (X = F, Cl, Br, Me, and others), H, H, COO*t*-Bu; 4-pyridyl, H, H, COO*t*-Bu; 2-naphthyl, H, H, COO*t*-Bu; *p*-MeC_6_H_4_, H, H, Ph; *p*-MeC_6_H_4_, H, Me, Ph, and others; a = Pd(OAc)_2_ (5 mol%), AgNO_3_ 4 (eq.), *p*-TosOH (0,5 eq.), *t*-AmOH, 120 °C; up to 78% yield.

**Scheme 110:**
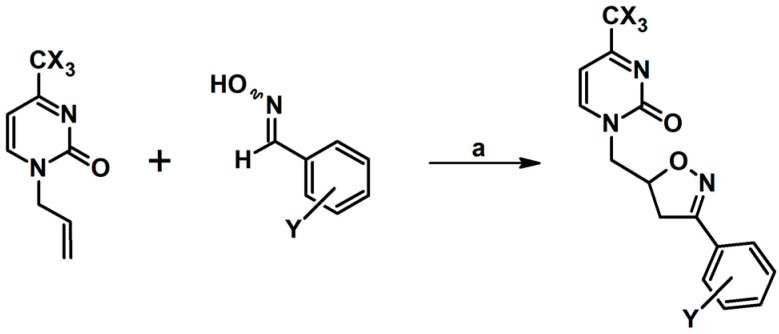
Syntheses of 3,5-disubstituted isoxazolines bearing trihalomethylpyrimidinone and phenyl or substituted phenyl motifs [118]. Y, X = H, Cl; *p*-F, Cl; *o*-Me, Cl; *o*-H, Cl; *o*-MeO, Cl; *p*-MeO, Cl; Ph, F; *p*-F, F; *o*-MeO, F; *p*-MeO, F and others; a = THF, NCS, HCl, rt, 2 h; then Et_3_N, rfx, 4 h; 55–99% yield.

**Scheme 111:**
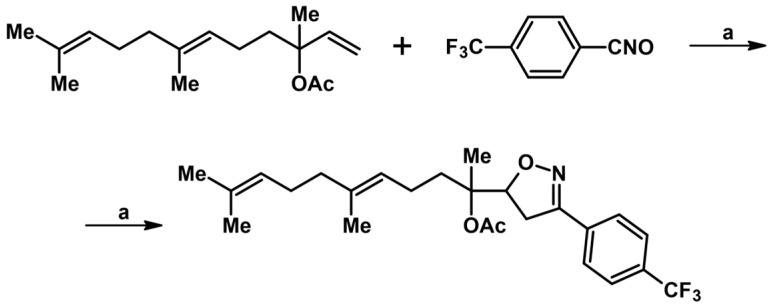
1,3-DP cycloaddition of ArCNO to polyunsaturated esters—an example [119]. a = CH_2_Cl_2_, rt; 30% yield (two diastereoisomers).

**Scheme 112:**
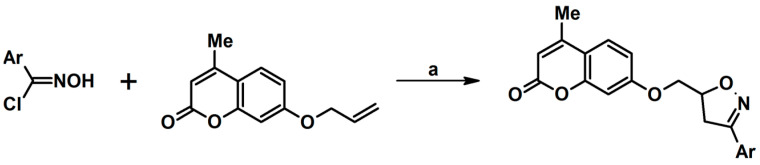
Syntheses of 3,5-disubstituted 2-isoxazolines equipped with the coumarin motif attached to the scaffold through CH_2_O linker [120]. Ar = Ph and XC_6_H_4_ (X = MeO, O_2_N, Et, Cl); a = PhMe, Et_3_N; up to 58% yield.

**Scheme 113:**
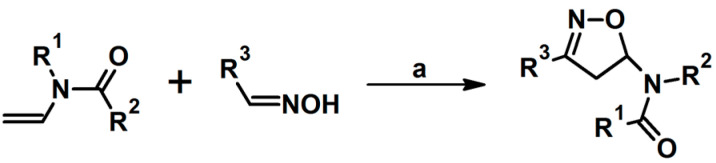
Syntheses of 5-acylamino-2-isoxazolines via 1,3-DP cycloaddition of R^3^CNO to CH_2_=CHN(R^1^)COR^2^ [121]. R^1^, R^2^, R^3^ = Bn, Ph, Et; Bn, Ph, *p*-MeOC_6_H_4_Ph; Bn, Ph, 2-thienyl; 3-PyCH_2_, Ph, *p*-MeC_6_H_4_; 3-PyCH_2_, Ph, 2-thienyl; and others; a = NCS, Et_3_N, PhMe or PhMe + CH_2_Cl_2_, up to rt, 2–18 h; up to 75% yield.

**Scheme 114:**
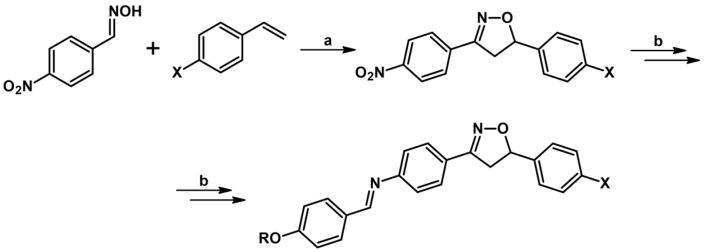
Syntheses of Schiff-base-type liquid crystal materials containing 3,5-disubstituted isoxazoline motif [122]. X, R = Me, *n*-C_6_H_12_; Me, *n*-C_12_H_25_; Br, *n*-C_8_H_17_; *n*-BuO, *n*-C_8_H_17_ and others; a = NaOCl, CH_2_Cl_2_; up to 89% isoxazoline yield; b = next steps.

**Scheme 115:**
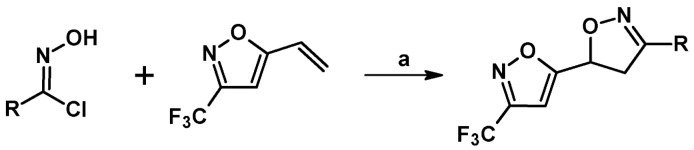
Syntheses of isoxazole–isoxazoline scaffolds using 1,3-DP cycloaddition [123]. R = *p*-MeOC_6_H_4_, *p*-CF_3_C_6_H_4_, CF_3_, CH=CHC_6_H_4_; a = Et_3_N, PhMe, rt, overnight; up to 99% yield.

**Scheme 116:**
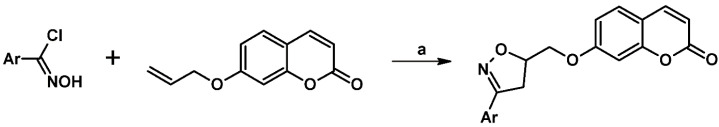
Syntheses of coumarins functionalized by 3,5-disubstituted-2-isoxazoline motif [124]. Ar = Ph, *p*-FC_6_H_4_, *p*-ClC_6_H_4_, 1-naphthyl, *o*-MeC_6_H_4_, and others; a = Et_3_N, THF, rt, 24 h; up to 90% yield.

**Scheme 117:**
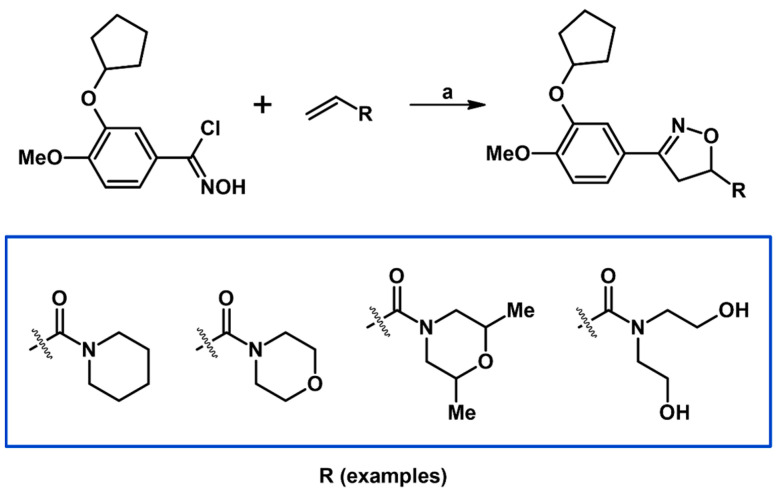
Syntheses of 3,5-disubstituted isoxazolines via 1,3-DP cycloaddition of ArC(Cl)=NOH to QCH=CH_2_ type dipolarophiles [125]. a = Et_3_N, DMF, up to rt, 72 h; up to 76% yield.

**Scheme 118:**
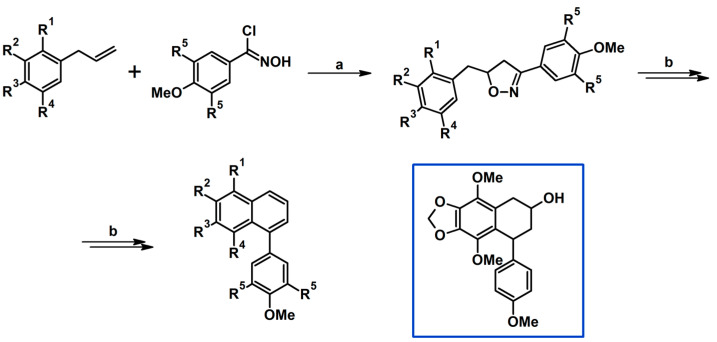
1,3-DP cycloaddition of RCNO to allylbenzene derivatives as a crucial step in the synthetic route leading to the 1-phenylnaphthalene derivatives [126]. R^1^, R^2^, R^3^, R^4^, R^5^ = H, –OCH_2_O–, H, H; MeO, –OCH_2_O–, MeO, H; and others; a = Et_3_N, CH_2_Cl_2_, 10–20 °C, 15 h; up to 79% yield; b = next steps.

**Scheme 119:**
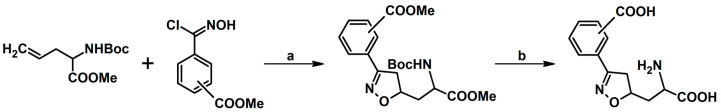
Creation of 3,5-isoxazoline via 1,3-DP cycloaddition as a crucial step in the syntheses of HOOC–L–CH(NH_2_)COOH glutamic acid homologues [127]. a = NaHCO_3_, EtOAc, rt, 72 h, up to 45% yield; b = NaOH, MeOH, rt, 2 h, next CF_3_COOH, CH_2_Cl_2_, 0 °C, 3 h, up to 70% yield.

**Scheme 120:**
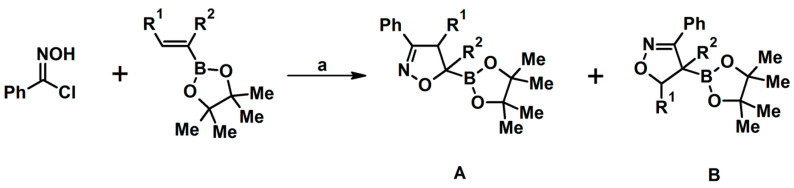
Syntheses of isoxazolines bearing boronic ester functional group [128]. R^1^, R^2^, A:B = H, H, 100:0; H, Me, 100:0; *n*-Bu, H, 0:100; CO_2_Me, H, 0:100; a = Et_3_N, THF, rt, 3 h; 85–97% yield. (**A**,**B**) = mixture of products.

**Scheme 121:**
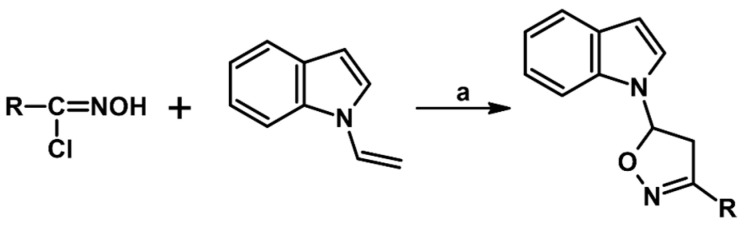
1,3-DP cycloaddition of nitrile oxide to *N*-vinylindole [129]. R = *p*-ClC_6_H_4_, COOEt; a = NaHCO_3_, PhMe, rt, overnight, up to 72% yield.

**Scheme 122:**
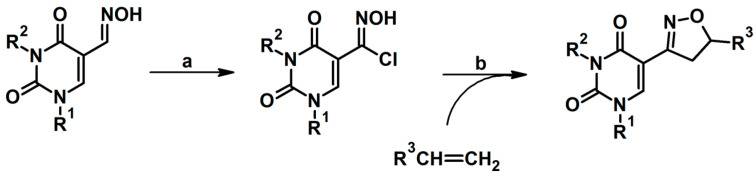
Syntheses of 2-isoxazolines possessing uracyl motif in position 3 [130]. R^1^, R^2^, R^3^ = Me, Me, CN; CH_2_CH_2_COOMe, H, CN; Me, Me, OEt; and others; a = NCS, CHCl_3_, rt, 2 h; b = NEt_3_, CHCl_3_, rt, 24 h; up to 65% yield.

**Scheme 123:**
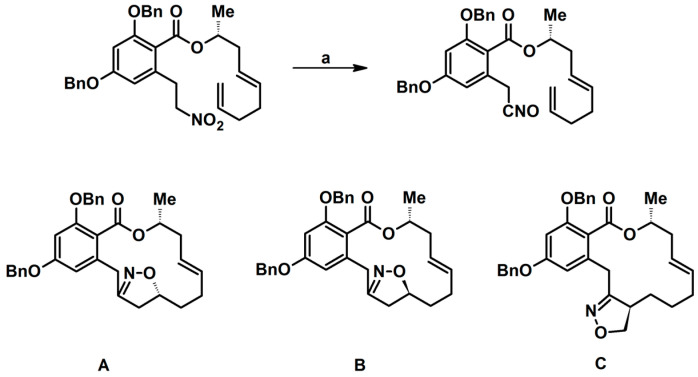
Syntheses of 3,5-disubstituted isoxazolines using INOC strategy [131]. a = *p*-ClC_6_H_4_CNO, benzene, Et_3_N, rfx, 41 h; 90% yield; (**A**/**B**/**C**) = 4/4/1.

**Scheme 124:**
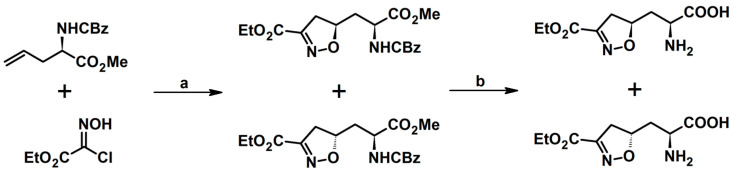
Synthesis of diastereoisomeric DAP analogues possessing isoxazoline motif [132]. a = NaHCO_3_, AcOEt, up to 42% yield; b = deprotection, up to 43% yield.

**Scheme 125:**
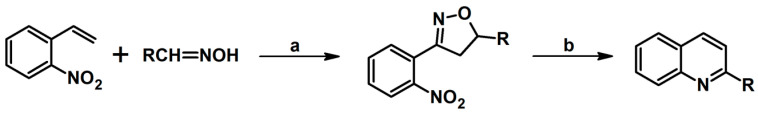
1,3-DP cycloaddition leading to isoxazolines as an initial step in the syntheses of 2-arylquinolines [133]. R = Ph, *p*-BrC_6_H_4_, 2-thienyl, 3-pyridyl, 2,4,6-F_3_C_6_H_2_, 2-Cl-5-O_2_NC_6_H_3_, and others; a = NCS, Et_3_N, DMF, up to 90% yield; b = method 1: Fe, EtOH, NH_4_Cl in H_2_O, 80 °C, 6 h; method 2: Na_2_S_2_O_4_, DMSO, 100 °C, 3–5 h, up to 82% yield.

**Scheme 126:**
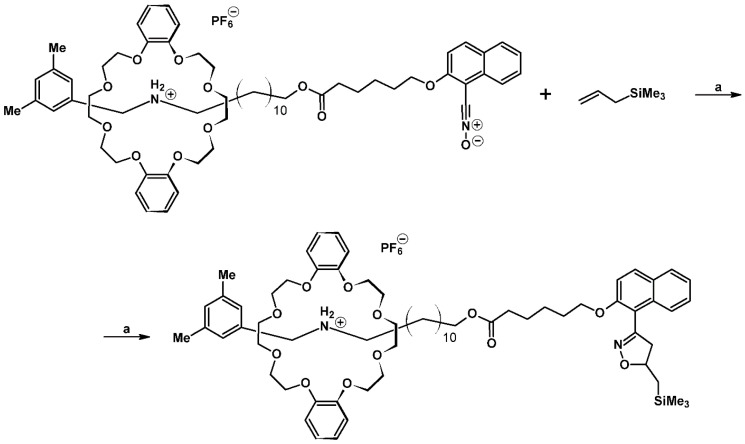
Syntheses of 3,5-disubstituted isoxazolines equipped with catenane motif—an example [134]. a = CHCl_3_, 50 °C, 24 h, yield not given.

**Scheme 127:**
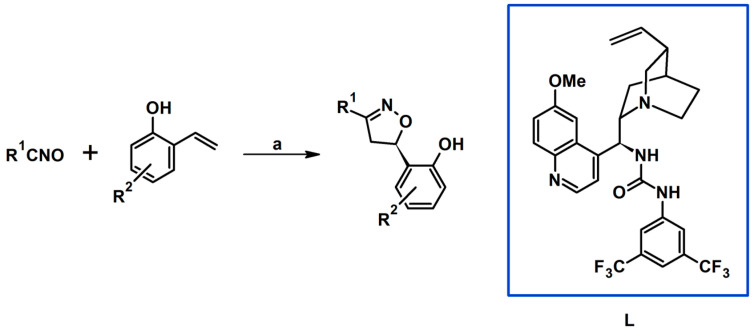
Syntheses of chiral 3,5-disubstituted 2-isoxazolines via 1,3-DP cycloaddition of RCNO to *o*-hydroxystyrenes mediated by the chiral amine-urea bifunctional catalyst [135]. R^1^, R^2^ = 2,4,6-(Me)_3_C_6_H_2_, H; 2,4,6-(MeO)_3_C_6_H_2_, H; 2,6-Cl_2_C_6_H_3_, H; 2,4,6-(Me)_3_C_6_H_2_, 3-Br; 2,4,6-(Me)_3_C_6_H_2_, 3-MeO; *t*-Bu, H; Cy, 5-F and others; L = cinchona-alkaloid-based chiral ligand; up to 97% yield; *ee* = up to 96%; a = (when R^1^ = aryl) catalyst (up to 100 mol%), PhMe, up to rt; (when R^1^ = alkyl or cycloalkyl or Ph) catalyst (30 mol%), oxymoyl chloride as R^1^CNO precursor, Cs_2_CO_3_ or NEt_3_, PhMe, up to 0 °C.

**Scheme 128:**
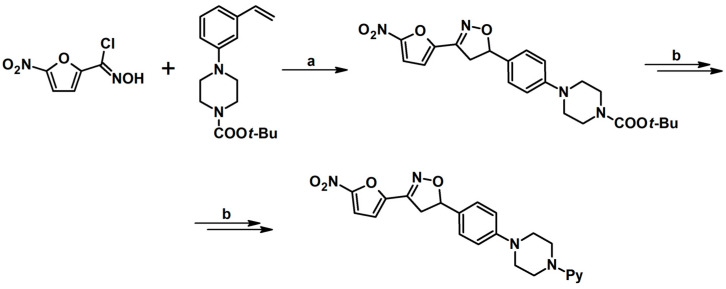
1,3-DP cycloaddition as a crucial step in the syntheses of nitrofuran–isoxazoline–pyridine hybrids [136]. Py = 2-, 3-, or 4-pyridyl, 4-F-3-pyridyl, 4-MeO-3-pyridyl; a = Et_3_N, CHCl_3_, rt, 20 h, up to 98% yield; b = next steps.

**Scheme 129:**
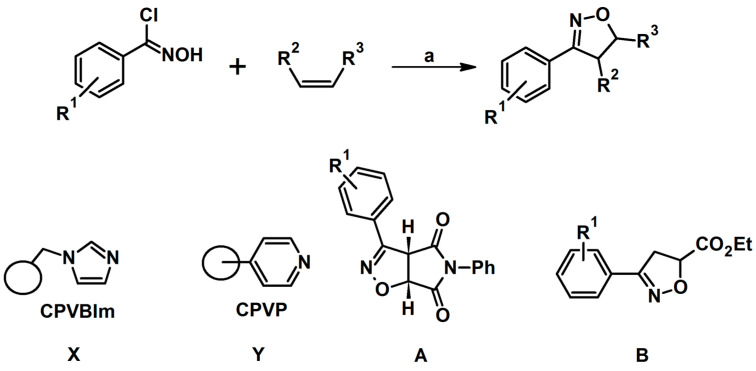
Syntheses of 3,5-di- and 3,4,5-trisubstituted 2-isoxazolines: polymeric bases as catalysts for ArCNO generation from ArC(Cl)=NOH [137]. R^1^ = H, o-Cl, *p*-Cl, *p*-O_2_N, *m*-O_2_N, *m*-Br, *p*-Me; R^2^ = H; R^3^ = COOEt; up to 85% yield (both for A and B); a = MeCN, rt, 1 h (for (**A**)) or 6 h (for (**B**)), cross-linked poly-1-(4-vinylbenzyl)imidazole (**X**) or cross-linked poly-4-vinylpyridine (**Y**).

**Scheme 130:**
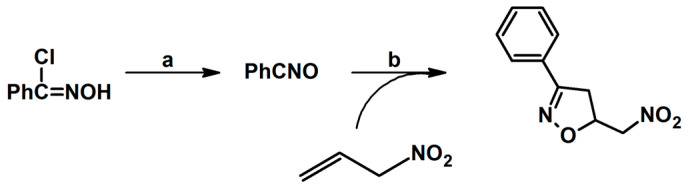
Synthesis of 5-(nitromethyl)-3-phenyl-isoxazoline [138]. a = Et_2_O, from 0 °C to rt, Et_3_N; b = Et_2_O, 25 °C, 20 h; 80% yield.

**Scheme 131:**
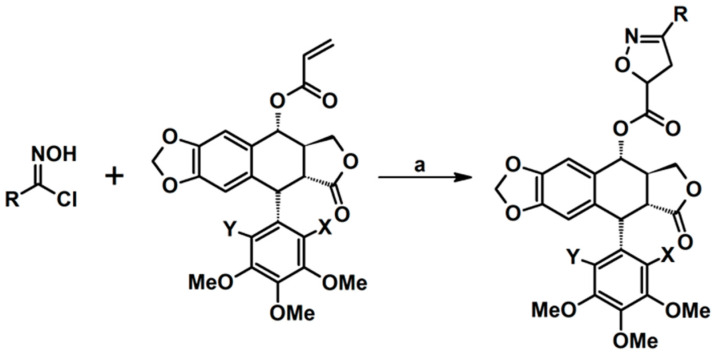
1,3-dipolar cycloaddition as a crucial step in the syntheses of isoxazolines possessing podophyllotoxin motif [139]. R = Ph, *p*-MeC_6_H_4_, *p*-ClC_6_H_4_, *p*-FC_6_H_4_, *p*-O_2_NC_6_H_4_; X, Y = H, H; H, Cl; Cl, Cl; H, Cl; a = Et_3_N, CH_2_Cl_2_, 0 °C, up to 7 h; up to 93% yield.

**Scheme 132:**
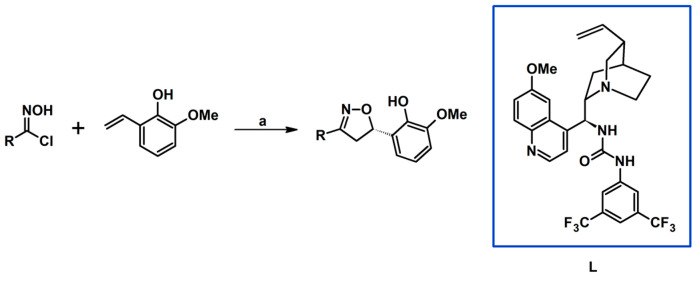
Syntheses of chiral isoxazolines using chiral cinchonine-urea bifunctional catalyst [140]. R = *c*-hexyl, *n*-pentyl, *p*-MeC_6_H_4_, styryl, and others; a = oxymoyl chloride, Et_3_N, PhMe, rt, 30 min, filtration, then dipolarophile and catalyst, −20 °C, 48 h; up to 98% yield; *ee* = 51–90%.

**Scheme 133:**
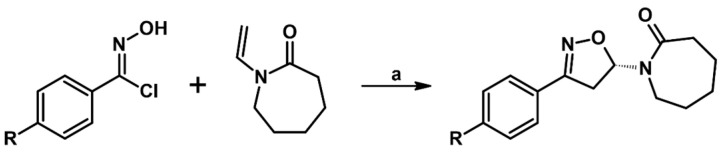
Syntheses of 3,5-disubstituted 2-isoxazolines via regioselective dipolar cycloaddition of nitrile oxide generated in situ from oxymoyl chloride to enamide-type dipolarophile [141]. R = H, Me, F, O_2_N, Br, Cl, MeS, MeO; a = Et_3_N, CH_2_Cl_2_, rt, 24 h; 60–73% yield.

**Scheme 134:**
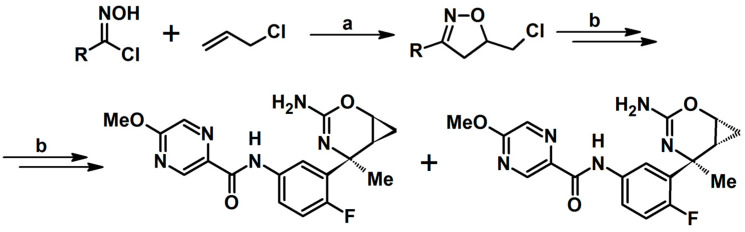
Syntheses of isoxazolines equipped with CH_2_Cl substituent using allyl chloride and selected nitrile oxide as dipolarophile and dipole, respectively [142]. R = Me or 5-Br-2-FC_6_H_3_; a = Et_3_N, Et_2_O; b = next steps (an example); 50% yield (for R = Me) and 52% yield (for R = 5-Br-2-FC_6_H_3_).

**Scheme 135:**
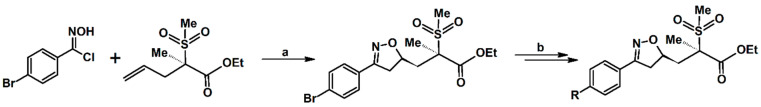
Syntheses of 3,5-disubstituted isoxazolines designed by computational method (virtual screening) [143]. R = Et, cyclopropyl, propyn-1-yl, Ph, 4-phenylpyridin-2-yl, and others; a = Et_3_N, diethyl ether, 0 °C to rt, 3 h; 35% yield (cycloaddition step); b = next steps.

**Scheme 136:**
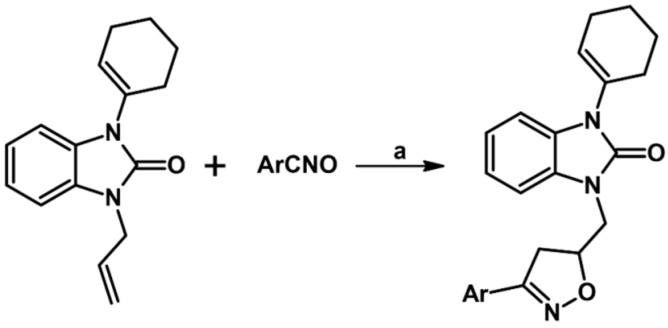
Syntheses of disubstituted isoxazolines possessing a benzimidazolone motif in position 5 [144]. Ar = Ph, *p*-ClC_6_H_4_, *p*-O_2_NC_6_H_4_, Mes, and others; up to 90% yield; a = NaOCl, CHCl_3_, 0–24 °C, 5 h (ArCNO is generated from ArCH=NOH) or Et_2_O, rt, 10 days (only when Ar = Mes).

**Scheme 137:**
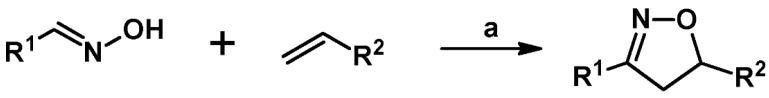
Syntheses of 3,5-disubstituted 2-isoxazolines via 1,3-DP cycloaddition of in situ-generated nitrile oxide to alkenes [145]. R^1^, R^2^ = *i*-Pr, allyl; *i*-Pr, 2-pyridyl; *i*-Pr, CH_2_CH_2_OH; *i*-Pr, CN; Mes, CO_2_*t*-Bu; 2-thienyl, CO_2_*t*-Bu; 2-furyl, CO_2_*t*-Bu; PhC≡C, CO_2_*t*-Bu and others; a = NaCl, oxone, Na_2_CO_3_, MeCN/H_2_O, rt, 12 h, up to 98% yield.

**Scheme 138:**
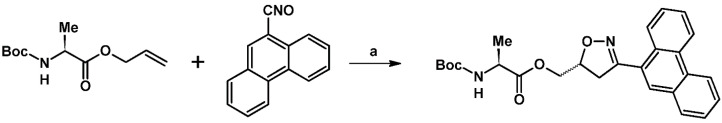
Synthesis of 3,5-disubstituted 2-isoxazolines equipped with N-Boc-protected (S)-alanine and phenanthrene motifs [146]. a = CH_2_Cl_2_, 24 h, rt, 66% yield.

**Scheme 139:**
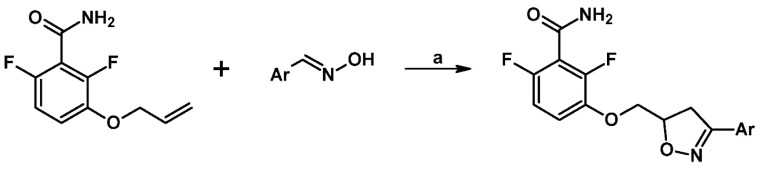
Syntheses of 3,5-disubstituted isoxazolines via dipolar cycloaddition of in situ-generated arylnitrile oxide to allyl-phenyl ether derivative [147]. Ar = Ph; *o*-, *m*-, and *p*-ClC_6_H_4_; *p*-cyclohexylC_6_H_4_; *o*-, *m*-, and *p*-MeOC_6_H_4_; *p*-Me_2_NC_6_H_4_; 2-furyl and others (21 examples); a = chloramine-T, EtOH, rt, up to 74% yield.

**Scheme 140:**
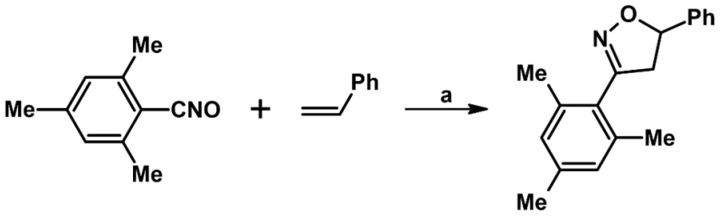
2-Isoxazolines syntheses under supercritical CO_2_—an example [149]. a = scCO_2_, 2600–2700 psi, 70 °C, 24 h; 96% yield.

**Scheme 141:**
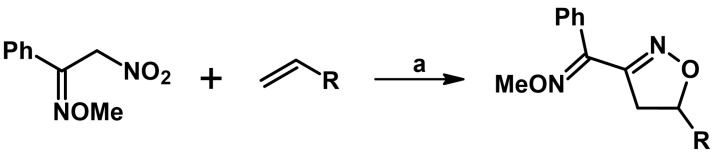
Nitrocompounds as sources of nitrile oxide applied in the synthetic route leading to isoxazolines [150]. R = CN, CO_2_Me, *n*-hexyl, *p*-ClC_6_H_4_; a = PhNCO, Et_3_N, THF, rt, 24 h; up to 98% yield.

**Scheme 142:**
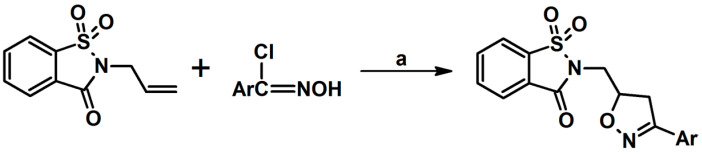
1,3-DP cycloaddition engaged in the syntheses of saccharin–(2-isoxazoline) hybrids [151]. a = Et_3_N, AcOEt; Ar = Ph, *m*- or *p*-O_2_NC_6_H_4_; *m*- or *p*-MeOC_6_H_4_; 3- or 4-pyridyl; 2-thienyl and others; up to 97% yield.

**Scheme 143:**
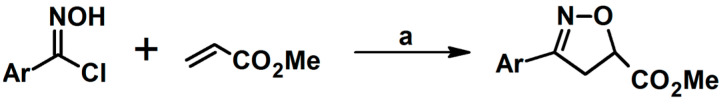
Syntheses of 3,5-disubstituted 2-isoxazolines from benzonitrile oxide derivatives and methyl acrylate [152]. Ar = Ph, 3,4-(methylenedioxy)C_6_H_3_, *p*-MeOC_6_H_4_, *m*-ClC_6_H_4_; a = pyridine, CH_2_Cl_2_, 25 °C, 24 h; up to 80% yield.

**Scheme 144:**
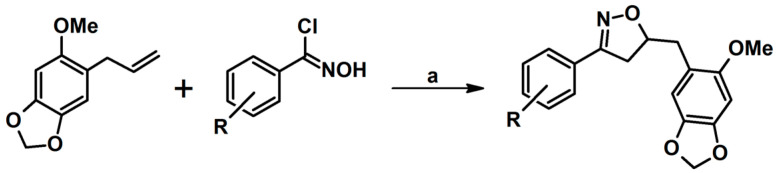
Syntheses of sarisan possessing 3,5-disubstituted 2-isoxazoline moieties via 1,3-DP cycloaddition of in situ-generated benzonitrile oxide derivatives to 1-allyl-3,4-methylenedioxy-5-methoxybenzene [153]. R = H, *o*-Cl, *o*-MeO, *m*-F, *p*-O_2_N, *p*-F_3_C, *m*-Cl, *o*-Br, *m*-Br, *p*-Br, 2-F-4-Cl, *m*-F, *o*-F, and others; a = Et_3_N, CH_2_Cl_2_, rt, 6–24 h; up to 81% yield.

**Scheme 145:**
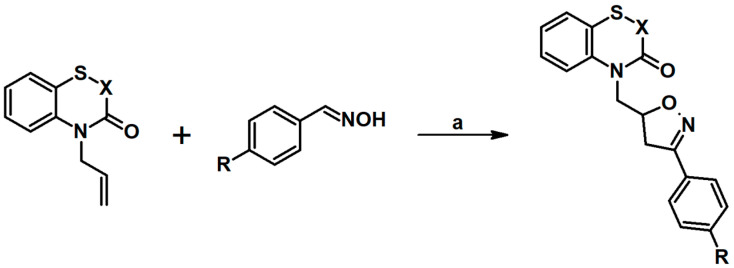
Synthesis of isoxazoline–CH_2_–benzothiazinone hybrids via 1,3-DP cycloaddition [154]. X, R = CH_2_, H; CH_2_, Cl; CH_2_, NMe_2_; CH_2_, Br; CH=CHC_6_H_4_, H; CH=CHC_6_H_4_, Cl; CH=CHC_6_H_4_, Br; CH=CHC_6_H_4_, NMe_2_; a = NaOCl, H_2_O, CHCl_3_, 0 °C; up to 75% yield.

**Scheme 146:**
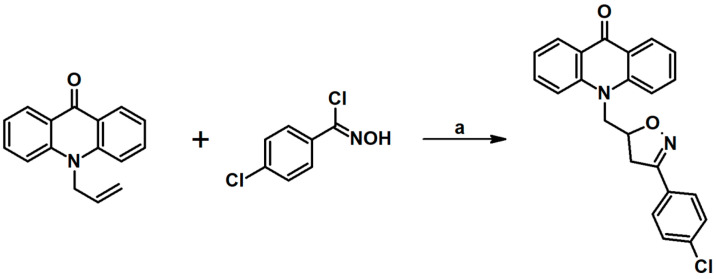
Synthesis of acridone-CH_2_-isoxazoline hybrids via 1,3-DP cycloaddition [155]. a = Et_3_N, CHCl_3_, 40–70 °C, 25 min, MW; 77% yield.

**Scheme 147:**
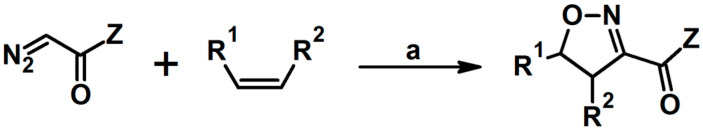
Syntheses of 3,5-di- and 3,4,5-trisubstituted isoxazolines via 1,3-DP cycloaddition of in situ-generated RCNO to carbon–carbon double bond [156]. Z = OEt or NEt_2_ or Ph (for all alkene substrates); R^1^, R^2^ = H, COOMe; H, *n*-hexyl; *N*-phthaloimide; a = *t*-BuONO, CHCl_3_, rt, 2–5 h; up to 96% yield.

**Scheme 148:**
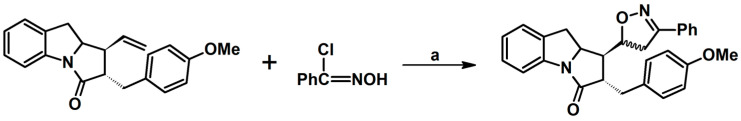
Synthesis of chiral 3,5-disubstituted 2-isoxazolines involving 1,3-DP cycloaddition of in situ-generated PhCNO and a chiral dipolarophile [157]. Dipolarophile *ee* ≥ 99%; a = Et_3_N, CH_2_Cl_2_, 12 h, rt; 75% yield; *dr* = 1,5/1; *ee* ≥ 99%.

**Scheme 149:**
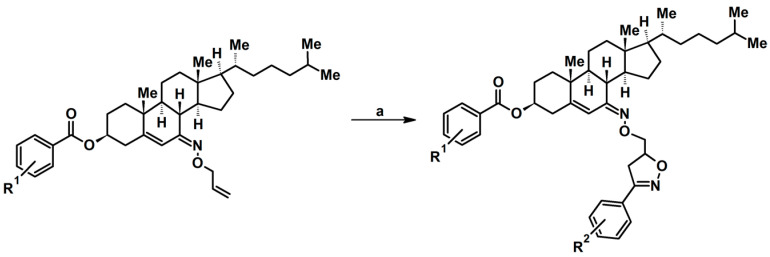
1,3-DP cycloaddition engaged in the syntheses of cholesterol–isoxazoline hybrids [158]. R^1^ = H, *p*-Me, *p*-F, *p*-Cl, *o*-Br; R^2^ = H, *p*-Me, *p*-Cl; 2,4-di-Cl, *p*-O_2_N, and others; a = R^2^C(Cl)=NOH, Et_3_N, CH_2_Cl_2_, rt, up to 120 h; up to 59% yield.

**Scheme 150:**
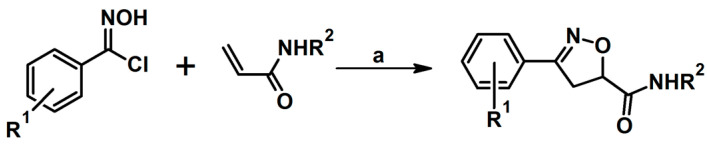
Syntheses of 3,5-disubstituted 2-isoxazolines possessing aryl substituent in position 3 and *N*-substituted aminoacyl group in position 5 [159]. R^1^, (R^2^) = *p*-Me, *o*-Cl, *m*-Br, *p*-Cl, *p*-Fand others, (cyclohexyl); Ph, *p*-F, (*n*-octyl); Ph, 4-FPh, Ph, *p*-F, *p*-Cl, *p*-O_2_N, (6-MeO-2-aminobenzothiazol-1-yl), and others (more than 30 examples); a = Et_3_N, CH_2_Cl_2_, up to 15 h, rt; up to 92% yield.

**Scheme 151:**
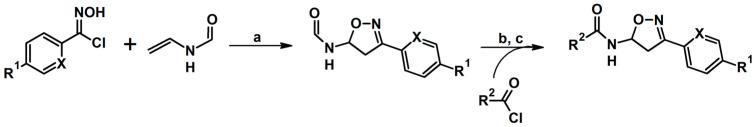
Syntheses of 3,5-disubstituted isoxazolines via in situ-generated RCNO to an amide of formic acid followed by the one-pot cycloadduct acylation [160]. X, R^1^, R^2^ = CH, cyclohexyl, CF_3_; N,3,5-dimethoxyphenyl, Cl; CH, CF_3_, Bz; N, Cl, 4-FBz; N, Cl, 5-Me-isoxazol-3-yl; and others; a = Et_3_N, MTBE, rt, 16 h; b = HCl, MeOH; c = Et_3_N, CH_2_Cl_2_, 25 °C, 1 h; up to 89% yield.

**Scheme 152:**
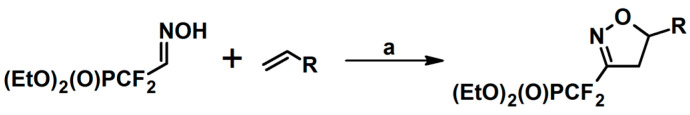
A DIB-promoted one-pot synthetic method providing access to 3,5-disubstituted isoxazolines bearing a difluoromethyl phosphonate group [161]. R = Bn, CO_2_Et, CH_2_Br, Ph, CH_2_NHBoc, C_6_F_5_, and others; a = DIB, CH_2_Cl_2_, from 0 °C to rt; up to 96% yield.

**Scheme 153:**
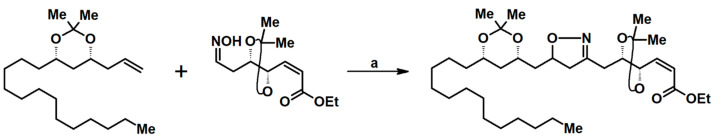
Crucial step in total synthesis of (*+*)-EBC-23 (a potent anticancer agent from the Australian rainforest): obtaining isoxazoline from oxime [162]. a = OXONE, KCl, H_2_O, 23 °C, 4 h; 45% yield.

**Scheme 154:**
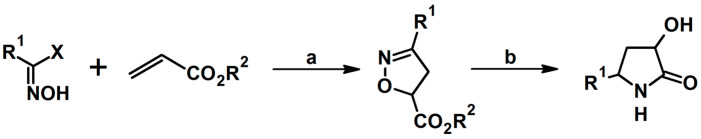
3,5-disubstituted 2-isoxazolines as intermediates in the synthetic route leading to the α-hydroxy lactams [163]. R^1^, R^2^ = CO_2_Et, Me; Ph, Me; Et, Et; *t*-Bu, Me; CHF_2_, Et and others (25 examples); a (X = Cl) = NaHCO_3_, AcOEt, rt, overnight; a (X = H) = 1. NCS, TMSCl (cat.), rt, overnight, 2. NaHCO_3_, overnight, rt; b = H_2_ (1 atm), Pd-C or Pd(OH)_2_-C or Pt-C, MeOH or EtOH, rt, up to 72 h; up to 97% yield (isoxazolines)’ up to 89% yield (3-hydroxypyrrolidin-2-ones).

**Scheme 155:**
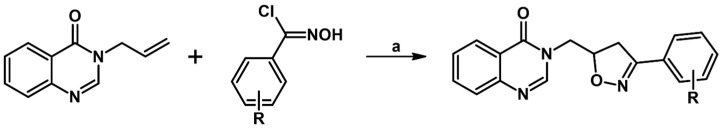
Syntheses of quinazolinone–isoxazoline hybrids via 1,3-DP cycloaddition of RC_6_H_4_CNO to *N*-allyl quinazolinones [164]. R = H, *o*-Cl, *p*-Cl, *p*-Br, *p*-MeO, *p*-Me, *p*-O_2_N; a = CHCl_3_, Et_3_N, rt; up to 96% yield.

**Scheme 156:**
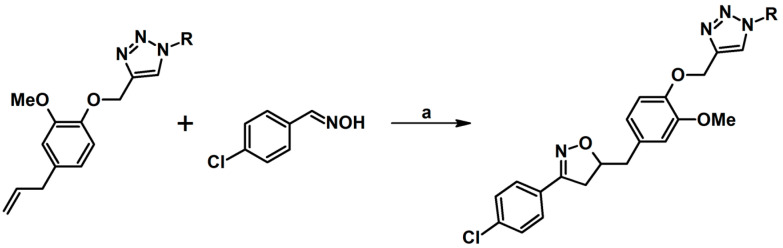
Syntheses of multicomponent hybrids containing 3,5-disubstituted isoxazoline’s moiety [165,166]. R = Bn, EtOOCCH_2_, n-C_8_H_17_, Ph, *p*-C_6_H_4_, *p*-MeOC_6_H_4_, *p*-O_2_NC_6_H_4_; a = NaOCl, CHCl_3_, 6 h, rt; up to 72% yield.

**Scheme 157:**
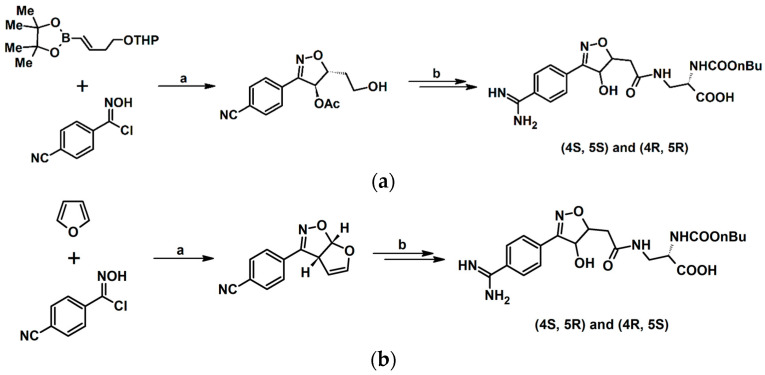
1,3-DP cycloaddition of *p*-NCC_6_H_4_CNO to dipolarophile of the R^1^CH=CHR^2^ type as a crucial step in the syntheses of *cis* and *trans* isomers of an isoxazoline-ring-hydroxylated metabolite of Roxifiban [167]. (**a**) a = Na_2_CO_3_‚ H_2_O_2_, next acetic anhydride, pyridine, next acetic acid, H_2_O; b = next steps; 95% yield; (**b**) a = Et_3_N, excess of furan as solvent; b = next steps; 23% yield.

**Scheme 158:**
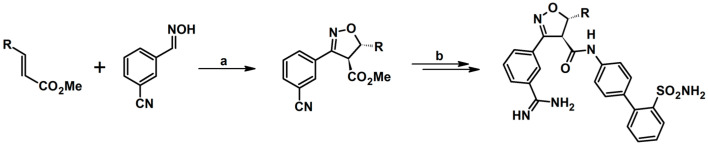
Syntheses of trisubstituted isoxazolines followed by structural modification [168]. R = H, MeOCH_2_, tetrazole-CH_2_; a = NCS; b = next steps**;** moderated yield.

**Scheme 159:**
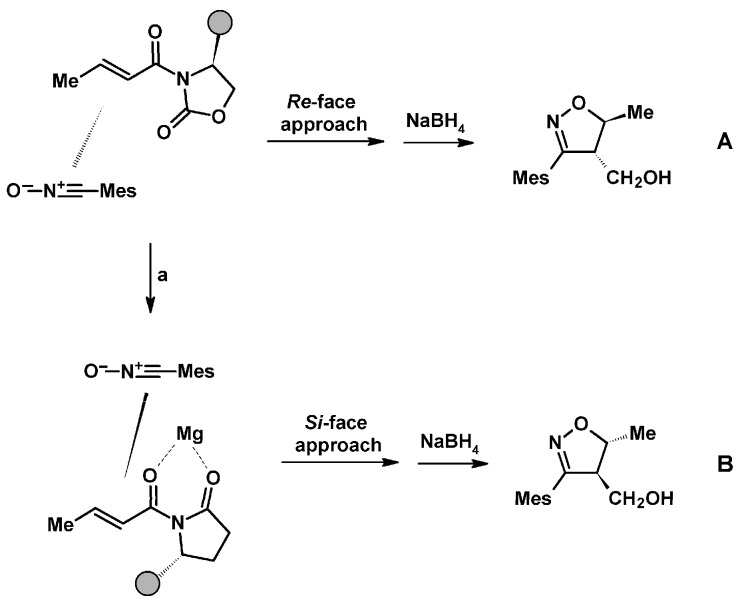
Syntheses of chiral 3,4,5-trisubstituted isoxazolines: the role of auxiliaries and complexation with Mg^2+^ (an example) [169]. a = Mg(ClO_4_)_2_, MeCN, CH_2_Cl_2_, rt, 7 days; up to quantitative yield; *ee* = up to 86% (**A**) and up to 23% (**B**).

**Scheme 160:**
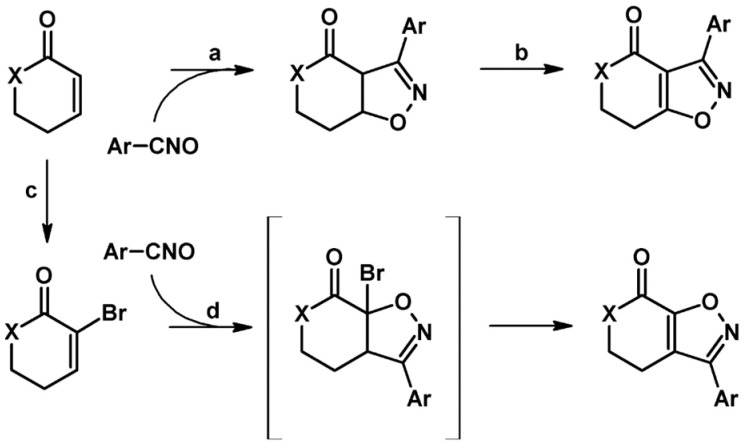
3,4,5-trisubstituted isoxazolines as precursors for obtaining isomeric isoxazoles (selected examples) [170]. X = O or CH_2_; Ar = 2,6-Cl_2_C_6_H_3_; a = THF, up to 64%; b = NiO_2_ or γ-MnO_2_, up to 71%; c = bromine, up to 75%; d = Et_3_N, up to 54% yield.

**Scheme 161:**
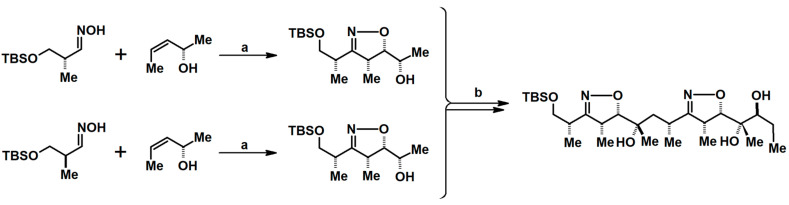
Regio- and enantioselective syntheses of 3,4,5-trisubstituted isoxazolines as a crucial step in the synthetic strategy leading to polyketide building block [171]. a = *t*-BuOCl, −78 °C, dipolarophile, then *i*-PrOH, EtMgBr, CH_2_Cl_2_, 12 h, rt; b = next steps.

**Scheme 162:**
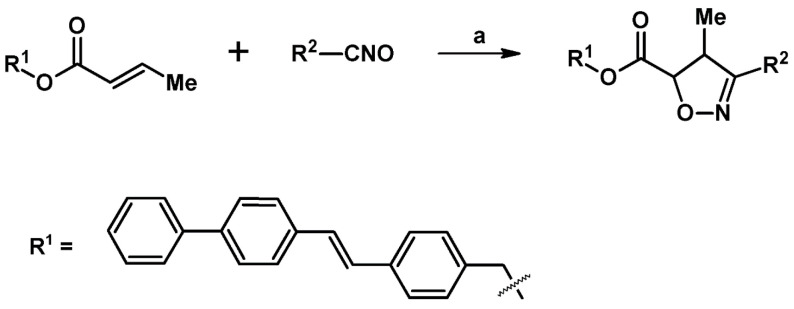
Syntheses of isoxazolines equipped with the (*E*)-stilbene motif [16]. R^2^ = *t*-Bu, Ph; a = pyridine, CH_2_Cl_2_, 0 °C; 75% (for R^2^ = *t*-Bu) or 74% (for R^2^ = Ph) yield.

**Scheme 163:**
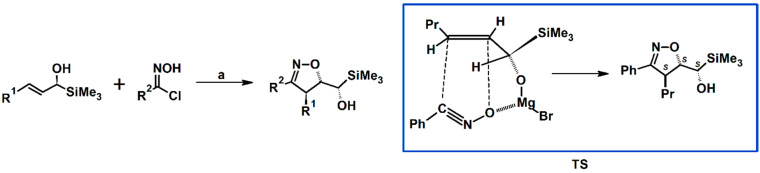
*Mg*-mediated highly enantioselective 1,3-DP cycloaddition leading to chiral isoxazolines [172]. TS-structure explaining the reaction stereoselectivity. R^1^, R^2^, % *ee* A, % *ee* B = *n*-C_3_H_7_, Ph, 87, 82; *n*-C_3_H_7_, CH_2_CH_2_C_6_H_4_, 87, 83; Me, Ph, 85, 80; and others; a = BuOMgBr, CH_2_Cl_2_, next −78 °C to rt, Et_3_N, 17 h; 40–75% yield.

**Scheme 164:**
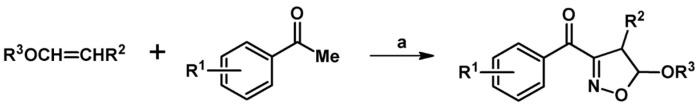
Obtaining the 3,4,5-trisubstituted isoxazolines possessing: benzyloxy moiety in position 3, alkyl substituent in position 4, and alkoxy group attached to the C-5 [173]. R^1^, R^2^, R^3^ = *o*-Me, H, Bu; *p*-F, H, Bu; *o*-Cl, H, Bu; 3,4-Cl_2_, Me, Et and others; a = BuONO, HCl, Et_2_O, rt, 5–16 h; followed by NaHCO_3_, *i*-PrOH, rt, overnight; up to 91% yield.

**Scheme 165:**
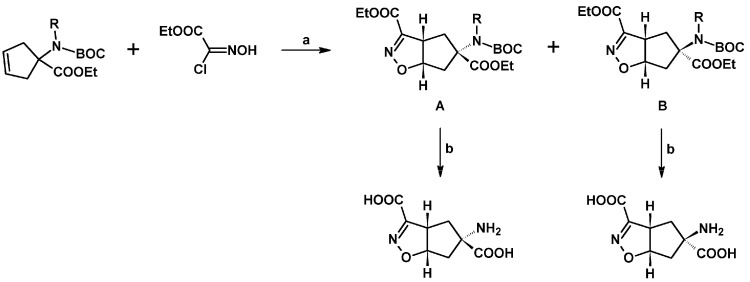
3,4,5-trisubstituted 2-isoxazolines as substrates for obtaining bicyclic acidic amino acids [174]. R = Hor BocH or Boc; a = NaHCO_3_, AcOEt; yield up to 84% for (**A**) and up to 67% for (**B**).

Pharmacological tests proved that both bicyclic acids in Scheme 166 behaved as antagonists at mGluR1,5 and agonists at mGluR2. Furthermore, whereas A was inactive at all ionotropic glutamate receptors, B displayed a potent antagonism at the NMDA receptors. In the in vivo tests on DBA/2 mice, both compounds displayed an anticonvulsant activity. Moreover, the pharmacological profile of B qualifies it as a neuroprotective agent.

Additionally, several papers devoted to the syntheses of isoxazoline-based amino acids were presented in the review from 2012 [175].

Regio- and stereoselective 1,3-DP cycloaddition performed by Mineno and Tiller in 2003 between specially designed dipole and dipolarophile was a crucial step in the total convergent synthesis of the racemic anti-influenza agent BCX-1812 (RWJ-270201)—Scheme 166 [176].

**Scheme 167:**
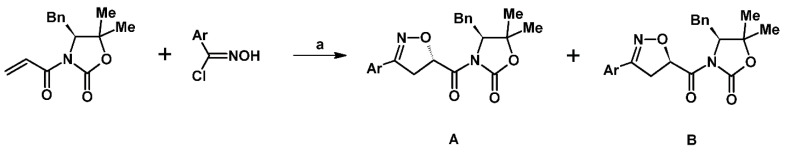
Diastereoselective syntheses of chiral 3,4,5-trisubstituted isoxazolines via Lewis-acid-controlled 1,3-DP cycloaddition of ArCNO to a chiral 3-acryloylamide-type dipolarophile bearing 2-oxazolidinone motif as a chiral auxiliary [177]. Ar, (a, (**A**,**B**)) = Ph (no LA, 43/57 MgBr_2_ in MeCN, 99/1 Yb(OTf)_3_ in CH_2_Cl_2_, 96/4); *p*-MeOC_6_H_4_ (no LA, 52/48; MgBr_2_ in MeCN, 97/3; Yb(OTf)_3_ in CH_2_Cl_2_, 95/5); *p*-ClC_6_H_4_ (no LA, 43/57; MgBr_2_ in MeCN, 98/2; Yb(OTf)_3_ in CH_2_Cl_2_, 95/5); and others; up to 85% yield.

The same year, a combination of equimolar amounts of Lewis acids, especially MgBr_2_ or Yt(OTf)_3_, and a dipolarophile equipped with a chiral oxazolidinone motif allowed Yamamoto and colleagues to perform 1,3-DP cycloaddition in a highly diastereoselective manner—Scheme 167 [177].

**Scheme 168:**
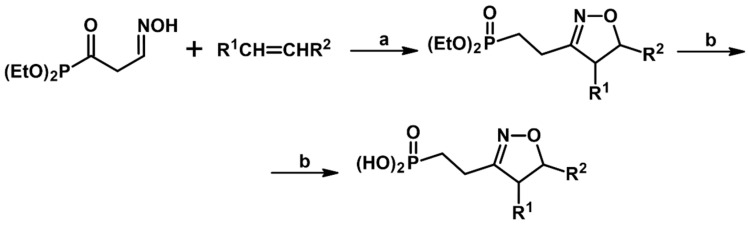
One-pot 1,3-DP cycloaddition procedure for the syntheses of di- and trisubstituted isoxazolines followed by hydrolysis to isoxazoles [178]. R^1^, R^2^ = H, COOMe; H, CH_2_OH; Me, COOMe; Me, COOH; succinimide motif; and others; a = NCS, CHCl_3_, next dipolarophile and Et_3_N; 63–86% yield; b = Me_3_SiBr, CH_2_Cl_2_, 0 °C, next H_2_O, rt.

In 2004, 3,5-di- and 3,4,5-trisubstituted isoxazolines bearing a (EtO)_2_P(O) moiety tethered to a heterocyclic scaffold via a –CH_2_CH_2_– linker were obtained in the 1,3-DP cycloaddition reactions—Scheme 168 [178].

All obtained isoxazolines were transformed into appropriate isoxazoles—see the scheme presented above. Interestingly, some isoxazoles and isoxazolines enhanced indole alkaloids’ accumulation in periwinkle cell cultures.

**Scheme 169:**
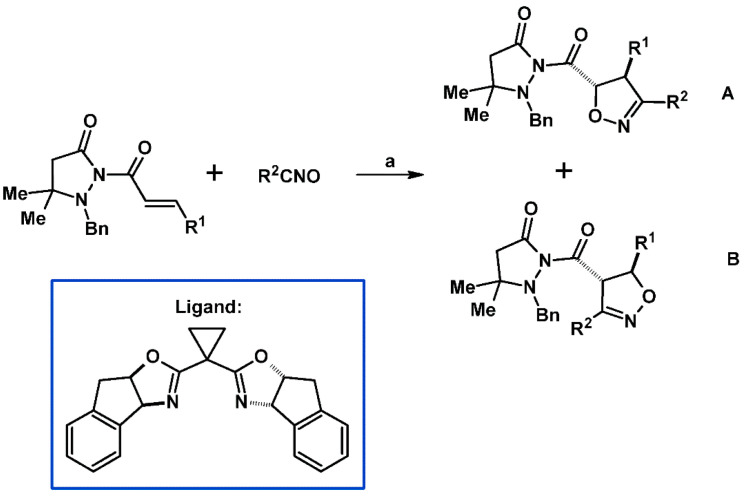
Syntheses of chiral 3,4,5-trisubstituted isoxazolines via cycloaddition of aryl nitrile oxide to enamide-type dipolarophiles [179]. Enantioselectivity was reached by *Mg*-complex with chiral bis-isoxazoline-type ligands. R^1^, R^2^ = Me, 2,4,6-Me_3_C_6_H_2_; Ph, 2,4,6-Me_3_C_6_H_2_; Me, Ph; Me, *p*-MeOC_6_H_4_; and others; a = 30 mol% (MgI_2_ + L), CH_2_Cl_2_, rt, MS 4Å; up to 86%; (**A**/**B**) = 99/1 (except for two examples) yield; *ee* = 99% (except for three examples).

The same year, Sibi, Itoh, and Jasperse reported the synthesis of chiral 3,4,5-trisubstituted isoxazolines using dipolar cycloaddition of aryl (Ar = substituted phenyl) or *t*-butyl nitrile oxides to enamide-type dipolarophiles—Scheme 169 [179].

Various chiral ligands, Mg, Ni, Cu, and Zn compounds, for instance, MgI_2_, Zn(OTf)_2_, Ni(ClO_4_)_2_, and various templates in nitrile oxide, were tested. It was confirmed that all tested parameters, i.e., the kind of Lewis acid, the bulkiness of the template, and the chiral ligand structure, are decisive as far as the regio- and enantioselectivity are concerned.

**Scheme 170:**
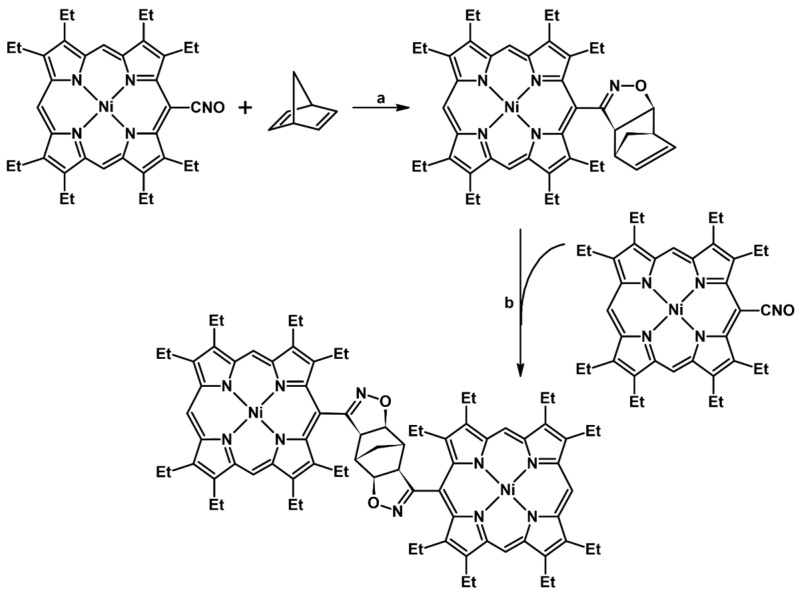
Syntheses of isoxazolines possessing one or two *Zn*-porphyrin moieties [180]. a = 40 °C, 2–3 h, 97% yield; b = 70 °C, 72 h, 92% yield.

3,4,5-trisubstituted 2-isoxazolines equipped with one or two *Zn*-porphyrin moieties were obtained via 1,3-DP cycloaddition of (*Zn*-porphyrin)CNO to norbornadiene by Morozova and colleagues—Scheme 170 [180].

**Scheme 171:**
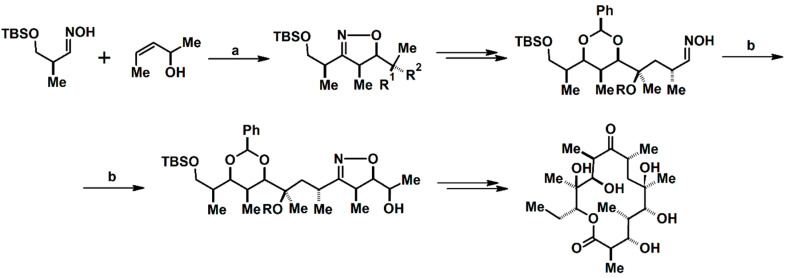
1,3-DP cycloaddition engaged in the stereoselective total synthesis of erythronolide A [181]. a = *t*-BuOCl, CH_2_Cl_2_, −78 °C, then *i*-PrOH, EtMgBr, 0 °C to rt, 86% yield, *dr* ≥ 95:5; b = *t*-BuOCl, CH_2_Cl_2_, −78 °C, then *i*-PrOH, EtMgBr, 0 °C to rt, 86% yield, *dr* ≥ 99:1.

As reported by Muri, Lohse-Fraefel, and Carreira in their work from 2005, cycloaddition mediated by Mg 1,3-DP was twice engaged in the 21-linear-step synthetic route leading to erythrinolide A—Scheme 171 [181].

Notably, the isoxazoline (3,4,5-trisubstituted) is a robust protecting group for ß-hydroxyketones that can be cleaved at a late stage of the synthetic method. Moreover, isoxazoline proved to be suitable for the delicate macrolactonization of erythronolide A seco acid (one of the last intermediates).

**Scheme 172:**
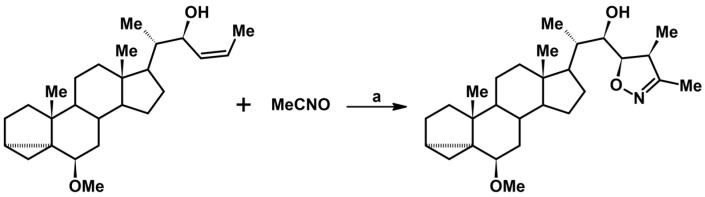
Synthesis of (4′*R*,5′*R*,22*R*)-22-Hydroxy-22-(3′,4′-dimethylisoxazolin-5′-yl)-6b-methoxy-3a,5-cyclo-23,24-dinorcholane [182]. a = THF, rt, 4 h, yield not given.

3,4,5-trisubstituted 2-isoxazoline possessing a steroid motif was the only diastereomer formed in the 1,3-dipolar cycloaddition of acetonitrile oxide to steroid-type dipolarophile—Scheme 172 [182]. The product described by Litvinovskaya et al. has the 4′R,5′R stereochemistry in the newly formed chiral centers.

**Scheme 173:**
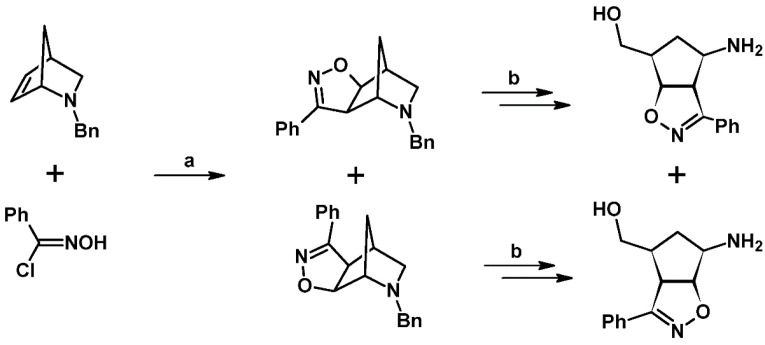
1,3-DP cycloaddition as a starting step in the synthetic procedure leading to the specially designed aminols (an example) [183]. a = Et_3_N, CH_2_Cl_2_, rt, 48 h up to 49% yield; b = next steps.

In 2006, isoxazoline-fused carbocyclic aminols, valid for the synthesis of isoxazoline-carbocyclic nucleosides, were obtained starting from 1,3-DP cycloaddition of in situ-generated PhCNO to 2-azanorbornenes—Scheme 173 [183].

Analogous reactions and (similar) bicyclo-isoxazolines as those shown in the scheme above were the subject of follow-up work by Quadrelli et al. [184]. Moreover 1,3-DP cycloaddition of bromonitrile oxide to 2-azanorbornene was used in the synthesis and molecular modelling of dihydroxycyclopentane-carbonitrile nor-nucleosides [185].

**Scheme 174:**
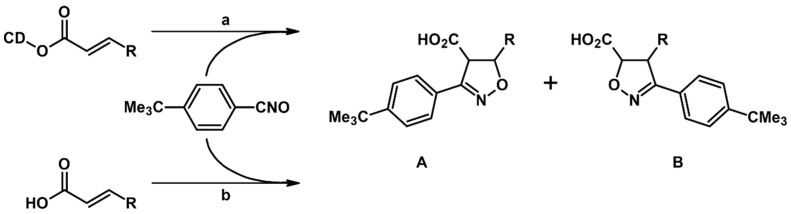
Usage of dipolarophile attached to cyclodextrin: spectacular influence on DP cycloaddition’s regioselectivity (selected examples) [186]. a = H_2_O, 15 h next hydrolysis; b = THF or MeOH/H_2_O next hydrolysis. R = H, (**A**/**B**) = 20/1 (CD-Dip) and 1/20 (Free-Dip); R = Me, (**A**/**B**) = 100/1 (CD-Dip) or 1/1 (Free-Dip); CD-Dip = dipolarophile tethered to cyclodextrin; Free-Dip = free dipolarophfile.

Applying dipolarophile in the form of 6^A^-deoxy-6^A^-propynamido-β-cyclodextrin esters resulted in reversing the regioselectivity of RCNO cycloadditions and increasing the reaction rate up to 475 times—Scheme 174 [186]. Notably, as reported by Barr, Lincoln, and Easton, the products were readily released from the cyclodextrin through ester hydrolysis, so the cyclodextrin could, in principle, be recycled.

In 2006, cyclodextrin was also used in the 1,3-dipolar cycloaddition of RCNO to adamantylidenefulvene but without spectacular influence on the regioselectivity [187].

**Scheme 175:**
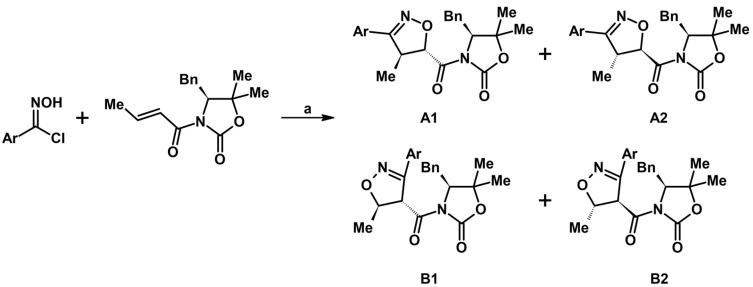
Syntheses of chiral 3,4,5-trisubstituted 2-isoxazolines using chiral auxiliary and Mg(ClO_4_)_2_ as a catalyst [188]. Ar, (**A1**/**A2**), B1/B2 = Ph, 66/34^x^ or 82/2^y^, 41/59 or 95/5, none detected; *p*-MeOC_6_H_4_, 63/37 or 96/4, 40/60 or 94/6, 56/44 or 94/6 and others; ^x^ = without Mg; ^y^ = 1 eq. Mg; up to 88% overall yield; a = Et_3_N, MeCN, MgClO_4_, 0 °C. All regioisomers (**A1**,**A2**,**B1**,**B2**) were observed in all cases.

Hayashi’s team observed regio- and stereoselective 1,3-DP cycloaddition in the reaction between crotonamide equipped with the chiral auxiliary and in situ-generated ArCNO—Scheme 175 [188]. Adding Mg(ClO_4_)_2_ to the reaction strongly influenced the regio- and diastereoselectivity of isoxazoline formation.

**Scheme 176:**
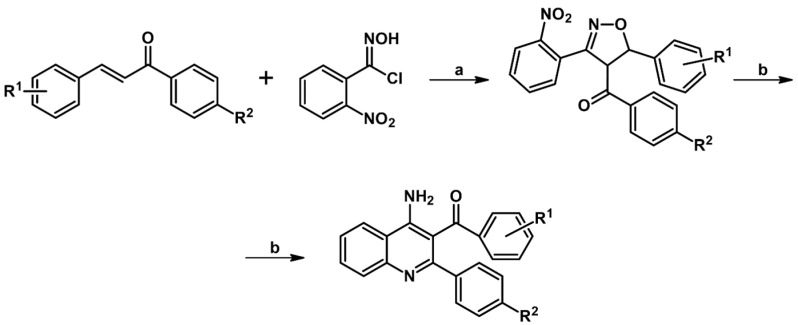
Syntheses of 3,4,5-trisubstituted 2-isoxazolines in the 1,3-DP cycloaddition between 2-nitrobenzonitrile oxide and aryl-styryl ketones—a crucial step in the synthetic route leading to the quinoline derivatives [189]. R^1^ = *p*-Cl, *p*-Br, 2,3-Cl_2_, 2,4-Cl_2_, 3,4-Cl_2_; R^2^ = H or Me. a = Et_3_N, Et_2_O, −78 °C to rt, 14–16 h; up to 64% isooxazoline yield; b = Fe, AcOH, rfx, 110 °C, 30 min.

In 2007, Madapa and colleagues reported the creation of 3,4,5-trisubstituted isoxazolines on the way to substituted isoxazolo [4,3-c]quinolines—Scheme 176 [189].

**Scheme 177:**
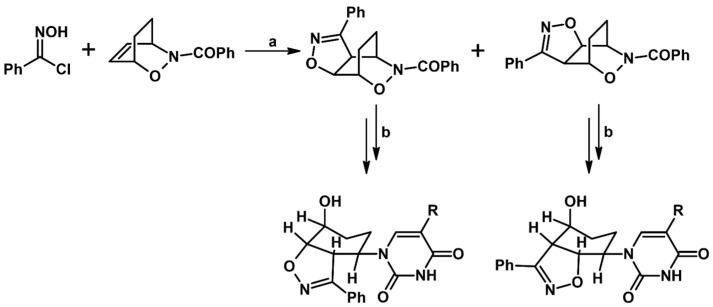
1,3-DP cycloaddition as a crucial step in the synthetic route leading to the synthons for the linear construction of pyrimidine nucleosides [53]. a = Et_3_N, CH_2_Cl_2_, rt, 48 h, 22% and 20%; b = next steps.

Another report came from Quadrelli’s team in 2008. Namely, it concerned 1,3-DP cycloaddition of PhCNO to *N*-benzoyl-2-oxa-3-azabicyclo [2.2.2]oct-5-ene as an initial step of a synthetic procedure leading to the *syn* and *anti* isoxazoline-fused-oxa-azabicyclo octane—Scheme 177 [53]. Unfortunately, only the anti-cycloadducts were reactive and could be transformed into stereodefined isoxazoline-carbocyclic aminols. The two regioisomers, *syn*-1,3-DP cycloaddition products, were completely unreactive.

**Scheme 178:**
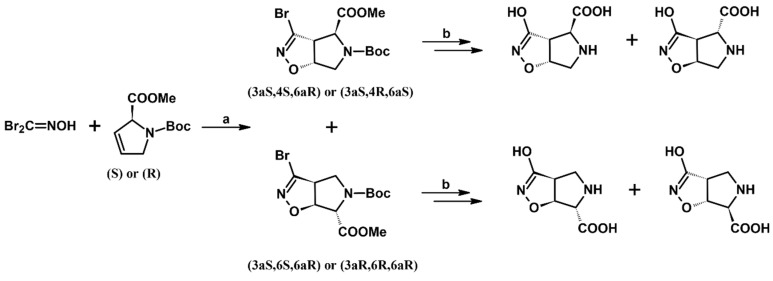
1,3-DP cycloaddition of in situ-generated BrCNO to (*S*)- and (*R*)-*N*-Boc-3,4-didehydroproline methyl ester as an essential step in the syntheses of enantiomerically pure inhibitors of excitatory amino acid transporters [190]. a = NaHCO_3_, AcOEt; 25% and 27% yield, respectively; b = next steps.

1,3-DP cycloaddition was involved in the 2008 synthesis of the two couples of regioisomers (+)-(3*aS*,4*R*,6*aS*)-/()-(3*aR*,4*S*,6*aR*)-3-hydroxy-3*a*,4,6,6*a*-tetrahydro-pyrrolo [3,4-*d*]isoxazole-4-carboxylic acid [(+)-HIP-A and (−)-HIP-A] and (+)-(3*aS*,6*S*,6*aS*)-/()-(3*aR*,6*R*,6*aR*)-3-hydroxy-3*a*,4,6,6*a*-tetrahydro-pyrrolo [3,4-*d*]isoxazole-6-carboxylic acid [(+)-HIP-B and (−)-HIP-B]—Scheme 178 [190].

Notably, the biological activity evaluation and docking experiments show that the absolute configuration of the stereogenic centers undoubtedly plays a crucial role in the interactions with the target proteins. A similar synthetic strategy to the one shown in the scheme above was used in an earlier paper dedicated to obtaining enantiopure stereoisomeric homologues of glutamic acid [191].

**Scheme 179:**
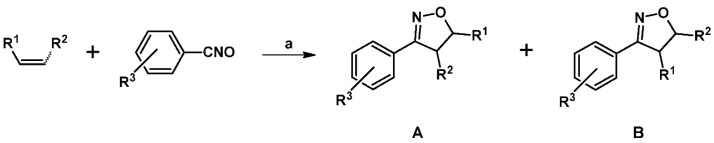
1,3-DP cycloaddition mediated by (*R*)-(+)-BINOL-Yt(OTf)_3_ complex: syntheses of 3,4,5-trisubstituted isoxazolines [192]. R^1^, R^2^, (*E* or *Z*), R^3^ = HOCH_2_, Et (*E*), 4-F_3_C; 68% yield; (**A**/**B**) = 1.4/1; %*ee* = 70 (for A) and 60 (for B); HOCH_2_, Et (*Z*), 4-*i*-Pr; 61% yield; A/B = 1/2,9; % *ee* = 48 (for A) and 71 (for B); Me, COOMe (*E*), 4-F_3_C; 68% yield; (**A**/**B**) = 1.6/1; %*ee* = 25 (**A**) and 89 (**B**); Et, COO*t*-Bu (*E*), 4-F_3_C; 51% yield; %*ee* = 23 (**A**) and 65 (**B**); and others; a correlation between dipolarophile and cycloadduct configuration: *E* gives *trans* and *Z* gives *cis*; a = (*R*)-(+)-BINOL, Y(OTf)_3_; CH_2_Cl_2_, Amberlyst 21 (for ArCNO generation).

In another article, Gołębiewski and Gucma presented chiral, 3,4,5-trisubstituted isoxazolines obtained via regio- and stereoselective 1,3-DP cycloaddition of nitrile oxides to unsaturated alcohols, esters, and amides catalyzed by *R*-(+)-BINOL-Yb-complex—Scheme 179 [192].

According to the authors, both reaction partners were coordinated by *Yb* atom, which resulted in lowering all FMOs and finally accelerating the cycloaddition.

**Scheme 180:**
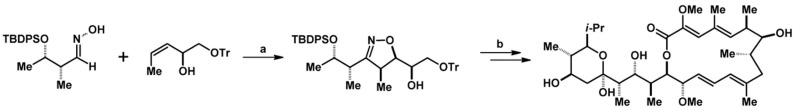
Total synthesis of bafilomycin A1: 3,4,5-trisubstituted 2-isoxazoline ring creation as a crucial step in the synthetic path [193]. a = *t*-BuOCl, CH_2_Cl_2_, −78 °C; next: *i*-PrOH, EtMgBr, 0 °C to rt; 74% yield, *dr* ≥ 95:5; b = next steps.

As Kleinbeck and Carreira reported in their paper from 2009, 1,3-DP cycloaddition involving the appropriate nitrile oxide and dipolarophile was a crucial step in the multistep total synthesis of bafilomycin A1—Scheme 180 [193].

Bafilomycin A1 (isolated in 1983 from a culture of *Streptomyces griseus*) is characterized by broad antibacterial and antifungal activity and selective inhibition of V-type ATPases.

**Scheme 181:**
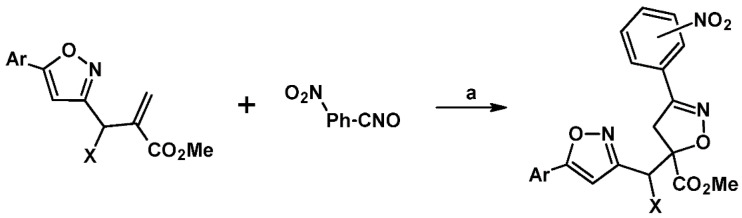
Syntheses of isoxazole-X-isoxazoline hybrids containing 2-, 3- or 4-nitrophenyl group in position 3 (in isoxazoline motif) [194]. Ar, X, nitro group position in phenyl = HO, Ph, 2-O_2_N; AcO, *p*-MeC_6_H_4_, 2-O_2_N; H, Ph, 3-O_2_N; H, 4-O_2_N, Ph; a = Et_3_N, Et_2_O; up to 89% yield.

3,5-disubstituted isoxazole-X-3,5,5-trisubstituted 2-isoxazoline hybrids containing a nitrophenyl group in position 3 (in the isoxazoline motif) were obtained via 1,3-DP cycloaddition of nitro-benzonitrile oxides to methyl acrylate equipped with the aryloxazole motif (attached to double bond via –CHX– linker)—Scheme 181 [194].

According to the authors, the cleavage of the isoxazole ring in the hybrids during the catalytic hydrogenation is influenced by the substituent on the 3-position of the 2-isoxazoline ring.

**Scheme 182:**
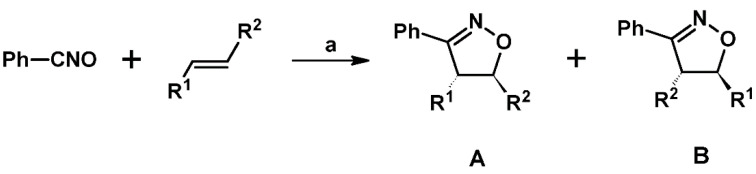
Syntheses of isoxazolines in various solvents, especially in ionic liquids [195]. R^1^, R^2^ = Ph, CO_2_Et; Me, CO_2_Et; Et, CH_2_OH; 12–89% yield; a = tested solvents: MeCN, AcOEt, THF, [BMIM]^+^X^−^, X = [(CF_3_SO_2_)_2_N], [PF_6_], [(NC)_2_N]; Et_3_N, up to 24 h. (**A**,**B**) = mixture of products.

Rosella and Harper described 3,4,5-trisubstituted isoxazolines typically synthesized in various solvents, including ionic liquids—Scheme 182 [195].

Interestingly, the rate and regioselectivity of DP cycloaddition strongly depend on the nature of the solvents; namely, ionic liquids favor the least sterically hindered product. Take, for instance, a comparison of MeCN and [BMIM][PF_6_]: A/B = 6.5 and 12.5, respectively; 44% and 84% yield, respectively. That is consistent with an increased cohesive pressure resulting in a smaller, more sterically demanding transition state.

**Scheme 183:**
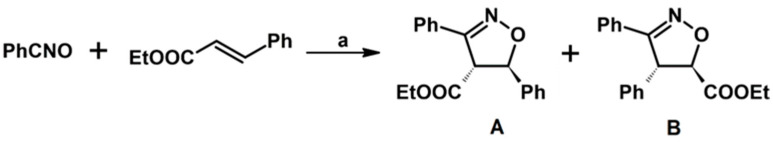
1,3-DP cycloaddition of PhCNO to (*E*)-EtOOCCH=CHPh in various solvents [196]. a = Et_3_N, rt; solvent, (**A**/**B**), = MeCN, 6.5, 44% yield; MeCN + Li[N(CF_3_SO_2_)_2_], 4.6, 15% yield; MeCN + [NBu_4_]BF_4_, 7.5, 30% yield; [BMIM][N(CF_3_SO_2_)_2_], 15.2, 84% yield.

The same year, the significant influence of ionic liquids on the regioselectivity and isoxazoline yields in 1,3-DP cycloaddition was observed by Yau and colleagues—Scheme 183 [195,196].

**Scheme 184:**
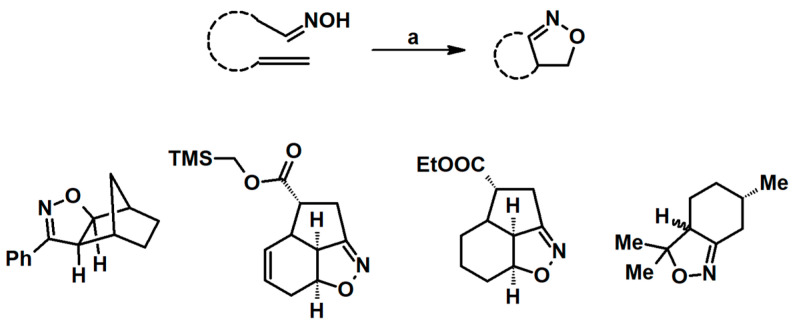
Hypervalent iodide oxidation and 1,3-DP cycloaddition engaged in the syntheses of di- and tricyclic 3,4,5-trisubstituted 2-isoxazolines from oximes via inter- and intramolecular versions of the synthetic route [71]. Selected products are also presented. a = MeOH, CF_3_COOH (catalytic amount), DIB, rt; up to 85% yield.

As Mendelsohn and colleagues presented in their work from 2009, bi- and tricyclic isoxazolines can be prepared via intra- and intermolecular cycloaddition of a CNO motif into a –CH=CH– motif, both coming from two or the same molecules—Scheme 184 [71]. The CNO motif was generated from an oxime motif using hypervalent iodide oxidation.

**Scheme 185:**
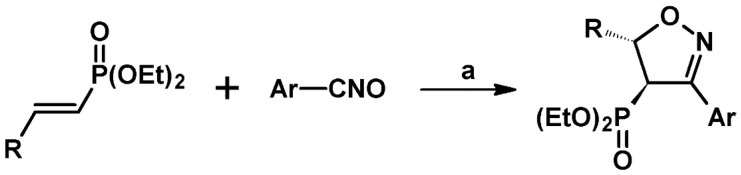
Syntheses of trisubstituted isoxazolines possessing –P(O)(OEt)_2_ motif in position 4 [197]. Ar, R = *n*-hexyl, *p*-FC_6_H_4_; *n*-hexyl, Ph; P, Ph and others; a = THF, Et_3_N, from −10 °C to rt, up to 48 h; up to 69% yield.

Ye’s team reported obtaining 3,4,5-trisubstituted 2-isoxazolines equipped with diethylphosphonate substituent in position 4 by utilizing ArCNO 1,3-DP cycloaddition to (*E*)-RCH=CHPO(OEt)_2_—Scheme 185 [197].

**Scheme 186:**
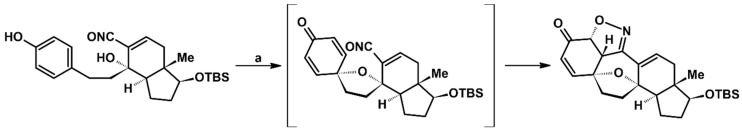
Synthesis of polycyclic isoxazoline via intramolecular 1,3-DP cycloaddition of CNO motif into enone motif [198]. a = PhI(OAc)_2_, CF_3_CH_2_OH, rt, 1 h, next 50 °C, 75 min; 80% yield.

According to Frie et al., intramolecular, fully regio- and stereoselective 1,3-DP cycloaddition of a –CNO motif generated from the appropriate oxime motif and I(III) to enone motif (both, i.e., oxime and enone motifs present in the substrate) led to polycyclic, 3,4,5-trisubstituted isoxazoline—Scheme 186 [198].

Attention should be paid to the preceding 1,3-DP cycloaddition dearomatization of the benzene ring via the formation of a spiroether motif. The resulting isoxazoline is a crucial intermediate in the synthetic route leading to (+)-cortistatin A.

**Scheme 187:**
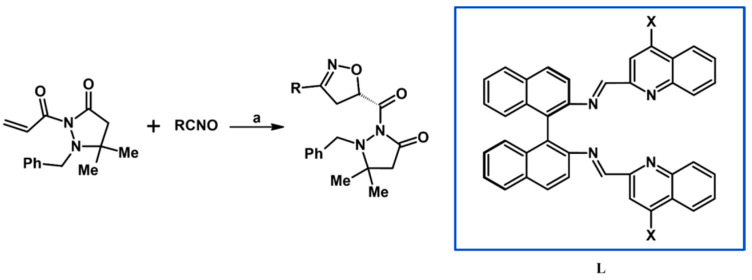
Syntheses of chiral isoxazolines via cycloaddition catalyzed by chiral *Ni*-complex [199]. R = Ph, *p*-MeC_6_H_4_, *p*-MeOC_6_H_4_, *p*-ClC_6_H_4_, *p*-O_2_NC_6_H_4_, *i*-Bu; X = Ph (*R*)-configuration; regioselectivity = up to 95%; ee = up to 91%; a = L, MS 4Å, rt, up to 66 h; up to quantitative yield.

As found in a report from Suga et al., chiral 3,4,5-trisubstituted isoxazolines can be obtained in the 1,3-DP cycloaddition of selected RCNO to 3-(2-alkenoyl)-2-oxazolidinones and 2-(2-alkenoyl)-3-pyrazolidinone derivatives mediated by chiral binaphthyldiimine (BINIM)-Ni(II) complexes [199]. The most attractive results are presented below (Scheme 187).

**Scheme 188:**
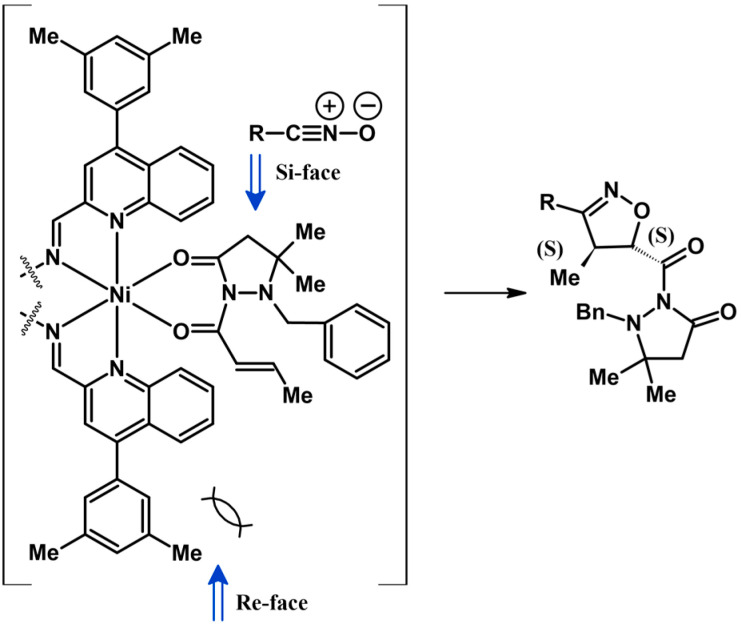
Syntheses of chiral isoxazolines via cycloaddition catalyzed by chiral Ni-complex [199]. Rationalization of the observed enantioselectivity—a substrate complexation by a catalyst—a model.

The cycloaddition mechanism, including the explanation of the observed enantioselectivity as a result of specific substrates’ interaction with the chiral catalyst, is also presented—see Scheme 188 presented below.

**Scheme 189:**
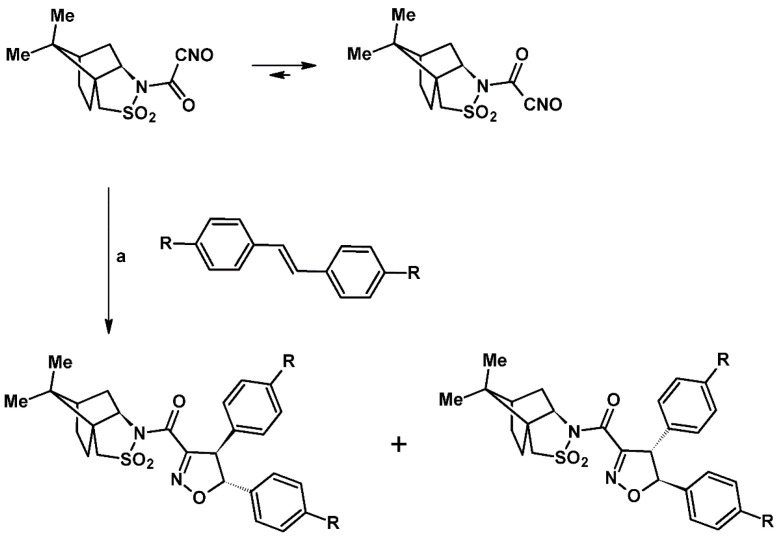
Syntheses of 3,4,5-trisubstituted 2-isoxazolines possessing (2*R*)-bornane-10,2-sultam motif in position 3 [200]. R = H, MeO, Me, CF_3_, O_2_N, Cl, CN, and others; a = KHCO_3_, CHCl_3_, 20 °C, 6 h; up to 90% overall yield.

1,3-DP cycloaddition between 2-oxoethanenitrile oxide derived from (2*r*)-bornane-10,2-sultam and electronically modified 4,4′-disubstituted stilbenes was analyzed by Romanski, Chapuis, and Jurczak in terms of regioselectivity and mechanism—Scheme 189 [200].

It was revealed that the observed diastereoselectivity is related to the electronic nature of the dipolarophiles and may be predicted based on their σ-para or σ-stilbene Hammett parameters. A nonsynchronous mechanism is suggested by calculations and experimental evidence resulting from the cycloaddition of *cis*-stilbene as a model dipolarophile.

**Scheme 190:**
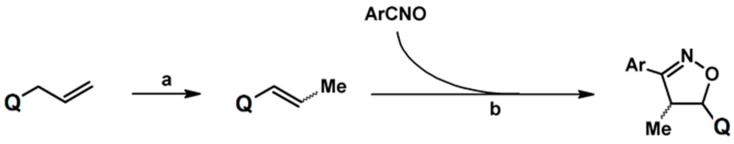
Derivatives of 3-amino-2-isoxazoline: syntheses from Qallyl and ArCNO via tandem double bond migration-1,3-dipolar cycloaddition [201]. Ar = 2,6-Cl_2_C_6_H_3_; Q = NMe_2_, NH(CO)NH_2_, NHCOMe, N(COMe)(*p*-MeC_6_H_4_), NH(2-thienyl), *N*-imidazolyl, N=CH(2,6-Cl_2_C_6_H_3_), and others; up to 95% yield (*cis* + *trans*; for Q = NMe_2_ *cis* only); a = [RuClH(CO)(PPh_3_)_3_] or [RhH(CO)(PPh_3_)_3_] or Verkade’s super base or 18-crown-6; b = NEt_3_, CH_2_Cl_2_, 0–5 °C, then rt, 24 h.

In 2010, derivatives of 5-amino-2-isoxazolines belonging to 3,4,5-trisubstituted 2-isoxazolines were prepared from Qallyl systems and nitrile oxide via tandem double bond migration-1,3-dipolar cycloaddition—Scheme 190 [201].

Cycloadditions were fully regioselective, which was justified in the DFT calculations for the model dipolarophile, i.e., CH_3_CH=CHNH_2_. When the isomerization catalyst was *Rh*- or *Ru*-complexes, a metal scavenger agent, namely phosphotungstic-modified active carbon (STREM), was used to remove Ru and Rh from the product quantitatively.

**Scheme 191:**
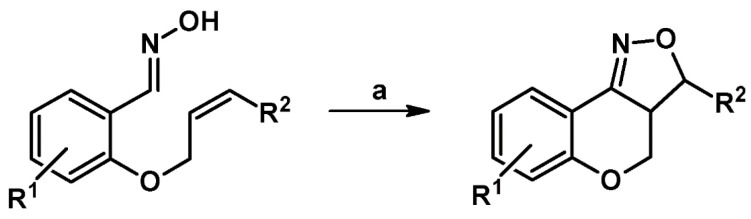
Intramolecular cycloaddition of nitrile group into double bond—syntheses of 3,4,5-disubstituted isoxazolines [6]. R^1^, R^2^, = H, Me; H, Ph; 5-MeO, Ph and others; up to 89% yield; a = HTPI, water, rt, 50 min.

3,4,5-risubstituted 2-isoxazolines were also obtained by Raihan and coworkers via intramolecular dipolar cycloaddition of a nitrile oxide motif to the double bond coming from substituted allyloxy group present in the substrate—Scheme 191 [6]. One should add that the synthetic procedure was analogically applied for the syntheses of 3,4-disubstituted isoxazolines (described in Section 2.2). Importantly, the nitrile oxide motif was generated in situ from the oxime group using air-stable [hydroxy(tosyloxy)iodo]benzene (HTIB) as a dehydration agent.

**Scheme 192:**
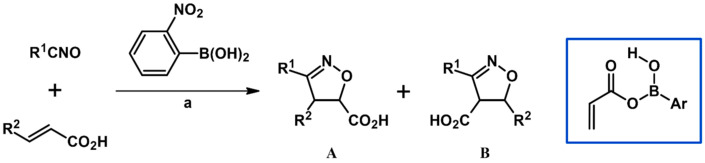
Syntheses of substituted isoxazolines via 1,3-DP cycloaddition mediated by 2-nitrophenyl boronic acid [202]. R^1^, R^2^, (**A**/**B**) = Ph, H, >98/2, 67% yield; C_6_H_4_CH=CH, H, >98/2, 67% yield; CH_2_CH_2_C_6_H_4_, H, >98/2, 65% yield; Ph, Me, <2/98, 77% yield; Ph, Ph, <2/98, 65% yield; a = 2-nitrophenylboronic acid (5mol%), ClCH_2_CH_2_Cl, 25 °C, 24–48 h.

The preparation of 3,4,5-trisubstituted 2-isoxazolines in the regioselective 1,3-DP cycloaddition, mediated by 2-nitrophenyl boronic acid, of nitrile oxide to acrylic acid derivatives was covered in work by Zheng, McDonald, and Hall from 2010—Scheme 192 [202].

The activating role of boronic acid is to convert the COOH group into a boronic ester intermediate, whose LUMO has a lower energy than the LUMO of unsaturated acid.

**Scheme 193:**
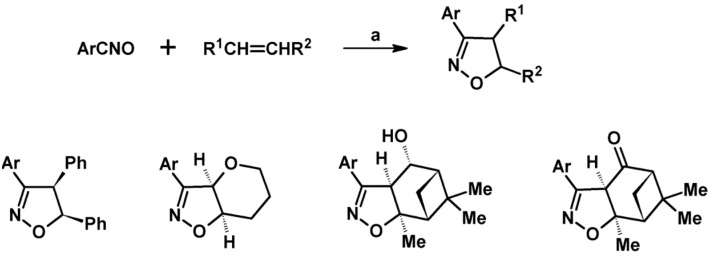
Water as an effective solvent for the 1,3-DP cycloaddition [203]. Products—examples. Ar = 2,4,6-trimethyl-3,5-dichlorophenyl; a = water, rt; up to 91% yield.

1,3-DP cycloaddition of stable nitrile oxide to various dipolarophiles R^1^CH=CHR^2^ was performed by Molteni and Del Buttero in pure water giving 3,4,5-trisubstituted 2-isoxazolines—Scheme 193 [203].

Water-insoluble or sparingly soluble monosubstituted dipolarophiles react smoothly with ArCNO, especially in the presence of NaCl as an enhancer of the reaction medium’s ionic strength. On the contrary, adding SDS as a surfactant in the reaction mixture did not significantly reduce the reaction times. Interestingly, reaction with chiral dipolarophiles, namely (*S*)-*cis*-verbenol and (1*S*)-(−)-verbenone, allowed researchers to obtain almost enantiopure (*ee* > 95) isoxazolines via complete diastereofacial-selective cycloadditions.

**Scheme 194:**
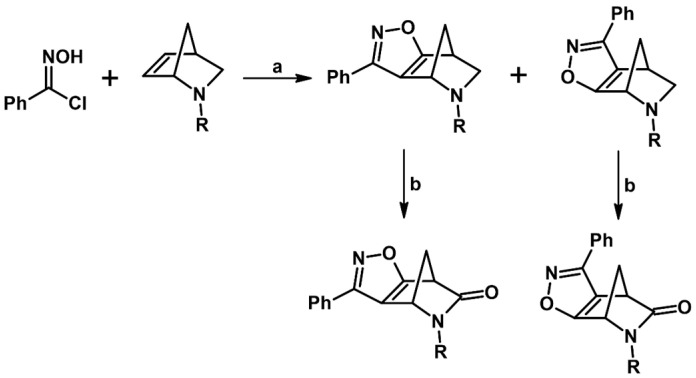
1,3-DP cycloaddition of PhCNO to 2-azanorbornene as a starting point in the synthetic procedure leading to the tricyclic γ-lactams [204]. R = Me, Et, *i*-Pr, *t*-Bu; a = Et_3_N, CH_2_Cl_2_, 0 °C, 48 h, up to 93% yield (both isomers); b = oxidation by RuO_4_ generated in situ from RuO_2_·H_2_O + NaIO_4_.

The same year, in another report from Quadrelli’s team, the synthesis of conformationally constrained γ-lactams was described. Those molecules were prepared via 1,3-DP cycloaddition of in situ-generated PhCNO to 2-azanorbornene, followed by RuO_4_ oxidation of obtained isoxazolines—Scheme 194 [204].

The analogical strategy was applied in the next Quadrelli et al. paper devoted to RuO_4_-catalyzed oxidation of isoxazolino-2-azanorbornane derivatives leading to tricyclic lactams and peptidomimetic γ-amino acids [205].

**Scheme 195:**
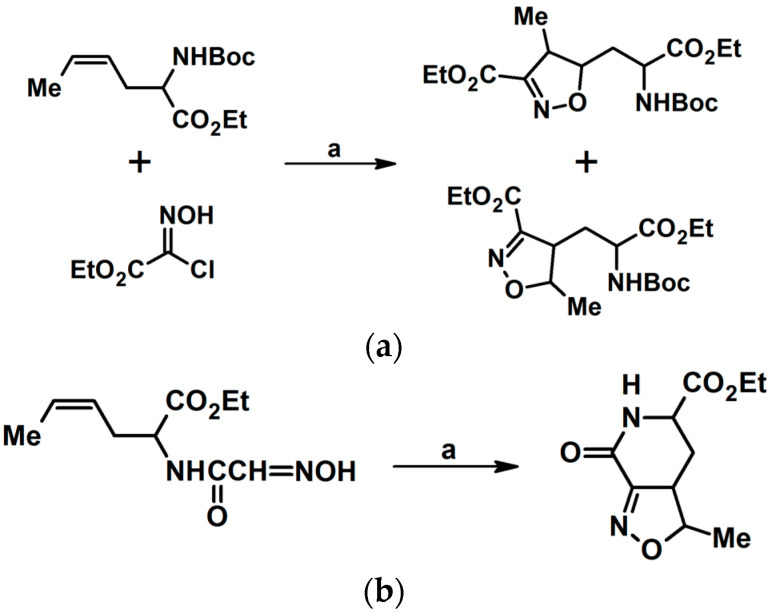
Syntheses of 3,4,5-trisubstituted isoxazolines possessing special functional groups [206]. (**a**): a = NaHCO_3_, EtOAc, 80 ^°^C, μW, 1 h; 70% yield; (**b**): a = NaOCl, CH_2_Cl_2_, rt, 3 h; 65% yield.

Pinto and colleagues once again reported synthesizing 3,4,5-trisubstituted 2-isoxazolines equipped with desired functional groups (COOR and protected NH_2_) through the inter- and intramolecular cycloaddition of nitrile oxide to the carbon–carbon double bond—Scheme 195 [206].

All obtained isoxazoline-based amino acids mimicked known glutamate receptor ligands as they were tested regarding receptor binding affinities to AMPA or NMDA receptors.

**Scheme 196:**
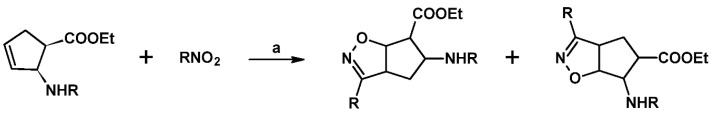
Synthesis of isoxazoline-fused cispentacin stereoisomers [207,208]. a = DMAP, THF, Boc_2_O, rt, 15 h, up to 68% yield.

In another group’s publication, the synthesis of isoxazoline-fused cispentacin derivatives, including enantiomerically enriched forms via 1,3-DP cycloadditions of in situ-generated (from nitroalkanes) RCNO to ethyl 2-amino-3-cyclopentene-carboxylates was reported—Scheme 196 [207,208]. Unfortunately, this method was neither regio- nor stereoselective; all regio- and stereoisomers were formed.

**Scheme 197:**
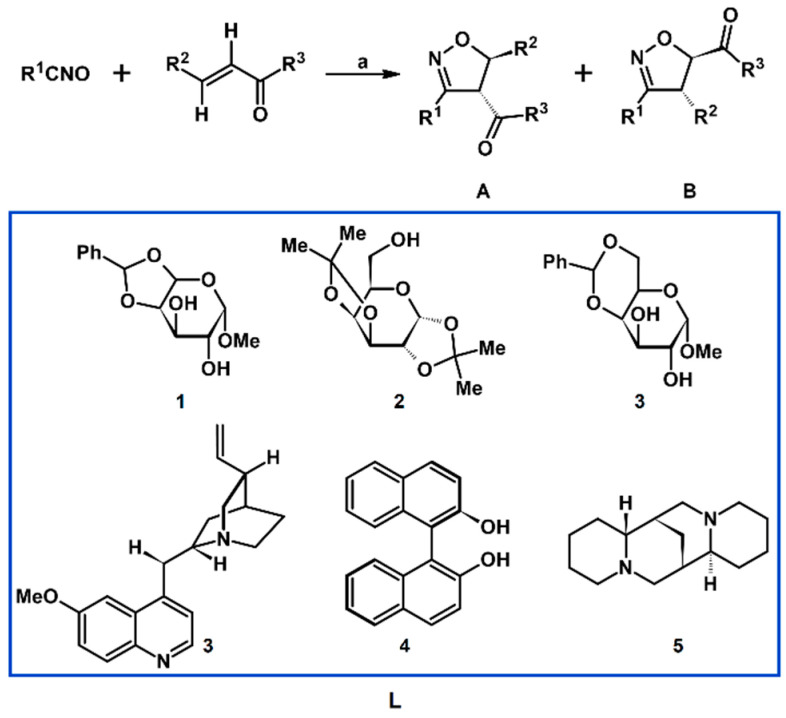
Syntheses of chiral 3,4,5-trisubstituted isoxazolines via 1,3-DP cycloaddition of RCNO to α,ß-unsaturated amides and esters, catalyzed by chiral ligand–Lewis acid systems (selected results) [209,210]. *Mg*-catalysts: R^1^ = *p*-CF_3_Ph; R_2_ = Me; R^3^ = *o*-MeOC_6_H_4_; a = Mg(OTf)_2_–L1 or Mg(OTf)_2_–L2: overall yield = 69% for Mg(OTf)_2_–L1 and 94% for Mg(OTf)_2_–L2; (**A**/**B**) = 38/62 for Mg(OTf)_2_–L1 and 67/33 for Mg(OTf)_2_–L2; *ee* A and B = 0% and 99,2 for Mg(OTf)_2_–L1; *ee* (**A**,**B**) = 0,2% and 96 for Mg(OTf)_2_–L2; Yb-catalysts: L, R^1^, R^2^, R^3^, % y, A/B, %*ee* A, %ee = 1, *p*-CF_3_C_6_H_4_, Me, NH(*o*-MeOC_6_H_4_), 74, 60/40, 0.4, 95; 2, *p*-CF_3_C_6_H_4_, Me, NH(*o*-MeOC_6_H_4_), 84, 80/36, 0, 95; 3, *p*-*i*Pr-C_6_H_4_, Me, NHCH_2_-2-furyl, 53, 36/64, 3, 94 and others; *Ti*-catalysts: a = 24 h, rt, solvent; TiCl_4_-L. R^1^, R^2^, R^3^, (**A/B**), %*ee* (**A**), %*ee* (**B**) = cyclohexyl, CO_2_Me, H, 25/75, 99, 31; Me, CO_2_Bn, H, 65/35, 93, 100; H, Et, CH_2_CO_2_Me, 82/18, 65, 97; H, Et, CH_2_OCOCH_2_OMe, 81/19, 26, 99; overall (**A**,**B**); up to 40% yield.

The following comprehensive research study from Gucma and Gołębiewski came out in 2011 and concerned 1,3-DP cycloaddition between ArCNO and α,ß-unsaturated amides and esters. It was used to obtain chiral substituted isoxazolines—Scheme 197 [209,210]. These reactions were mediated by complexes of carbohydrates A and B alkaloids ((+)-cinchonine, (−)-cinchonidine, (−)-sparteine), R-BINOL with Yb(OTf)_3_, Yb(ClO_4_)_3_, YbCl_3_, TiCl_4_, Mg(OTf)_2_, MgBr_2_, and CsF complexes without any auxiliaries.

Usually, 4*R*,5*R*-trisubstituted isoxazolidines were obtained, but in the cases of the Yb(ClO_4_)_3_-L4 or –ent-L4 systems, L4- or L3-CsF, and R-BINOL-Yb(OTf)_3_ catalytic systems, reactions led to 4S,5S-isoxazolidines. Unfortunately, attempts to decrease the amounts of catalytic systems from equimolar to catalytic quantities were effective only for R-BINOL-Yb(OTf)_3_ and L4-Yb(OTf)_3_ systems. In most cases, cycloaddition was not regioselective, but in many cases, it was highly enantioselective for both or one of the regioisomers [209,210].

**Scheme 198:**
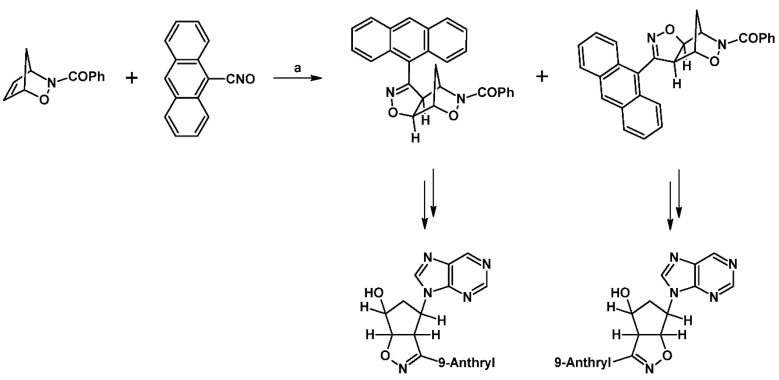
1,3-DP cycloaddition as an initial step in the synthetic method leading to the isoxazoline-fused cyclopentane-purine derivatives [211]. X = Cl or NR^1^R^2^ (R^1^, R^2^ = H, H; Me, H; Et, H; Me, Me); a = CH_2_Cl_2_, rt, 48 h; 39% and 47% yield.

In 2012, another report from Quadrelli’s team, Moggio and coworkers presented novel isoxazoline derivatives. Starting from the exo-selective 1,3-DP cycloaddition of the 9-anthracenenitrile oxide to the *N*-benzoyl-2,3-oxazanorborn-5-ene followed by the subsequent transformations, derivatives of isoxazolino-carbocyclic nucleoside analogues equipped with the anthracene motif were obtained—Scheme 198 [211].

Selected nucleoside analogues were tested for their inhibitory activity against some viruses, namely Hepatitis B and C, Human Papilloma virus, as well as Influenza viruses of type A and B. Good anti-viral activity was observed for compound B (X = NHEt) with no cellular toxicity at the dose tested in the case of Human Papilloma virus.

**Scheme 199:**
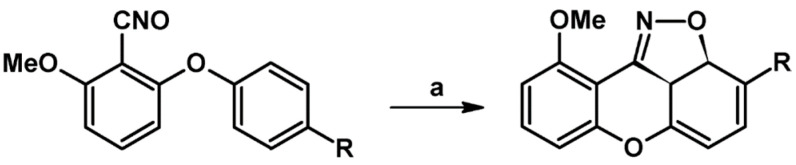
Syntheses of 3,4,5-trisubstituted 2-isoxazolines via intramolecular dipolar cycloaddition of nitrile oxide motif to the neighboring benzene ring [212]. R = H, Me, MeO, CO_2_Et; a = NCS, NEt_3_, CHCl_3_, 0 °C, then rfx, up to 4.5 h, up to 93% yield.

The same year, Yonekawa et al. described intramolecular 1,3-dipolar cycloaddition engaging both fragments of Ar^1^–*O*–Ar^2^-type molecules applied for the syntheses of 3,4,5-substituted 2-isoxazolines—Scheme 199 [212]. In these perfectly *cis* additions coming from Ar^1^_,_ the nitrile motif reacts with a double bond coming from Ar^2^_,_ which is accompanied by the dearomatization of Ar^2^.

The substituents on the benzene rings markedly affected the reaction rate, yield, and structure of the final product.

**Scheme 200:**
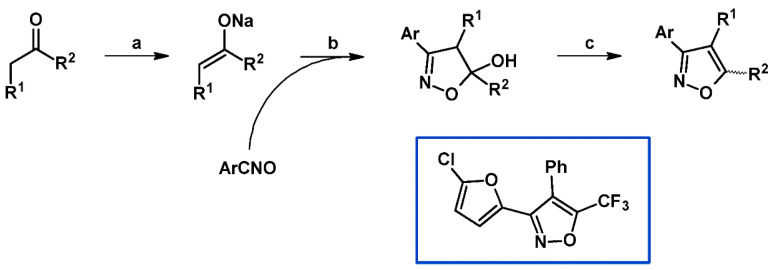
Syntheses of trisubstituted isoxazoles starting from 1,3-DP cycloaddition of ArCNO to ketoenolates [213]. R^1^, R^2^ = *m*-ClC_6_H_4_, Ph; *o*-ClC_6_H_4_, *o*-FC_6_H_4_; 5-chlorofuran-2-yl, CF_3_ and others; a = NaH/THF, 1 h, 0 °C; b = ArCNO, THF, 12 h, rt, aq NH_4_Cl; c = aq. Na_2_CO_3_, MeOH.

**Scheme 201:**
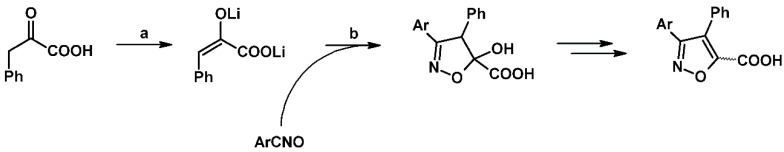
Syntheses of trisubstituted isoxazoles starting from 1,3-DP cycloaddition of ArCNO to dilithium salt of 3-phenyl-2-oxopropanoic acid enol form [213]. Ar = 5-chlorofuran-2-yl, furan-2-yl, tetrahydrofuran-2-yl’ a = LDA, THF, 1 h, 0 °C; b = ArCNO, THF, 16 h; sat. NH_4_Cl; 2 N HCl.

Vitale and colleagues reported in 2013 other 3,4,5-trisubstituted 2-isoxazolines. Using the appropriate sodium enolate or dilithium salt, they were prepared via dipolar cycloaddition and applied for the syntheses of appropriate trisubstituted isoxazoles—Scheme 200 and Scheme 201 [213].

Pharmacological characterization and docking analysis of obtained diarylisoxazoles revealed that the derivative depicted in the frame (Scheme 200) represents a candidate for preclinical development as an antithrombotic agent. It is over 1000-fold more potent to inhibit COX-1 than COX-2.

**Scheme 202:**
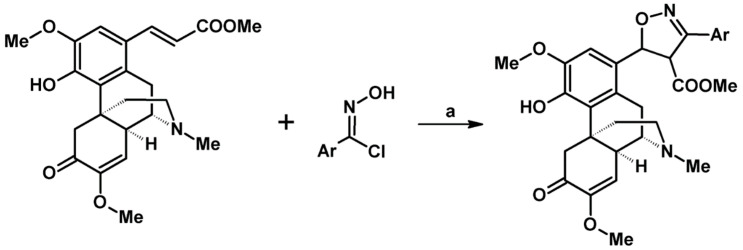
Sinomenine derivative preparation via 1,3-DP cycloaddition of in situ-generated ArCNO to cinnamate ester equipped with the sinomenine motif [214]. Ar = Ph, *m*-BrC_6_H_4_, *m*-ClC_6_H_4_, *p*-BrC_6_H_4_, *p*-MeOC_6_H_4_, and others; a = NCS, DMF, rt, then 85 °C; 96% yield.

As found in another report from 2013, sinomenine derivatives possessing an isoxazoline motif in the C-1 position were obtained via 1,3-DP cycloaddition of ArCNO to sinomenine derivative equipped with the CH=CHCOOEt motif in position C-1—Scheme 202 [214].

Interestingly, using the continuous-flow capillary microreactor for these cycloadditions significantly improved the reaction yield and shortened the reaction time. All obtained derivatives were tested concerning their biological inhibition and suppressing ability.

**Scheme 203:**
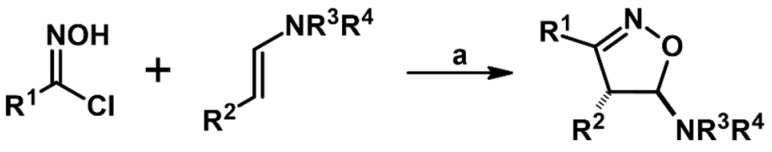
Syntheses of trisubstituted isoxazolines as precursors for the preparation of 3,4-disubstituted isoxazoles [215]. R^1^, R^2^, R^3^, R^4^ = Ph, *i*-Pr, pyr-1-yl; 2-thienyl, Ph, pyr-1-yl; *p*-ClC_6_H_4_, Ph, pyr-1-yl; 1-naphthyl, Ph, pyr-1-yl; Ph, *i*-Pr, Et, Et and others; a = Et_3_N, CH_2_Cl_2_, 0 °C to rt; up to 99% yield.

In 2013, an article by Jia et al. described 3,4,5-trisubstituted 2-isoxazolines obtained via highly regioselective 1,3-DP cycloaddition between both in situ-generated RCNO and enamines—Scheme 203 [215].

In the final step, the obtained 3,4,5-trisubstituted dihydroisoxazoles can be smoothly converted into 3,4-disubstituted isoxazoles through a high-yielding Cope-elimination.

**Scheme 204:**
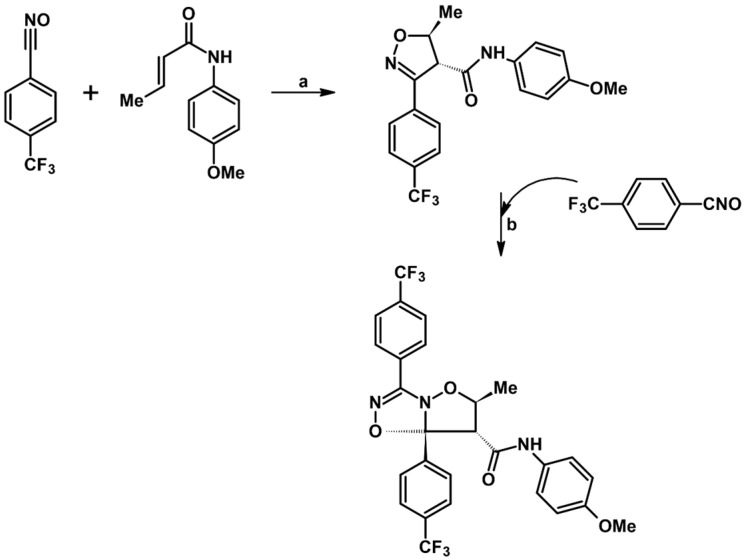
1,3-DP cycloaddition of RCNO to acrylamide (an example): predicted and unexpected products [216]. a = rt, 19 h; 45% yield; b = rt; 7% yield.

In 2014, Gucma and Gołębiewski revisited the cycloaddition of *p*-F_3_CC_6_H_4_CNO to *N*-(4-methoxyphenyl)acrylamide, which produced a bicyclic tetrahydro-oxazolo-(3,2-*b*)[1,3]oxazine-2-carboxamide derivative as a result of *N*-acylation of the initially formed isoxazoline—Scheme 204 [216]. Interestingly, the side product mentioned above could be obtained as the main product when reaction conditions were modified.

Moreover, the cycloaddition of the same dipole to *N*-(4-methoxyphenyl)crotonamide yielded dihydro [1,2]-oxazolo [2,3-*d*][1,2,4]oxadiazole-7-carboxamide because of the second addition of the dipole to the C═N bond of the initially formed 2-isoxazoline.

**Scheme 205:**
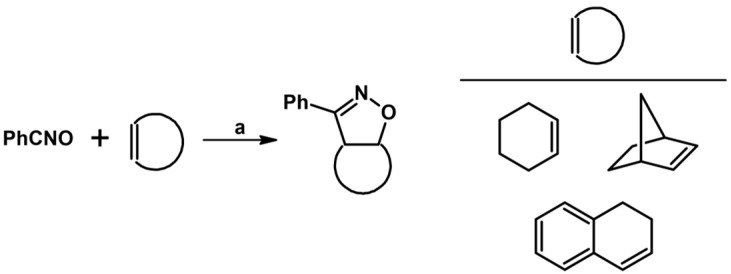
Syntheses of trisubstituted isoxazolines using in situ-generated (from PhCH=NOH) ArCNO and cyclic alkenes [107]. a = *m*-ClC_6_H_4_COOOH, PhI, CF_3_CH_2_OH, rt, 12 h; up to 70% yield.

The same year, Xiang, Li, and Yan reported the preparation of 3,4,5-trisubstituted 2-isoxazolines via 1,3-DP cycloaddition of in situ-generated nitrile oxide to cycloalkenes as dipolarophiles—Scheme 205 [107]. Nitrile oxide was generated from benzaldoxime and hypervalent iodine; the latest was created via the reaction of PhI with *m*-ClC_6_H_4_COOOH.

**Scheme 206:**
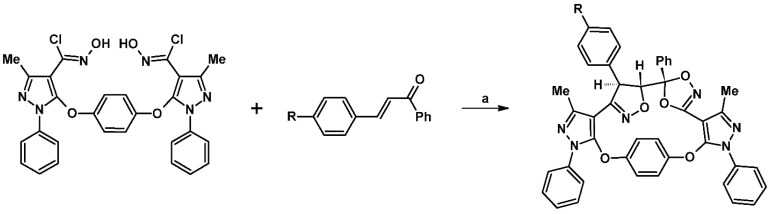
Syntheses of potent antioxidants equipped with isoxazoline, pyrazole, and dioxazole moieties [217]. R = H, Me, MeO; a = Et_3_N, CH_2_Cl_2_, rt; up to 33% yield.

The next year, macrocycles containing a 3,4,5-trisubstituted isoxazoline motif were synthesized by Zeng et al.—Scheme 206 [217]. It should be noted that the final products are created as an effect of 1,3-DP cycloaddition of nitrile oxide to C=C and C=O double bonds.

Interestingly these compounds exhibit a highly radical-scavenging activity against model, stable 1,1-diphenyl-2-picrylhydrazyl (DPPH), comparable to that of vitamin E.

**Scheme 207:**
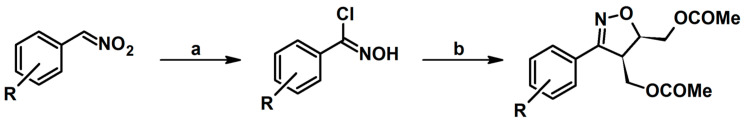
Syntheses of *cis*-3,4,5-trisubstituted 2-isoxazolines [218]. R = H, *p*-Me, *p*-Cl, *p*-F, *p*-CN, *m*-F, *m*-O_2_N, and others; a = chlorine; b = Et_3_N, Ch_2_Cl_2_; up to 37% yield.

Syntheses of *cis*-isoxazolines were obtained via 1,3-DP cycloaddition of benzonitrile oxide derivatives to (*Z*)-2-buten-1,4-diol diacetate—Scheme 207 [218].

As the authors reported, the perfect transition of dipolarophile configuration into all cycloadducts indicated the concerted reaction mechanism. The correlation analysis using Hammett substituent constant and other constants and regression analysis allowed researchers to explain the effect of the substituent on the ^13^C NMR chemical shift.

**Scheme 208:**
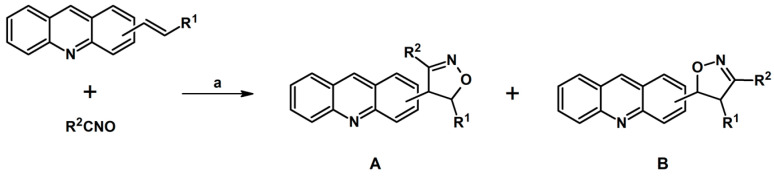
Syntheses of 3,4,5-trisubstituted isoxazolines bearing acridin-9- or 4-yl moiety [219]. R^1^ = 2,4,6-Me_3_C_6_H_2_, 2,4,6-(MeO)_3_C_6_H_2_, 2,6-Cl_2_C_6_H_3_, 2,4,6-(MeO)_3_-3-BrC_6_H; R^2^ = Ph, COOEt, or CONH_2_ (the last was examined only for acridin-4-yl motif); a = CHCl_3_, rt; up to 38% yield (both regioisomers). (**A**,**B**) = mixture of products.

Other 3,4,5-trisubstituted isoxazolines bearing an acridin-9- or 4-yl motif were synthesized via 1,3-DP cycloaddition of stable ArCNO to dipolarophiles derived from 9- or 4-vinyl acridin—Scheme 208 [219]. Importantly, cycloaddition was fully regioselective for one of the 4-vinylacridin (R^1^ = COOMe, R^2^ = Ph); only A regioisomer was created.

As stated by Vilková, Maľučká, and Imrich, a strong correlation was observed between ^13^C NMR chemical shifts of dipolarophilic CH=CH carbons and 1,3-DP cycloadditions regioselectivity. Moreover, the ratios of regioisomers were similar for both acridin-9- and 4-yl dipolarophiles. In general, the polarity of the CH=CH bond, donor effects in ArCNO, and stabilization by stacking of aromatic substituents in the products determined the regioselectivity of examined 1,3-DP cycloaddition. The problem of regioselectivity and relations between structure and reactivity were deeply examined, especially by NMR as well as mass spectrometry and X-ray crystallography in subsequent work [220].

**Scheme 209:**
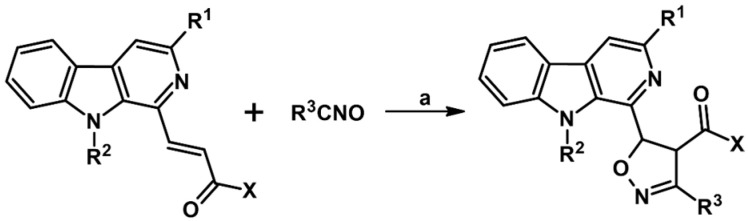
Syntheses of ß-carboline and isoxazoline-based molecular hybrids via 1,3-DP cycloaddition [221]. R^1^, R^2^, R^3^, X = CO_2_Me, Me, Ph, OEt; CO_2_Me, Et, *p*-ClC_6_H_4_, OEt; CO_2_Et, Me, *p*-MeC_6_H_4_, OEt; CO_2_Me, Et, *p*-ClC_6_H_4_ and others; a = Et_3_N, THF, −78 °C to rt, 3–7 h; up to 93% yield.

Molecular hybrids containing ß-carboline and isoxazoline motifs were obtained by Singh and coworkers in 2016. The metal-free procedure involved 1,3-DP cycloaddition of nitrile oxide to –CH=CH– dipolarophile fragment present in the carboline motif—Scheme 209 [221].

In the next step, the obtained isoxazolines underwent aromatization to appropriate isoxazoles.

**Scheme 210:**
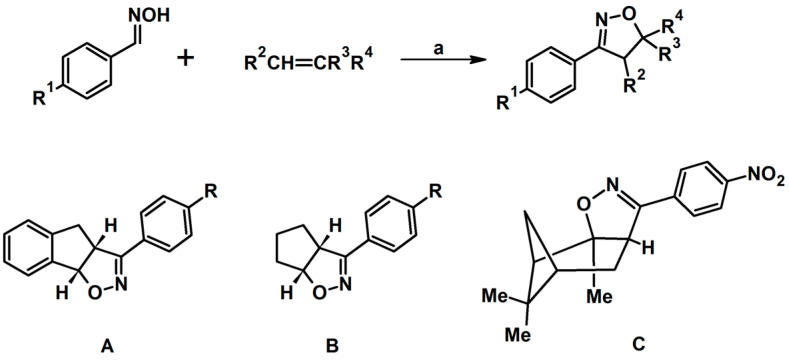
Isoxazolines obtained in water conditions [222]—selected products. a = water-acetone, phosphate buffer, pH = 4.0, rt, 15 h; up to 81% yield (**A**); up to 83% (**B**); 48% yield (**C**).

Another 2016 report presented 2-isoxazolines obtained via 1,3-DP cycloaddition of various RCNOs to diverse dipolarophiles, including chiral pinenes—Scheme 210 [222]. Interestingly, nitrile oxides can be generated from oxymoyl halides in an aqueous solution without catalysts. Contrary to the conventional method, the RCNO formation proceeds not in basic but under mildly acidic conditions. The procedure mentioned above is characterized by excellent stereoselectivity, and it was efficient for synthesizing enantiomerically pure isoxazolines.

According to the authors, the formation of RCNO from RC(Cl)=NOH in water is initiated by the loss of a chlorine atom from the oxime chloride with the subsequent abstraction of the oxime proton by the chloride ion. Nitrile oxide is well-dissolved in water because it is charged. Thus, the solubility drives the reaction forward and favors the production of nitrile oxide in water.

**Scheme 211:**
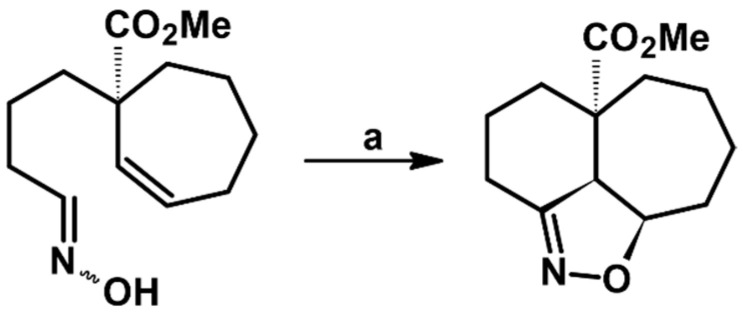
Intramolecular cycloaddition of in situ-generated –CNO motif into –CH=CH– motif leading to tricyclic isoxazoline belonging to 3,4,5-trisubstituted isoxazolines [223]. a = NCS, pyridine, CHCl_3_, 60 °C; 82% yield.

Intramolecular 1,3-DP cycloaddition, presented by Li and colleagues, was one of the crucial steps in the synthetic approach for constructing the ABF ring systems of 7,17-seco-type C 19-diterpenoid alkaloids—Scheme 211 [223].

**Scheme 212:**
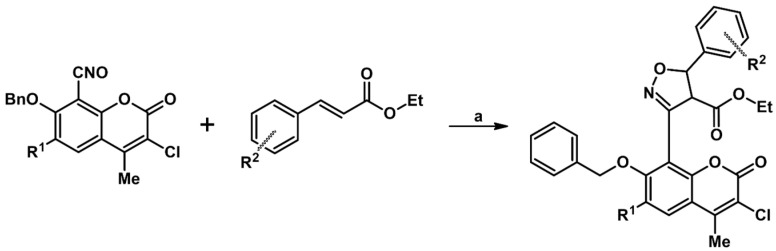
1,3-DP cycloaddition engaged in the syntheses of coumarin equipped with isoxazoline moieties [224]. R^1^, R^2^ = H, H; Cl, *m*-CF_3_; Br, *m*-CF_3_; Me, 3,5-F_2_; and others a = Et_3_N, CH_2_Cl_2_, rt, 24 h; up to 75% yield.

In another article from 2016, the acquisition of 3,4,5-trisubstituted 2-isoxazoline equipped with a coumarin moiety was reported. Those molecules were obtained via 1,3-DP cycloaddition of ethyl 3-aryl prop-2-enoate to nitrile oxide possessing a coumarin moiety—Scheme 212 [224].

Antibacterial tests against Gram-positive and Gram-negative bacteria revealed that all obtained coumarin–isoxazoline hybrids are active against Gram-positive and Gram-negative bacteria—from moderate to suitable levels. Moreover, the presence of bromine in the coumarin motif significantly enhanced the activity of tested compounds.

**Scheme 213:**
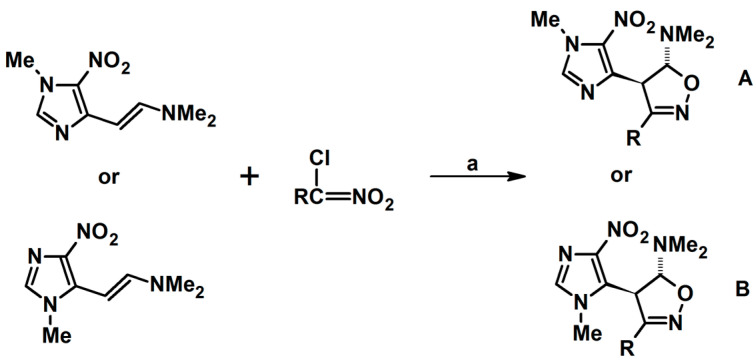
Syntheses of 3,4,5-trisubstituted 2-isoxazolines equipped with 1,3-diazolyl and dimethylamine moieties [225]. R = Ph, *p*-ClC_6_H_4_ (**A**), or cyclohexyl (**B**); a = 1,4-dioxane, rt, 12–48 h; 67% (for Ph), 65% (for *p*-ClC_6_H_4_), and 21% (for cyclohexyl) yields.

As Efimov and colleagues reported in 2016, 1,3-diazolyl and dimethylamine motifs bearing 2-isoxazolines were formed in the fully regio- and stereoselective 1,3-DP cycloaddition of RCNO to (*E*)-(Diazolyl)CH=CHNMe_2_ dipolarophiles—Scheme 213 [225].

Results of comprehensive theoretical studies are presented in Section 3 of this review.

**Scheme 214:**
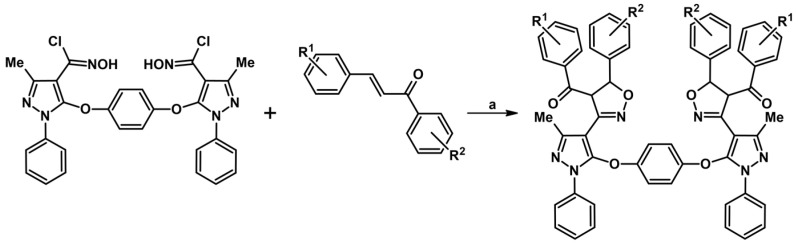
Bis-isoxazolines obtained via 1,3-DP cycloaddition [226]. R^1^, R^2^ = H, Cl, Br, O_2_N, and other substituents in the position *o*-, *m*-, or *p*. a = EtONa, CHCl_3_, 50 °C; up to 79% yield.

The same year, Zeng et al. reported the syntheses of bisisoxazolines containing an isoxazole moiety involving the appropriate ONC–L–CNO and (*E*)-styryl-phenyl ketones as dipolarophile—Scheme 214 [226].

In vitro antioxidant activity tests of obtained bis-isoxazolines revealed that the compounds with nitro- and chloro-substituents at the *para*-position of the benzene ring were more effective as an antioxidant and radical scavenger than butylated hydroxytoluene, trolox, caffeic acid, and ascorbic acid, respectively.

**Scheme 215:**
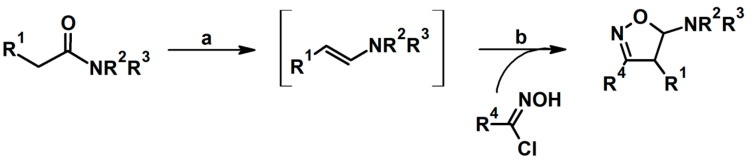
Syntheses of 3,4,5-trisubstituted 2-isoxazolines equipped with NR_2_ group in position 5 from amides [227]. R^1^, R^2^, R^3^, R^4^ = Ph, *N*-morpholinyl, *p*-MeOC_6_H_4_; Ph, *n*-Bu, *n*-Bu, *p*-MeOH; Ph, Ph, Me, *p*-MeOC_6_H_4_; *p*-BrC_6_H_4_, *N*-piperidyl, *p*-MeOC_6_H_4_; *p*-O_2_NC_6_H_4_, *N*-piperidyl, *p*-MeOC_6_H_4_, and others; a = [Mo(CO)_5_], TMDS, AcOEt, 65 °C, 0.5 h; b = Et_3_N, rt, 1 h; up to 96% yield.

Slagbrand and coworkers used a highly effective one-pot procedure to prepare trisubstituted isoxazolines involving 1,3-DP cycloaddition and both in situ generated RCNO and dipolarophiles—Scheme 215 [227].

**Scheme 216:**
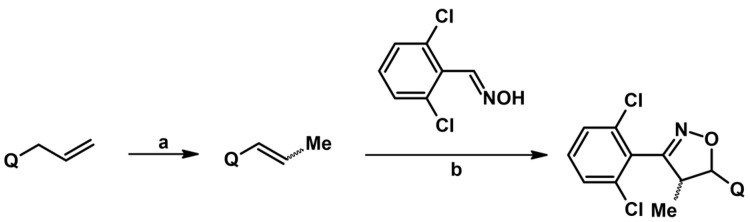
Application of 18-crown-6-*t*-BuOK super-base catalytic system for one-pot synthesis of 3,4,5-trisubstituted 2-isoxazolines from Qallyl via isomerization to Q(1-propenyl)-nitrile oxide generation-1,3-DC [228,229]. Q, *Z*/*E* (after isomerization of Qallyl to Q(1-propenyl), *cis*/*trans* = *n*-BuO, 100%*Z*, 100%*cis*; PhO, PhS, *N*-imidazolyl; up to 75% yield; a = *t*-BuOK, 18-crown-6; b = H_2_O, KHCO_3_ when Cl_2_C_6_H_3_C(Cl)=NOH.

Krompiec’s team described other 3,4,5-trisubstituted 2-isoxazolines in the 2017 article and 2020 Polish patent. Mentioned molecules were obtained from selected allyl compounds in a one-pot cascade: (a) Qallyl isomerization to *Q*(1-propenyl); (b) adding water and KHCO_3_, which caused the decrease in reaction environment basicity; (c) adding oxymoyl chloride and in situ nitrile oxide generation; (d) 1,3-DP cycloaddition of nitrile oxide to *Q*(1-propenyl), which resulted in isoxazoline formation—Scheme 216 [228,229].

**Scheme 217:**
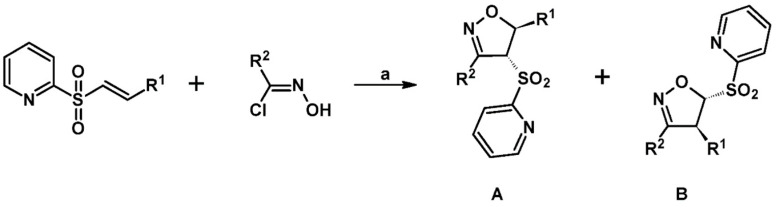
Syntheses of 3,4,5-trisubstituted isoxazolines via dipolar cycloaddition of nitrile oxide to (*E*)-vinyl sulfones possessing 2-PySO_2_ and alkyl or aryl groups, especially CF_3_ group [230]. a = K_2_CO_3_, Et_2_O, 25 °C, 24 h; R^1^, R^2^ = CF_3_, Ph; CF_3_, *p*-FC_6_H_4_; CFH_2_, Ph; CF_2_Br, Ph; F_5_C_2_, Ph and others; up to 87% yield; (**A**/**B**) = up to 99/1.

Cycloaddition of nitrile oxides to substituted-vinyl-(2-pyridyl) sulfones leading to 3,4,5-trisubstituted 2-isoxazolines was presented by Ou et al.—Scheme 217 [230]. The product yield was exceptionally high when R^1^ = perfluoroalkyl or CF_2_Cl or CF_2_Br. On the contrary, a significant decrease in isoxazoline yield was observed when R^1^ = Me, Ph, or CHF_2_ instead of CF_3_.

In summary, the version presented above of 1,3-dipolar cycloaddition of nitrile oxides to (*E*)-dipolarophiles equipped with (2-Py)SO_2_ and perfluoro- or CF_2_Br or CF_2_Cl substituents provided a wide range of appropriate 2-isoxazolines with perfect diastereoselectivities (31 examples, up to 85% yield and >99:1 *dr*).

**Scheme 218:**
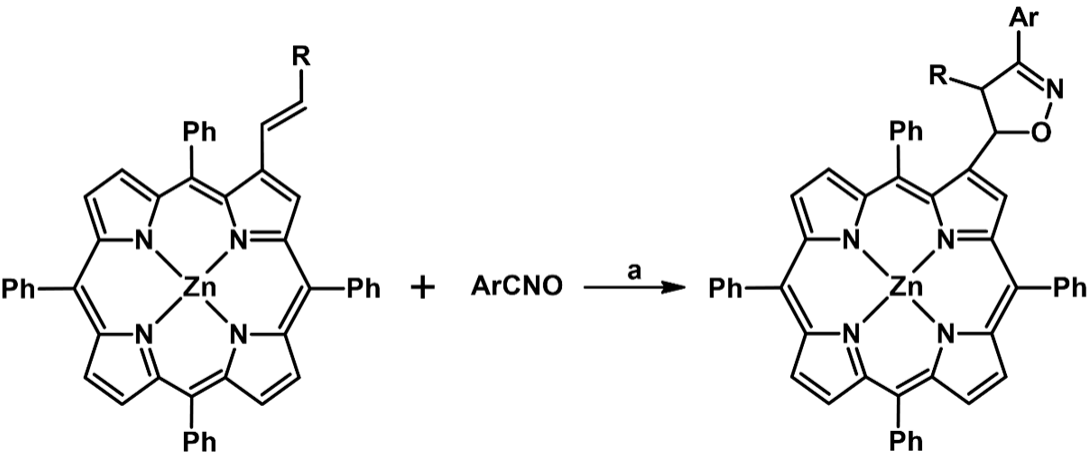
1,3-DP cycloaddition engaged in the syntheses of *Zn*-porphyrin equipped with isoxazoline motif [231]. R = *p*-methoxycarbonylphenyl; a = PhMe, rfx, 6 h. 30% yield (for R = 2,4,6-Me_3_Ph), 48% yield (for R = 2,6-Cl_2_C_6_H_3_).

Liu, Ma, and Feng synthesized two isoxazoline-substituted *Zn*-porphyrin derivatives via the regioselective 1,3-DP cycloaddition of ArCNO to *Zn*-vinyl-porphyrin—see Scheme 218 [231]. Importantly, the regioselectivity was forced by steric effects.

Additionally, in the crystal, a pair of enantiomeric modified porphyrins assembled into a dimeric structure with the fifth chelation of a Zn^2+^ ion by the oxygen atom coming from the carbonyl group of the other molecule.

**Scheme 219:**
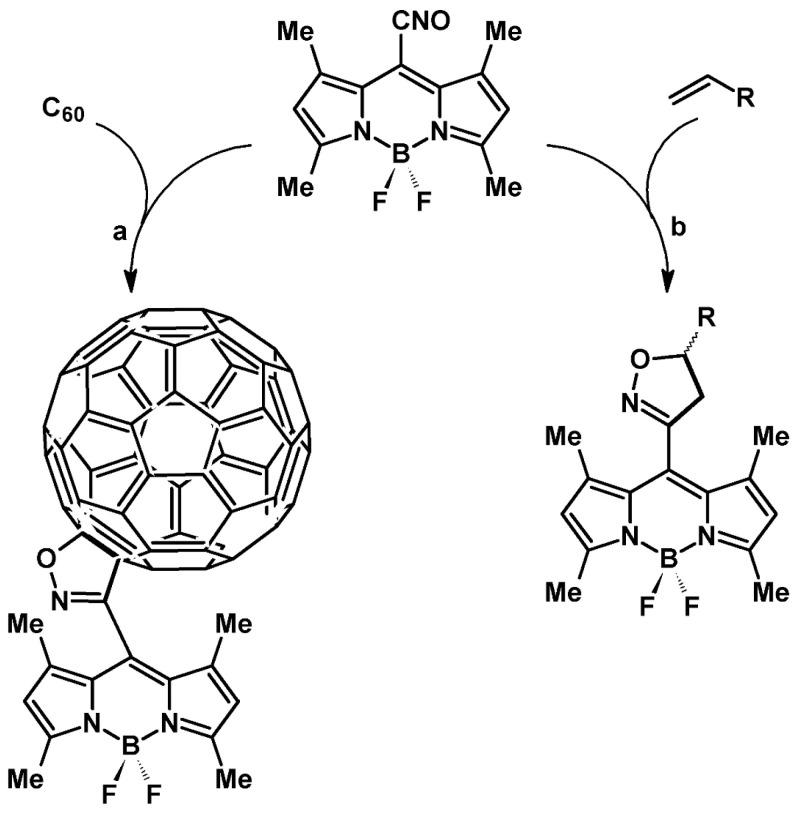
1,3-DP cycloaddition involved in the syntheses of BODIPYs equipped from isoxazoline moieties [232]. R = Ph, COOH, COOMe; a = toluene, 30 min, Ar, rfx; b = toluene, 15 min, Ar, rfx.

In 2019, Zatsikha et al. reported a simple, scalable method for preparing stable meso-(nitrile oxide)-substituted BODIPYs and applying the above-mentioned RCNO for the syntheses of BODIPY-isoxazoline (and isoxazole) derivatives in mild conditions and excellent yields—Scheme 219 [232]. Some BODIPYs possess prominent molecular rotor properties with high sensitivity to the solvent viscosity.

Derivatives of boron dipyrromethene (4,4-difluoro-4-bora-3a,4a-diaza-s-indacene, BODIPY) are characterized by excellent thermal, chemical, and photochemical stability as well as tunable optical properties, lack of ionic charge, and good solubility in organic liquids.

**Scheme 220:**
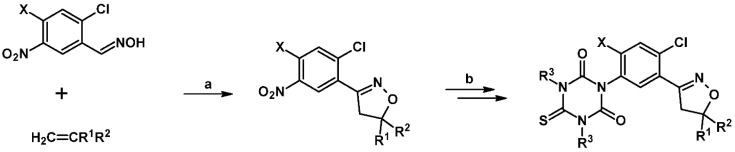
Syntheses of *N*-isoxazolinyl phenyltriazinones as promising protoporphyrinogen IX oxidase inhibitors starting from 1,3-DP cycloaddition [233]. X = F, Cl, Br, H; R^1^, R^2^, R^3^ = Me, CO_2_Et, COOH; a = NCS, DMF, 35 °C, then dipolarophile, Et_3_N, CH_2_Cl_2_, 0 °C; b = next steps.

1,3-DP cycloaddition was an initial step in the synthetic route leading to triazinone-L-isoxazoline-type potential herbicides, when L is a substituted phenyl ring—Scheme 220 [233]. According to Wang and other authors, a scaffold-hopping strategy and systematic SAR-guided structure optimization were crucial in this research.

Moreover, the herbicidal examinations indicated that the derivatives mentioned above are characterized by excellent and broad-spectrum weed control against 32 kinds of tested weeds by postemergence application. Due to these pre-application results, selected compounds equipped with isoxazoline motifs met the expectation as herbicide candidates for weed control in paddy fields.

**Scheme 221:**
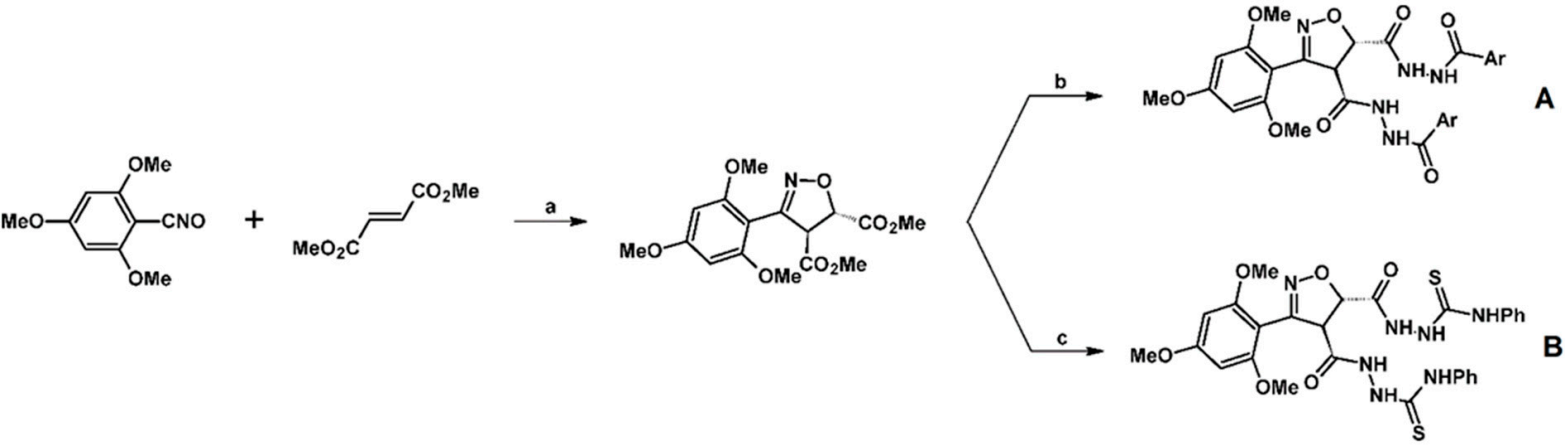
1,3-DP cycloaddition as a crucial step in the synthesis of isoxazoles equipped with 4,5-bis[carbonyl-(4′phenyl)thiosemicarbazide or two 4,5-bis(aroylcarbohydrazide) motifs (the last step, namely aromatization, was omitted) [234]. Ar = Ph, *m*-MeOC_6_H_4_, *o*-O_2_NC_6_H_4_; a = THF, rfx, 6 h; b = NH_2_NH_2_, EtOH, rfx, 24 h, 67% yield, then ArCOCl, K_2_CO_3_, THF, rt, up to 6 h, up to 83% yield; c = NH_2_NH_2_, EtOH, rfx, 24 h, 67% yield; then PhNCS, EtOH, rt, 20 h, 80% yield (final products (**A**,**B**)).

In 2020, Alshamari and coworkers reported synthesizing derivatives of *trans*-3-(2,4,6-trimethoxyphenyl)-4,5-dihydroisoxazole equipped with two 4,5-bis[carbonyl-(4′phenyl)thiosemicarbazide or two 4,5-bis(aroylcarbohydrazide) motifs. They were prepared from trans-3-(2,4,6-trimethoxyphenyl)-4,5-dihydro-4,5-bis(hydrazenocarbonyl)isoxazole—Scheme 221 [234]. The starting 3,4,5-trisubstituted isoxazoline was obtained typically, via 1,3-DP cycloaddition of ArCNO to (*E*)-MeOOCCH=CHCOOMe. Next, the obtained isoxazoline was transformed into final products A and B (equipped with 3,4,5-trisubstituted isoxazoline motif) via two, different synthetic way.

Evaluation of antioxidant and antibacterial activity against particular Gram-positive and Gram-negative bacteria of the obtained isoxazolines indicated that these compounds have suitable scavenging activities. Especially effective was isoxazoline A—see scheme above.

**Scheme 222:**
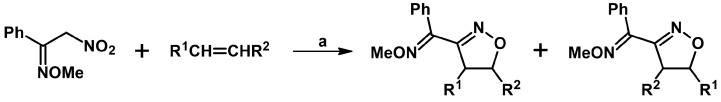
Nitrocompounds as sources of nitrile oxide applied in the synthetic route leading to isoxazolines [150]. R^1^, R^2^ = COOMe, COOMe; Me, COOMe, COOMe, Ph, cyclohexene; a = PhNCO, Et_3_N, THF, rt, 24 h; up to 82% yield.

Nitrile oxides derived from *O*-alkyloxime-substituted nitroalkanes, various (*E*)- or (*Z*)-R^1^CH=CHR^2^-type dipolarophiles, and 1,3-DP cycloaddition were engaged in the syntheses of 3,4,5-trisubstituted 2-isoxazolines—Scheme 222 [150].

Unfortunately, when R^1^ + R^2^, cycloaddition is not regioselective. The synthetic strategy presented above was effective for syntheses of 3,5-di- and 3,5,5-trisubstituted 2-isoxazolines—see Section 2.3 and Section 2.6.

**Scheme 223:**
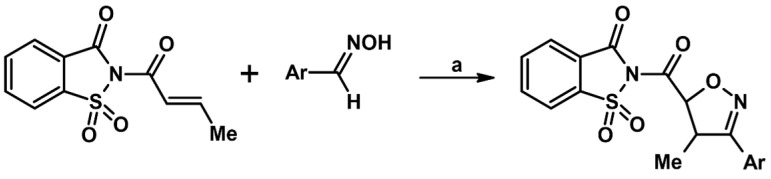
Cycloaddition of aromatic nitrile oxide under non-typical conditions. The procedure has been successfully used for the synthesis of 3,5,5-tri- and 3,4,4,5-tetrasubstituted isoxazolines [149]. Classical conditions: Ar = Ph, *p*-ClC_6_H_4_, *p*-O_2_NC_6_H_4_, 2,4,6-(Me)_3_C_6_H_2_, *p*-Me_2_NC_6_H_4_, and others; a = NaOCl (or NCS), CH_2_Cl_2_, up to rt, MW 103–118 °C, 3 min; up to 96% yield. MW conditions: Ar = *p*-XC_6_H_4_ (X = Cl, O_2_N, MeO, F, Me_2_N, Me), 2,4,6-Me_3_C_6_H_2_; a = CH_2_Cl_2_, 103–118 °C, *p*-HAP300, MW, 3 min; up to 98% yield.

Dipolar cycloaddition activated by MW irradiation increased the yield of dipolar cycloaddition leading to 3,4,5-trisubstituted isoxazolines even more than two times—Scheme 223 [149].

Interestingly, applying MW irradiation in the syntheses of 3,4,5-trisubstituted isoxazolines allowed shortening of the reaction time from more than 20 h to 3 min.

However, it should be remembered that there is no specific MW effect, which has been convincingly proven [235].

**Scheme 224:**
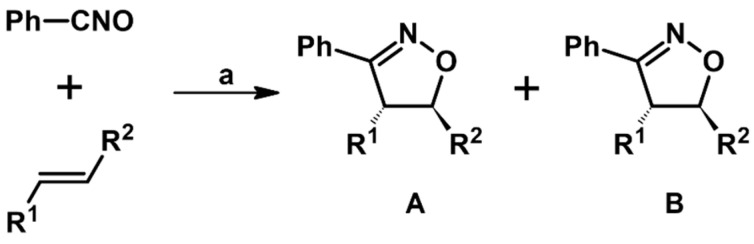
Ionic liquids in the syntheses of isoxazolines [149]. R^1^, R^2^ = Ph, COOEt; Me, COOEt, Et, CH_2_OH; a = rt, MeCN or THF or AcOEt or [BMIM]X where X = [F_3_CSO_2_NSO_2_CF_3_] or [PF_6_] or N_3_, up to 84% yield, stereoisomers ratio (**A**/**B**) = up to 15.

In the same work [149], 3,4,5-trisubstituted 2-isoxazolines were also obtained in ionic liquids, namely in [BMIM]X differing in X—Scheme 224.

However, only in one case did the use of liquid ionic result in greater regioselectivity. In general, the influence of ionic liquids on the chemo-, regio-, and stereoselectivity of dipolar cycloaddition leading to isoxazolines is not simple.

**Scheme 225:**
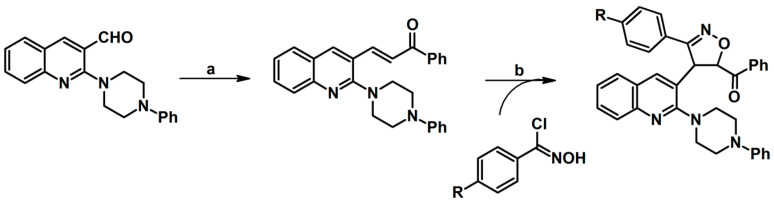
Syntheses of quinoline–isoxazoline hybrids using appropriate reagents and 1,3-DP cycloaddition [236]. R = H, MeO, Cl; a = CH_2_Cl_2_, Et_3_N, rt, PhC(O)CH=PPh_3_, up to 3 h; b = CH_2_Cl_2_, TEA, rt, up to 5 h; 57% yield (for all products).

In 2020, Tang, Peng, and Liu described several quinoline-(9-oxadiazole or isoxazoline or triazolothiadiazole or triazolothiadiazine or piperazine) hybrids which were obtained via 1,3-DP cycloaddition of in situ-generated nitrile oxide (benzenonitrile oxide derivatives) to dienophile generated in situ from quinolyl aldehyde and PhC(O)CH=PPh_3_—Scheme 225 [236].

**Scheme 226:**
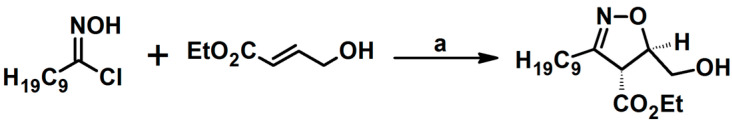
Mediated by *Zn*-(*R*,*R*)-diisopropyl tartrate enantioselective 1,3-DP cycloaddition involved in the total synthesis of mutanobactin D [237]. a = Et_2_Zn, (*R*,*R*)-DIPT, CHCl_3_, 0 °C; 57% yield; *ee* = 96%.

As reported by Pultar et al. in their article from 2021, *Zn*-(*R*,*R)*-DIPT-mediated synthesis of chiral 3,4,5-trisubstituted 2-isoxazoline possessing the appropriate substituent was a crucial step in the total synthesis of a non-ribosomal, cyclic peptide isolated from Streptococcus mutans, namely mutanobactin D—Scheme 226 [237].

The influence of synthetic mutanobactin D on the morphology of *Candida albicans* was assessed in biofilm and filamentation assays, which showed that mutanobactin D could effectively modulate the pathogenic yeast-to-hyphae transition of different *Candida albicans* strains. In addition, mutanobactin D also elicits growth-inhibiting activity against streptococci and other microbial species competing with *Streptococcus mutans* within the oral cavity.

**Scheme 227:**
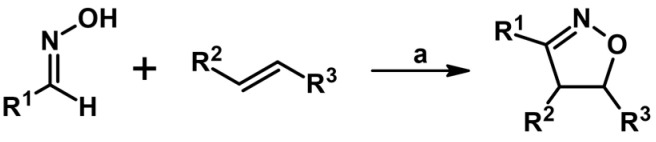
Solvent-free synthesis of isoxazolines via 1,3-DP cycloaddition of RCNO to carbon–carbon double bond in mechanochemical (ball-milling) conditions [238]. R^1^, R^2^, R^3^ = *p*-MeC_6_H_4_, H, CO_2_tBu; *p*-MeC_6_H_4_, H, CN; *p*-MeC_6_H_4_, SiMe_3_; CO_2_Me, CO_2_Me; CO, *p*-MeC_6_H_4_, CO_2_Me, CO_2_Me; 2-Thienyl, H, CO_2_Me; *p*-MeC_6_H_4_, Ph, COC_6_H_4_; and others; a = NaCl, Oxone, Na_2_CO_3_, 30 Hz, 1 h, rt, milling conditions; up to 86% yield.

3,5-di- and 3,4,5-trisubstituted-2-isoxazolines were obtained via 1,3-DP cycloaddition realized under ball-milling conditions—Scheme 227 [238].

#### 2.5.1. Syntheses of 3,4,5-Trisubstituted 2-Isoxazolines from Qallyl and RCNO Involving Double Bond Migration, Metathesis, and Dipolar Cycloaddition in Various Sequences

This part is devoted to obtaining 3,4,5-isoxazolines starting from Qallyl substrate and involving double bond migration, metathesis, and 1,3-DP cycloaddition of aromatic nitrile oxides to the products of the above-mentioned allyl substrate transformation. Importantly, typical, i.e., thermal or high-pressure conditions, were used in these reactions [33,201,239,240,241,242,243,244,245].

##### Syntheses of 3,4,5-Trisubstituted 2-Isoxazolines from Qallyl via Qallyl Izomerization to Q(1-Propenyl) Derivatives followed by 1,3-DP Cycloaddition of RCNO into Q(1-PROPENYL) Compounds

**Scheme 228:**
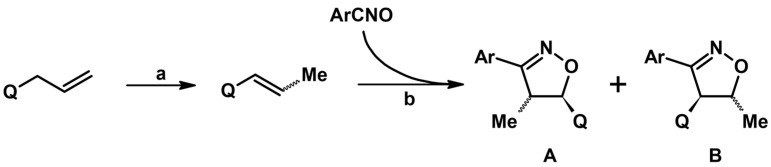
Application of double bond migration and 1,3-DP cycloaddition of RCNO to carbon–carbon double bond starting from Qallyl compounds. a = [RuClH(CO)(PPh_3_)_3_] or [RhH(CO(PPh_3_)_3_] or *t*-BuOK/18-crown-6 or Verkade’s super base; b = Et_3_N, immobilized amine, THF or CH_2_Cl_2_, up to 24 h, up to rfx; up to 95% yield. Ar, Q, (**A**/**B**) = *p*-MeOC_6_H_4_, PhO, 100% A; *p*-ClC_6_H_4_, PhO, 100%A; *o*-BrC_6_H_4_, PhO, 100%A; 2,6-Cl_2_C_6_H_3_, menthylO, 100%A; 2,4,6-Me_3_C_6_H_2_, Ph_3_COO, 100%A; 2,4,6-Me_3_C_6_H_2_, 9-anthracenyl, 100%A; 2,4,6-Me_3_C_6_H_2_, PhO, 100%A; 2,6-Cl_2_C_6_H_4_, (*i*-Pr)_2_N, 100%A; 2,6-Cl_2_C_6_H_3_, Ph_3_Si,31/69; 2,6-Cl_2_C_6_H_3_, 9-carbazolyl,56/44; 2,6-Cl_2_C_6_H_3_, H_2_NCONH, 100%A; 2,6-Cl_2_C_6_H_3_, Me_2_, 100%A; 2,6-Cl_2_C_6_H_3_, *p*-MeC_6_H_4_CH=N,100%A; 2,6-Cl_2_C_6_H_3_, (2-furyl)CH=N, 100%A; 2,6-Cl_2_C_6_H_3_, PhSe, 66/34; 2,6-Cl_2_C_6_H_3_, *N*-imidazolyl, 100%A and others (more than 50 examples) [33,201,239,240,241,242,243,244,245].

Several 3,4,5-trisubstituted isoxazolines were efficiently obtained starting from 3-C-, 3-*O*, 3-*S*-, 3-*P*-, and 3-*N*-allyl compounds of the QCH_2_CH=CH_2_-type, which underwent double bond migration to QCH=CHCH_3_, followed by dipolar cycloaddition—see Scheme 228 [33,201,239,240,241,242,243,244,245].

Importantly, 1,3-DP was fully regioselective for *O*- and *N*-(1-propenyl) compounds—only A isoxazolines were obtained. Moreover, a complete transfer of dienophile configuration into cycloadducts was observed, namely (*Z*)-dienophiles were transformed into *cis*-isoxazolines, but (*E*)-dienophiles into *trans*-isoxazolines. Such an observation explicitly indicated that 1,3-dipolar cycloaddition in the above-mentioned reactions proceeded via a concerted mechanism. Further discussion of the structure and reactivity relationship and 1,3-DP cycloaddition mechanism was presented in Section 3.

##### Synthesis of 3,4,5-Trisubstituted 2-Isoxazolines from Qallyl by the following Reaction Sequences: Double Bond Migration–Self-Metathesis-1,3-DP Cycloaddition

**Scheme 229:**
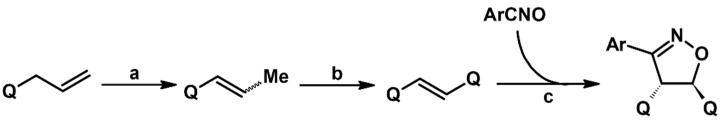
Syntheses of 3,4,5-triarylsubstituted isoxazolines from Qallyl and involving double bond migration–self metathesis–nitrile oxide cycloaddition reaction sequences [33,201,239,240,241,243,244,245]. Ar, Q = 2,6-Cl_2_C_6_H_3_, 3,4-methylenedioxyphenyl; 2,4,6-Me_3_C_6_H_2_, 3,4-methelenedioxyphenyl; 2,4,6-(MeO)_3_C_6_H_2_, 3,4-methylenedioxyphenyl; 9-anthracenyl, Ph. a = [RuClH(CO)(PPh_3_)_3_], solvent; b = CH_2_Cl_2_ or DMF, 40 °C or 100 °C, Hoveyda–Grubbs II generation catalyst; c = various conditions (depending on the Ar and Q); up to 50% overall yield.

3,4,5-trisubstituted 2-isoxazolines were obtained from Qallyl by the following reactions sequences: double bond migration in Qallyl to QCH=CHMe, then self-metathesis of QCH=CHMe to QCH=CHQ, and finally ArCNO 1,3-DP cycloaddition to metathesis products—Scheme 229 [33,201,239,240,241,243,244,245].

Thanks to *E*-stereoselective self-metathesis, only *trans*-isoxazolines were obtained. That strongly indicates a concerted one-step course of the 1,3-DP cycloaddition.

##### Syntheses of 3,4,5-Trisubstituted 2-Isoxazolines via Qallyl Self-Metathesis to QCH_2_CH=CHCH_2_Q Followed by RCNO Cycloaddition

**Scheme 230:**
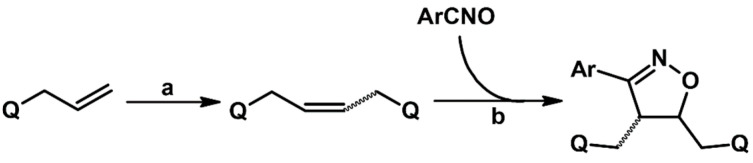
Self-metathesis of Qallyl to QCH_2_CH=CHCH_2_Q followed by DPC involved in the syntheses of trisubstituted isoxazolines [34,241,243,244,245]. Ar, Q = 2,6-Cl_2_C_6_H_3_, (NPh(COMe), *n*-BuO, PhO, *n*-PrC(O)O, Me_3_C, *N*-phthaloimidoyl, *n*-decyl*O*, PhC(*O*)*O*); 2,4,6-Me_3_C_6_H_2_, *N*-phthaloimidoyl; 2,4,6-(Me*O*)_3_C_6_H_2_, *N*-phthaloimidoyl. a = Grubbs II catalyst, CH_2_Cl_2_ or DMF, 40 °C or 100 °C, up to 96 h; b = see scheme above.

The strategy presented below, consisting of combining Qallyl self-metathesis with dipolar cycloaddition of nitrile oxide to the metathetically obtained dipolarophiles, allowed the acquisition of a series of 3,4,5-trisubstituted isoxazolines—Scheme 230 [33,241,243,244,245].

In the case of 3-*N*-substituted allyl substrates, only *E*-metathesis products were obtained, and consequently, fully stereoselective 1,3-DP cycloaddition led to only *trans*-isoxazolines. This 100% agreement between dipolarophiles and cycloadduct configuration indicates a concerted nature of cycloaddition.

##### 3,4,5-Trisubstituted 2-Isoxazolines from Qallyl and the following Reaction Sequences: Qallyl Self-Metathesis, Double Bond in Self-Metathesis Products, Dipolar Cycloaddition

**Scheme 231:**
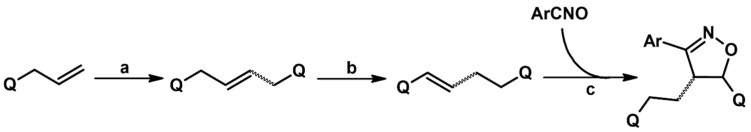
Syntheses of trisubstituted isoxazolines from Qallyl and with involving self-metathesis–isomerization–cycloaddition sequences [33,241,243,244,245]. Ar, Q = 2,6-Cl_2_C_6_H_3_, C_6_H_4_ N(COMe); 2,6-Cl_2_C_6_H_3_, *n*-BuO; 2,4,6-Me_3_C_6_H_2_, *n*-BuO; 2,4,6-(MeO)_3_C_6_H_2_, *n*-BuO; 2,6-Cl_2_C_6_H_3_, C_6_H_4_O; 2,6-Cl_2_C_6_H_3_, *n*-C_10_H_21_; a = Hoveyda–Grubbs II gen.; b = [RuClH(CO)(PPh_3_)_3_]; c = various conditions depending on Q and Ar.

In this synthetic approach, Qallyl was subjected to self-metathesis to QCH_2_CH=CHCH_2_Q, which was transformed via isomerization to QCH=CHCH_2_CH_2_Q, and the latter was finally subjected to cycloaddition—Scheme 231 [33,241,243,244,245]. Notably, the 1,3-DP cycloaddition was fully regioselective.

##### Syntheses of 3,4,5-Trisubstituted 2-Isoxazolines Starting from Allyl Substrate of the QCH_2_CH=CHCH_2_Q Type Obtained from XCH_2_CH=CHCH_2_X

**Scheme 232:**
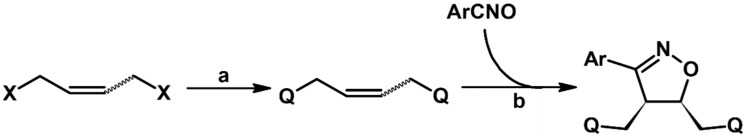
Syntheses of *cis*-3,4,5-trisubstituted isoxazolines via 1,3-DP cycloaddition of 2,6-Cl_2_C_6_H_3_CNO to 1,4-disubstituted (*Z*)-but-2-ene derivatives (*Z*)QCH_2_CH=CHCH_2_Q [33,241,244]. a = typical alkylation or acylation in the PTC conditions; X = OH or Cl; Q = *n*-BuO, *n*-C_10_H_21_O, *n*-PrC(O)O, C_6_H_4_C(O)O; b = 2,6-Cl_2_C_6_H_3_CNO, CH_2_Cl_2_, 40 °C, 24 h; up to 76% yield.

Only *cis*-3,4,5-trisubstituted isoxazolines were obtained by cycloaddition reaction of 1,3-dipolar disubstituted derivatives of (*Z*)-but-2-ene to 2,6-dichlorobenzonitrile oxide—Scheme 232 [33,241,244]. Dipolarophile was obtained from commercially available XCH_2_CH=CHCH_2_X using classical methods of organic synthesis.

Importantly, in the case of the 1,3-DP cycloaddition shown in Scheme 232, the observed complete transfer of the dipolarophile into isoxazolines configuration indicates the agreed nature of the cycloaddition.

##### Syntheses of 3,4,5-Trisubstituted 2-Isoxazolines Using High-Pressure Conditions

**Figure 7:**
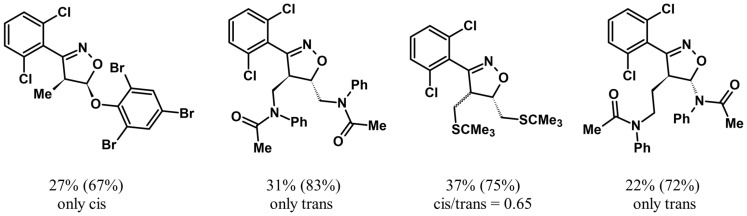
Isoxazolines obtained under high-pressure conditions (100 °C, 1.8 GPa, CH_2_Cl_2_, 24 h) and in a closed reactor (100 °C, up to 2–4 atm, CH_2_Cl_2_, 24 h) [33,241,245].

Cycloaddition reactions, particularly the Diels–Alder reaction and dipolar cycloaddition, are characterized by strongly negative activation volume, which is well-known [246,247]. This means that carrying out the reaction under high pressure (1 GPa or higher) allows for a significant increase in conversion, lowering the temperature and using strongly sterically crowded reagents. Interestingly, no effect of high pressure on the regio- and stereoselectivity of the reaction was observed [33,241,245]. The reactions and structures of isoxazolines obtained by the high-pressure method are shown below—Figure 7. Yields are also given—at equilibrium pressure (i.e., in a closed reactor) and high pressure (in parentheses).

It is easy to notice that applying high-pressure conditions resulted in spectacularly increased yields of all cycloadditions, especially in the case of reaction with phthalodinitrile dioxide.

### 2.6. 3,5,5-Trisubstituted 2-Isoxazolines

**Scheme 233:**
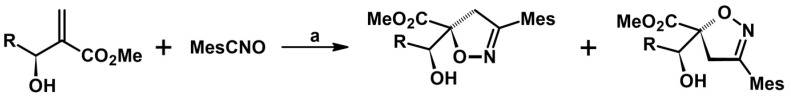
Syntheses of trisubstituted isoxazolines: influence of *Mg*-coordination on diastereoselectivity (an example) [238]. R = *i*-Pr; Mes = 2,4,6-(Me)_3_C_6_H_2_; a = PhMe, 2 h, 80 °C, 99% yield, A/B = <5/>95; CH_2_Cl_2_, MeMgBr, 101 × 21 s, from −20 to 0 °C, 59% yield, A/B = >95/<5.

In 2000, the preparation of 3,5,5-trisubstituted isoxazolines via 1,3-DP cycloadditions of mesitonitrile oxide to ß-hydroxy-α-methylene esters in various conditions was described by Mičúch and coworkers—Scheme 233 [248].

Interestingly, in the presence of the Grignard reagent, the diastereoselectivity of the cycloaddition is completely reversed due to the specific interactions between Mg^2+^ and dipolarophile and dipoles.

**Scheme 234:**
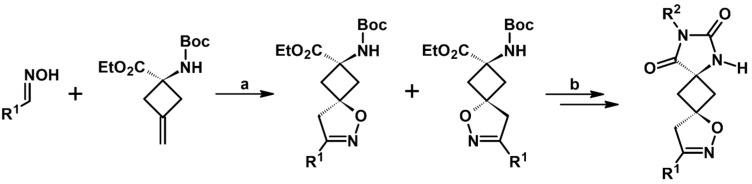
Synthesis of hydantoin- and 3,5,5-trisubstituted 2-isoxazoline-based pharmacophores [249]. The second regioisomer was also obtained; after separation, only the one presented on the scheme was transformed into the final product. R^1^, R^2^ = *p*-BrC_6_H_4_, Ph; *n*-Bu, Ph; Ph, Bn; *p*-BrC_6_H_4_, *p*-ClC_6_H_4_ and others; a = NaOCl, CH_2_Cl_2_, Et_3_N, from 0 °C to rt; b = next steps.

Another article from that year presented 1,3-DP cycloaddition involved in the synthetic method leading to the isoxazoline–hydantoin hybrids containing a cyclobutane motif as a spiro-bridge between heterocyclic motifs—Scheme 234 [249].

**Scheme 235:**
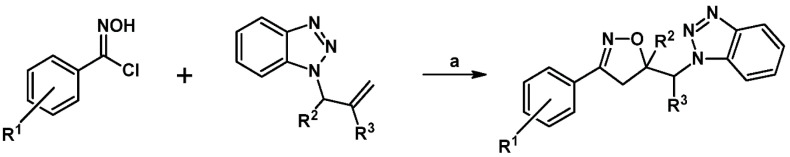
Syntheses of 3,5-di- and 3,5,5-trisubstituted isoxazolines equipped with –CH_2_-benzotriazol motif via 1,3-DP cycloaddition of in situ-generated R^1^PhCNO to N-allyl benzotriazoles [250]. R^1^, R^2^, R^3^ (%yield) = H, Br, H (13); p-MeO, H, H (94); p-Me, H, H (89); p-MeO, H, EtO (80); a = Et_3_N or KHCO_3_, CH_2_Cl_2_ or AcOEt or THF.

The same year, another group of researchers reported 1,3-DP cycloaddition applied in the syntheses of isoxazolines bearing a benzotriazol-1-yl moiety tethered via CH_2_ linker to the carbon atom (C-5)—Scheme 235 [250].

**Scheme 236:**
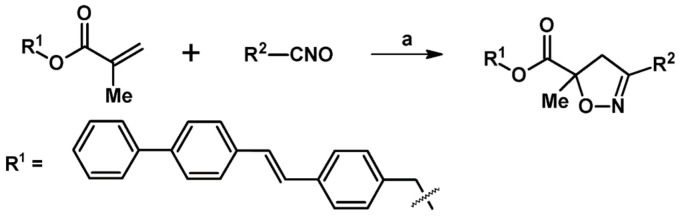
Syntheses of isoxazolines equipped with the (*E*)-stilbene motif [16]. R^2^ = *t*-Bu, Ph, *p*-O_2_NC_6_H_4_; a = Et_3_N, CH_2_Cl_2_, 0 °C; up to 87% yield.

In work cited in Section 2.3, Bosanac, Yang, and Wilcox also described 3,5,5-trisubstituted 2-isoxazolines possessing a Ph-substituted (*E*)-stilbene motif (joined through –CH_2_OC(O)– linker) in position 5, which were obtained via 1,3-DP cycloaddition of the appropriate nitrile oxide to methacrylate equipped with the above-mentioned stilbene fragment—Scheme 236 [16].

**Scheme 237:**
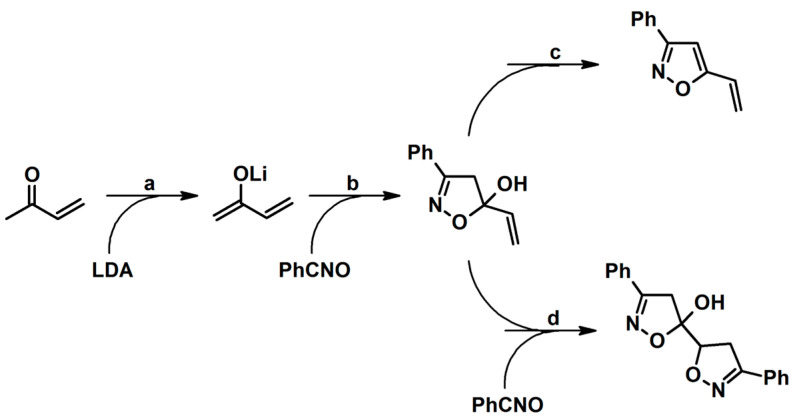
Synthesis and further transformation of vinylisoxazoline [251]. a = THF, −78 °C; b = THF, rt, 3 h, 70% yield; c = BF_3_-Et_2_O, quant.; d = THF, rt, 3 h, 70% yield.

In 2005, Di Nunno, Scilimati, and Vitale reported 1,3-DP cycloaddition of PhCNO to lithium enolate derived from methyl-vinyl ketone, which resulted from 5-hydroxy-3-phenyl-5-vinyl-2-isoxazoline—Scheme 237 [251].

The obtained vinylisoxazoline can be smoothly transformed into 5-vinylisoxazole (quantitatively) or bis-isoxazoline (via the next DP cycloaddition).

**Scheme 238:**
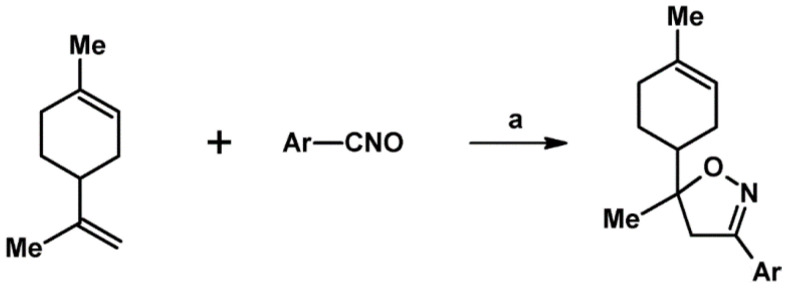
Syntheses of limonene motif contained 2-isoxazolines [252]. Ar = Mes, p-ClC_6_H_4_, m-O_2_NC_6_H_4_; a = CH_2_Cl_2_, rt, 3 days; up to 85% yield.

In 2006, Feddouli and colleagues presented an efficient approach to synthesizing 3,5,5-trisubstituted isoxazolines equipped with the limonene motif in positions 5,5 via 1,3-dipolar cycloaddition of nitrile oxide to limonene—Scheme 238 [252].

**Scheme 239:**
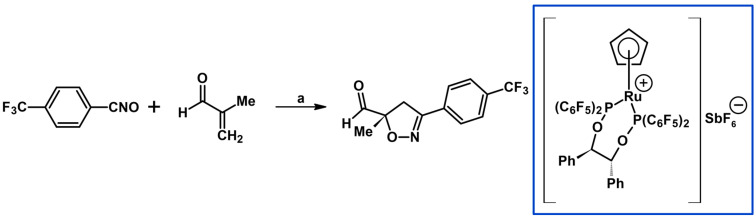
Synthesis of chiral isoxazoline mediated by chiral ruthenium complex [253]. a = [RuCp(*R*,*R*-(F_5_Ph)_2_OCH(Ph)CH(Ph)OP(PhF_5_)_2_)]SbF_6_, CH_2_Cl_2_, −20 °C, 16 h; 60% yield; *ee* = 93%.

A cationic ruthenium complex with chiral diphosphines was an effective catalyst for obtaining chiral 3,5,5-trisubstituted isoxazoline via cycloaddition of *p*-XC_6_H_4_CNO (the best result was reached for *p*-F_3_CC_6_H_4_CNO) to metacrolein—Scheme 239 [253]. For other substituents X (Me, MeO), yield and *ee* were significantly lower.

Importantly, the stereochemistry of the major enantiomer is *S*, consistent with an approach of the nitrile oxide to the C_α_-*Si* face of the dipolarophile in the anti-s-trans conformation in the *Ru*-complex site. Moreover, the presence of EWG in ArCNO was crucial for obtaining high ee using [Ru(acetone)(*R*,*R*)-(BIPHOP-F)Cp][SbF_6_].

**Scheme 240:**
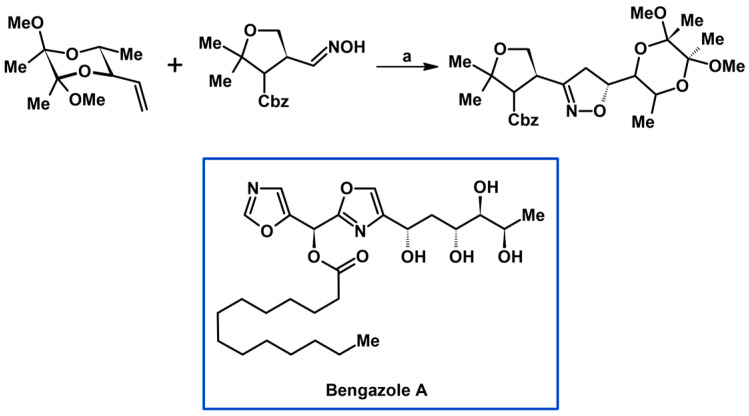
DP cycloaddition as one of the steps in the total syntheses of bengazole A—selected example [54]. a = NaOCl aq., Et_3_N, CH_2_Cl_2_, slow addition of oxime, 0 °C; 86% yield (*dr* = 1:1).

As presented by Bull and colleagues in 2007, 1,3-DP cycloaddition between specially designed nitrile oxide and dipolarophiles was an essential step in the stereocontrolled multistep synthetic methods leading to the various bengazoles (i.e., bengazole A)—Scheme 240 [54].

Bengazoles belong to a family of marine-sourced natural products that display potent antifungal activity.

**Scheme 241:**
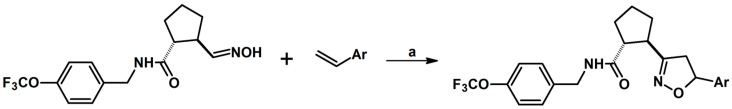
Specially substituted isoxazolines: new voltage-gated sodium channel blocker [254]. Ar = H, Ph; H, *p*-ClC_6_H_4_; H, *p*-CF_3_OC_6_H_4_; H, 2-pyridyl and others; a = CH_2_Cl_2_, NCS, 25 °C, 1 h then Et_3_N, 16 h, yield not given.

Two years later, a group of researchers presented 1,3-DP cycloaddition of specially designed nitrile oxide to vinyl arenes as a crucial step in synthesizing potential voltage-gated sodium channel blockers—Scheme 241 [254].

Significantly, modification of both substituents in the cyclopentane system, namely the amido motif and Ar group, resulted in a significant loss of sodium-channel-blocking activity, while non-aromatic heterocycle replacements were well-tolerated. Moreover, SAR studies showed that lipophilic substitutions on phenyl and isoxazoline rings were well-tolerated, but not polar substitutions.

**Scheme 242:**
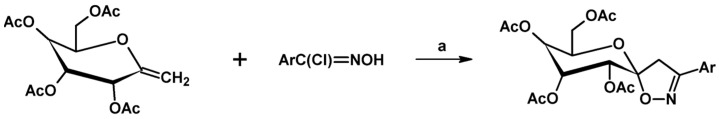
Syntheses of 3,5,5-trisubstituted 2-isoxazolines equipped with glucose motif attached via spiro motif [255,256]. Ar = Ph, *p*-MeOC_6_H_4_, *p*-MeC_6_H_4_, *p*-O_2_NC_6_H_4_, 2-naphthyl. a = ArC(Cl)=NOH, Et_3_N, CH_2_Cl_2_, rt, 16 h; 83–99% yield.

As stated in reports from Benltifa and coworkers, glucose-based spiro-isoxazolines belonging to the 3,5,5-trisubstituted 2-isoxazolines were obtained via fully regio- and stereoselective 1,3-DP cycloaddition of ArCNO to the methylene acetylated exo-glucal—Scheme 242 [255,256].

After deacetylation, the obtained isoxazolines were evaluated as inhibitors of muscle glycogen phosphorylase (GPb), including docking calculations with GLIDE in extra-precision (XP) [255].

**Figure 8:**
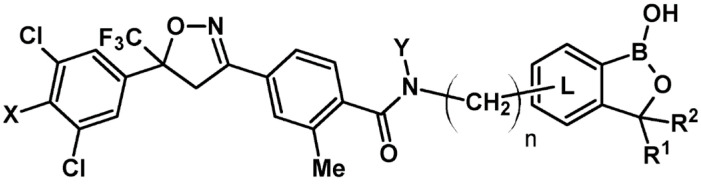
The general formula of the 3,5,5-trisubstituten isoxazolines described in the invention [257,258]. X = H, Cl or F; Y = H or Me (only for one example); L (link) to 5th or 6th carbon atom; n = 1 or 2; R^1^ = R^2^ = H or Me (in the majority of cases).

As first covered in a US patent in 2013 and three years later in an article, many 3,5,5-trisubstituted isoxazolines were designed as candidates for an oral long-acting animal ectoparasiticide. Zhang and teammates reported their preparation via typical 1,3-DP cycloaddition of RCNO to appropriate dipolarophiles and various further chemical transformations—Figure 8 [257,258].

An evaluation of activity against ticks and fleas in a rat model (for 23 isoxazolines) allowed researchers to identify active and orally bioavailable molecules.

**Scheme 243:**
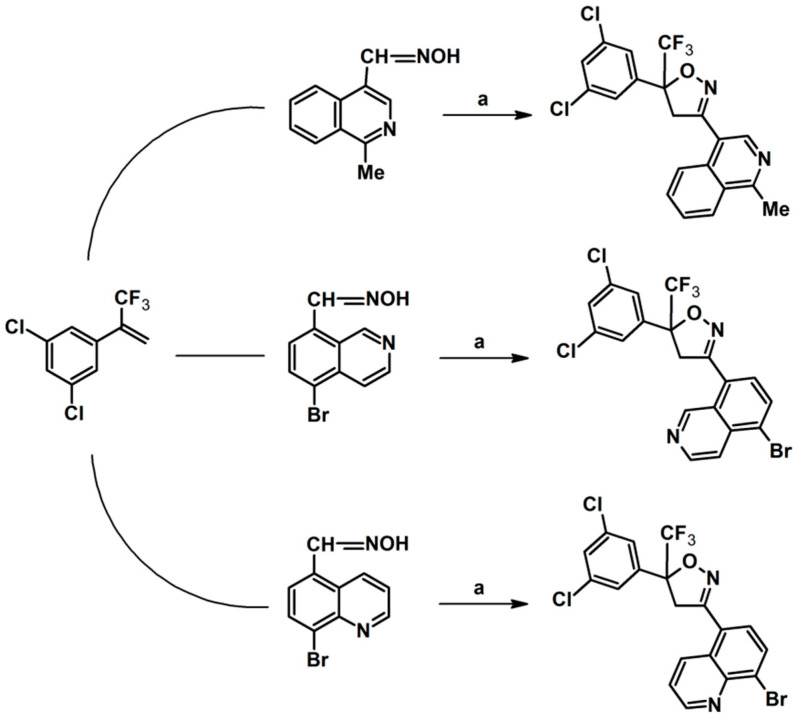
Syntheses of 3,5,5-trisubstituted isoxazolines using specially designed nitrile oxides and dipolarophile [259]. Further modifications: Me and Br substituents were transformed into C(O)NHR group. a = NCS, Et_3_N, CH_2_Cl_2_; 50–61% yields.

1,3-DP cycloaddition was a crucial step in the synthetic route presented by Xu et al. leading to 3,5,5-disubstituted isoxazolines bearing a CF_3_ motif in position 5 and a quinolinyl or isoquinolinyl motif in position 3—Scheme 243 [259].

After further modification, transforming Me and Br substituents into a C(O)NHR motif, the obtained modified isoxazolines were tested for their insecticidal properties. Interestingly, isoxazoline equipped with an isoquinoline motif (with nitrogen atom at the 7 position, and R = cyclopropylmethyl) has an excellent insecticidal activity—higher than the commercial insecticide Indoxacarb.

**Scheme 244:**
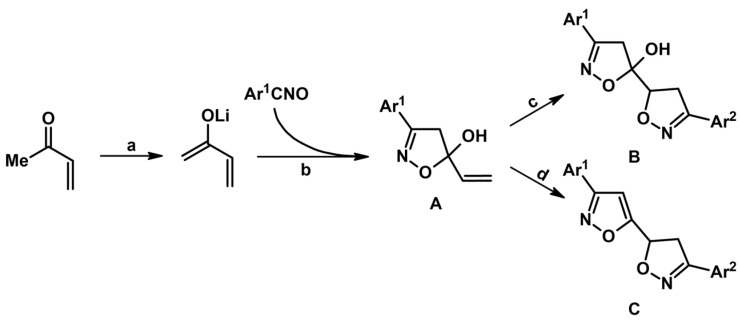
Syntheses of hydroxy-vinyl isoxazolines, bis-isoxazolines, and isoxazoline–isoxazole hybrids using ArCNO and methyl-vinyl ketone lithium enolate [260]. Ar^1^, Ar^2^ = 3-O_2_N, Ph; 5-Cl-furan-2-yl, Ph; *o*-ClC_6_H_4_, *o*-ClC_6_H_4_; a = LDA, −78 °C; b = THF, rt; up to 90% yield (**A**); c = Ar^2^CNO, THF; up to 96% yield (**B**); d = BF_3_-OEt_2_, CH_2_Cl_2_, rt, then Ar^2^CNO, THF; up to 74% (**C**).

As a continuation of Di Nunno et al.’s work [251], the acquisition of 3-aryl-5-hydroxy-5-vinyl-2-isoxazolines (A) through the 1,3-DP cycloaddition of ArCNO to the lithium enolate of methyl vinyl ketone was reported ten years later—Scheme 244 [260]. According to the authors, one species (A) can be easily transformed into bis-isoxazolines (B) and isoxazoline–isoxazole hybrids (C).

**Scheme 245:**
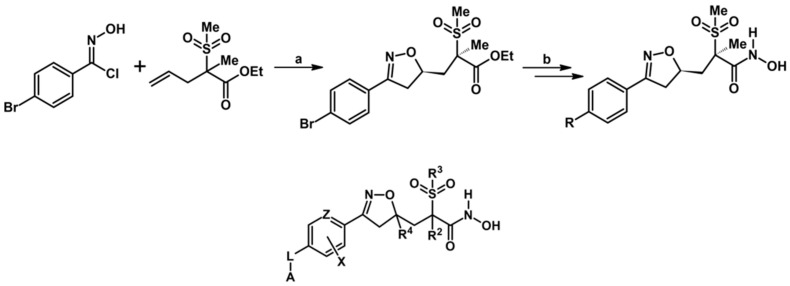
Isoxazolines for medicinal applications—general formula and synthetic procedure for one example. Z, X, L, A, R, R^2^–R^4^ are defined in the invention [112]. a = ZnEt_2_, (*R*,*R*)-DIPT, *t*-BuOCl, CHCl_3_, 1,4-dioxane, 0 °C to rt, 4 h; 62% yield (DP cycloaddition); b = next steps.

An invention already mentioned in Section 2.3 was dedicated to treating bacterial infections with 3,5- and 3,5,5-trisubstituted 2-isoxazolines. The latter were obtained using 1,3-DP cycloaddition of RCNO to the carbon–carbon double bond—see Scheme 245 [112]. This scheme presents the general formula of manufactured isoxazolines and selected synthesis.

**Scheme 246:**
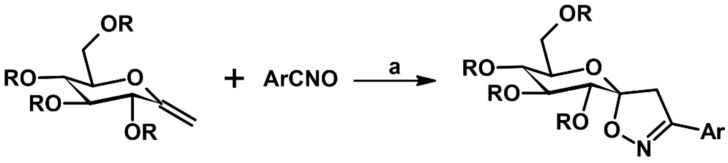
Syntheses of 3,5,5-trisubstituted 2-isoxazolines possessing glucose motif attached via spiro-mode in position 5,5 [261]. R = Ac or Bz; Ar = Ph, *p*-MeOC_6_H_4_, *p*-O_2_NC_6_H_4_, 2-naphthyl, 3,4-methylenedioxyphenyl, 9-phenanthrenyl, indol-2-yl, and others; a = method 1: exo-glucal, oxymoyl chloride, CH_2_Cl_2_, overnight; method 2: exo-glucal, aldoxime, THF, overnight, NaOCl_(aq)_; up to 99% yield.

In 2016, Goyard and colleagues reported that D-glucopyranosylidene-spiro-isoxazolines can be prepared from *O*-peracylated exo-D-glucals by regio- and stereoselective 1,3-DP cycloaddition of in situ-generated ArCNO by treatment of the corresponding oximes with bleach—Scheme 246 [261].

After the deprotection of hydroxyl groups, the obtained deprotected glucopyranosylidene-spiro-isoxazolines were evaluated as potent GP inhibitors. Interestingly, they exhibited IC 50 values ranging from 1 to 800 mM. The 2-naphthyl substituted glucopyranosylidene-spiro-isoxazoline was the most active. Namely, it lowered glucose blood levels by nearly 33% at 30 mg/kg. Such results confirmed that glucose-based spiro-isoxazolines could be considered as anti-hyperglycemic agents in the context of type 2 diabetes.

**Scheme 247:**
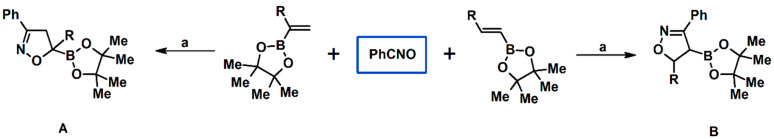
Fully regioselective 1,3-DP cycloaddition of PhCNO to alkenyl boronic esters [128]. R, (**A**:**B**) = H, 100:0; Me, 100:0; *n*-Bu, 0:100; CO_2_Me, 0:100; a = Et_3_N, THF, rt, 3 h; 85–97% yield.

As described in their article (also already mentioned in Section 2.3), Jeong, Zong, and Choe performed 1,3-DP cycloaddition of PhCNO to monosubstituted or 1,1-disubstituted alkenyl boronic esters. This attempt resulted in only 3,5,5- or 3,4,5-trisubstituted-2-isoxazolines, having the boronic ester group at the 5-position of the ring. On the other hand, the same reaction with *trans*-1,2-disubstituted alkenyl boronic esters as dipolarophiles gave 2-isoxazolines, bearing the boronic ester moiety at the 4-position of the ring—Scheme 247 [128].

Interestingly, DFT calculations revealed that the experimental results agreed well with the parameters based on the transition state energies in gas or solvent phase.

**Scheme 248:**
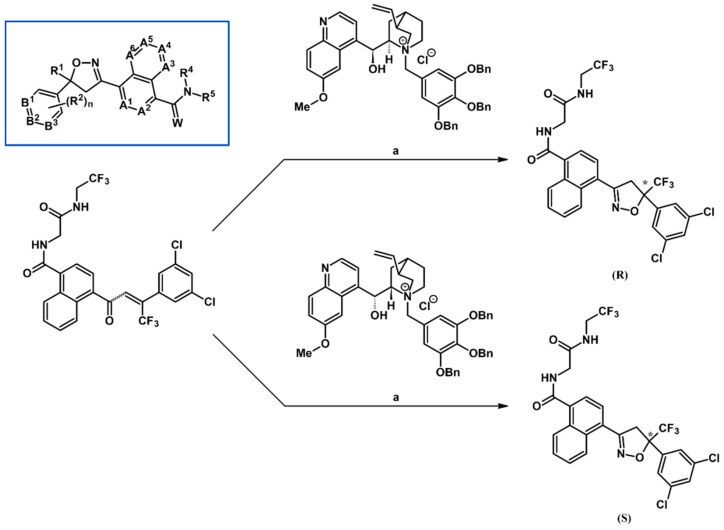
Syntheses of chiral 3,5,5-trisubstituted 2-isoxazolines: general formula of obtained compounds and example of the synthetic procedure [262,263]. a = NH_2_OH, NaOH, CH_2_Cl_2_, −10 °C, 16 h, 3% yield (*R* and *S*).

In the US invention from 2017 (and also in WO patent), the preparation of isoxazolines enriched in an enantiomer using quinine-based chiral phase transfer catalyst antiparasitic was described—Scheme 248 [262,263]. This synthetic method belongs to the intramolecular 1,3-DP cycloaddition of the CNO motif to the double bond, both coming from the same molecule.

Importantly, the syntheses of quinine-based phase transfer catalysts used for asymmetric induction are also presented in this invention.

**Scheme 249:**
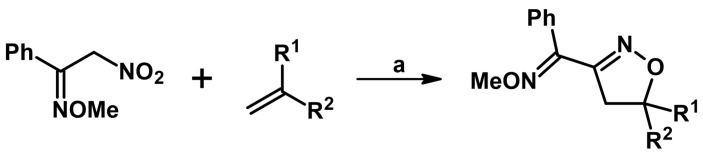
Nitro compounds as sources of nitrile oxide applied in the synthetic route leading to isoxazolines [150]. R^1^, R^2^ = Me, Ph; CF_3_, Ph; F, CO_2_Me; norbornene; a = PhNCO, Et_3_N, THF, rt, 24 h; up to 89% yield.

Nitrile oxides derived from *O*-alkyloxime-substituted nitroalkanes, various CH_2_=CHR^1^R^2^-type dipolarophiles, and 1,3-DP cycloaddition (regioselective) were engaged in the syntheses of 3,5,5-trisubstituted 2-isoxazolines—Scheme 249 [150].

The synthetic strategy presented above turned out to be effective for syntheses of 3,5-di- and 3,4,5-trisubstituted 2-isoxazolines—see Section 2.3 and Section 2.5.

**Scheme 250:**
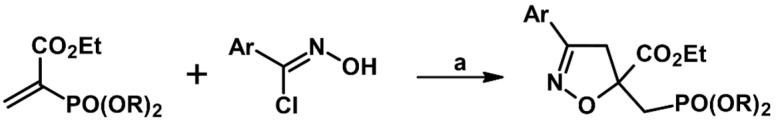
Syntheses of trisubstituted 2-isoxazolines equipped with P(O)(OEt)_2_ motif via DP cycloaddition of in situ-generated nitrile oxide to ethoxycarbonyl allylphosphonates [264].] R = Et, Me; Ar = Ph, *p*-MeC_6_H_4_, 2,3-(MeO)_2_C_6_H_3_; a = Et_3_N, CHCl_3_, rt, 18 h; up to 82% yield.

In another 2020 article, Abdelli et al. described regioselective cycloaddition of in situ-generated (from oxymoyl chloride) nitrile oxide to 2-ethoxycarbonyl allyl phosphonates leading to 3,5,5-trisubstituted 2-isoxazolines—Scheme 250 [264].

According to the authors, the steric effect is mainly responsible for the strong cycloaddition regioselectivity. Antibacterial activity against several bacterial species tested for select obtained isoxazolines revealed that some are characterized by antibacterial activity similar to the referring gentamicin. Interestingly, the presence of the P(O)(OEt)_2_ motif in the oxazolines was crucial for their specific activity against *Staphylococus aureus* Gram-positive bacteria.

**Scheme 251:**
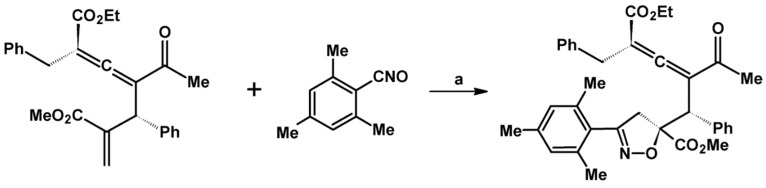
Synthesis of chiral 3,5,5-trisubstituted 2-isoxazolines possessing allenyl moiety [265]. a = PhMe, rt, 6 h; 94% yield; *dr* > 20:1; *ee* = 90%.

Hu and colleagues applied and described dipolar cycloaddition of stable MesNCO to chiral allenoate to synthesize chiral 3,5,5-trisubstituted 2-isoxazolines in the same year—Scheme 251 [265].

**Scheme 252:**
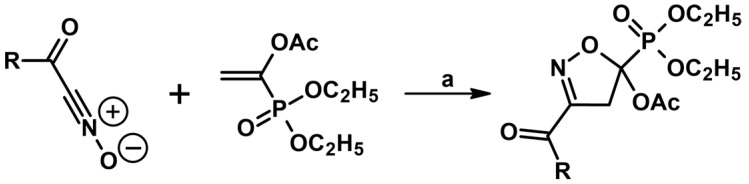
Dipolar cycloaddition of acyl nitrile oxide to the vinyl acetate derivative equipped with P(O)(OEt)_2_ moiety [266]. a = CAN, acetone or acetophenone, rfx, 10 h, yield not given.

In the Chinese patent from 2020, the syntheses of 2-isoxazolines equipped with a P(O)(OEt_2_)_2_ group in position 5 were presented—Scheme 252 [266].

**Figure 9:**
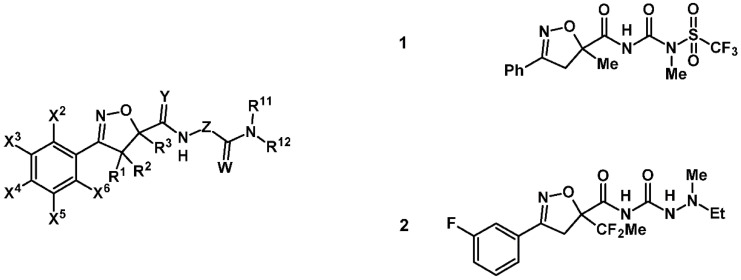
General formula (and selected examples—1 and 2) of 3,5,5-trisubstituted isoxazolines used in plant protection [267].

The invention from 2020 is devoted to obtaining and using 3-phenylisoxazoline-5-carboxamides of tetrahydro- and dihydrofuran carboxamides. The general formula is presented below, and obtaining those structures was carried out typically via 1,3-DP cycloaddition of RCNO to the double bond followed by further functionalization—Figure 9 [267].

**Figure 10:**
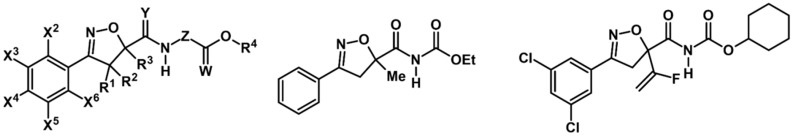
3,5,5-trisubstituted isoxazolines (esters or salts) attractive in the crop protection sector [268].

In the invention from 2021, structurally similar to the isoxazolines presented above (in Figure 9), 3-phenylisoxazoline-5-carboxamides of tetrahydro- and dihydrofurancarboxylic acids in salt or ester form, having the general formula as in Figure 10, were described [268].

**Scheme 253:**
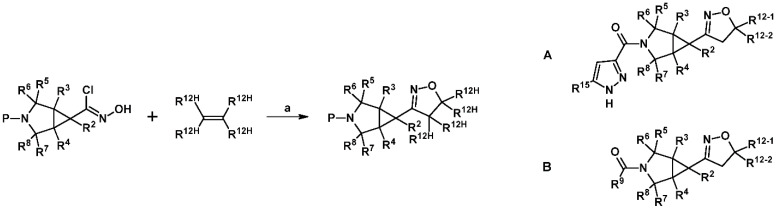
3,5,5-trisubstituted isoxazolines obtained via typical 1,3-DP cycloaddition of in situ-generated RCNO into R^1^R^2^C=CR^3^R^3^ dipolarophiles as useful prophylactic and therapeutic agents [269]. P and R^n^ are defined in the invention; a = base (e.g., Et_3_N or DIPEA), solvent (e.g., DMF), from 0 °C to 80 °C; up to 79% yield, (**A**,**B**) = structures of product with defined P moiety.

Another patent from 2021 described compounds with general formulas presented in Scheme 253 and useful as prophylactic or therapeutic agents for cancer, Huntington’s, and Alzheimer’s disease [269]. Importantly, the selected invented compounds belong to the 3,5,5-trisubstituted 2-isoxazolines.

**Scheme 254:**
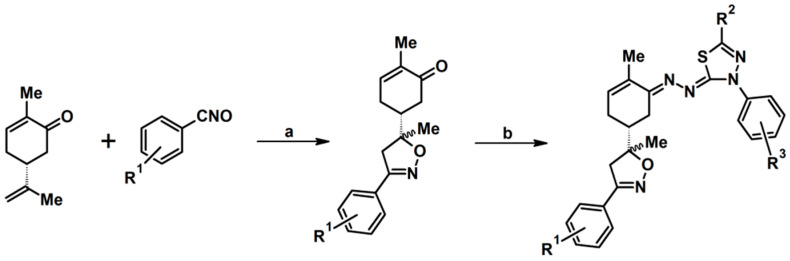
Syntheses of trisubstituted isoxazolines via 1,3-DP cycloaddition of R^1^-PhCNO to (*R*)-carvone [270]. a = CH_2_Cl_2_, NaOCl; b = NH_2_CSNHNH_2,_ H_2_SO_4_, EtOH, rfx., 3 h, then diarylnitrilimines, EtOH, rfx., 3 h; R^1^, R^2^, R^3^ = H, COOEt, Me; Me, COOEt, Me; *o*-MeO, COOEt, *p*-O_2_N; *o*-MeO, *p*-ClC_6_H_4_, H and others; up to 81% yield (final products).

Natural (*R*)-carvone was used by Oubella as a dipolarophile for the efficient syntheses of 3,5,5-trisubstituted 2-isoxazoline bearing the carvone-1,3,4-thiadiazole-deriving moiety in position 3—Scheme 254 [270].

Anticancer activity evaluation reveals that almost all hybrids have moderate to high antiproliferative activity, especially one of them (R^1^ = *p*-MeC_6_H_4_, R^2^ = Ph, R^3^ = *p*-MeC_6_H_4_). Moreover, data obtained on Annexin-V staining and caspase-3/7 activation showed that the effect of the tested isoxazolines could be instead attributed to an apoptotic mechanism.

**Scheme 255:**
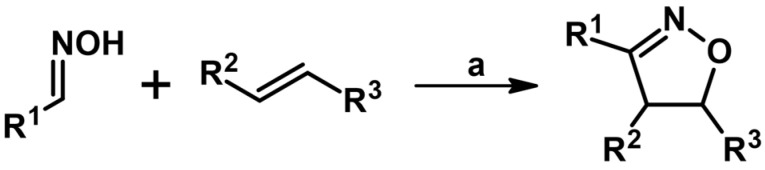
Syntheses of 3,4,5-trisubstituted isoxazolines via 1,3-DP cycloaddition and applying nitro compounds as the R^1^CNO sources [150]. R ^1^, R^2^, R^3^ = H, H, *p*-MeOC_6_H_4_; H, Me, Ph; H, CF_3_, Ph; COOMe, H, COOMe; CO_2_Me, H, Ph; a = PhNCO, Et_3_N, PhMe, 60 °C, 24 h; 36–95% yield.

As stated in another article by Umemoto, Imayoshi, and Tsubaki, 3,5-, 3,4,5-, and 3,5,5-trisubstituted 2-isoxazolines were prepared via 1,3-DP cycloaddition of oxime-substituted nitrile oxide to various alkenes—Scheme 255 [150]. In this procedure, a nitro compound, namely PhC=N(OMe)CH_2_NO_2_, played the role of the RCNO precursor.

**Scheme 256:**
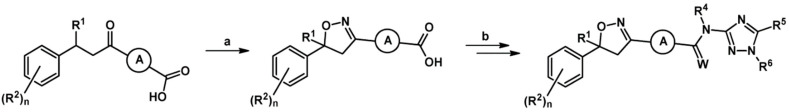
Intramolecular 1,3-DP cycloaddition as a starting step in a synthetic method leading to more complex compounds [271]. a = NH_2_OH, Bu_4_N^+^Br^−^, PhMe, H_2_O; b = further chemical transformations; R^1^, R^2^, R^4^, R^5^, R^6^, A, W, and n are defined in the invention.

The patented invention from 2022 reveals compounds bearing isoxazoline motifs connected with other structural motifs and dedicated as pest control agents—Scheme 256 [271]. The isoxazoline motif was constructed via intramolecular 1,3-DP cycloaddition, and the obtained isoxazolines underwent further structural modifications.

### 2.7. 3,4,4,5-Tetrasubstituted 2-Isoxazolines

**Scheme 257:**
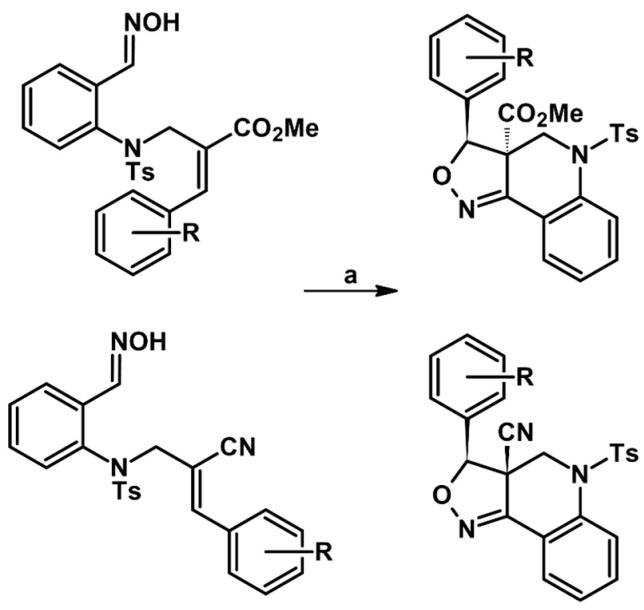
Intramolecular 1,3-DP cycloaddition of CNO into carbon–carbon double bond involved in the syntheses of tricyclic 2-isoxazolines containing fused isoxazoline and tetrahydroquinoline fragments [272]. R = H, *o*-Me, 3,4-(MeO)_2_, *m*-Cl, 2,4-Cl_2_, *p*-*i*-Pr, and others. a = NCS, Et_3_N, CCl_4_, rt, 6 h; up to 84% yield.

In 2015, Bakthadoss and Vinayagam presented tricyclic, tetrasubstituted (fused with tetrahydroquinoline) 2-isoxazolines obtained via intramolecular highly regio- and diastereoselective cycloaddition of an in situ-generated –CNO motif to a –CH=CH– motif, both coming from the same molecules—Scheme 257 [272].

### 2.8. 3,4,5,5-Tetrasubstituted 2-Isoxazolines

**Scheme 258:**
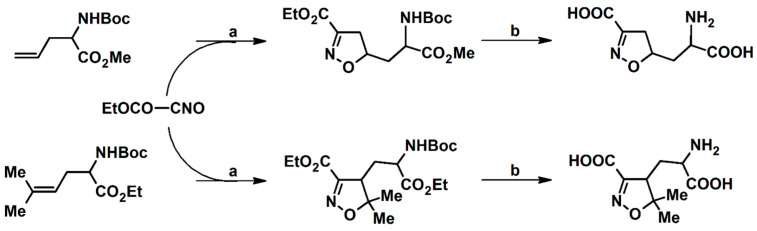
Syntheses of 3,5-di- and 3,4,5,5-tetrasubstituted isoxazolines bearing protected amino- and carboxylic groups [273]. a = NaHCO_3_, AcOEt followed by NaOH, MeOH; b = typical deprotection of ester and amido groups; up to 97% yield.

Conti and coworkers performed another 1,3-DP cycloaddition in the synthesis of 3,5-di- and 3,4,5,5-tetrasubstituted isoxazolines—Scheme 258 [273]. Free amino acids were obtained after the removal of a protected group in the reaction with NaOH in MeOH.

Two of the obtained amino acids behaved in vitro as antagonists of NMDA receptors and in vivo, after administration on DBA/2 mice, as effective anticonvulsants.

**Scheme 259:**
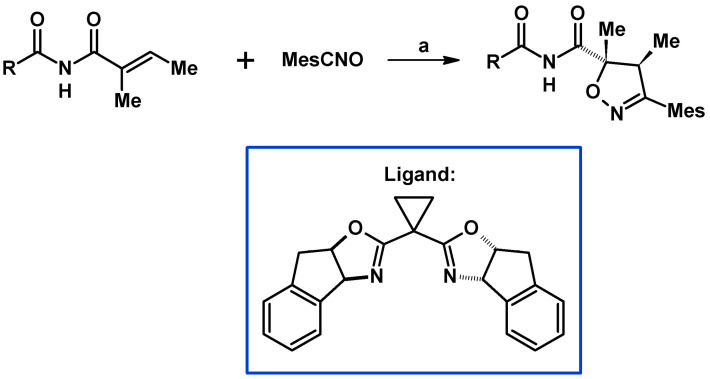
Enantioselective cycloaddition of nitrile oxide to acrylimide mediated by *Mg* and *Ni*-complexes with chiral bidentate ligand equipped with two chiral isoxazoline moieties [274]. R = Ph, *i*-Pr, *t*-Bu; a = MgI_2_, Mg(ClO_4_)_2_, Mg(Ntf_2_)_2_, and Ni(ClO_4_)_2_ complexes with L were tested. a = Mg or Ni salts (Lewis acid); CH_2_Cl_2_, rt, up to 7 days; 99% yield; *ee* = up to 83%.

A year later, Sibi et al. presented chiral 3,4,5,5-tetrasubstituted 2-isoxazolines obtained via 1,3-DP cycloaddition, mediated by Mg or Ni complexes with chiral bis-isoxazoline ligand, of nitrile oxide to acrylimides—Scheme 259 [274].

**Scheme 260:**
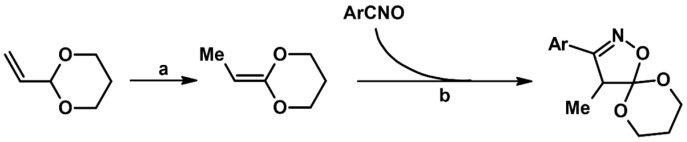
Syntheses of tetrasubstituted isoxazolines from acrolein acetals and nitrile oxide. Structures of obtained isoxazolines [239,275]. Ar = 2,6-Cl_2_C_6_H_3_; a = [RuClH(CO)(PPh_3_)_3_], THF; b = NCS, Et_3_N, CH_2_Cl_2_, from 0 °C to rt, 24 h; up to 87% yield.

**Figure 11:**
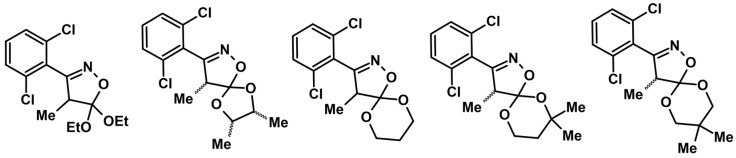
Tetrasubstituted isoxazolines obtained via the procedure presented in the scheme above (selected examples).

Tandem double bond migration-1,3-dipolar cycloaddition was engaged for the syntheses of 3,4,5,5-tetrasubstituted isoxazolines possessing a spiro motif starting from vinylacetals and nitrile oxide, as reported by Krompiec and coworkers—Scheme 260 and Figure 11 [239,275].

**Scheme 261:**
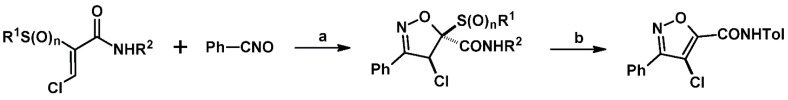
Regioselective cycloaddition of PhCNO to substituted acrylamides: syntheses of tetrasubstituted isoxazolines [276]. R^1^, R^2^, n = Bn, Bn, 1; Ph, Tol, 1; Ph, Me, 1; Bn, Bn, 0; Bn, *n*-Bu, 0, and others; a = Et_2_O, rt, 16 h; up to 72% yield b = next step (aromatization).

Other 3,4,5,5-tetrasubstituted isoxazolines were obtained via 1,3-DP cycloaddition of PhCNO to 2-thio-3-chloroacrylamides—Scheme 261 [276]. Interestingly, isoxazolines the obtained by Kissane and colleagues can be used to synthesize 3-chloroisoxazoles via HS(O)_n_R^1^ elimination. However, such a synthetic way was tested only for one reaction (R^1^ = Bn; R^2^ = Tol; 84% yield (isoxazole)).

The observed 1,3-DP cycloaddition regiochemistry agrees with theoretical predictions: nitrile oxide cycloadditions are dipole-LUMO controlled for conjugated dipolarophiles, and the most favorable direction of combination is that in which the carbon atom of the nitrile oxide adds to the ß-carbon of the ß-chloroacrylamide. This combination is also very favorable on steric grounds, as the dipole’s less crowded end adds to the dipolarophile’s more substituted end.

**Scheme 262:**
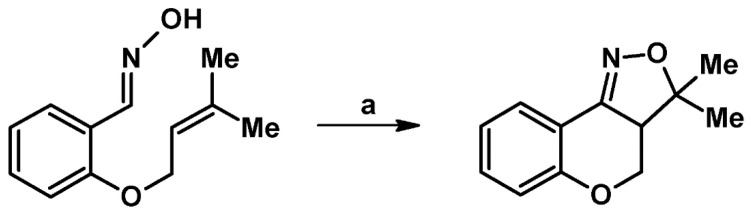
Intramolecular cycloaddition of nitrile group into double bond—synthesis of 3,4,5,5-tetrasubstituted isoxazolines (an example) [6,7]. a: HTPI, H_2_O, rt, 50 min; 81% yield.

3,4,5,5-tetrasubstituted 2-isoxazolines were obtained via intramolecular dipolar cycloaddition of a nitrile oxide motif to the double bond coming from the substituted allyloxy group present in the substrate—Scheme 262 [6,7]. The nitrile oxide motif was generated in situ from the oxime group using air- and water-stable [hydroxy(tosyloxy)iodo]benzene (HTIB)—analogically to the synthetic procedure applied for the syntheses of 3,4-disubstituted isoxazolines (see Section 2.2).

**Scheme 263:**
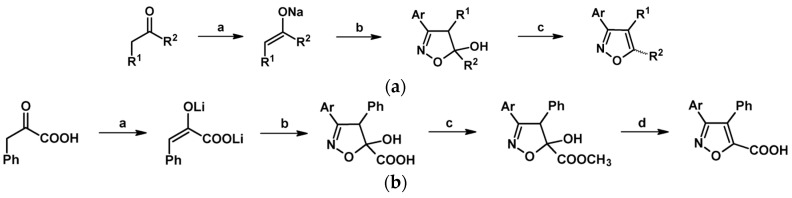
Synthesis of 5-hydroxy-2-isoxazolines via ArCNO 1,3-DP cycloaddition to lithium or sodium enolate. Dehydration of hydroxyisoxazolines into appropriate isoxazoles [213]. (**a**) R^1^, R^2^, Ar = Ph, Me, *m*-ClC_6_H_4_; *o*-FC_6_H_4_, Me, *o*-ClC_6_H_4_; Ph, Me, *o*-ClC_6_H_4_ and others; a = NaH/THF, 1 h, 0 °C; b = ArCNO/THF, 12 h, rt; aq. NH_4_Cl; c = aq. Na_2_CO_3_/MeOH. (**b**) Ar = 5-chlorofuran-2-yl; a = LDA, THF, 1 h, 0 °C; b = ArCNO/THF, 16 h; sat. NH_4_Cl; 2N HCl; c = BF_3_/MeOH, rt, 3 h; d = aq. Na_2_CO_3_, MeOH, rfx.

Vitale and coworkers described in their article a synthetic procedure leading to 3,4,5,5-tetrasubstituted 2-isoxazolines bearing a hydroxy group in position 5. It involved 1,3-DP cycloaddition of ArCNO to in situ-generated enolates from the appropriate benzyl ketones or 3-phenylpropanoic acid—Scheme 263 [213]. The obtained isoxazolines were finally transformed into isoxazoles via simple dehydration.

The pharmacological characterization and docking analysis conducted for the final products, i.e., diarylisoxazoles, showed that the selected derivatives are highly selective cyclooxygenase-1 (COX-1) inhibitors.

**Scheme 264:**
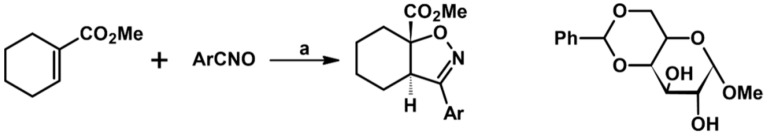
Fully diastereoselective 1,3-DP cycloaddition of p-CF_3_C_6_H_4_CNO to cyclohexene carboxylate mediated by chiral Yb(OTf)_3_-glucose-derivative (presented in the scheme) complex [277]. a = CH_2_Cl_2_, rt, 24 h; 20% yield.

Gucma et al. proved that 1,3-DP cycloaddition of p-CF_3_C_6_H_4_CNO to various cyclohexenecarboxylates can be regio, stereo-, and enantioselective, depending on carboxylate and catalyst [277]. A particularly interesting instance of 3,4,5,5-tetrasubstituted, chiral 2-isoxazolines synthesis is depicted in Scheme 264 below.

**Scheme 265:**
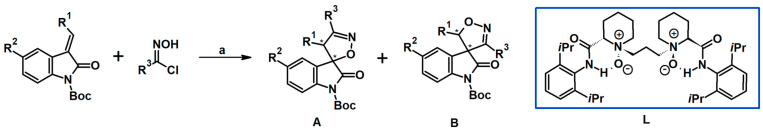
Syntheses of spiro-isoxazoline-oxindole hybrids: brilliant enatioselective 1,3-DP cycloaddition of RCNO to 3-arylidene-oxindole mediated by chiral Ni(II)-*N*,*N*’-diaminedioxide complex [278]. R^1^, R^2^, R^3^, %y, (**A**/**B**), %ee = H, Ph, Ph, 43, 97/3, >99; *p*-FC_6_H_4_, H, Ph, 45, 98/2, >99; 2,6-Cl_2_C_6_H_3_, H, Ph, 53, >99/1, 99; 2-naphthyl, H, 40, 71/29, 93; H, Ph, *p*-FC_6_H_4_, 60, 96/4, 97; H, Ph, *m*-BrC_6_H_4_, 46, 95/5, 99; and others; a = Ni(ClO_4_)_2_·6H_2_O, L, ClCH_2_CH_2_Cl, MS 4Å, 35 °C; = up to 65% yield (27 examples); A/B = usually > 99; *ee* = up to 99%, * = stereogenic center.

A highly enantioselective and good regio- and diastereoselective 1,3-DP cycloaddition of in situ-generated RCNO to 3-arylidene-oxindoles allowed researchers to obtain spiro-isoxazoline-oxindole hybrids—Scheme 265 [278]. The brilliant *ee* was gained due to the application of a chiral *N*,*N*′-dioxide−nickel(II) complex catalyst under mild reaction conditions.

An asymmetric catalytic model was also proposed as a hexacoordinated octahedral structure of the *N*,*N*′-dioxide-Ni(II)-dipolarophile complex. Namely, the Re face of the dipolarophile is effectively shielded by the amide moiety and piperidine ring underneath the ligand. In contrast, the Si face is located in a relatively open space, resulting in the highly selective approach of the RCNO toward the Si face of the bidentate-coordinated dipolarophile. Finally, the (3*S*,4′*R*)-cycloadduct is formed selectively.

**Scheme 266:**
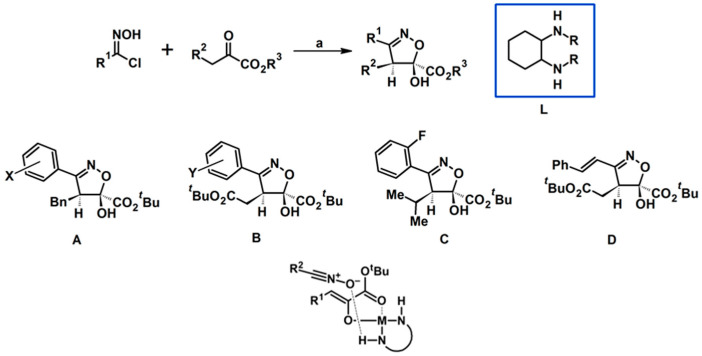
Syntheses of chiral 3,4,5,5-tetrasubstituted isoxazolines via 1,3-DP cycloaddition of RCNO to in situ-generated α-keto ester enolates [279]. The crucial TS for this cycloaddition. X = *p*-F, *p*-Me, *p*-F_3_C, *p*-Ph, *o*-F, *o*-MeO, *o*-Br, *o*-Me; Y = 4-Br, 4-Me, 3-F, 2-F; and others; R^1^, R^2^, R^3^ (examples)—see isoxazolines (**A**–**D**); a = Et_3_N, 0 °C, 10 min, then Cu(AcO)_2_ + L, *i*-PrOH, −40 °C; R (in L) = -CH_2_(2,4,6-Me_3_C_6_H) or -CH_2_(2-F_3_CC_6_H_3_); 45–99% yield; *ee* = 74–96% (in the majority of cases >90%).

An enantioselective [3 + 2] cycloaddition of both nitrile oxides and enolates, generated in situ, of α-keto esters, catalyzed by Cu(II)-chiral diamine complexes, was used for the synthesis of chiral, 5-hydroxy-isoxazolines—Scheme 266 [279].

Notably, the procedure presented above can be realized in the gram scale. Additionally, mechanistic aspects were also raised; namely, the crucial interaction between reagents and catalyst for regio- and stereoselectivity was discussed—see scheme above.

**Scheme 267:**
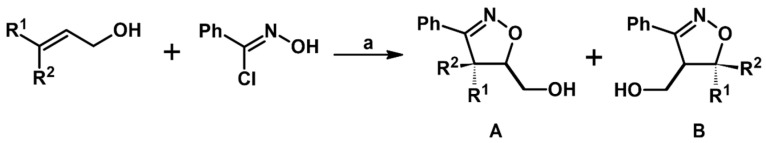
Coordination to *Mg* atom as a crucial element in the regioselective syntheses of isoxazolines from allyl alcohol [280,281]. R^1^, R^2^ = Me, H; *n*-Pr, H; H, *n*-Pr; Ph, H; Me, Me; a = *n*-BuOMe, CH_2_Cl_2_, rt; up to 100% yield; regioselectivity (**A**/**B**): up to 0:100.

The highly regioselective creation of tetrasubstituted isoxazolines was described in the review in 2018 by Roscales and Plumet [280]. They cited the 1992 cycloaddition of in situ-generated benzonitrile oxide to allyl alcohols performed successfully by Kanemasa, Nishiuchi, and Wada—Scheme 267 [281].

According to the authors, the coordination of both oxygen atoms to the *Mg* atom is essential for high regioselectivity of these reactions. Importantly, a variety of substitution patterns, both aromatic and aliphatic moieties, are well-tolerated, leading to isoxazolines with moderate to excellent yield. One should keep in mind that a number of publications devoted to metal-catalyzed (Ir, Mg, Ru, Yb, and others) 1,3-DP cycloaddition of RCNO leading to isoxazolines were presented in the review from 2018.

**Scheme 268:**
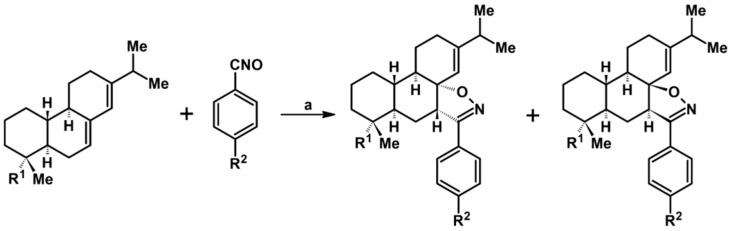
Syntheses of 3,4,5,5-trisubstituted 2-isoxazolines from selected abietates [282]. R^1^, R^2^ = Me, CF_3_; Me, *i*-Pr; Ph, CF_3_; Bn, CF_3_; a = CH_2_Cl_2_, rt, overnight, up to 15% yield.

Gołębiewski et al. described high regio- and site-selectivity of the [2 + 3] cycloaddition of *p*-XC_6_H_4_CNO to selected abietates—Scheme 268 [282].

Notably, only one isomeric isoxazoline was obtained; monoadducts to the more exposed C7–C8 double bond were observed.

**Scheme 269:**
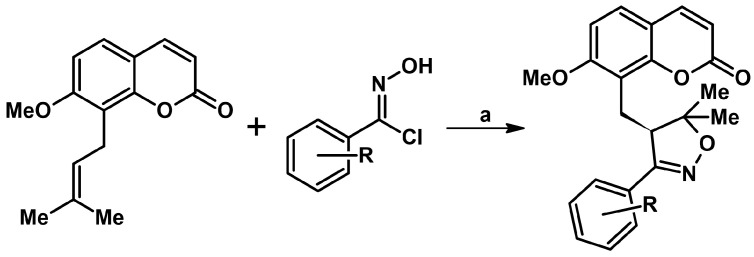
Synthesis of 3,4,5,5-tetrasubstituted 2-isoxazolines equipped with osthole motif [283]. R = *o*-, *m*-, or *p*-F; Cl or Br; and others; a = Et_3_N, CH_2_Cl_2_, rt, up to 36 h, up to 40% yield.

Recently, osthole-based 3,4,5,5-tetrasubstituted isoxazolines were synthesized typically, namely via 1,3-DP cycloaddition of in situ-generated substituted benzonitrile oxide to osthole—Scheme 269 [283].

Among all the osthole–isoxazoline hybrids, the derivative with R = *m*-F displayed the most promising growth-inhibitory effect on *Mythimna separata*. Namely, its mortality rate was 1.80 times higher than those of both osthole and toosendanin. Moreover, the derivative equipped with 2-F-5-BrC_6_H_3_ displayed the most promising larvicidal activity against *Plutella xylostella*, which was superior to rotenone. Additionally, the toxicity evaluation suggested that these osthole-based isoxazoline derivatives showed relatively low toxicity toward non-target organisms.

### 2.9. Multisubstituted Isoxazolines

This chapter presents literature reports, particularly patents, which simultaneously presented isoxazolines from mono- to fully substituted derivatives. That applies in particular to patents in which an extensive range of claims covers all possible substituted isoxazolines.

**Scheme 270:**
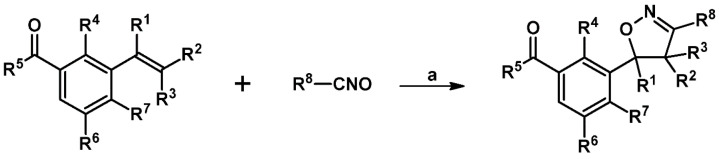
Syntheses of polysubstituted 2-isoxazolines—up to fully substituted ones [284]. R^1^–R^8^ = H, alkyl, aryl, halogen, cyano, nitro group, and others. Many substituents are enumerated in the claims; a = various conditions (depending on R^1^–R^8^).

In a US patent from 2003, several multisubstituted (up to fully substituted) 2-isoxazolines were presented [284]. All isoxazolines were obtained via dipolar cycloaddition of nitrile oxide (generated in a typical manner) to styrene derivatives—Scheme 270.

**Scheme 271:**
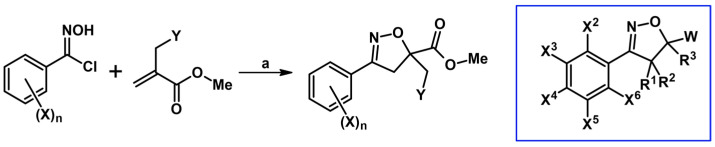
Isoxazolines for agriculture: preparation method and general formula [285]. a = typical 1,3-DP cycloaddition conditions. In the general formula, Y = *O*alkyl, Br, and others; X^2^–X^5^ and R^1^–R^3^ are hydrogen, halogen, organic substituents such as substituted alkyl, W is carboxyl, cyano, or CHO group; all substituents are defined in the invention.

Multisubstituted isoxazolines (3,5-di-, 3,5,5-tri-, up to 3,4,4,5,5-tetrasubstituted ones) dedicated to agriculture as herbicides were obtained involving 1,3-DP cycloaddition of various nitrile oxides to various dipolarophiles—Scheme 271 [285].

**Figure 12:**
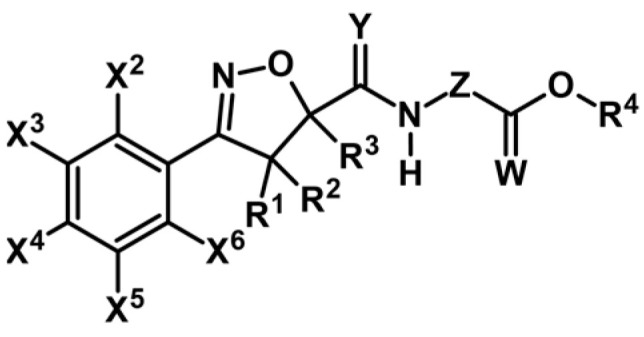
The general formula of herbicidally active isoxazolines [268]. Y and Z = O or S; X^1^–X^6^, R^1^–R^4^ and Z are defined in the invention.

1,3-DP cycloaddition of RCNO to various dipolarophiles was involved in the synthetic route leading to the herbicidally active isoxazolines, namely 3-phenylisoxazoline-5-carboxamides of tetrahydro- and dihydrofurancarboxylic acids and esters possessing the general formula presented in Figure 12 [268].

The isoxazolines mentioned above and their acceptable agrochemical salts have been applied in the crop protection sector.

## 3. Mechanistic Aspect

When discussing subsequent works on the synthesis of isoxazolines via cycloaddition, we included in the description elements relating to the reaction mechanism if the authors presented them. However, basic knowledge regarding the mechanism of 1,3-dipolar cycloaddition of nitrile oxide to the carbon–carbon double bond leading to 2-isoxazolines can be found in the reviews and books [149,280,286,287].

Considering the rapid development of computational methods, in our review, we discussed the works on the application of the DFT and ab initio methods for the analysis of 1,3-DP cycloaddition only concerning the works from the last few years. We have quoted the remaining works from the 21st century, including reviews, and presented their content in one or two sentences below.

Ab initio molecular orbital calculations at the MP4(SDTQ)/6-31Gp//RHF/6-31Gp level of theory were used to obtain detailed insight into the reaction profile of the Mg-controlled 1,3-DP cycloaddition of nitrile oxides with allylic alcohols. It was confirmed that the complex formation reduces the HOMO–LUMO energy gaps of the two reactants [288].

The regiochemistry of the cycloadditions of nitrile oxides to crotonaldehyde and cinnamaldehyde has been determined and is dictated by frontier orbital interactions and secondary orbital interactions as well. In cycloadditions to α,ß-unsaturated compounds, the directive effect of the frontier orbital interactions can be diverted by steric drifts and secondary orbital interactions [289].

The 1,3-dipolar cycloaddition reactions between nitrile oxides and chiral allylic fluorides were theoretically examined. Both experimental and transition-state modelling revealed that the electronegativity and steric bulk of the allylic substituents influence the diastereoselectivity of the reactions [290].

Mechanistic insight into diastereoselective 1,3-dipolar cycloadditions of nitrile oxides to chiral homoallylic alcohols, especially an explanation of the observed diastereoselectivity, is given by Luft et al. [291]. Namely, the transition structures of these reactions were explored with DFT calculations (B3LYP/6-11+G(d,p)+CPCM(dichloromethane)//B3LYP/6-31+G(d)). It was shown that the anti-product is favored (kinetically and thermodynamically) in both the thermal and *Mg*-mediated reactions. Notably, DFT calculation results revealed that diastereoselectivity is increased by adding Grignard reagents, in agreement with experimental results [70].

Furthermore, in the report from Efimov and coworkers [225] (described in Section 2.5), experimental and theoretical studies allowed the classification of the reaction of enamines with nitrile oxides as inverse electron demand 1,3-dipolar cycloaddition. In the mentioned work, the DFT/B3LYP level of theory was applied to gain insights into the cycloaddition mechanism between nitrile oxides and enamines. The calculational results suggest that the configuration around the double bond of the enamine will control the stereoconfiguration of the isoxazoline formed. The greater stability of the *E*-isomer of enamine compared to the *Z*-isomer generally affects the shift of the equilibrium towards the presence of the former and will have an impact on the formation of isoxazolines as *trans* isomers. Based on the theoretically determined energetics of the formation process of possible transition states, the most likely mechanism is the concerted path of cyclization in contrast to the also-considered stepwise mechanism’s pathway. Nevertheless, the transition state geometries obtained in calculations indicate that the concerted mechanism has a significantly asynchronous character. Of all the considered transition state structures, the creation of the lowest energy transition state, which determines the selective formation of the final product, is a result of both easier accessibility of the state governed by longer-range orbital interactions and a small value of the total geometry distortion energy of reagents. The analysis of molecular orbital isosurfaces shows that the HOMO orbital of enamine is predominantly localized at the β-carbon atom from the amino group. In contrast, the LUMO of nitrile oxide is mainly localized on the carbon atom of the nitrile group. These results correlate with the inverse electron demand concept for the investigated reaction, which explains the observed reaction rate increase when an electron-withdrawing group is introduced to the structure of nitrile oxide. Broadly speaking, the stereoselectivity of the considered cycloaddition is driven by the higher stability of the E-isomer of the dienophile, whereas the regioselectivity is controlled by a better orbital overlap in the transition state leading to the experimentally observed regioisomer.

The DFT method provided the opportunity to correctly assess the molecular structure of isoxazolines–benzothiazinone hybrids, obtained via 1,3-DP cycloaddition of benzonitrile oxide and its derivatives to N-allylbenzothiazinone by Sebbar et al. [154]. The described theoretical and experimental results were in good agreement (see more in Section 2.3).

Another case study concerned an acridone-CH_2_-isoxazoline hybrid obtained by Aarjane and colleagues via 1,3-DP cycloaddition of *p*-ClC6H_4_CNO to *N*-allylacridone, described in Section 2.3 [155]. As presented in this work, the calculational results from the DFT method can also be used to predict some aspects of the reactivity of isoxazoline systems. The estimated ionization potential and electron affinity enable the determination of the chemical reactivity descriptors such as electronegativity, chemical hardness and softness, and chemical potential, thus allowing the attribution of the global reactivity properties of isoxazolines. Potential directions of electronic and electrostatic interaction of the isoxazoline system in the environment of reactive factors can be foreseen, among others based on the distribution of Mülliken charges and maps of the molecular electrostatic potential. Such computational results for 10-{[3-(4-chlorophenyl)-4,5-dihydro-1,2-oxazol-5-yl]methyl} acridone showed that the positive regions are focused on the hydrogen atoms of the isoxazoline ring, while the negative regions are around the oxygen atoms of the acridone and isoxazoline rings.

Using the DFT approach with the hybrid B3LYB and double-hybrid B2PLYP functional allowed other research groups to investigate the reaction profile for the formation of previously analyzed isoxazoline (synthetic procedure described in Section 5.4) [292].

**Figure 13:**
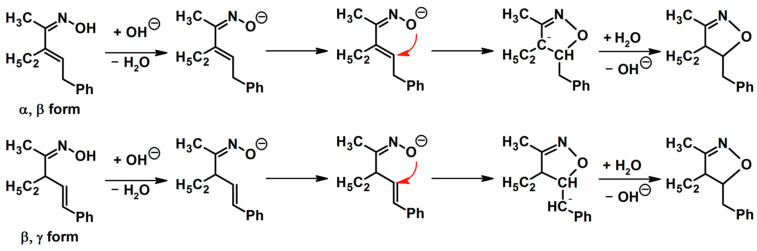
Isoxazoline ring closure mechanism involving form α,β and β, γ oxime.

In the reaction of ketones with aryl acetylenes and hydroxylamine, the step of the reaction directly leading to the formation of isoxazoline is intramolecular ring closure in the system of unsaturated oxime. Potentially, both the unsaturated α,β form and β,γ form of the oxime may participate in ring closure, as shown in Figure 13.

**Figure 14:**
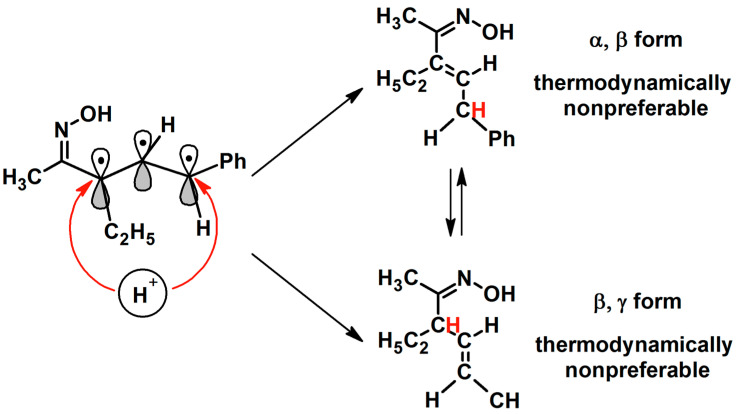
Formation of α,β and β,γ unsaturated forms of the oxime.

It is very interesting to note that in the case of the reaction under consideration, the intramolecular nucleophilic attachment to the double bond of an unsaturated oxime, the ring closure, and the formation of a five-membered heterocycle proceed with participation of the β,γ-unsaturated oxime, not the unsaturated form of α,β, as usually assumed. The argument in favor of the preferred participation of the form β,γ is the fact that when the terminal group of the oxime chain is a phenyl substituent, the β,γ-unsaturated form becomes more thermodynamically preferable, as shown in Figure 14. According to the calculational results, the migration of the double bond to the α,β-position is accompanied by a pronounced increase in the Gibbs free energy ranging from about 2.40 kcal/mol to about 4.5 kcal/mol, depending on the *E*/*Z* isomerization of the oxime group. Therefore, the calculational results at the DFT level of theory clearly show that the prototropic rearrangement of the β,γ-unsaturated oxime to the α,β-unsaturated form is rather thermodynamically unfavorable, and simultaneously, those findings suggest that the β,γ-unsaturated form of oxime can directly participate in the closure of the isoxazoline ring.

**Figure 15:**
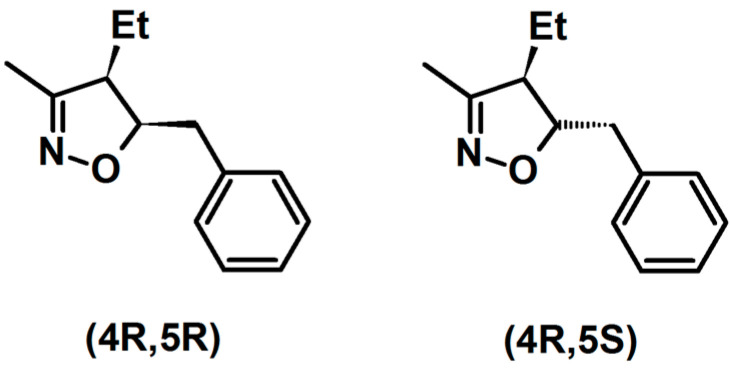
Stereoisomers of the 5-benzyl-4-ethyl-3-methyl-2-isoxazolines.

The calculational investigations also predict that the cyclization involving an oxygen anion is associated with a high activation barrier in the case of the α,β form. On the contrary, the closure of the β,γ-unsaturated oximate ion is very easy with a smaller energy barrier. For the final stage of the reaction, the calculated Gibbs free energy barrier, associated with ring closure for β,γ-unsaturated oxime, is about 40% lower compared to the analogous barrier found in the cyclization of the α,β-unsaturated form. Then, after ring closure, the protonation of the intermediate carbanion with the participation of a water molecule completes the formation of 4,5-dihydroisoxazole throughout the reaction cycle. It is worth noting that regardless of the form of the unsaturated oxime, the theoretically predicted reaction pathways always lead to the thermodynamically preferred (4*R*,5*S*) stereoisomer of isoxazoline. As the calculations show, the energy of the stable (4*R*,5*R*) isomer is always slightly higher than that of the most stable conformations of (4*R*,5*S*) isomers—see Figure 15 below. Hence it is clear that, according to the computationally proposed reaction paths, the real reaction should lead to the formation of a stable (4*R*,5*S*) isomer with significant yield.

**Scheme 272:**
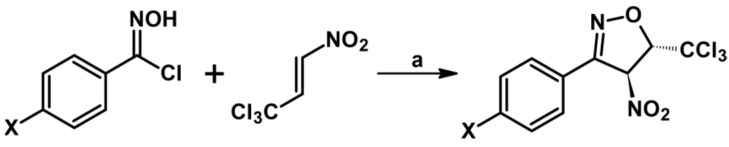
Reaction between *p*-XC_6_H_4_CNO and (*E*)-Cl_3_CCH=CHNO_2_ leading to isoxazolines [293]. X = H, Me, MeO, O_2_N, F, Cl; a = base (K_2_CO_3_ or Et_3_N), rt and 24 h, or rfx and 6 h; up to 47% yield.

In the work of Zawadzinska et al. from 2021, 1,3-DP cycloaddition of nitrile oxide to 3,3,3-trichloro-1-nitroprop-1-ene was presented in the light of the experimental and MEDT quantum-chemical study—Scheme 272 [293].

From a theoretical perspective at the DFT level of theory, the [3 + 2] cycloaddition of arylsubstituted nitrile *N*-oxides with trichloronitropropene has a polar character. It proceeds through a two-stage one-step mechanism in which the formation of the O–C bond probably occurs when the C–C bond is practically formed. The observed regioselectivity of this cycloaddition reaction was computationally explained at the molecular level. Calculational results indicate that ortho isoxazolines should be major isomers formed in the reaction, whereas meta isomers should arise in negligible amounts. For the R–C≡N–O structure of nitrile *N*-oxides, the calculations show that the most probable electronic valence structure of a monovalent –CNO functional group has ionic character –C≡N^+^–O^−^ and corresponds to substantial polarization of electron density between the oxygen atom and the remaining C≡N fragment. Therefore, like the simplest acetonitrile oxide, arylsubstituted nitrile *N*-oxides can participate in polar-type cycloaddition reactions. Simultaneously, theoretical reactivity indices of reagents, like the global electrophilicity and global nucleophilicity, suggest that nitrile oxides behave as moderate nucleophiles, while trichloronitropropene acts as a very strong electrophile in the considered reactions. The analysis based on P_k_^−^ and P_k_^+^ Parr functions explains in more detail that most nucleophilic and most electrophilic centers of these reacting species are, respectively, oxygen of nitrile oxides and carbon adjacent to the –CCl_3_ group in trichloronitropropene. Because, in the polar reactions, the bond formation takes place between the most nucleophilic center of the nucleophile and the most electrophilic center of the electrophile, in the case of the discussed cycloaddition, the course of the reaction should be determined by the strong interaction of nitrile oxide with the corresponding carbon of trichloronitropropene. Thus, the distribution of nucleophilic and electrophilic properties seen in the theoretical results clearly indicates that the preferred reaction pathway is the one that should lead to the formation of the ortho isomer. Moreover, theoretical results indicate that the differences in the reactivity of nitrile oxides substituted by the various aryl substituents come from the different nucleophilic activation of the trichloronitropropene and polar character of the cycloaddition reactions. The preferred direction of the reaction leading to the formation of ortho isomers was also theoretically confirmed based on the energy profile of the possible reaction paths as a function of the calculated Gibbs free energy and enthalpy. Although the differences in the activation energy of the transition state and the stabilization of the cycloaddition products on the paths leading to the formation of *ortho* and *meta* isomers, respectively, are not very clear and do not allow the firm determination of the preferred direction of the reaction, the composition of the reaction mixture calculated on the basis of the transition state theory and Eyring–Polany equation for the reaction of nitrile oxides with trichloronitropropene positively predicts the dominant proportion of the ortho isomer. The terms *ortho* and *meta* were introduced by the authors, and they should be treated only figuratively. From a stereochemistry point of view, they are regioisomers (*ortho*: 4-nitro-5-trichloromethyl; *meta*: 5-nitro-4-trichloromethyl). It is also worth pointing out that the calculations do not reveal any significant differences in the electronic structure of the –CNO group upon aryl substitution, suggesting that such substitution is unlikely to lead to significant reactivity changes. From a quantum-chemical point of view, the similar mechanistic and molecular aspect of dipolar cycloaddition with the participation of the three-atom component and the appropriate double bond of the two-atom alkene fragment is presented in the review work [294].

Quinazoline–isoxazoline hybrids obtained via regioselective 1,3-DP cycloaddition of substituted benzonitrile oxide to *N*-allylquinazolinone by Rhazi and coworkers were once the subject of theoretical assessment [164] In this work, the analysis of theoretically predicted nucleophilic and electrophilic properties of reagents clearly explains a concerted mechanism for the dipolar cycloaddition reaction. The quantum theory of the atom in the molecule used to characterize the electronic structure of the transition state reveals C–C bond formation between the terminal carbon atom of *N*-allylquinazolinone and the carbon atom of the CNO group of benzonitrile oxide. At the same time, a CN bond is formed, which finally leads to the closure of the isoxazole ring. The localized orbital localizer (LOL), introduced by Schmider and Becke, confirms the existence of a chemical bond in the C–C region, but also shows a slightly weaker interaction between the carbon atom and the oxygen atom. Thus, these results are significantly similar to the computational results presented in the *Molecules* article [293]. This character of the computational research remains fully consistent with the experimental results and confirms observed regiochemistry for the 1,3-dipolar cycloaddition between the arylnitriloxides and *N*-allylquinazolinone.

In conclusion, chemistry in silico, especially DFT calculations, plays an increasingly important role in planning target structures and, above all, in understanding the mechanism of 1,3-DP cycloaddition, including regio- and stereoselectivity of these reactions.

## 4. Side Reaction Accompanying 1,3-Dipolar Cycloaddition

**Figure 16:**
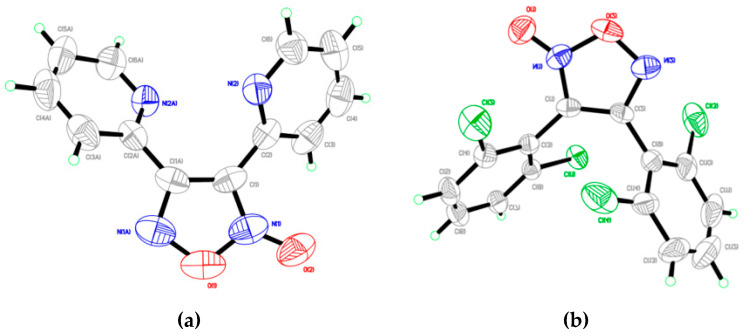
Pirydyn-2-yl carbonitrile (**a**) and 2,6-dichlorobenzonitrile oxide (**b**) dimers: 3,4-bis(pirydin-2-yl)furoxane (**a**) and 3,4-bis(2,6-dichlorophenyl)furoxane (**b**).

As in almost every synthesis, in the synthesis of isoxazolines via 1,3-DP of RCNO to C=C, side reactions reduce the final yield of the expected cycloadduct. In this review, we do not discuss side effects involving dipolarophiles, as these vary greatly depending on the functional groups present in the dipolarophiles. There are, of course, dipolarophiles that are completely stable and do not undergo any side reactions, e.g., simple alkenes. However, side reactions related to nitrile oxides are crucial, which results from their varied stability and is well-known and described [295]. For example, it is generally known that oxides such as 2,4,6-trimethylbenzonitrile oxide and 2,4,6-trimethoxybenzonitrile oxide are very stable and do not undergo dimerization or other undesirable transformations. However, most nitrile oxides are unstable and dimerize very quickly to furoxanes and other undesirable transformations. Therefore, the vast majority of isoxazoline syntheses are carried out in a way that nitrile oxides are generated in situ from appropriate oxymoyl chlorides. The latter can also be, and most often are, generated in situ and then further transformed in situ into RCNO. When discussing the synthesis of isoxazolines in Section 2, we always showed how RCNO was created. As mentioned, the most commonly observed adverse reaction was dimerization of RCNO into furoxanes. In some cases, it was possible to confirm the structure of RCNO dimers using X-ray—Figure 16 [201,240].

It should be added that RCNO dimerization to furoxanes is not only a side reaction accompanying cycloaddition but also an important method of furoxane synthesis [156,296]. These compounds are attractive as pharmaceuticals [297] or even as explosive materials [298].

In summary, when RCNOs are stable, they can be used for cycloaddition without worrying about dimerization, and additionally, a significant excess of dienophile is not required. However, when their durability is insufficient, generating them in situ and using a large excess of dienophile is preferable. In addition, basic information on RCNO (stability, acquisition, in situ generation) can be found in many papers, including reviews [149,287,299,300].

## 5. Methods of 2-Isoxazolines Synthesis Other Than Dipolar Cycloaddition

2-Isoxazolines can also be obtained via methods other than 1,3-DP cycloaddition of RCNO to the carbon–carbon double bond. This chapter briefly presents the most important and the most interesting, in our opinion, methods of synthesis of isoxazolines outside of DP cycloaddition. In the review by Kumar and Shankar in 2021, all synthetic routes leading to 2-isoxazolines are schematically presented; however, they are presented without details [301]. Selected syntheses of isoxazolines using methods other than 1,3-DP cycloaddition of RCNO are presented below in more detail.

### 5.1. From Nitrocompounds

**Scheme 273:**
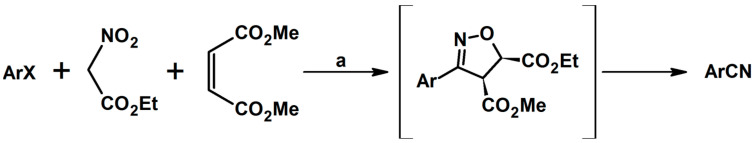
Isoxazolines as intermediates: *Pd*-catalyzed aryl cyanide syntheses [302]. X = Br or I; Ar = *o*-*i*PrC_6_H_4_, *p*-ClC_6_H_4_, *o*-NCC_6_H_4_, *p*-AcC_6_H_4_, 3-Py, 1-naphthyl, and others; a = [Pd(OAc)_2_], PPh_3_, PhOK, K2CO3, DMF, 120 °C, 24 h; up to 95% yield of ArCN (isoxazolines were not isolated).

In 2016, the three-component procedure—involving nitroacetate, aryl halide, and (*Z*)-dimethyl butenoate—was presented for the synthesis of trisubstituted isoxazolines equipped with two CO_2_Et groups—Scheme 273 [302]. The reaction involves the following steps: (**a**) *Pd*-mediated α-arylation of ethyl nitroacetate; (**b**) ArCNO formation; (**c**) 1,3-DP cycloaddition leading to 3,4,5-trisubstituted 2-isoxazolines; (**d**) isoxazoline cleavage to ArCN formation. The scheme below shows that the obtained isoxazolines can be useful intermediates in the cyanide-source-free *Pd*-catalyzed synthetic procedure.

**Scheme 274:**
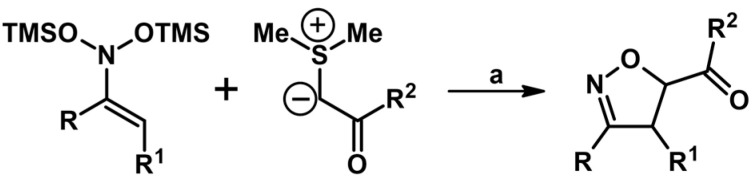
Syntheses of di- and trisubstituted isoxazolines from aliphatic nitro compounds—an alternative to [3 + 2] cycloadditions with nitrile oxides or silyl nitronates [303]. R, R^1^, R^2^ = Me, H, COPh; H, H, COPh; CH_2_Ph, H, COPh; Ph, Me, COPh and others; a = MeCN, 50 °C, 2 h; up to 91% yield.

3,5-di, 4,5-di-, and 3,4,5-trisubstituted isoxazolines were obtained in 2019 using the [4 + 1]-annulation of α-keto-stabilized sulfurylides with *N,N*-bis(siloxy)enamines derived from aliphatic nitro compounds—Scheme 274 [303].

**Scheme 275:**
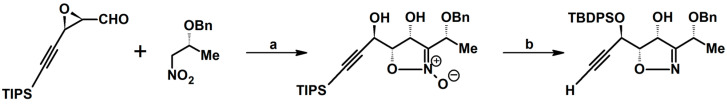
Syntheses of isoxazolines from nitro compounds and chiral epoxides—an example [304]. A = Cu(Oac)_2_, chiral ligand, *i*PrNEt_2_, dioxane, 41 h, 4 °C and then Et_3_N, 23 °C; 95% yield; b = TBAF, THF, 23 °C, R_3_SiCl and then P(Ome)_3_; 75% yield.

As reported in a paper from 2021, the application of specially designed nitro compounds and chiral epoxides as substrates in the synthesis of isoxazoline was one of the crucial steps in the synthetic route leading to the amino sugar fragment of the lincosamide antibiotics—Scheme 275 [304].

3-benzoylisoxazolines were obtained in the reactions involving alkenes, various α-nitroketones, and chloramine-T as the base. However, nitrile oxides are not involved in these reactions. In the key stage, the cycloaddition of acid-introtautomer of nitro compounds takes place. The resulting tetrahydroisoxazole is then transformed into isooxazoline [305]. Many similar syntheses (Michael Additions versus Cycloaddition Condensations) involving nitro compounds leading to 2–isoxazolines were previously published by Trogu et al. [306].

**Scheme 276:**
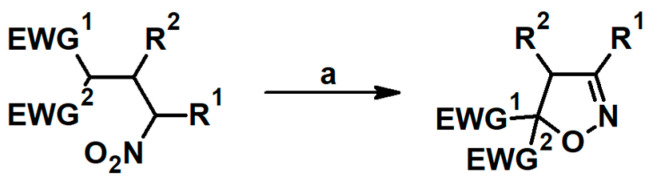
Syntheses of 3,4,5,5-tetrasubstituted isoxazolines equipped with two EWG group (CO_2_Me in most cases) substituents in position 5,5 [307]. EWG^1^, EWG^2^, R^1^, R^2^ = COOMe, COOMe, Me, 3,4,5-(MeO)_3_C_6_H_2_; COOMe, COOMe, Me, 5-bromofuran-2-yl; COOBn, COOBn, Me, *p*-ClC_6_H_4_; COOEt, COMe, COOEt, *p*-ClC_6_H_4_; and others; a = PivCl, base (Et_3_N + DMAP or DBU), MeCN, 20–40 °C, 2–7 days; up to 91% yield.

In August 2022, a new and promising synthetic procedure led to isoxazolines under mild conditions. The approach uses simple reagents with broad tolerance scope for versatile reagents—Scheme 276 [307]. In this work, 3,4,5,5–tetrasubstituted isoxazolines were obtained from nitroalkylmalonates via redox-neutral ring-closure reaction as a crucial step.

What is particularly worth emphasizing is that the synthesis of chiral isoxazolines via the procedure mentioned above is also achievable. Namely, chiral isoxazolines with high enantioselectivity (*er* = up to 96/4) involving chiral nitroalkylmalonates were obtained. Importantly, the obtained isoxazolines are valuable substrates for preparing pyrrolidone and furane derivatives, demonstrating the utility of the obtained products in organic synthesis. The mechanism of sooxazoline formation in the transformations mentioned above is still under investigation.

**Scheme 277:**
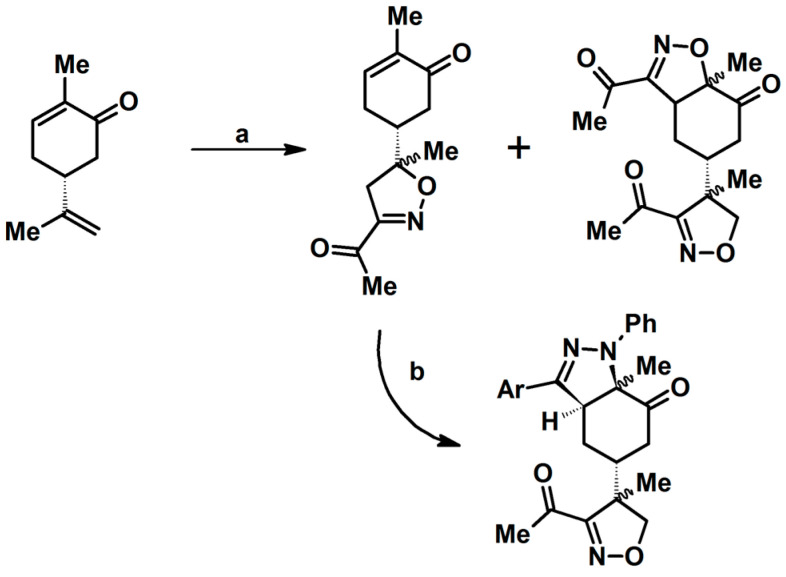
Syntheses of acetyl isoxazolines using acetone-Fe(NO_3_)_3_ system [308]. Ar = Ph, p-MeC_6_H_4_, p-ClC_6_H_4_; a = acetone, Fe(NO_3_)_3_; b = next step.

The method involving nitro-intermediates was used in 2022 by Fawzi and colleagues for obtaining 3-acetylisoxazolines and 3-acetylisoxazole-pyrazole hybrids from I-carvone aI(R)-limonene using an acetone/iron(III) nitrate system in the 1,3-DP cycloaddition step—Scheme 277 [308].

Unfortunately, anticancer activity evaluation of the obtained compounds against four human cancer cell lines showed weak activities of the tested agents. However, the bis-3-acetylisoxazoline showed the most potent cytotoxic activity against MCF-7 and MDA-MB-231 cell lines, while the docking studies revealed that stsooxazolinezoline might have good inhibitory activity against Pim-1 kinase by forming a stable complex. Good ADMET properties and possible oral bioavailability of this compound are also attractive.

**Scheme 278:**
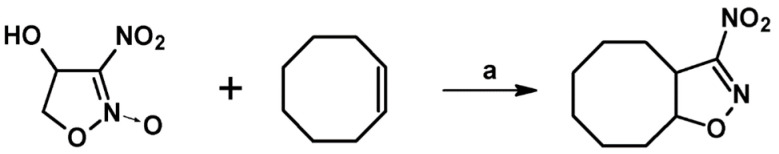
Syntheses of 3,5,5-trisubstituted isoxazolines possessing nitro group in position 3 (an example) [309]. a = DCE, 55 °C, 24 h, then NaHCO_3_, benzene/H_2_O, 25 °C, 0.5 h; 62% yield.

3,5,5-trisubstituted isoxazolines possessing a NO_2_ group in position 3 cannot be obtained via 1,3-DP cycloaddition, but recently, an effective method dedicated to the preparation of such isoxazolines was presented by Sedenakova and coworkers in their article from 2022—Scheme 278 [309].

### 5.2. From Nitronates

**Scheme 279:**
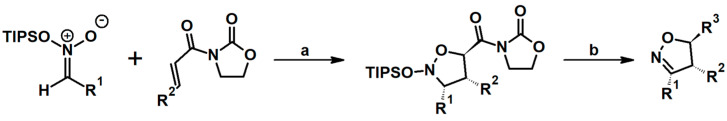
Two-step syntheses of chiral isoxazolines: isoxazolidines formation via cycloaddition of nitronates to unsaturated imides followed by C=N creation via TIPSOH elimination [310]. R^1^ = H, alkyl; R^2^ = H, COOMe; R^3^ = CH_2_COO(*p*-BrC_6_H_4_); a = Cu(II)-Chiral-Bisisoxazoline, −50 °C or −15 °C, 12–24 h; b = PTSA, CHCl_3_, –0 °C—rt; isoxazoline yields up to 100%; *ee* (isoxazolines) = 70–92%.

In 2015, chiral 3,5-di- and 3,4,5-trisubstituted isoxazolines were synthesized via 1,3-DP cycloadditions, mediated by Cu(OTf)_2_-chiral BOX ligand-catalysts, of triisopropylsilyl nitronates to α,β-unsaturated carboximides followed by TIPSOH elimination catalyzed by PTSA—Scheme 279 [310].

**Scheme 280:**
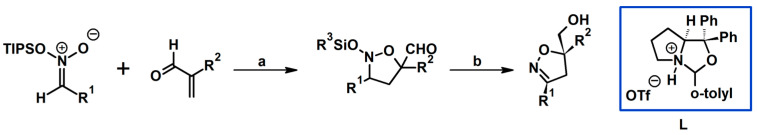
Enantioselective syntheses of isoxazolines from nitronates [311]. a = L, PhMe, −60 °C, 12 h; b = NaBH_4_, MeOH, −60 °C to rt; up to >99% yield; *ee* = up to 96% (16 examples).

The following report from 2015 describes chiral 3,5,5-trisubstituted isoxazolines bearing a CH_2_OH group in position 5 synthesized via 1,3–DP cycloadditions of triisopropylsilyl nitronates and 2–alkylacroleins involving a chiral oxazaborolidine as catalyst—Scheme 280 [311].

Interestingly, one of the obtained isoxazolines was converted into (*R*)-(*+*)-Tanikolide in nine steps and with a total yield of 43%.

**Scheme 281:**
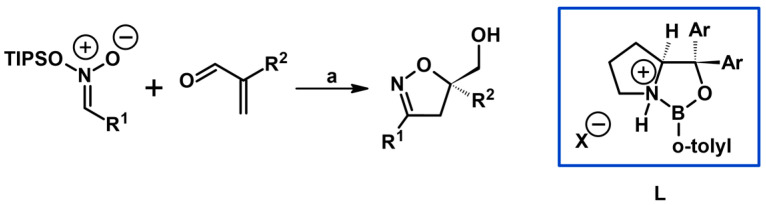
Syntheses of 3,5,5-trisubstituted 2-isoxazolines from silyl nitronates [312]. a = 10 or 15mol% cat., −60 or −50 °C, PhMe, 12 h next NaBH_4_, MeOH, from −60 or −50 °C to rt; up to >99% yield; *ee* = up to 96%.

Two years later, researchers reported that triisopropylnitronates, 2-alkylacroleins, a chiral oxazaborolidine catalyst, and 1,3-DP cycloadditions were involved in the synthetic route leading to isoxazolines with high yields and high enantioselectivities—Scheme 281 [312].

**Scheme 282:**
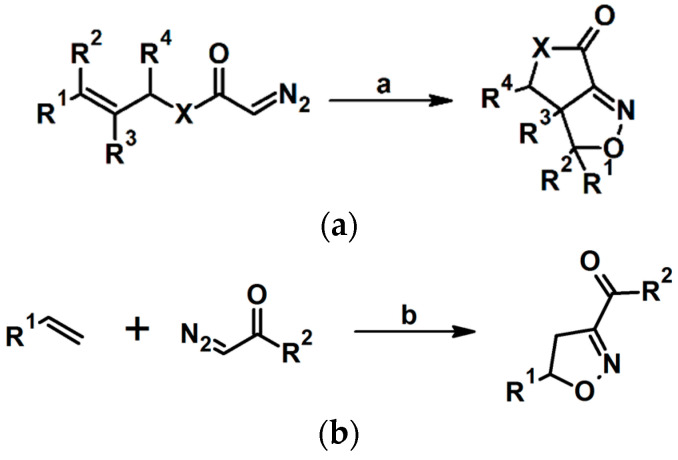
Intra- (**a**) and intermolecular (**b**) transformation leading from nitronates to isoxazolines [313]. (**a**) a = *t*-BuONO, DMF, rt; up to 92% yield; (**b**) b = *t*-BuONO, BF_3_·Et_2_O, MeCN, rt, 12 h; up to 92% yield; ((**a**,**b**): more than 70 examples).

In an edge article from 2021, Ma et al. described intra- and intermolecular acyclic nitronate olefin cycloaddition (ANOC) reactions that enable the highly efficient syntheses of isoxazolines bearing various functional groups—Scheme 282 [313].

Both experimental results and DFT calculations indicate that these transformations proceed via the in situ formation of acyclic nitronates together with concerted [3 + 2] cycloaddition and, finally, *tert*-butyloxy group elimination.

**Scheme 283:**
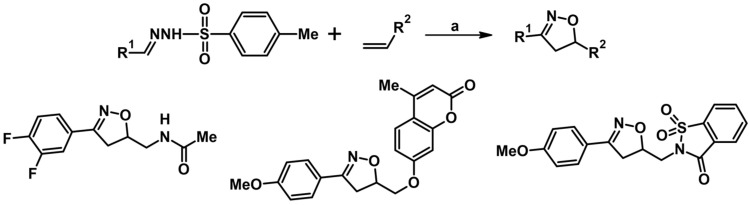
Syntheses of 3,5-disubstituted isoxazolines from in situ-generated nitronates [314]. Examples of biologically important compounds. a = *t*-BuONO, CuCl_2_, TMEDA, THF, 65 °C, 24 h, air; up to 88% yield (73 examples).

In the following edge article from 2021, a new type of CuCl_2_-catalyzed [2 + 2 + 1] cycloaddition leading to isoxazolines was presented—Scheme 283 [314]. Significantly, the RCNOs are not involved in the reaction method.

This process’s advantages are as follows: a broad substrate scope, nearly universal functional group compatibility, tolerance of moisture and air, and readiness to scale up. Experiments and theoretical calculations indicate that this transformation proceeds via the in situ generation of a nitronate from the coupling of *N*-tosylhydrazone and TBN, followed by cycloaddition to the carbon–carbon double bond, and finally, elimination of a *tert*-butyloxy group to give the desired isoxazoline.

**Scheme 284:**
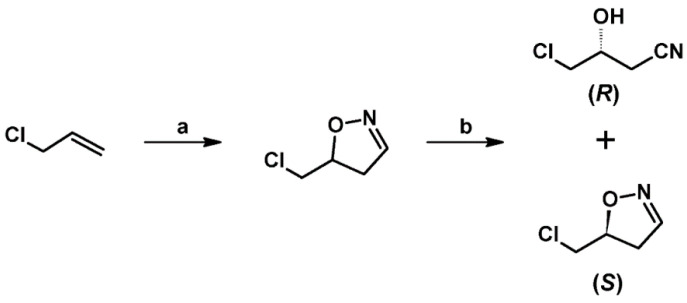
Synthesis of (*S*)-chloromethylisoxazoline from allyl chloride engaging enzymatic resolution [315]. a = Me_3_SiCl, Et_3_N, CH_2_Cl_2_, 50 °C, 48 h; 22% yield; b = enzyme (OxdA-L318I or lyophilized whole cell of OxdA-L318I), 3h; 30% yield; *ee* = 99%.

Zheng and Asano reported the synthesis of chiral 2-chloromethyl isoxazoline via intramolecular cyclization of in situ-generated allyl nitronate followed by enzymatic resolution of obtained racemic isoxazoline—Scheme 284 [315]. In the next step, the obtained isoxazoline was smoothly transformed into (*S*)-3-chloro-2-hydrohybutanonitrile, essential for organic synthesis, with high *ee*.

**Scheme 285:**
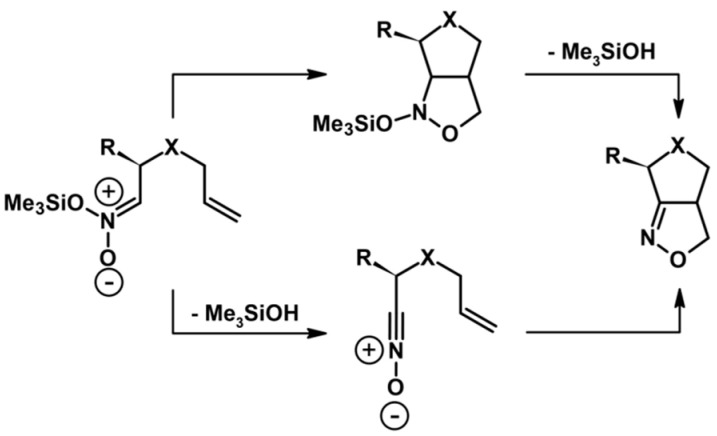
Syntheses of isoxazolines fused with 5-membered S- or O-containing saturated ring [316]. R, X = Me, O; Me, S; *i*-Pr, S; Ph, S; NO_2_, S.

The final report from 2021 describes the obtention of bicyclic isoxazolines belonging to the 3,4-disubstituted isoxazolines from unsaturated *N*-trialkylsilyloxy nitronates via highly stereoselective domino reaction—Scheme 285 [316].

DFT calculations with the ωB97XD functional clearly indicated that this reaction proceeds via a domino process, including two pseudocyclic elemental reactions, namely nitronate-[3 + 2] cycloaddition and the elimination of trimethylsilanol.

### 5.3. From β-Carboxy-Substituted α,β-Unsaturated Ketones via Cascade Oxa-Michael-Cyclization

**Scheme 286:**
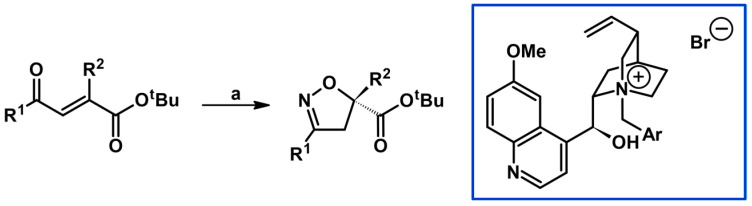
Syntheses of chiral isoxazolines applying chiral PTC catalyst and cascade oxa-Michael-cyclization of hydroxylamine with β-carboxy-substituted α,β-unsaturated ketones [317]. Ar (in catalyst) = 2,5-(F_3_C)_2_C_6_H_3_; R^1^, R^2^ = Ph, Me; 3-thienyl, Me; 2-naphthyl, Me; *n*-Pr, Ph, PhCH_2_CH_2_CH_2_; Ph, BnSCH_2_CH_2_CH and others; a = NH_2_OH, Cs_2_CO_3_, MTBE, −30 °C, 24 h; up to 98% yield; *ee* = up to 96%.

In 2018, Lee et al. published an article regarding chiral 3,5,5-trisubstituted isoxazolines bearing a *t*-BuOOC moiety in position 5 and a synthetic route towards it using chiral quinidine-based PTC in cascade oxa-Michael-cyclization of hydroxylamine with various β-carboxy-substituted α,β-unsaturated ketones—Scheme 286 [317]. Importantly, this procedure was successfully applied to synthesize herbicide, namely (*S*)-methiozolin, on a large scale.

**Scheme 287:**
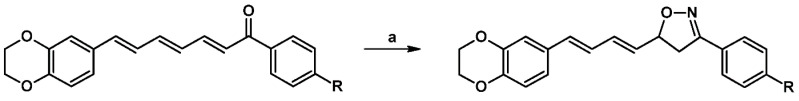
Intramolecular 1,3-DP cycloaddition engaged in the syntheses of piperine analogues [318]. R = F or Cl; a = NH_2_OH·HCl, CH_2_Cl_2_, next NaOH, EtOH, from rt to ~65 °C, 9–12 h, 36% yield (for R = Cl) and 45% yield (for R = F).

Intramolecular 1,3-DP cycloaddition of a CNO motif to the double bond, both coming from the same molecule, was applied by Yang and colleagues in the syntheses of 3,5-disubstituted isoxazolines bearing the special arene-diene motif in position 5—Scheme 287 [318].

Biological examination showed that the obtained isoxazolines are characterized by higher oral toxicity against *P. xylostella* than toosendanin, and both displayed more promising growth-inhibitory activity against *M. separata* than toosendanin. The methylenedioxy and isoxazoline scaffolds were also necessary for oral toxicity and growth-inhibitory activity against *P. xylostella* and *M. separata*, respectively. Interestingly, the above-mentioned motifs were vital for the acaricidal activity against *T. cinnabarinus*.

**Scheme 288:**
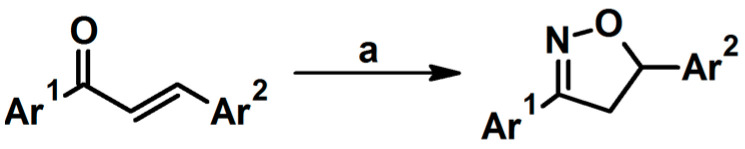
Synthesis of 3,5-disubstituted isoxazolines from Ar^1^COCH=CHAr^2^ [319]. Ar^1^, Ar^2^ = Ph, substituted Ph, naphthyl, heteroaryl; a = NH_2_OH or NH_2_OH·HCl, solvent (EtOH, AcOH, and others), various conditions.

In the review from 2022, the recent advances in the synthesis of isoxazolines from Ar^1^COCH=CHAr^2^ are presented—Scheme 288 [319].

### 5.4. From Oximes (Simple Oximes, Unsaturated and Oxo-Oximes)

Several methods have been developed in recent years to synthesize isoxazolines from oximes without their transformation into RCNO. Recent advances in the synthesis of isoxazolines with oxime participation were presented in the review given by Liao et al. [320]. Selected works before 2020 and all those published after 2020, i.e., after the aforementioned publication, are briefly described below.

**Scheme 289:**
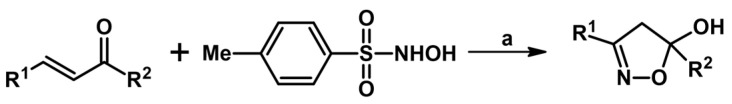
Syntheses of 5-hydroxy-2-isoxasolines from oxo-oximes [321]. R^1^, R^2^ = Ph, Me; Ph, Ph; 2-furyl, Ph; styryl, Ph; *p*-MeOC_6_H_4_, Ph; Ph, *p*-O_2_NC_6_H_4_; and others. a = K_2_CO_3_, MeOH/H_2_O, 35 °C, 24 h; up to 92% yield.

In 2010, 5-hydroxy-2-isoxazolines were obtained via highly regio- and stereoselective intramolecular cyclization of in situ-generated ß-oxo-oximes—Scheme 289 [321].

The method presented above is specific for synthesizing 5-hydroxyisoxazolines, but according to the authors, it is more effective and sustainable compared to 1,3-DP cycloaddition of RCNO to the appropriate dipolarophiles.

**Scheme 290:**
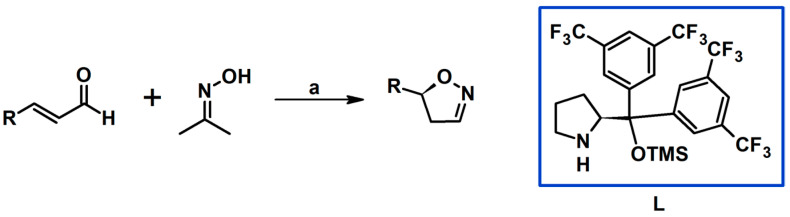
Syntheses of chiral 3,5-disubstituted isoxazolines via cyclization of in situ-generated oximes mediated by the chiral ligand [322]. R = PhH_2_CH_2_, *n*-C_5_H_11_ a = L, PhCOOH, PhMe, 0 °C; 2. MeOH/H_2_SO_4_; BnOCH_2_, TBDPSOCH_2_CH_2_CH_2_CH_2_, and others; up to 71% yield; *ee* = up to 95%.

In the same year, the obtention of chiral, 3-substituted 2-isoxazolines via enantioselective cyclization of in situ-generated oximes in the presence of a chiral ligand was reported by Pohjakallio and colleagues—Scheme 290 [322]. The obtained isoxazolines can be smoothly transformed into ß–hydroxynitrile—see chapter 6.

**Scheme 291:**
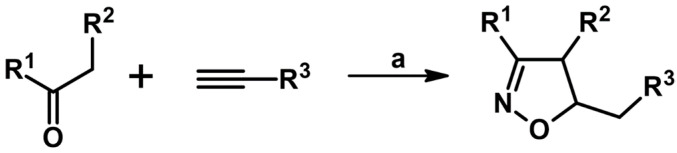
Syntheses of 3,4,5-trisubstituted isoxazolines via KOH-mediated cyclization of in situ-generated α,ß-unsaturated oximes [323]. R^1^, R^2^ = alkyl, cycloalkyl, or aryl; R^3^ = aryl; a = (1) KOBu*t*, DMSO, 100 °C, 30 min; (2) H_2_O, NH_2_OH·HCl, 70 °C, 1.5–3.5 h; 3. KOH, 70 °C, 30 min; up to 88% yield.

As presented in an article from 2013, 3,4,5-trisubstituted isoxazolines can be smoothly synthesized from in situ-generated oximes using ketones, arylacetylenes, and NH_2_OH·HCl and applying the one-pot procedure—Scheme 291 [323].

Recently, the main steps of the reaction mechanism presented above were studied in terms of quantum chemistry—see the comment in Section 3 [292].

**Scheme 292:**
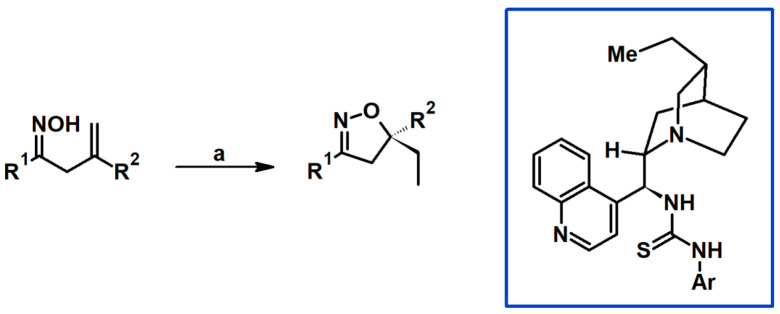
Cyclization of β,*γ*-unsaturated ketoximes using NIS: the furnishing of D2–isoxazolines containing a quaternary stereogenic center [324]. a = NIS, I_2_, L, PhMe + CHCl_3_, −78 °C; up to 91% yield; *ee* = up to 98.5%.

Chiral isoxazolines were also obtained in 2013 using a bifunctional thiourea catalyst in highly enantioselective cyclization-iodoetherification of β,γ-unsaturated oximes by Tripathi and Mukherjee—Scheme 292 [324].

**Scheme 293:**
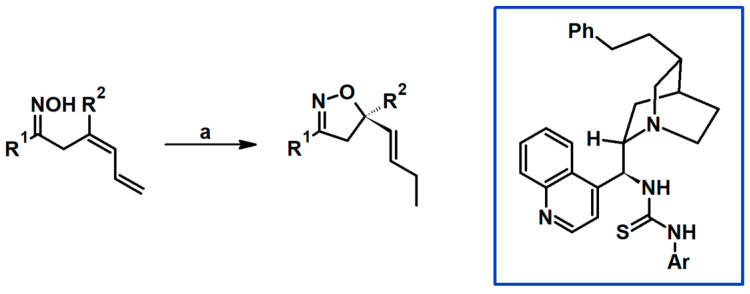
Synthesis of chiral 3,5,5-trisubstituted isoxazolines starting from β,γ,δ,ε-unsaturated oximes [325]. a = catalyst, NIS, PhMe, CH_2_Cl_2_, −80 °C, MS 4Å, 72 h; 99% yield; *ee* = up to 99%.

In the following paper, the same authors described chiral isoxazolines obtained via catalytic enantioselective, cyclization-1,4-iodofunctionalizations of β,γ,δ,ε-unsaturated oximes by NIS and aminothiourea derivatives as chiral catalysts—Scheme 293 [325].

**Scheme 294:**
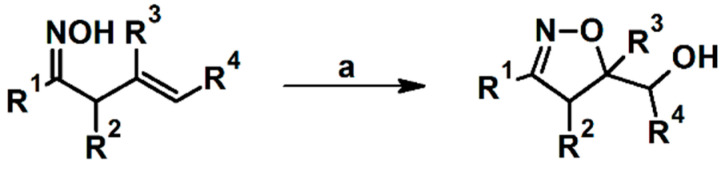
Syntheses of isoxazolines from allyloximes: applying 2,2,2-trifluoroacetophenone as organocatalyst and H_2_O_2_ as the green oxidant [326]. a = H_2_O_2_, PhCOCF_3_, MeOH, *t*-BuOH, aq. buffer, rt, 18 h; up to 93% yield (23 examples).

In an article from 2017, Triandafillidi and Kokotos reported synthesizing 3,5-disubstituted isoxazolines from allyloximes involving a green, sustainable, organocatalytic, and efficient environmentally friendly protocol—Scheme 294 [326].

In 2018, Drosik and Suwiński published a critical review devoted to obtaining 2-isoxazolines from unsaturated oximes via various types of cyclization [327].

**Scheme 295:**
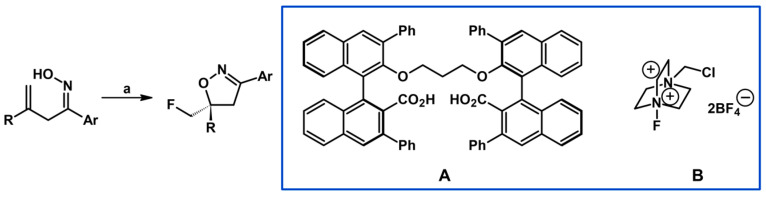
Syntheses of chiral 3,5,5-trisubstituted isoxazolines bearing FCH_2_ moiety in position 5 [328]. a = precatalyst (**A**), selectfluor (**B**), Na_3_PO_4_, PhMe, 15 °C, 72 h; up to 68% yield; *ee* = up to 84%.

Researchers in 2019 used a chiral PTC catalyst for the preparation of chiral isoxazolines via the enantioselective 5-exo-fluorocyclization of ene-oxime—Scheme 295 [328].

**Scheme 296:**
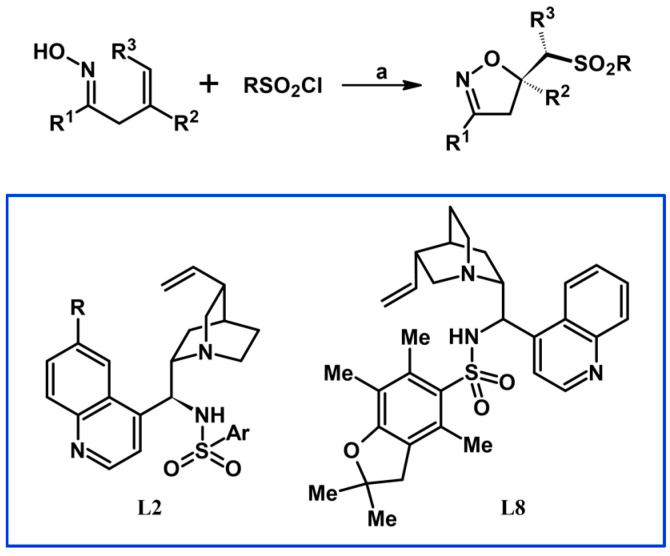
Syntheses of chiral 3,5,5–isoxazolines equipped with sulfonyl motif in position 5 [329]. a = Cu(OAc)_2_, L1: R = H, Ar = 2,3,5,6–(CH_2_)_4_-Ph; or L8, −10 or 0 °C, 96 h, CHCl_3_, MS 4Å; up to 95% yield.

The following year, the sulfonyl-containing 3,5,5-trisubstituted chiral isoxazolines were obtained in the asymmetric radical oxysulfonylation of the C–C double bond in ß,γ-unsaturated ketoximes using chiral copper(I)-cinchona alkaloid-based sulfonamide complex—Scheme 296 [329].

The obtained sulfonyl-containing isoxazolines could undergo further transformations to provide valuable building blocks prevalent in numerous bioactive molecules. Interestingly, this strategy would open a new door for precisely controlling diastereo- and enantioselectivities in the challenging asymmetric radical-initiated difunctionalization of internal alkenes.

**Scheme 297:**
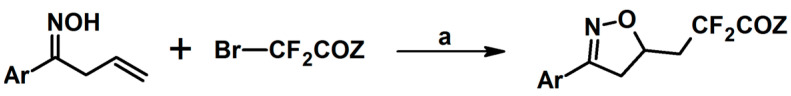
Syntheses of isoxazolines bearing CF_2_COZ group in position 5 from oximes via cascade: difluoroalkylation of C–C double bond followed by nucleophilic attack of the hydroxyl group coming from β,γ-unsaturated oximes [330]. Ar = Ph, substituted-Ph, and others; Z = n–BuNH, Et_2_N, BnBH, EtO, N-morpholinyl, and others; a = CuI/PMDETA, NaHCO_3_, Na_2_S_2_O_8_, DMSO, 110 °C; up to 73% yield (24 examples).

In the same year, a team of researchers described in their work isoxazolines synthesized via a copper-catalyzed difluoroalkylation of β,γ-unsaturated oximes, followed by intramolecular nucleophilic attack of the hydroxyl group of oximes—Scheme 297 [330].

**Scheme 298:**
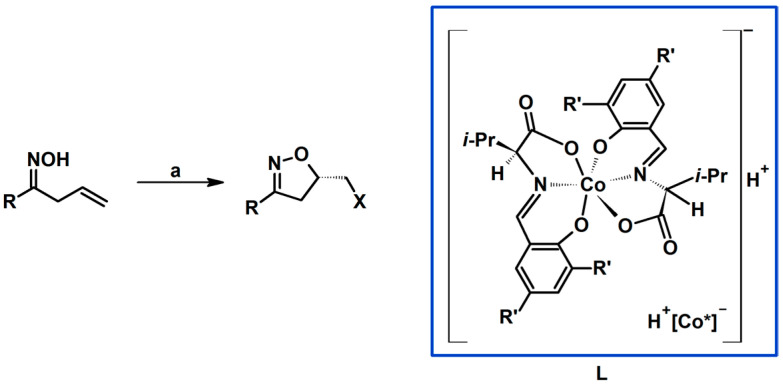
Syntheses of 3,5-disubstituted isoxazolines bearing CH_2_Br or CH_2_I moiety in position 5 [331]. a = NBAc or NBS (for Br) or DIDMH (for I), L, MS 5Å, solvent; X = Br or I; up to 99% yield (16 examples); *ee* = up to 95.5%, * = chiral complex.

Another team in the same year used Brønsted acids of anionic chiral Co(III) complexes as catalysts in the synthesis of chiral 5-halomethyl isoxazolines. The products, which bear a tertiary stereocenter, were obtained by catalytic halocyclization of β,γ-unsaturated ketoximes—Scheme 298 [331].

Moreover, preliminary tests indicated that some obtained isoxazolines exhibited significant antifungal activities.

**Scheme 299:**
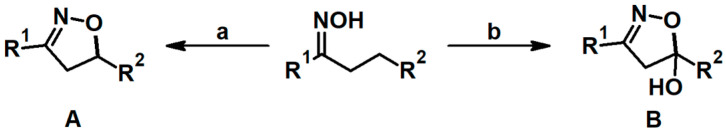
Syntheses of 3,5-disubstituted isoxazolines via aliphatic δ-C(sp^3^)-H bond oxidation of oximes [332]. a = TEMPO, K_2_S_2_O_8_, MeCN, rt to 50 °C, 24 h to 48 h; b = TEMPO, PhI(OAc)_2_, MeCN, rt, N_2_, 24 h; up to 81% yield (for A), up to 89% yield (for B).

3,5-di- and 3,5,5-trisubstituted 2-isoxazolines, including 5-hydroxy-2-isoxazolines, were prepared from oximes using inexpensive inorganic reagents, namely TEMPO and K_2_S_2_O_8_ or TEMPO and PhI(OAc)_2_ as oxidation systems—as reported in an article from 2021 (Scheme 299) [332].

**Scheme 300:**
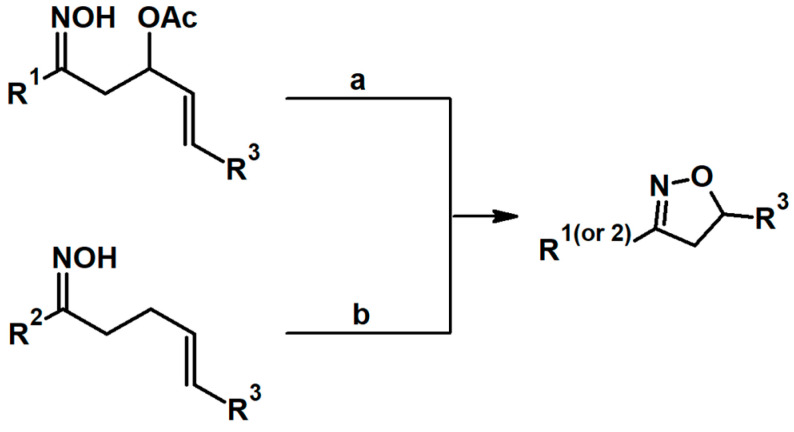
Pd-Ag-mediated syntheses of 3,5-disubstituted isoxazolines from unsaturated oximes [333]. R^1^, R^2^ = aryl, cyclopropyl; R^3^ = H or Me; a (by leaving group ionization) = [Pd(PPh_3_)_4_], K_2_CO_3_; b (by allylic C-H activation) = Pd(AcOEt)_2_, Ag_2_CO_3_, up to 95% yield.

The same year, Fernandes, Gangani, and Panja reported synthesizing 3,5-disubstituted isoxazolines bearing CH=CHR group in position 5 via palladium-catalyzed intramolecular *O*-allylation of ketoximes—Scheme 300 [333].

**Scheme 301:**
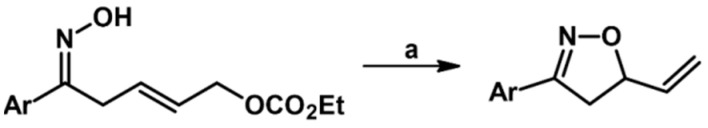
Syntheses of chiral 3,5-disubstituted 2-isoxazolines via *Ir*-complex-mediated intramolecular substitution [334]. a = [Ir(cod)Cl]_2_ + L (chiral BINOL-derived phosphoramidite ligands), K_2_CO_3_, CH_2_Cl_2_, rt, 20 h; Ar = Ph, *p*-ClC_6_H_4_, *p*-BrC_6_H_4_, *p*-MeOC_6_H_4_, 2-naphthyl, 2-thienyl, and others; up to 99% yield; *ee* = up to 95%.

Recently, efficient routes leading to chiral isoxazolines from unsaturated oximes via intramolecular substitution catalyzed by iridium complexes with chiral BINOL-derived phosphoramidite ligands were described by Hu and colleagues—Scheme 301 [334].

**Scheme 302:**
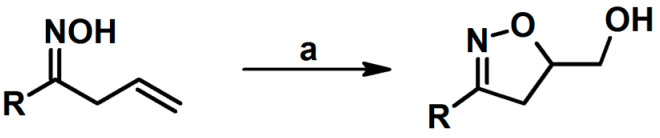
Synthesis of 3,5-disubstituted isoxazolines from unsaturated oximes (22 examples) [335]. R = aryl, hetaryl, (the most often), cyclopentyl, cyclohexyl, benzyl; a = *g*-C_3_N_4_, air, H_2_O/MeCN, LED; 31–76% yield.

In February 2022, Fu et al. published a fascinating synthetic method of obtaining 5-hydroxymethyl-2-isoxazolines via visible-light-promoted recyclable-carbon-nitride-catalyzed dioxygenation of *β*,*γ*-unsaturated oximes—Scheme 302 [335].

The one-pot methodology presented above is undoubtedly feasible and attractive for constructing a large variety of 3-aryl-5-hydroxymethyl isoxazolines from unsaturated oximes in the presence of *g*-C_3_N_4_ under blue light irradiation conditions. Notably, the reaction could proceed smoothly only using commercially available and cheap *g*-C_3_N_4_ as the photocatalyst; NaHCO_3_ as a base is necessary, but other reagents, for instance, an oxidant or reductant, are not required. The eminent advantages of this work also include step-economy, recyclable catalyst, and mild reaction condition. A proposed reaction mechanism has also been presented.

**Scheme 303:**
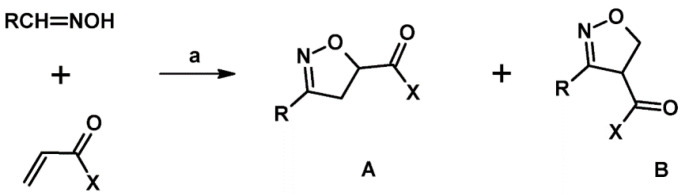
Electrochemical synthesis of isoxazolines from oximes and acrylates [336]. SS—stainless steel electrode, HFIP—hexafluoroisopropanol. R = alkyl, aryl, pyridine; X = OMe, O*t*Bu; a = Et_4_NCl, HFIP, MeCN, C(+)/SS(−), 25 mA, 3 F mol^−1^, (**A**,**B**) = regioisomers.

Very recently, a broadly applicable electrochemical methodology for the synthesis of 3,5-disubstituted isoxazolines that is quantifiably greener than comparable non-electrochemical methods was published—Scheme 303 [336].

The method is characterized by short reaction times, minimal waste generation, and avoiding toxic or expensive oxidizing reagents, and both aromatic (including heteroaromatic ones) and alkyl substrates are tolerated. It also enables easy control and monitoring of the process and increases its scale (towards manufacture). Thus, what is worth noting is that this methodology meets all twelve principles of green chemistry. Profound mechanistic studies supported by DFT calculations were also performed, and for the reader’s convenience, we presented them here. The DFT/M06-2X level of theory was used to model possible reaction pathways including the stepwise radical-mediated path and the concerted [3 + 2] cycloaddition path. The kinetic modelling and calculations on the level of density functional theory support a radical mechanism and disfavor the hypothesized involvement of a closed-shell [3 + 2] cycloaddition mechanism. The stepwise radical path of the reaction is consistent with experimental observations of diastereoselectivity, whereas in the presence of a [3 + 2] cycloaddition mechanism, the reaction should be stereoretentive. Moreover, the [3 + 2] cycloaddition path is characterized by energetically unfavorable barriers, particularly the predicted step initiating the reaction, i.e., *N*-chlorination of the oxime and subsequent E2 elimination, has a very high energy barrier, which has been theoretically estimated at almost 200 kcal/mol. Instead, the stepwise radical pathway consistently has about a 2.5 times lower C–C bond formation barrier between the oxime radical and the disubstituted alkene compared to the energy barrier of the one-step ring closure in the [3 + 2] cycloaddition pathway. According to the theoretical model of the electrochemical synthesis of isoxazolines, that reaction is highly likely initiated by oxidation on the anode of the chloride, derived from the Et_4_NCl additive. The emerging chlorine radical subsequently participates in the abstraction of the hydrogen atom from the oxime, generating the nucleophilic hydroxyimoyl radical. This radical reacts with the acceptor, forming the C–C bond of the isoxazoline product. With less probability, it is also allowed that the reaction can be initiated by direct electrochemical oxidation of the oxime to its radical cation.

### 5.5. From Organometallics (Allyl–Metal)

**Scheme 304:**
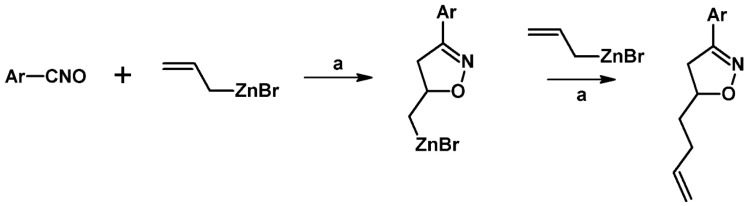
Allylzinc bromide in the syntheses of 3-aryl-5-(3-butenyl)-2-isoxazolines [337]. Ar = Ph, *p*-MeOPh, 2,6-Cl_2_C_6_H_3_, *p*-Me_2_NC_6_H_4_; and others; a = THF, up to rt, 12–14 h; up to 82% yield.

In 2005, a team of researchers described synthesizing 3,5-disubstituted isoxazolines bearing a 3-butenyl moiety in position 5 via regioselective domino 1,3-DP cycloaddition –nucleophilic addition of allylzinc bromide to ArCNO—Scheme 304 [337].

**Scheme 305:**
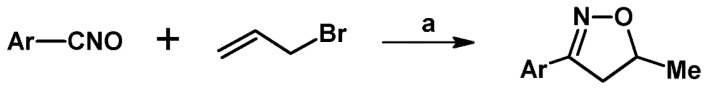
Syntheses of 3-aryl-5-methyl-2-isoxazolines starting from the addition of in situ-generated allylindium to nitrile oxides [60]. Ar = Ph, 2,6-Cl_2_C_6_H_3_, *p*-MeC_6_H_4_, 2-Cl-5-O_2_NC_6_H_3_, *p*-MeOC_6_H_4_, and others; a = In-powder, THF/H_2_O; up to 81% yield.

Two years later, a synthesis of 3-aryl-5-Methylisoxazolines was reported. It was realized by highly selective nucleophilic addition of in situ-generated allylindium reagent to PhCNO and to its substituted derivatives followed by cyclization and protonation—Scheme 305 [60].

**Scheme 306:**
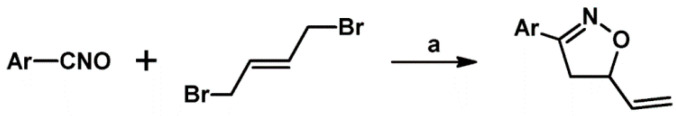
Syntheses of 2-vinyl isoxazolines: domino nucleophilic addition-anionic C–O-heterocyclization *In*- or *Mg*-allyl organometallics reacting with ArCNO [338]. Ar = Ph, *p*-MeOC_6_H_4_, *m*-O_2_NC_6_H_4_, *p*-FC_6_H_4_, 2-naphthyl; and others; a = In or Mg, THF; reaction time = up to 5 h (Mg) or up to 45 h (In); up to 85% yield (in every case higher for Mg).

In the same year, reactions were reported between ArCNO (typically generated in situ from oxymoyl chlorides) and organometallics generated in situ from (*E*)-1,4-dibromobut-2-ene and metallic indium or magnesium that produced 5-vinyl 2-isoxazolines—Scheme 306 [338].

**Scheme 307:**
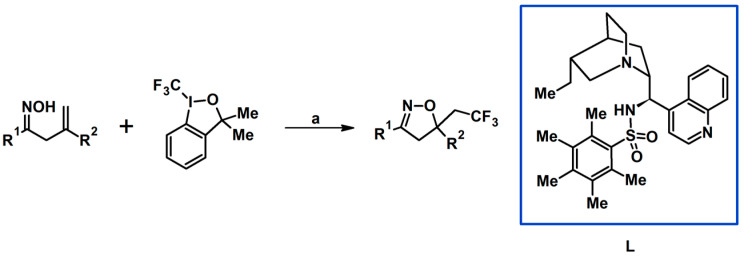
Syntheses of 3,5,5-trisubstituted 2-isoxazolines via *Cu*-chiral ligand complex-mediated asymmetric radical oxytrifluoromethylation of alkenyl oximes [339]. a = Cu(OAc)_2_, L, MS 4Å, CHCl_3_; up to 99% yield; *ee* = up to 92%.

Over a decade later, copper complexes with cinchona-alkaloid-based sulfonamides were reported to be effective catalysts in the asymmetric radical oxytrifluoromethylation of alkenyl oximes leading to CF_3_-containing 3,5,5-trisubstituted 2-isoxazolines possessing an α-tertiary stereocenter with excellent yield and enantioselectivity—Scheme 307 [339].

**Scheme 308:**
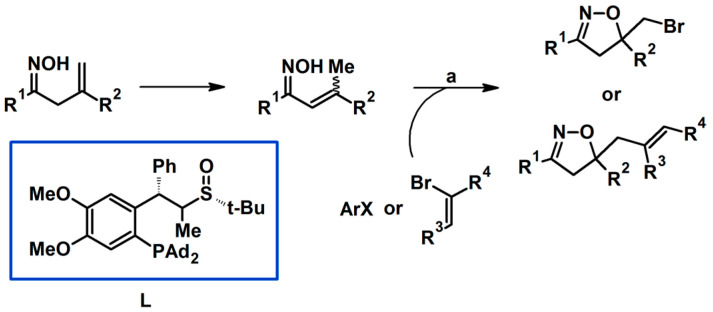
Syntheses of 3,5-di- and 3,5,5-trisubstituted 2-isoxazolines involving Pd/L-catalyzed enantioselective carboetherification of alkenyl oximes with either aryl or alkenyl halides [340]. a = 3 mol% [Pd_2_(dba)_3_], 10 mol% L, *t*-BuONa, 65–90 °C, PhMe; up to 95% yield; *ee* = up to 97%.

In work from 2020, a highly efficient, *Pd*-chiral-ligand-catalyzed, enantioselective carboetherification of alkenyl oximes with either aryl or alkenyl halides was applied for obtaining chiral 3,5-di- and 3,5,5-trisubstituted 2-isoxazolines in good yields and enantioselectivity—Scheme 308 [340].

Importantly, the transformations presented above are characterized by good functional group tolerance and can be realized in mild reaction conditions. Moreover, scale-up and application in the late-stage modification of bioactive compounds is easy. The obtained isoxazolines can be readily transformed into chiral 1,3-aminoalcohols useful for pharmaceutical syntheses.

### 5.6. From Alkenes, In Situ-Generated Carbenes, and Nitroso Radical

**Scheme 309:**
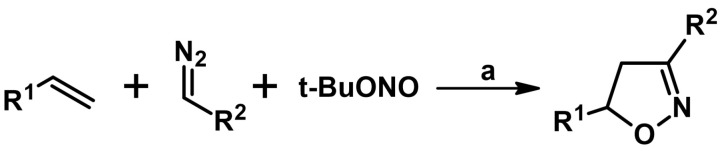
Coupling reaction of Cu-based carbene and nitroso radical: a tandem reaction to construct isoxazolines [341]. a = Cu(AcO)_2_·H_2_O, DABCO, PhMe, 12 h, 80 °C; up to 96% yield.

Isoxazolines were synthesized via cross-coupling reaction between copper carbene and nitroso radical. The reported synthesis procedure from 2017 is characterized by mild reaction conditions, broad substrate scope, and simple procedures—shown in Scheme 309 [341].

Notably, in this radical–carbene coupling reaction (RCC reaction), the construction of C−C, C−O, and C=N bonds takes place in a one-pot process.

### 5.7. From Alkenes, Diazocompounds and t–BuONO

**Scheme 310:**
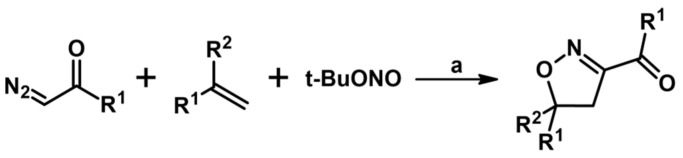
Electrosynthesis of isoxazolines: intermolecular free radical [2 + 1 + 2] annulation of alkenes with *t*-BuONO and diazo compounds as radical acceptors [342]. a = C(+)/Pt(–); I = 10 mA, [*n*-Bu_4_N]BF_4_, HFIP, CH_2_Cl_2_, undivided cell, N_2_, rt, 1 h; up to 75% yield.

In 2019, 3,5-di- and 3,5,5-trisubstituted isoxazolines were obtained through the electrochemical intermolecular annulation of alkenes with *tert*-butyl nitrite and diazo compounds as radical acceptors—Scheme 310 [342].

In this paper, the authors presented a method of obtaining isoxazolines based on organic electrosynthesis. Replacement of the toxic or dangerous oxidant by electrochemical oxidation is eco-friendly and allows easy scale-up of the process. Interestingly, the described method is more versatile, and after replacing the vinyl substrate with an allyl derivative, one can obtain 1,2-oxazines.

### 5.8. From R^2^–C≡C–CH(R^1^)O–NH_2_

**Scheme 311:**
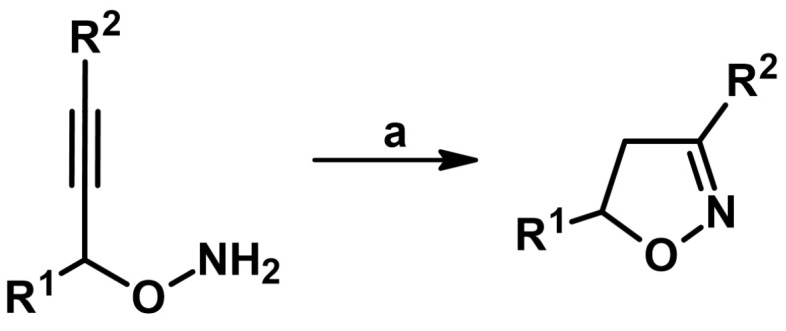
Synthesis of disubstituted isoxazolines from propargylamines [343]. R^1^ = alkyl, Ph; R^2^ = H, alkyl, aryl; a = Ag(NO_3_)-SiO_2_, CH_2_Cl_2_, rt, 0.25 h; up to 96% yield.

As stated in an article from 2018, 3,5-disubstituted isoxazolines can also be obtained via Ag(I)-mediated propargylamines transformation—Scheme 311 [343].

### 5.9. From Isoxazoles

**Scheme 312:**
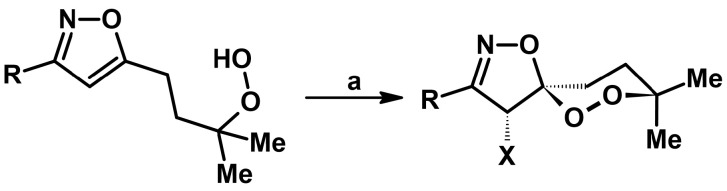
Transformation of spiro-isoxazoles into halogenated hybrid spiro-peroxide-isoxazolines [344]. R = alkyl, aryl, ester motif; X = Br or Cl; a = PIDA/ZnX_2_; up to 95% yield.

In 2019, a group of researchers published a work regarding biologically relevant chiral isoxazolines bearing a spiro-peroxide motif. Those chemical species were obtained via reduction halogenation of appropriate isoxazoles—Scheme 312 [344].

In conclusion, new methods of isoxazoline synthesis (other than those based on 1,3-DP cycloaddition of RCNO to the carbon–carbon double bond) have been emerging, especially in recent years. They are an excellent complementing synthetic tool to the possibility of synthesizing isoxazolines using RCNO, and, above all, they allow the synthesis of systems inaccessible to simple dipolar cycloaddition.

## 6. 2-Isoxazolines in Organic Synthesis

**Figure 17:**
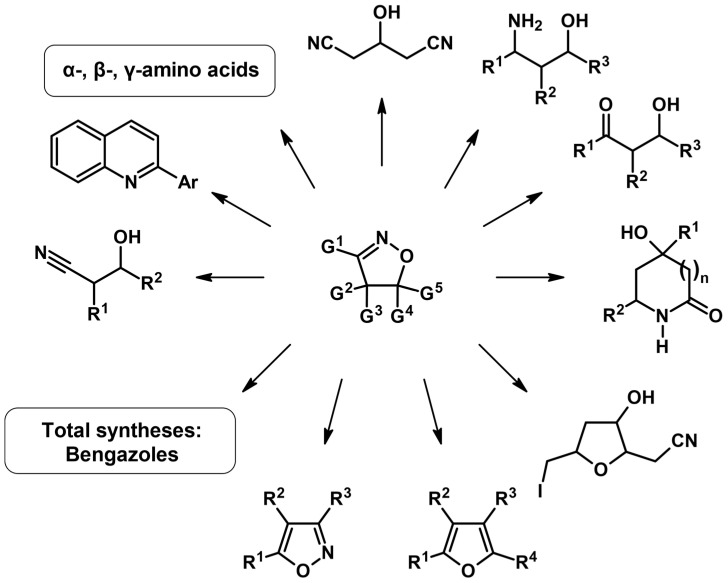
2-Isoxazolines in organic synthesis—selected examples [22,54,133,163,175,301,315,321,322,345,346,347,348,349,350,351,352].

Functionalized isoxazolines are highly important in organic and biomedical chemistry, which is fully confirmed by numerous literature reports. They are also attractive intermediates used in organic synthesis, for example, as precursors of such organic compounds as isoxazoles, β-lactams, ß- and γ-aminoalcohols, β hydroxyketones, substituted furanes, β-hydroxynitriles, 2-arylquinolines, α-hydroxylactams, and others. Figure 17 presents the most significant applications of isoxazolines in synthesizing various classes of organic compounds. When discussing the synthesis of isoxazolines in our review (see Section 2), we always tried to show applications in organic synthesis, especially if not the synthesis, but further transformations were the authors’ goal. The diagram below shows selected, not all, possibilities of transforming isoxazolines into various types of organic compounds.

To sum up this short fragment of our review: undoubtedly, isoxazolines are valuable organic semiproducts, scaffolds and syntons, and numerous examples of the use of isoxazolines in organic synthesis can be found in the reviews and books [22,54,133,163,175,301,315,321,322,345,346,347,348,349,350,351,352].

## 7. 2-Isoxazolines—Biological Activity

We are aware of the enormous impact that 2-isoxazolines bear due to their vast and varied biological and pharmacological activity, which is covered by most publications dedicated to this class of compounds. Therefore, when discussing the synthesis of isoxazolines, we always paid attention to this aspect of the presented works, i.e., the purpose for which the isoxazolines were designed and synthesized. However, our review does not include a deeper analysis of the biological activity of isoxazolines. In particular, we do not discuss in depth the evaluation results of their biological activity. Therefore, we quote below the most significant review from recent years, comprehensively and deeply discussing all aspects of the biological activity and pharmacological applications (in humans, animals, and plants) of isoxazolines. For instance, 2-isoxazoline has emerged as one of the most important frameworks for drugs such as lotilaner, fluralaner, sarolaner, and afoxolaner given their clinical use in treating various disorders. Notably, as the isoxazoline scaffold has been associated with a wide range of activities such as anti-inflammatory, anticancer, antituberculosis, antimalarial, and antimicrobial, among many others, there is a significant possibility of enhancing the biological profile of natural products utilizing synthetic modifications with this pharmacophore. As we have already mentioned, the importance of 2-isoxazolines for medicinal chemistry, pharmacy, and agriculture has been thoroughly and deeply discussed in review papers [301,353,354].

In the review from 2014, isoxazolines are commented on as an important class of nitrogen- and oxygen-containing heterocycles that belong to the azoles family, which has gained much importance in the field of medicinal chemistry as anticancer agents [355]. In turn, in the review of Zhou et al. from 2021, the application of isoxazolines in veterinary medicine was presented [356]. In 2021, Itamar et al. discussed isoxazoline-based insecticides, commercially available products, and their design and synthesis via 1,3-DP cycloaddition in their review [357]. This review highlights the utilization of isoxazoline as an insecticide: its mode of action, commercial preparations, and consideration in developing novel insecticides. Similarity analyses were performed with 235 isoxazoline derivatives in three different cheminformatic approaches—chemical property correlations, similarity network, and compound clustering. In general, the cheminformatic methodologies are interesting tools to evaluate the similarity between commercial isoxazolines and clarify the main features explored within their derivatives. In the review from 2018, the significance of oxazole, isoxazole, and their derivatives, including isoxazoline scaffolds, in crop protection chemistry has been analyzed. The main herbicidally, fungicidally, and insecticidally active heterocycles mentioned above are presented with their synthesis routes, modes of action, and biological efficacies [358]. In the review from 2018, Ziemecki et al. presented and commented in depth on the immunoregulatory properties of isoxazole derivatives, including isoxazolines. Among the presented compounds, particular attention was paid to the class of immune stimulators with a potential application in chemotherapy patients [353]. The review given by Agrawal and Mishra is an endeavor to highlight the progress in the chemistry and biological activity of isoxazole derivatives, especially isoxazolines, which could provide a low-height bird’s eye view of isoxazole derivatives to the medicinal chemists for the development of clinically viable drugs using this information [352]. Recently, in the review from 2022, isoxazolines dedicated to veterinary medicine, as a novel class of ectoparasiticides with potent inhibitory activity on the glutamate- and gamma-aminobutyric-acid-gated chloride channel located in the nervous system of invertebrates, are discussed [356]. The article from 2020 reviews the status of isoxazolines concerning labelled uses in dogs and cats in the United States, extralabel clinical use for the treatment of demodicosis in these species, and the safety of orally administered formulations of these drugs. [359]. Recently, the biological efficacy of afoxolaner against the two-spotted spider mite Tetranychus urticae was evaluated. Afoxolaner is a novel representative of the isoxazolines, a class of ectoparasiticides commercialized for controlling tick and flea infestations in dogs. Furthermore, as isoxazolines are known inhibitors of γ-aminobutyric-acid-gated chloride channels (GABACls), the molecular mode of action of afoxolaner on *T. urticae* GABACls (TuRdls) was examined [360]. One part of the book from 2018 entitled “Isoxazolines: Preeminent Ectoparasiticides of the Early Twenty-first Century” is dedicated to isoxazolines as certain drugs [361], and antitubercular and antibacterial activities of isoxazolines derived from natural products were described by Anuchit Phanumartwiwath et al. [362].

## 8. Syntheses of Isoxazolines via 1,3-DP Cycloaddition of Nitrile Oxide—Remaining Challenges

The literature on the synthesis of isoxazolines via 1,3-DP cycloaddition of RCNO to the carbon–carbon double bond is abundant: over 1000 publications, more than 500 patents, and several books. That is due to the industry’s increasing interest in this group, as mentioned in the introduction. It might seem that virtually any design of isoxazoline can be synthesized because appropriate procedures have been described for structurally similar compounds. However, e.g., cycloaddition between sterically crowded dipoles and dipolarophiles encounters difficulties. Our experience shows that an excellent, very effective solution for reactions between sterically demanding reagents is to conduct the reaction under high pressure (>1 GPa) [240,245]. However, there are only a few syntheses of isoxazolines under high pressure described or patented, probably due to the limited availability of equipment for carrying out the reaction under hp. Thus, 1,3-DP cycloaddition under hp is a promising, even guaranteed success method that should be developed further. The activation volume for cycloaddition reactions, including 1,3-DP reactions, is strongly negative, which is well-known [246,363].

The use of transition metal complexes or transition metals immobilized on various surfaces as catalysts in synthesizing isoxazolines is problematic due to limiting the content of transition metals in products of pharmacological importance [364]. It is possible to use commercially available scavengers to remove metal traces, and this is effective for many catalytic processes, e.g., hydrogenation coupling and others [365,366,367,368]. Due to the lack of or minimal harmfulness, magnesium compounds (including complexes with chiral ligands) seem to be particularly promising.

However, the most critical challenge is the synthesis of chiral isoxazolines, in particular, optically pure isoxazolines possessing not one but two stereogenic centers (strictly defined configuration on the carbon C-2 and C-3). The importance of asymmetric synthesis concerning isoxazolines is obviously due to the biological potential of chiral isoxazolines, i.e., the possibility of using them as drugs, antifungal agents, antibacterials, anticancer agents, and others. As is known, pure enantiomers are required, not mixtures of all isomers. This review presents several spectacular successes in optical pure isoxazoline synthesis: using chiral auxiliaries, chiral ligands, enzymatic resolutions, chiral natural dipolarophiles, and others.

Moreover, further development of asymmetric synthesis with isoxazolines is essential. That applies to the 1,3-DP cycloaddition of RCNO into the carbon–carbon double bond, which is the subject of this review and, perhaps, primarily, to other methods. The analysis of progress in the synthesis of isoxazolines indicates that the role of procedures other than dipolar cycloaddition for the synthesis of these compounds is increasing—see Section 6. The spectacular successes of the synthesis of isoxazolines from nitronates, nitro compounds, unsaturated oximes, Cu or Pd catalysts, and others show that alternatives are available. In the case of 1,3-DP cycloaddition, the methods provide opportunities that the mentioned 1,3-DP cycloaddition does not.

The mechanism of RCNO 1,3-DP cycloaddition to the carbon–carbon double bond is well-known and described. Each review of isoxazolines comprises an introduction devoted to the mechanistic aspect of 1,3-DP cycloaddition, including considerations from the quantum chemistry point of view. However, there are insufficient studies on applying theoretical calculations to predict the regio- and stereoselectivity of cycloaddition. Our research shows that the DFT calculations explain the regioselectivity observed for the ArCNO cycloaddition to QCH=CHMe [201,240]. It should be noted, however, that in the latest works, there is practically always an element of chemistry in silico, including detailed mechanistic analysis using the results of DFT calculations. Undoubtedly, the increase in the importance of chemistry in silico for designing structures of target compounds is necessary along with the development of quantum chemistry.

The analysis of the state of knowledge indicates that the most critical challenge for the synthesis of isoxazolines is the design of their structures for specific applications as drugs, plant protection agents, and insecticides, generally due to their expected selective action on molecular objects. The vast majority of publications concern the biological activity of this group of compounds and, more precisely, their impact on molecular objects (DNA, receptors). Therefore, the problem is not how to obtain a given isoxazoline, but what its structure should be due to the expected biological effect. That is the general challenge facing organic synthesis, particularly the synthesis of compounds that are supposed to interact selectively with a receptor coming from a living organism. When it comes to isoxazolines, it seems that obtaining isoxazolines with any planned structure is possible today. The spectrum of available synthesis methods is vast and constantly expanding. The problem is chemo-, regio-, and stereoselectivity, especially for highly complex structures. Thus, that is the most crucial challenge for the synthesis of isoxazolines.

Undoubtedly, it is also essential to better understand drug molecule–receptor interactions, which also applies to isoxazolines. However, the issue of the biological activity of isoxazolines was not widely discussed in this review, although it was emphasized. Therefore, we leave defining the challenges in this area to specialists.

## 9. Conclusions

This review presents state-of-the-art syntheses of 2-isoxazolines via 1,3-dipolar cycloaddition of nitrile oxide to the carbon–carbon double bond starting from the year 2000. Moreover, double bond migration and metathesis in dipolarophile acquisition and various nitrile oxide preparation and in situ generation were shown. Special attention was paid to the application of multiple combinations of dipolar cycloaddition (DPC) with double bond migration (DBM) and with alkene metathesis (AM) in the syntheses of trisubstituted isoxazolines. Allyl compounds of the type QCH_2_CH=CH_2_ (Q = ArO, ArS, Ar, and others) play the role of dipolarophile precursors in the above-mentioned combinations of DPC, DBM, and AM. It is easy to see that the literature devoted to synthesizing isoxazolines via 1,3-DP cycloaddition of RCNO to R^1^R^1^C=CR^3^R^4^ is robust. That also applies to patents owned by large corporations, such as ASTRA-Zeneca, Pfizer, Merck, and others, which demonstrates the practical importance of this class of compounds. Notably, the constant development of the topic of “isoxazolines—synthesis and applications” is observed, which manifests as an increase in the number of publications and, above all, the number of patents. Regarding synthesis, the leading role is played by the RCNO 1,3-DP cycloaddition to the carbon–carbon double bond, which is the subject of this review. However, many other methods of synthesizing isoxazolines are also (briefly) presented in this article. Moreover, some of them allow obtaining isoxazolines unattainable by 1,3-DP cycloaddition. Undoubtedly, a gradual increase in the importance of methods other than simple 1,3-DP cycloaddition for the synthesis of isoxazolines is observed—in particular, methods starting from nitro compounds, nitronates, simple oximes, unsaturated and oxo-oximes, organometallics, those involving transition metal complexes, including chiral ones, and electrochemical methods. However, when it comes to isoxazolines used in pharmacy, agriculture, and animal and human medicine, 1,3-DP cycloaddition of RCNO into the carbon–carbon double bond remains the leading method. Importantly, DPC is also often a crucial step in the total synthesis of natural compounds—this review also discusses this topic. The importance of isoxazolines stems from many being of natural origin, and new ones are constantly being isolated from biological materials. Mechanistic aspects of cycloadditions, i.e., concerted or stepwise reaction mechanism and their regio- and stereoselectivity, are also concisely discussed. That is because, in many reviews and books already devoted to isoxazolines, mechanistic aspects were analyzed from the quantum chemistry point of view. Undoubtedly, with the development of the theoretical chemistry mechanism and all aspects of regio- and stereoselectivity of DPC, they are commented on more deeply. Side reactions accompanying cycloaddition, especially nitrile oxide dimerization, are considered. RCNO dimerization into furoxanes is a severe limitation, yet some furoxanes are attractive for pharmacy, being a target for synthesis. However, the generation of RCNO in situ allows for the realization of cycloaddition of unstable nitrile oxides. 2-Isoxazoline applications in organic synthesis and their pharmacological evaluation were also raised. When it comes to applications in organic synthesis, they are briefly presented, but it can be seen that the role of isoxazolines in organic synthesis, e.g., the synthesis of amino acids, aminoalcohols, lactams, and isoxazoles, to name a few, is significant. The biological activity of isoxazolines shown during the synthesis presentation is significant for all discussed publications and patents. It is easy to notice that the introduction of practically all publications concerning isoxazolines contains numerous references to their extensive biological activity. Finally, we conclude with a quote that perfectly captures the meaning of isoxazolines: “Isoxazolines are the animal health industry’s most successful molecules to date…” (from Dr. S. Juneja [1]).

## Abbreviations

1,3-DP	1,3-dipolar
AcOEt	ethyl acetate
ADMET	absorption, distribution, metabolism, excretion, and toxicity
AM	alkene-metathesis
AMPA	α-amino-3-hydroxy-5-methyl-4-isoxazolepropionic acid
AMPAR	α-amino-3-hydroxy-5-methyl-4-isoxazolepropionic acid receptors
ANOC	acyclic nitronate olefin cycloaddition
BACE1	β-site APP cleaving enzyme-1
BARAC	biarylazacyclooctynone
BINIM	binaphthyldiimine ligand
BINOL	1,1′-bi-2-naphthol
BMIM	1-butyl-3-meth-ylimidazolium cation
BNO	benzoyl hydroxamic acid
Boc	tert-butyloxycarbonyl group
BODIPY	4,4-difluoro-4-bora-3a,4a-diaza-s-indacene
BTMA	benzyltrimethylammonium group
CAN	cerium(IV) ammonium nitrate
CD-Dip	dipolarophile tethered to cyclodextrin
cod	cyclooctadiene
COX	cyclooxygenase
CPBA	chloroperoxybenzoic acid
CPVBIm	cross-linked poly-1-(4-vinylbenzyl)imidazole
CPVP	cross-linked poly-4-vinylpyridine
DABCO	1,4-diazabicyclo[2.2.2]octane
DAP	diaminopimelic acid analogous
dba	dibenzylideneacetone
DBM	double bond migration
DBU	1,8-diazabicyclo(5.4.0)undec-7-ene
DCE	dichloroethane
DCM	dichloromethane
de	diastereomeric excess
DFT	density functional theory
DIB	(diacetoxyiodo)benzene
DIDMH	1,3-diiodo-5,5-dimethylhydantoin
DIPEA	*N*,*N*-diisopropylethylamine
DIPT	diisopropyl tartrate
DMAP	4-dimethylaminopyridine
DME	dimethoxyethane
DMF	dimethylformamide
DMSO	dimethyl sulfoxide
DNA	deoxyribonucleic acid
DNMT1	DNA methyltransferase 1
DPC	dipolar cycloaddition
DPPH	1,1-diphenyl-2-picryl-hydrazyl
dr	diastereomeric ratio
DVB	divinylbenzene
ee	enantiomeric excess
EP	european patent
er	enantiomeric ratio
EWG	electron withdrawing group
FMO	frontier molecular orbital
Free-Dip	free dipolarophfile
FtsZ	filamentous temperature-sensitive mutant Z
*g*-C_3_N_4_	graphitic carbon nitride
GABACls	γ-aminobutyric acid-gated chloride channels
GP	glycoprotein
GPb	glycogen phosphorylase
HAP	hydroxyapatite
hCA	human carbonic anhydrase
HDAC	histone deacetylase
HFIP	hexafluoroisopropanol
HOMO	highest occupied molecular orbital
HPLC	high-performance liquid chromatography
HTIB	[hydroxy(tosyloxy)iodo]benzene
HTPI	hydroxytelechelic cis-1,4-polyisoprene
huMIF	human migration inhibitory factor
IC 50	half-maximal inhibitory concentration
ICl	iodine monochloride
INOC	intramolecular nitrile oxide 1,3-dipolar cycloaddition
iNOS	inducible nitric oxide synthase
LA	Lewis acid
LDA	lithium diisopropylamide
LED	light-emitting diode
LO	lipoxygenas
LOL	localized orbital localizer
LUMO	lowest unoccupied molecular orbital
*m*-Ddh	meso-diaminopimelate dehydrogenase
MCD	malonyl-coenzyme A decarboxylase
MDR	multiple drug-resistant
MEDT	molecular electron density theory
MEM	2-methoxyethoxymethyl group
MEP	electrostatic potential map
Mes	mesityl
MesCNO	2,4,6-trimethylbenzonitrile
MIC	minimal inhibitory concentration
MIF	migration inhibitory factor
MOM	methoxymethyl group
MPM	methoxybenzyl group
MS	molecular sieves
Mtb	mycobacterium tuberculosis
MTBE	methyl tert-butyl ether
MW	microwaves
MXT	mitoxantrone
nAChRs	neuronal nicotinic acetylcholine receptors
NBAc	*N*-bromoacetamide
NBS	*N*-bromosuccinimide
NCS	*N*-chlorosuccinimide
NIS	*N*-iodosuccinimid
NMDA	*N*-methyl-D-aspartic acid
NMO	*N*-methylmorpholine *N*-oxide
NMR	nuclear magnetic resonance
NRK	normal rat kidney
OTf	triflate group
OXONE	potassium peroxymonosulfate
PEG	poly(ethylene glycol)
PIDA	phenyliodine(III) diacetate
Piv	pivaloyl group
PMB	p-methoxybenzyl group
PMDETA	*N*,*N*,*N*’,*N*″,*N*″-pentamethyldiethylenetriamine
PMMA	poly(methyl methacrylate)
POSS	polyhedral oligomeric silsesquioxane
PS	polystyrene
PSA	polar surface area
PTC	phase-transfer catalysis
PTSA	p-toluenesulfonic acid
Py	pyridine
RCC	radical-carbene coupling
SAHA	suberoylanilide hydroxamic acid
SAR	structure-activity relationship
scCO_2_	supercritical carbon dioxide
SDS	sodium dodecylsulfate
SmA	smectic A mesophase
SS	stainless steel electrode
STREM	metals scavenging agent
t-Am	tert-amyl group
TBAF	tetra-n-butylammonium fluoride
TBDMS	tert-butyldimethylsilyl group
TBDPS	tert-butyldiphenylsilyl group
TBN	tert-butyl nitrite
TBS	tert-butyldimethylsilyl group
TBTO	tributyltin oxide
TEMPO	2,2,6,6-tetramethylpiperidin-1-oxyl
TFE	2,2,2-trifluoroethanol
THF	tetrahydrofuran
THP	tetrahydropyranyl group
TIPS	triisopropylsilyl group
TMDS	1,3,3-tetramethyldisiloxane
TMEDA	tetramethylethylenediamine
TMS	trimethylsilyl group
Tos	tosyl group
TS	transition state
XP	extra-precision
